# Supplementary figures and images for: Ancestral Reconstructions Decipher Major Adaptations of Ammonia-Oxidizing Archaea upon Radiation into Moderate Terrestrial and Marine Environments
Source: mBio. 2020 Oct 13;11(5):e02371-20. doi: 10.1128/mBio.02371-20 (PMC7554672; doi:10.1128/mBio.02371-20)

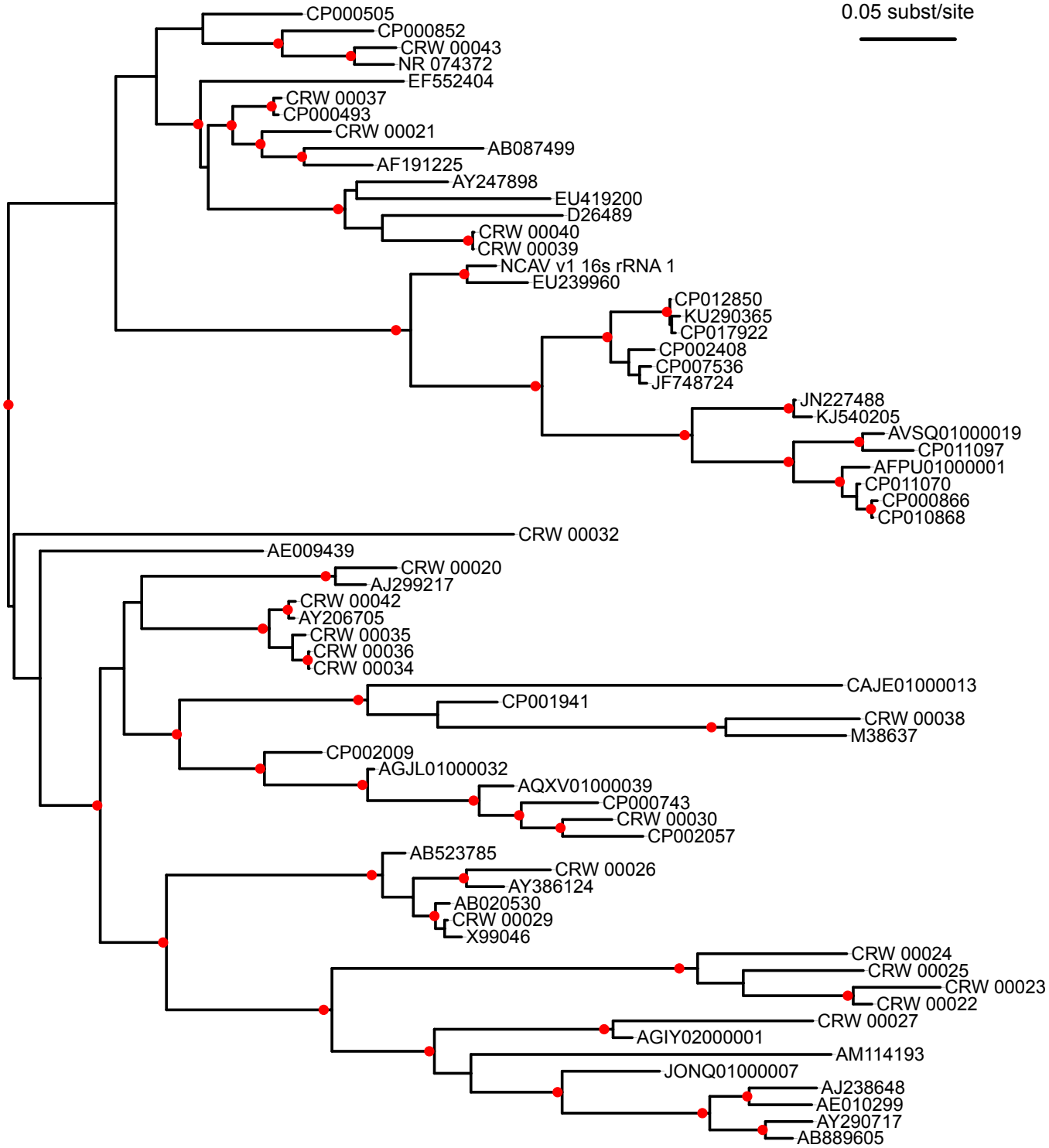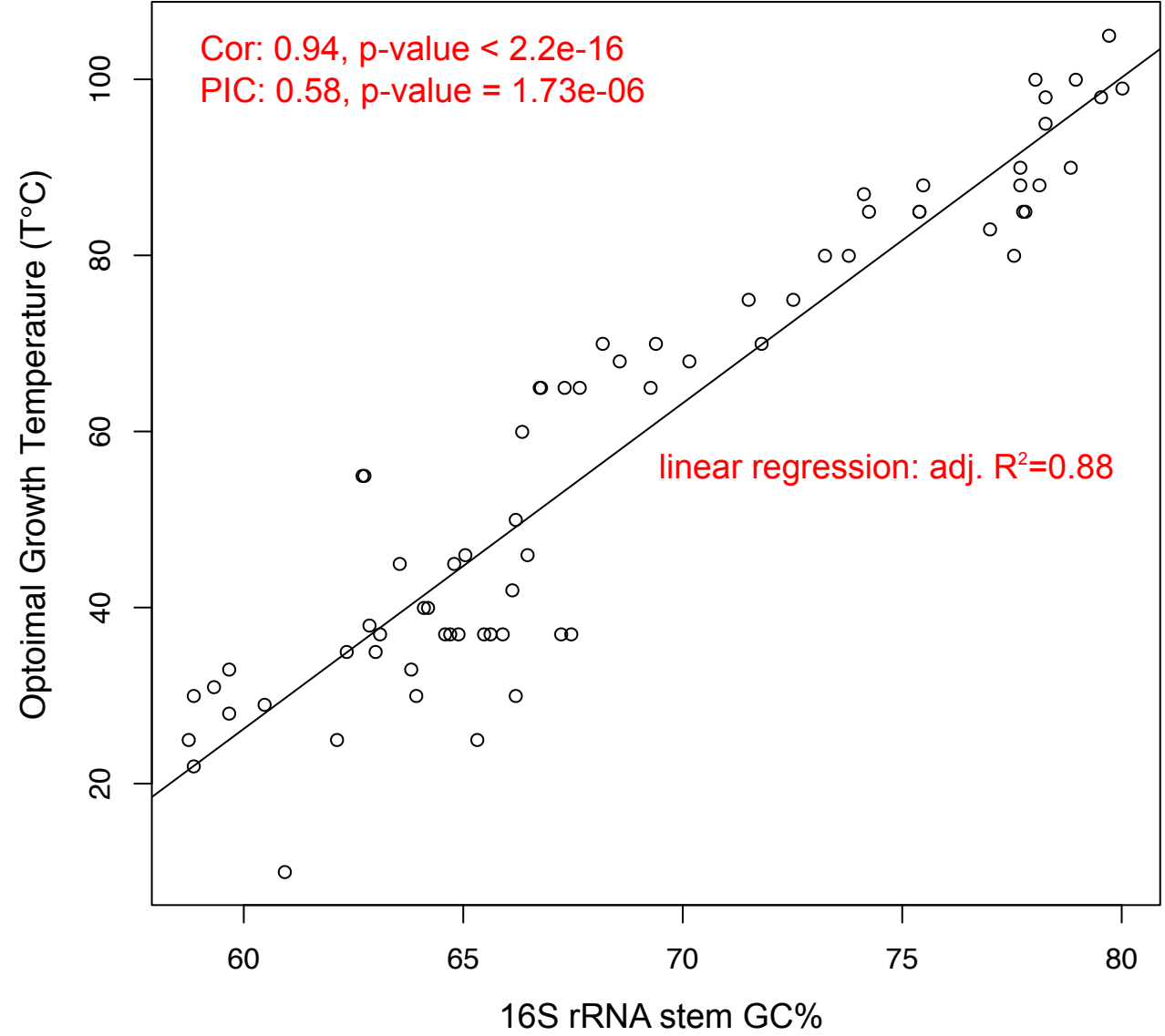

Supplement: FIG S1 [file mBio.02371-20-sf001.pdf]

Tree scale: 0.1

families in extant genomes

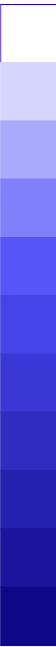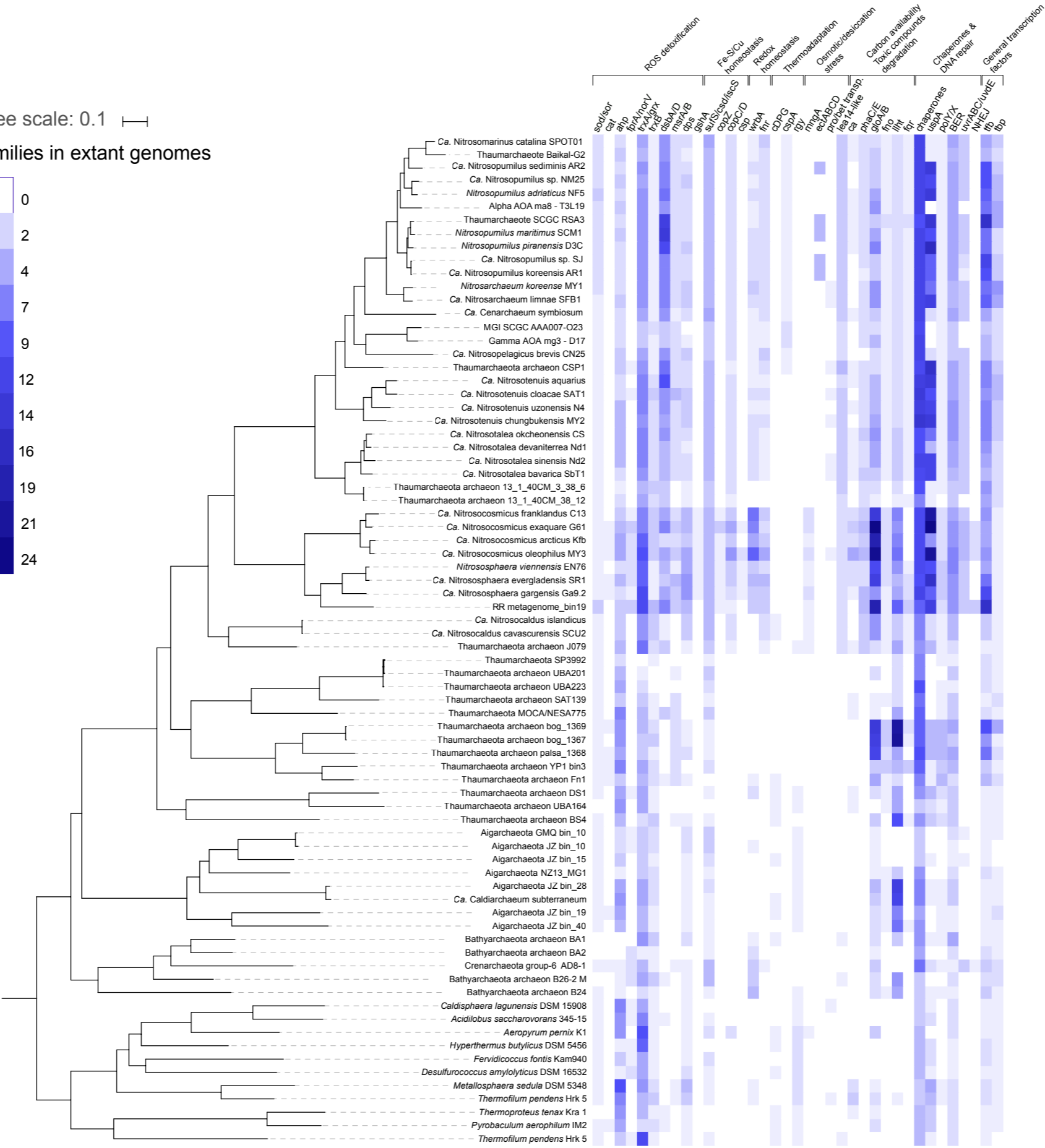

Supplement: FIG S3 [file mBio.02371-20-sf003.pdf]

0.3 subst/site

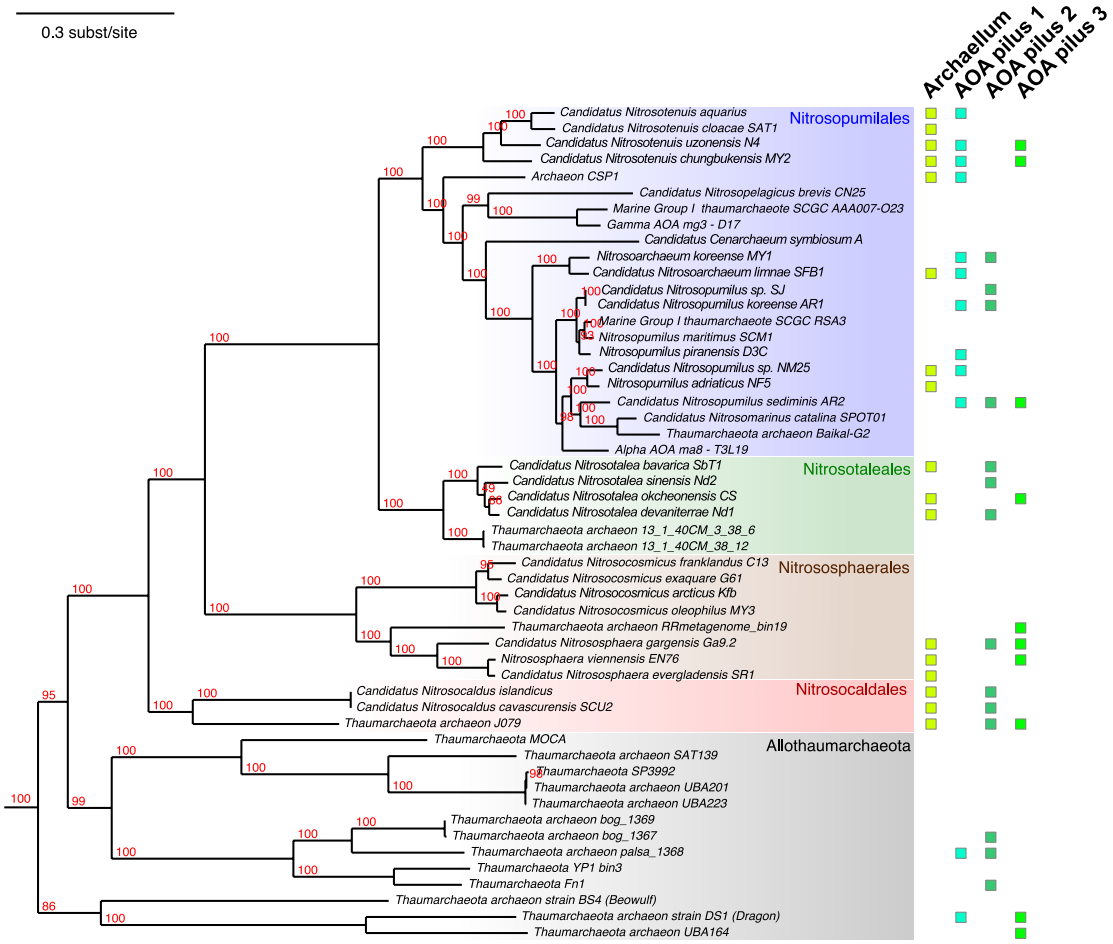

Supplement: FIG S4 [file mBio.02371-20-sf004.pdf]
