# Supplementary material for: Ancestral Reconstructions Decipher Major Adaptations of Ammonia-Oxidizing Archaea upon Radiation into Moderate Terrestrial and Marine Environments
Source: mBio. 2020 Oct 13;11(5):e02371-20. doi: 10.1128/mBio.02371-20 (PMC7554672; doi:10.1128/mBio.02371-20)
Supplement: FIG S2 [file mBio.02371-20-sf002.pdf]

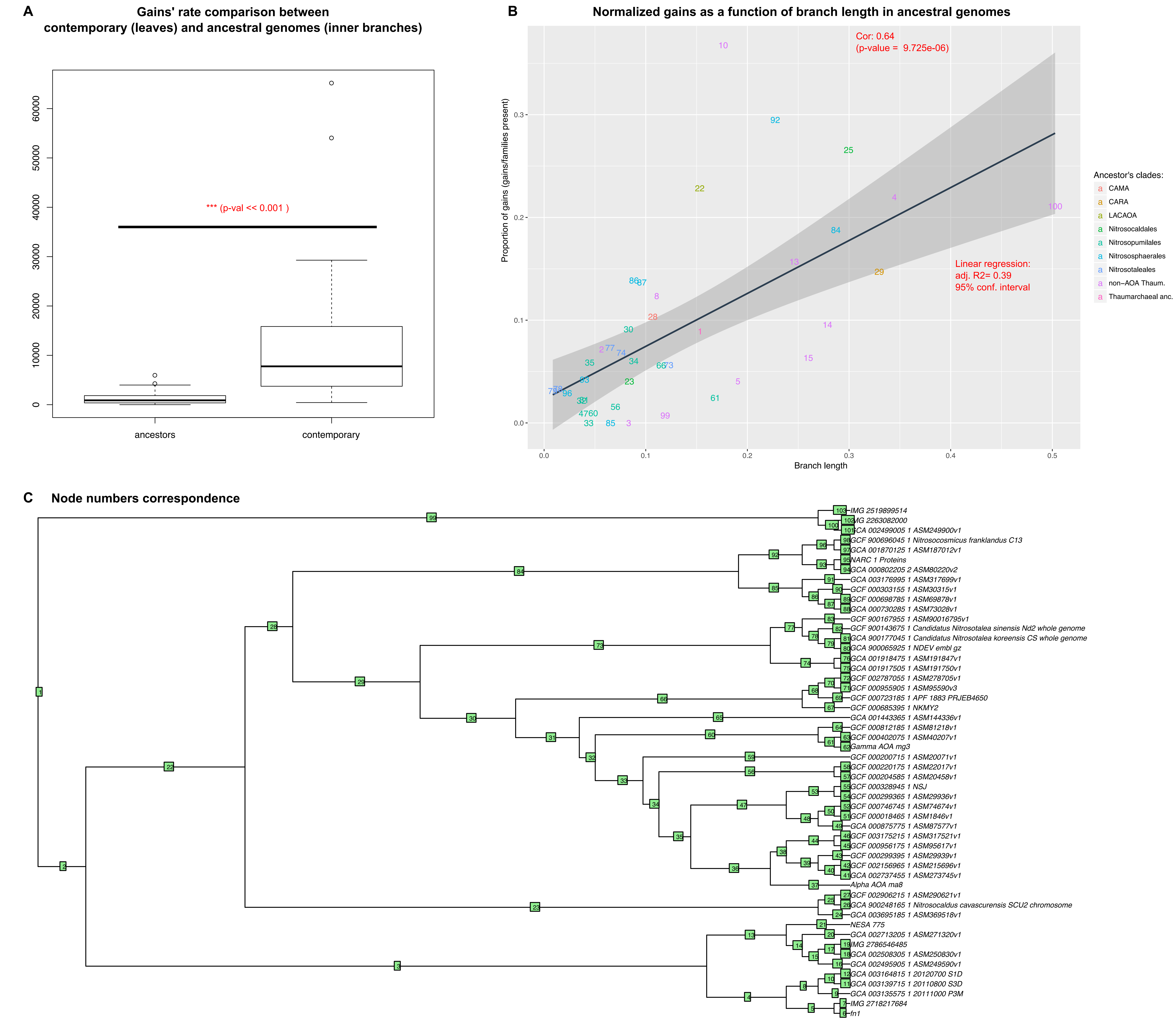
A
Gains' rate comparison between
contemporary (leaves) and ancestral genomes (inner branches)
*** (p-val << 0.001)
ancestors
contemporary
B
Normalized gains as a function of branch length in ancestral genomes
Cor: 0.64
(p-value = 9.725e-06)
Linear regression:
adj. R2= 0.39
95% conf. interval
Proportion of gains (gains/families present)
Branch length
Ancestor's clades:
CAMA
CARA
LACAOA
Nitrosocaldales
Nitrosopumilales
Nitrososphaerales
Nitrosotaleales
non−AOA Thaum.
Thaumarchaeal anc.
C
Node numbers correspondence
IMG 2519899514
IMG 2263082000
GCA 002499005 1 ASM249900v1
GCF 900696045 1 Nitrosocosmicus franklandus C13
GCA 001870125 1 ASM187012v1
NARC 1 Proteins
GCA 000802205 2 ASM80220v2
GCA 003176995 1 ASM317699v1
GCF 000303155 1 ASM30315v1
GCF 000698785 1 ASM69878v1
GCA 000730285 1 ASM73028v1
GCF 900167955 1 ASM90016795v1
GCF 900143675 1 Candidatus Nitrosotalea sinensis Nd2 whole genome
GCA 900177045 1 Candidatus Nitrosotalea koreensis CS whole genome
GCA 900065925 1 NDEV embl gz
GCA 001918475 1 ASM191847v1
GCA 001917505 1 ASM191750v1
GCF 002787055 1 ASM278705v1
GCF 000955905 1 ASM95590v3
GCF 000723185 1 APF 1883 PRJEB4650
GCF 000685395 1 NKMY2
GCA 001443365 1 ASM144336v1
GCF 000812185 1 ASM81218v1
GCF 000402075 1 ASM40207v1
Gamma AOA mg3
GCF 000200715 1 ASM20071v1
GCF 000220175 1 ASM22017v1
GCF 000204585 1 ASM20458v1
GCF 000328945 1 NSJ
GCF 000299365 1 ASM29936v1
GCF 000746745 1 ASM74674v1
GCF 000018465 1 ASM1846v1
GCA 000875775 1 ASM87577v1
GCF 003175215 1 ASM317521v1
GCF 000956175 1 ASM95617v1
GCF 000299395 1 ASM29939v1
GCF 002156965 1 ASM215696v1
GCA 002737455 1 ASM273745v1
Alpha AOA ma8
GCF 002906215 1 ASM290621v1
GCA 900248165 1 Nitrosocaldus cavascurensis SCU2 chromosome
GCA 003695185 1 ASM369518v1
NESA 775
GCA 002713205 1 ASM271320v1
IMG 2786546485
GCA 002508305 1 ASM250830v1
GCA 002495905 1 ASM249590v1
GCA 003164815 1 20120700 S1D
GCA 003139715 1 20110800 S3D
GCA 003135575 1 20111000 P3M
IMG 2718217684
fn1
