# Supplementary material for: Ancestral Reconstructions Decipher Major Adaptations of Ammonia-Oxidizing Archaea upon Radiation into Moderate Terrestrial and Marine Environments
Source: mBio. 2020 Oct 13;11(5):e02371-20. doi: 10.1128/mBio.02371-20 (PMC7554672; doi:10.1128/mBio.02371-20)
Supplement: DATA SET S1 [file mBio.02371-20-sd001.pdf]

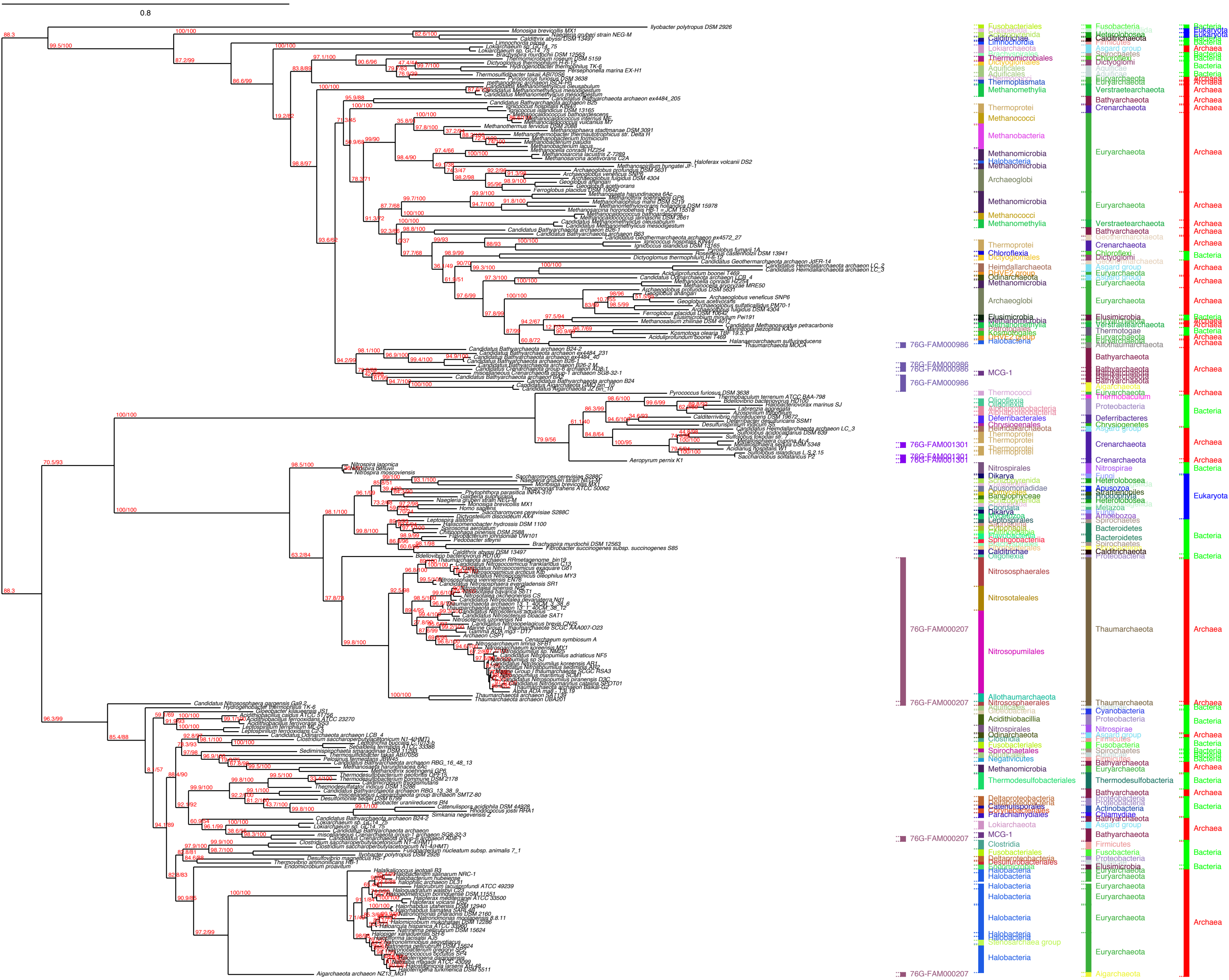
0.8
76G-FAM000986
MCG-1
76G-FAM001301
76G-FAM000207
Nitrososphaerales
Nitrosotaleales
Nitrosopumilales
Thaumarchaeota
Euryarchaeota
Halobacteria
Methanomicrobia
Archaeoglobi
Bathyarchaeota
Crenarchaeota
Eukaryota
Archaea
Bacteria

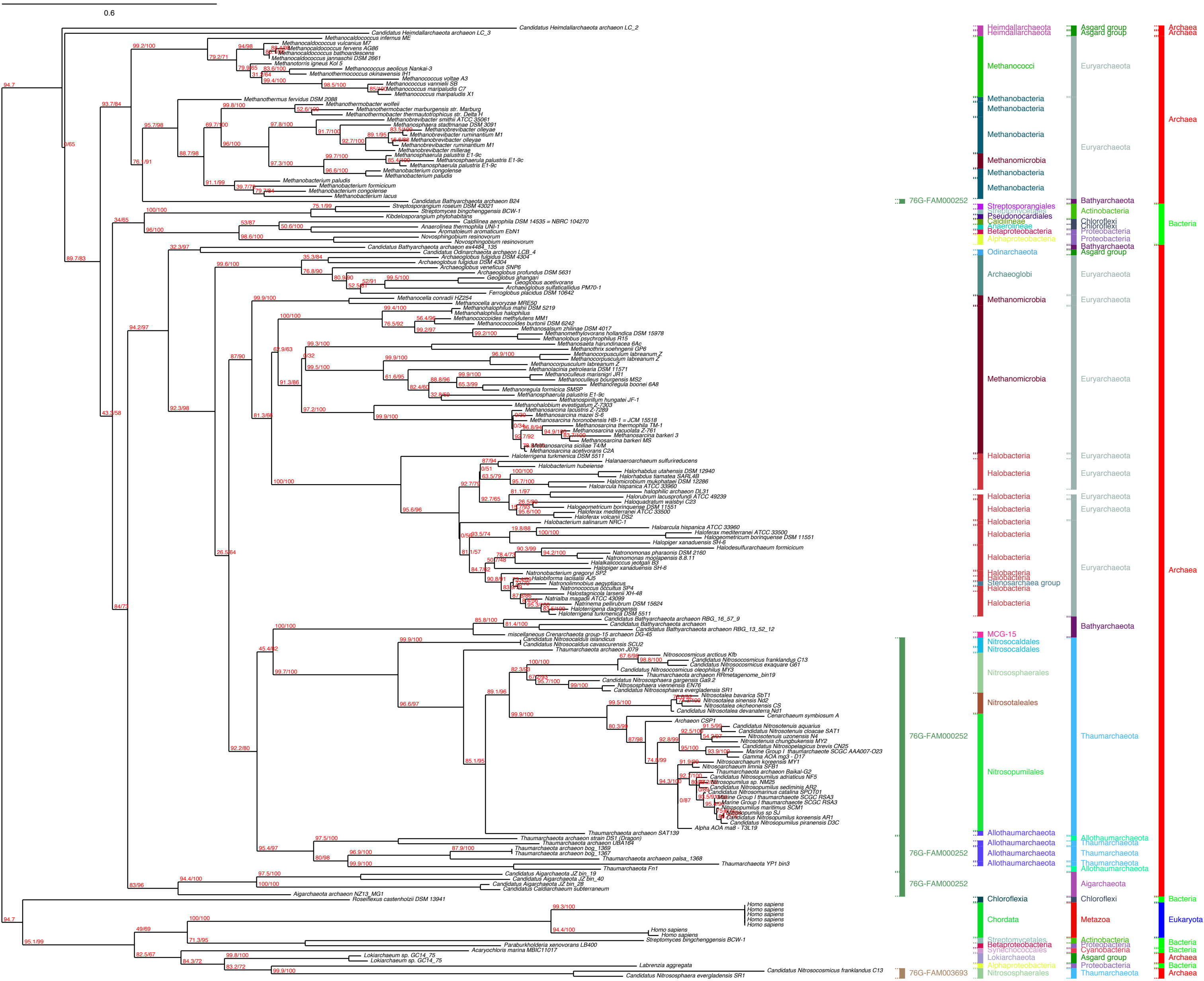
0.6
Heimdallarchaeota
Methanococci
Methanobacteria
Methanomicrobia
76G-FAM000252
Streptosporangiales
Streptomycetales
Pseudonocardiales
Caldilineae
Anaerolineae
Betaproteobacteria
Alphaproteobacteria
Odinarchaeota
Archaeoglobi
Halobacteria
Stenosarchaea group
MCG-15
Nitrosocaldales
Nitrososphaerales
Nitrosotaleales
Nitrosopumilales
Allothaumarchaeota
Chloroflexia
Chordata
Synechococcales
Lokiarchaeota
76G-FAM003693
Asgard group
Euryarchaeota
Bathyarchaeota
Actinobacteria
Chloroflexi
Proteobacteria
Thaumarchaeota
Aigarchaeota
Metazoa
Cyanobacteria
Archaea
Bacteria
Eukaryota

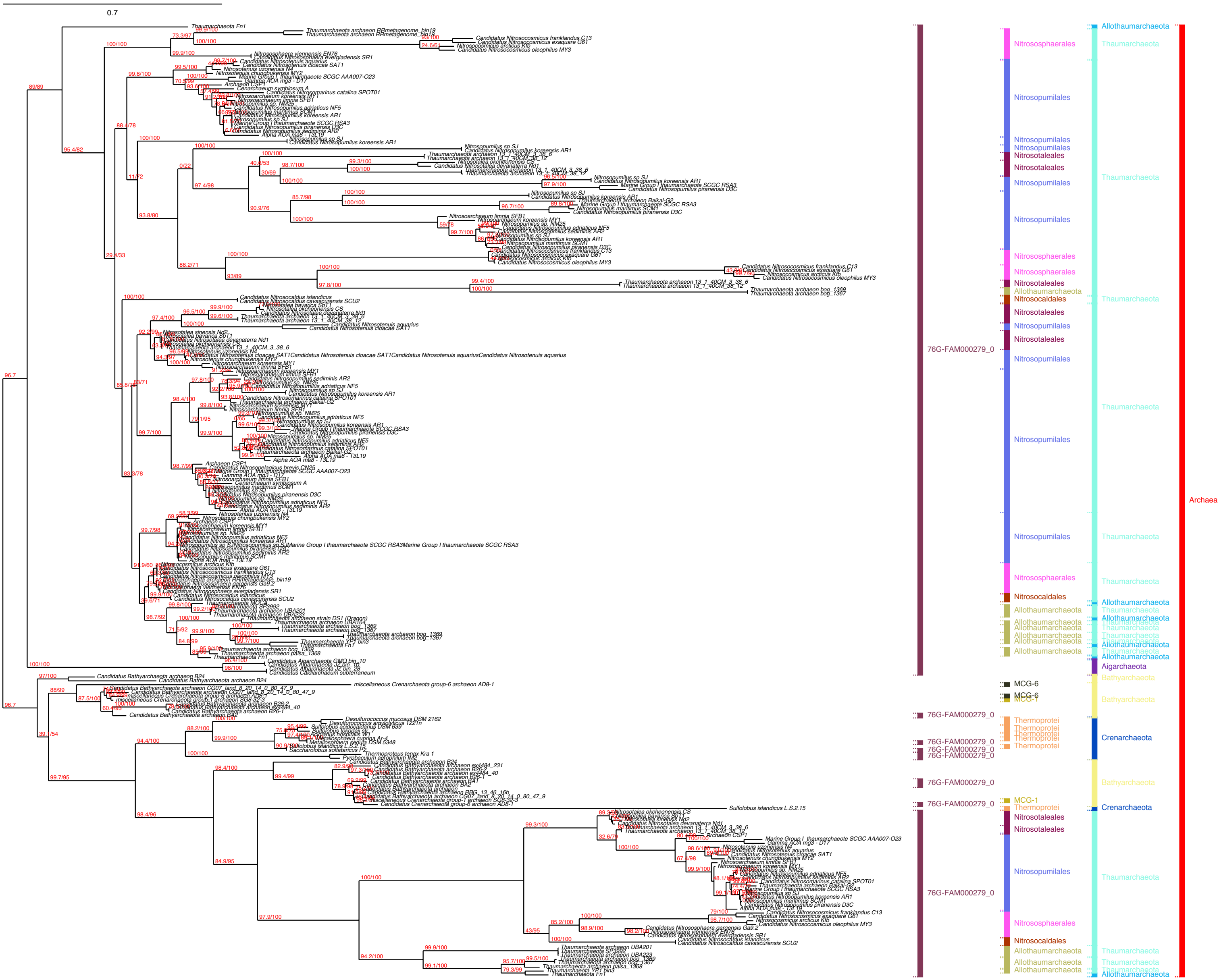

0.7
76G-FAM000279_0
Nitrososphaerales
Nitrosopumilales
Nitrosotaleales
Nitrosocaldales
Allothaumarchaeota
Thaumarchaeota
MCG-6
MCG-1
Thermoprotei
Aigarchaeota
Bathyarchaeota
Crenarchaeota
Archaea

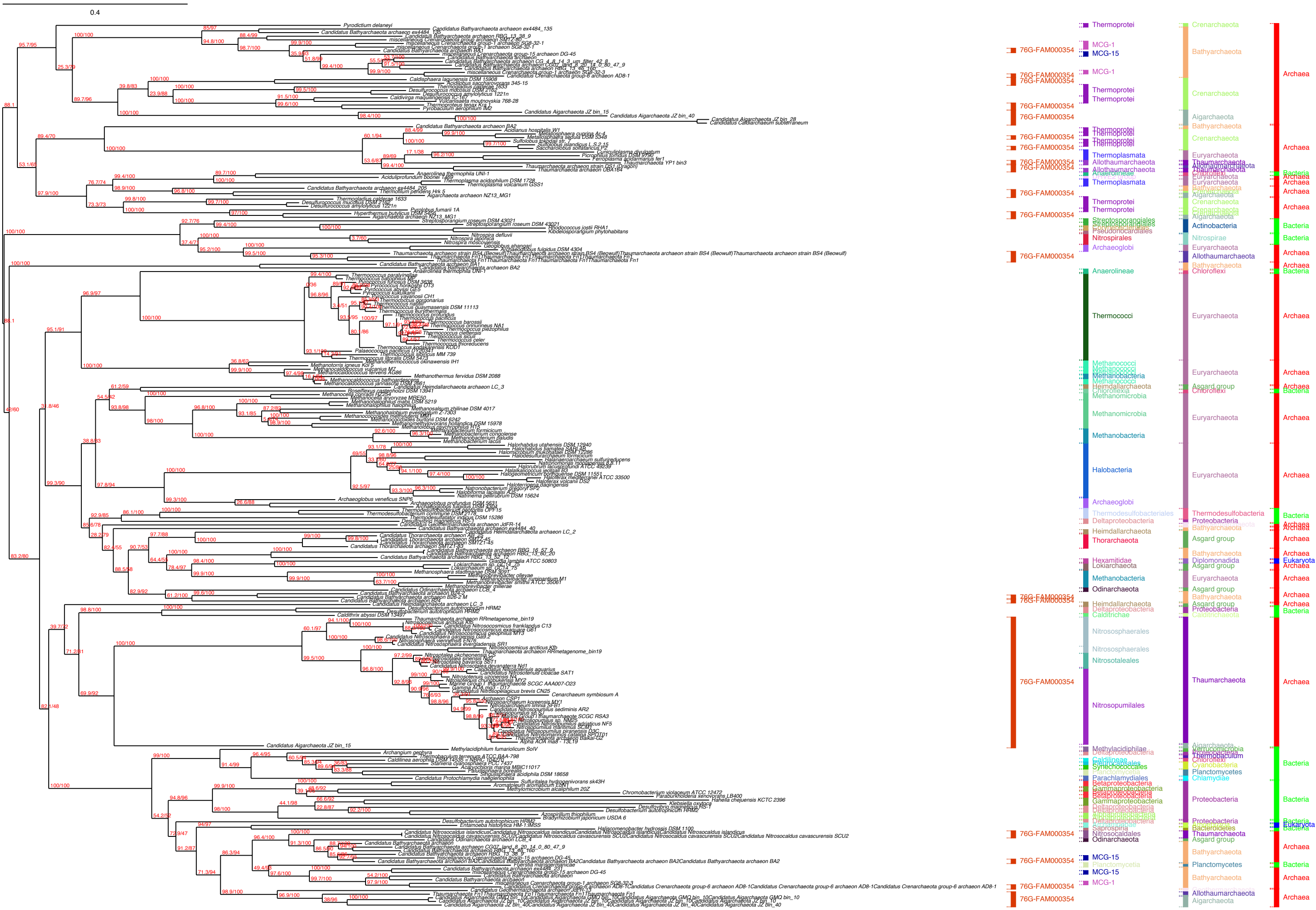
0.4
76G-FAM000354
Thermoprotei
MCG-1
MCG-15
Thermoplasmata
Allothaumarchaeota
Anaerolineae
Streptosporangiales
Pseudonocardiales
Nitrospirales
Archaeoglobi
Thermococci
Methanococci
Methanobacteria
Heimdallarchaeota
Chloroflexi
Methanomicrobia
Halobacteria
Thermodesulfobacteriales
Deltaproteobacteria
Thorarchaeota
Hexamitidae
Lokiarchaeota
Odinarchaeota
Caldithrix
Nitrososphaerales
Nitrosotaleales
Nitrosopumilales
Methylacidiphilae
Caldilineae
Synechococcales
Parachlamydiales
Betaproteobacteria
Gammaproteobacteria
Saprospiria
Nitrosocaldales
Crenarchaeota
Bathyarchaeota
Aigarchaeota
Euryarchaeota
Thaumarchaeota
Actinobacteria
Nitrospirae
Asgard group
Thermodesulfobacteria
Proteobacteria
Diplomonadida
Verrucomicrobia
Thermoplasmata
Cyanobacteria
Planctomycetes
Chlamydiae
Bacteroidetes
Archaea
Bacteria
Eukaryota

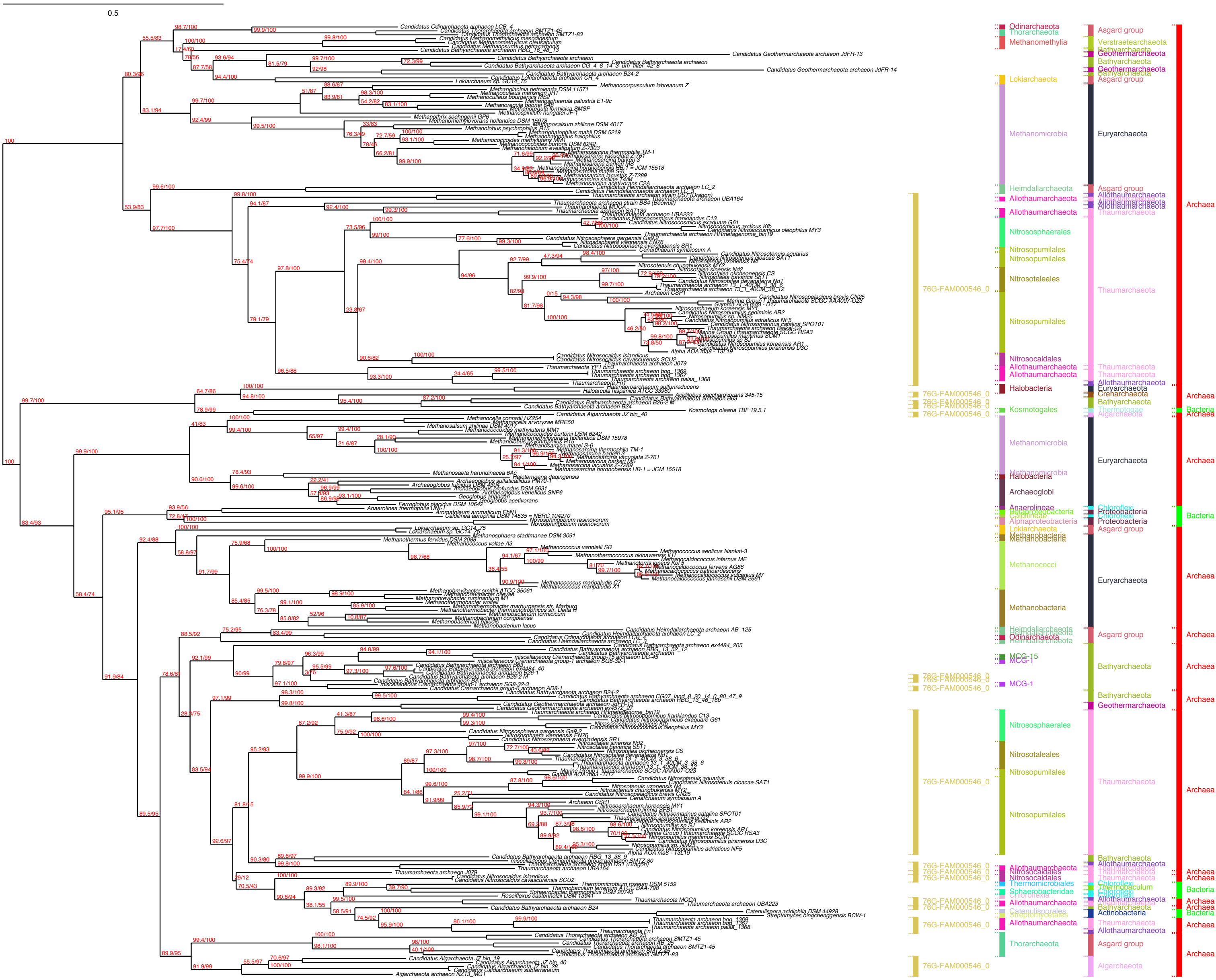
0.5
Odinarchaeota
Thorarchaeota
Methanomethylia
Lokiarchaeota
Methanomicrobia
Heimdallarchaeota
Allothaumarchaeota
Nitrososphaerales
Nitrosopumilales
Nitrosotaleales
Nitrosocaldales
Halobacteria
Kosmotogales
Archaeoglobi
Anaerolineae
Caldilineae
Betaproteobacteria
Alphaproteobacteria
Methanobacteria
Methanococci
MCG-15
MCG-1
Thermomicrobiales
Sphaerobacteridae
Chloroflexia
Catenulisporales
Streptomycetales
76G-FAM000546_0
Asgard group
Verstraetearchaeota
Bathyarchaeota
Geothermarchaeota
Euryarchaeota
Thaumarchaeota
Crenarchaeota
Thermotogae
Aigarchaeota
Chloroflexi
Proteobacteria
Actinobacteria
Archaea
Bacteria

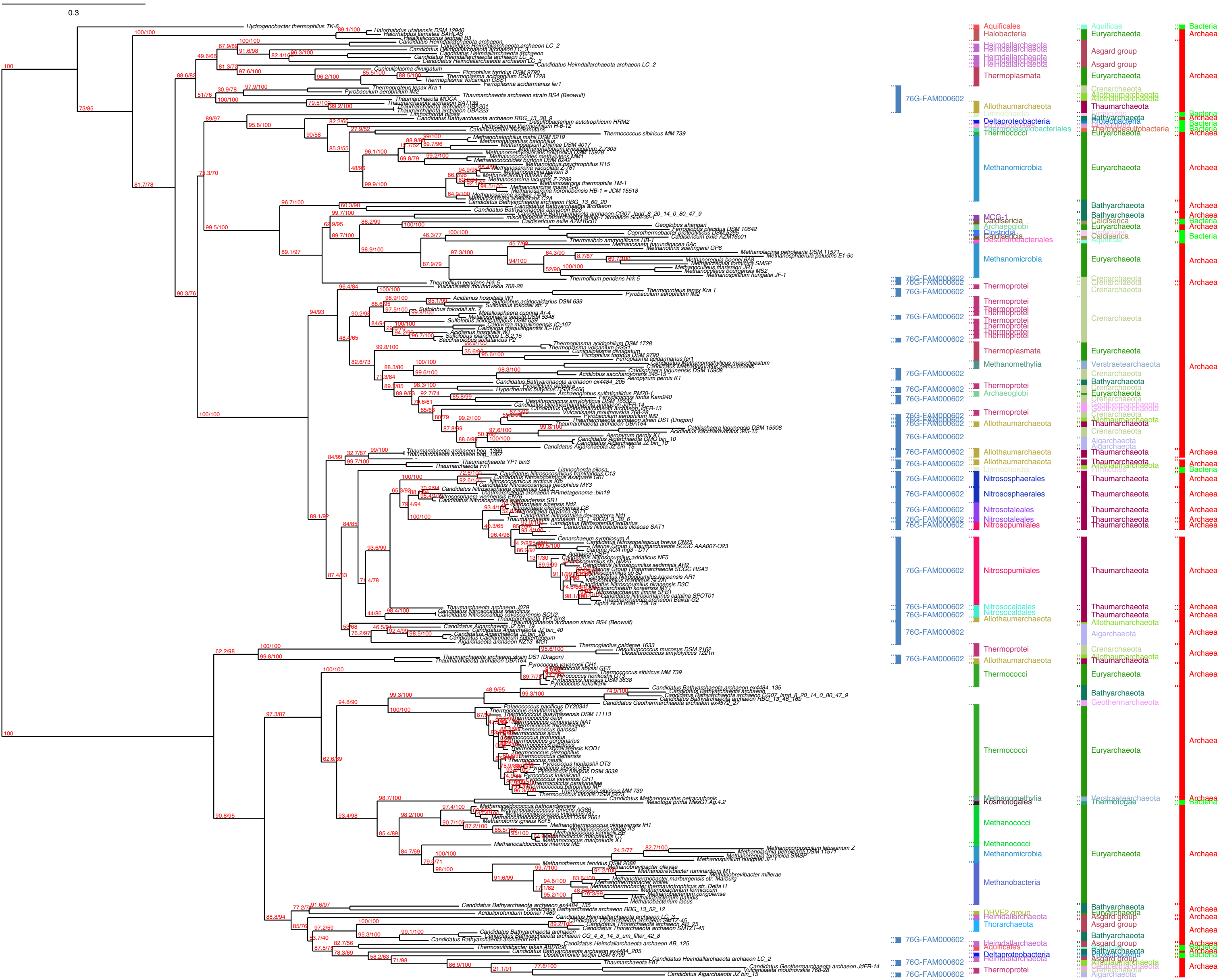

0.3
Aquificales
Halobacteria
Heimdallarchaeota
Thermoplasmata
76G-FAM000602
Allothaumarchaeota
Deltaproteobacteria
Thermococci
Methanomicrobia
MCG-1
Caldiserica
Archaeoglobi
Clostridia
Desulfurobacteriales
Thermoprotei
Methanomethylia
Nitrososphaerales
Nitrosotaleales
Nitrosopumilales
Nitrosocaldales
Kosmotogales
Methanococci
Methanobacteria
DHVE2 group
Thorarchaeota
Aquificae
Euryarchaeota
Asgard group
Crenarchaeota
Thaumarchaeota
Bathyarchaeota
Proteobacteria
Thermodesulfobacteria
Verstraetearchaeota
Geothermarchaeota
Aigarchaeota
Thermotogae
Bacteria
Archaea

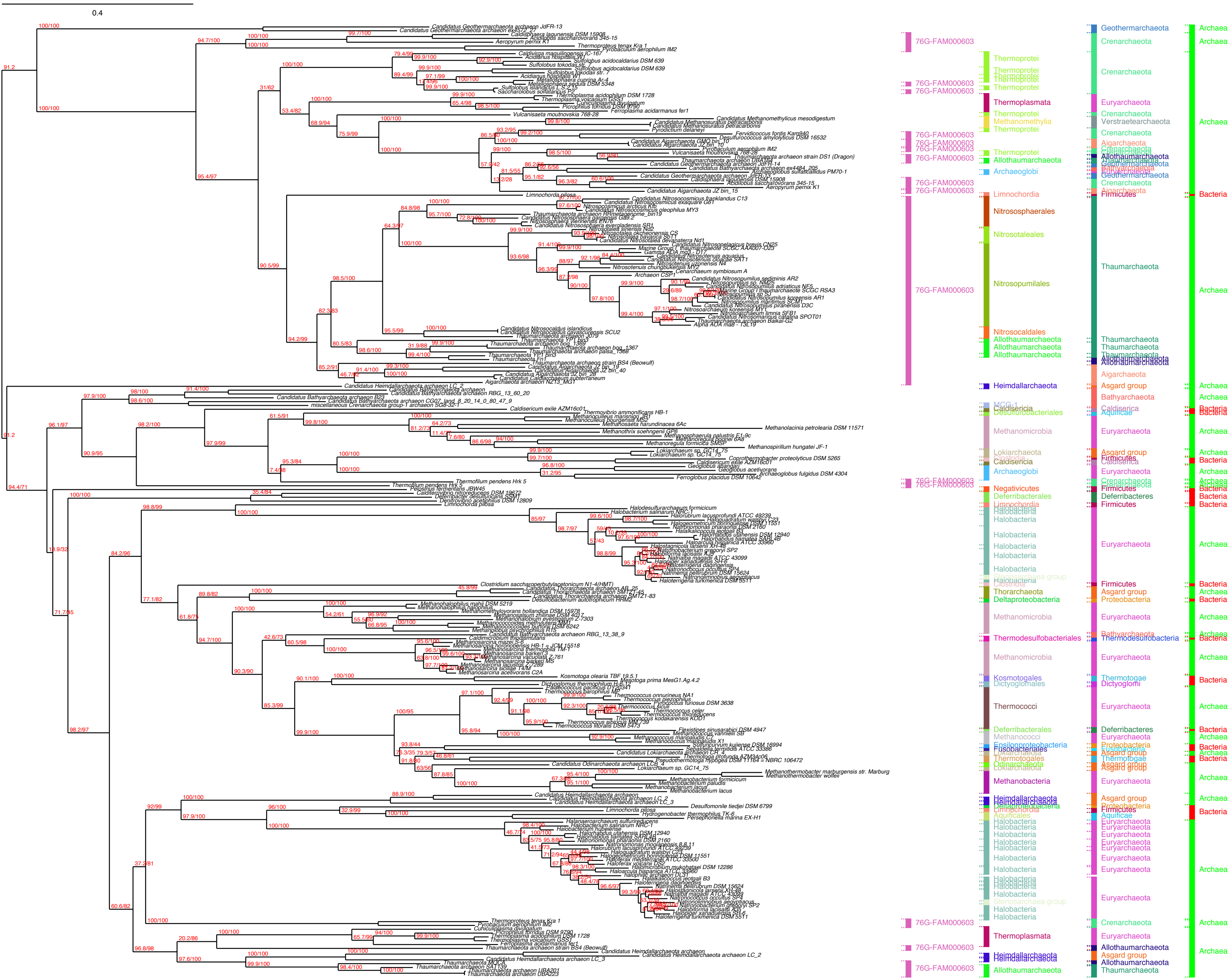
0.4
76G-FAM000603
Thermoprotei
Thermoplasmata
Methanomethylia
Allothaumarchaeota
Archaeoglobi
Limnochordia
Nitrososphaerales
Nitrosotaleales
Nitrosopumilales
Nitrosocaldales
Heimdallarchaeota
Caldiserica
Desulfurobacteriales
Methanomicrobia
Lokiarchaeota
Negativicutes
Deferribacterales
Halobacteria
Thorarchaeota
Deltaproteobacteria
Thermodesulfobacteriales
Kosmotogales
Dictyoglomales
Thermococci
Methanococci
Epsilonproteobacteria
Fusobacteriales
Thermotogales
Odinarchaeota
Methanobacteria
Aquificales
Geothermarchaeota
Crenarchaeota
Euryarchaeota
Verstraetearchaeota
Aigarchaeota
Firmicutes
Thaumarchaeota
Asgard group
Bathyarchaeota
Aquificae
Deferribacteres
Proteobacteria
Thermodesulfobacteria
Thermotogae
Dictyoglomi
Archaea
Bacteria

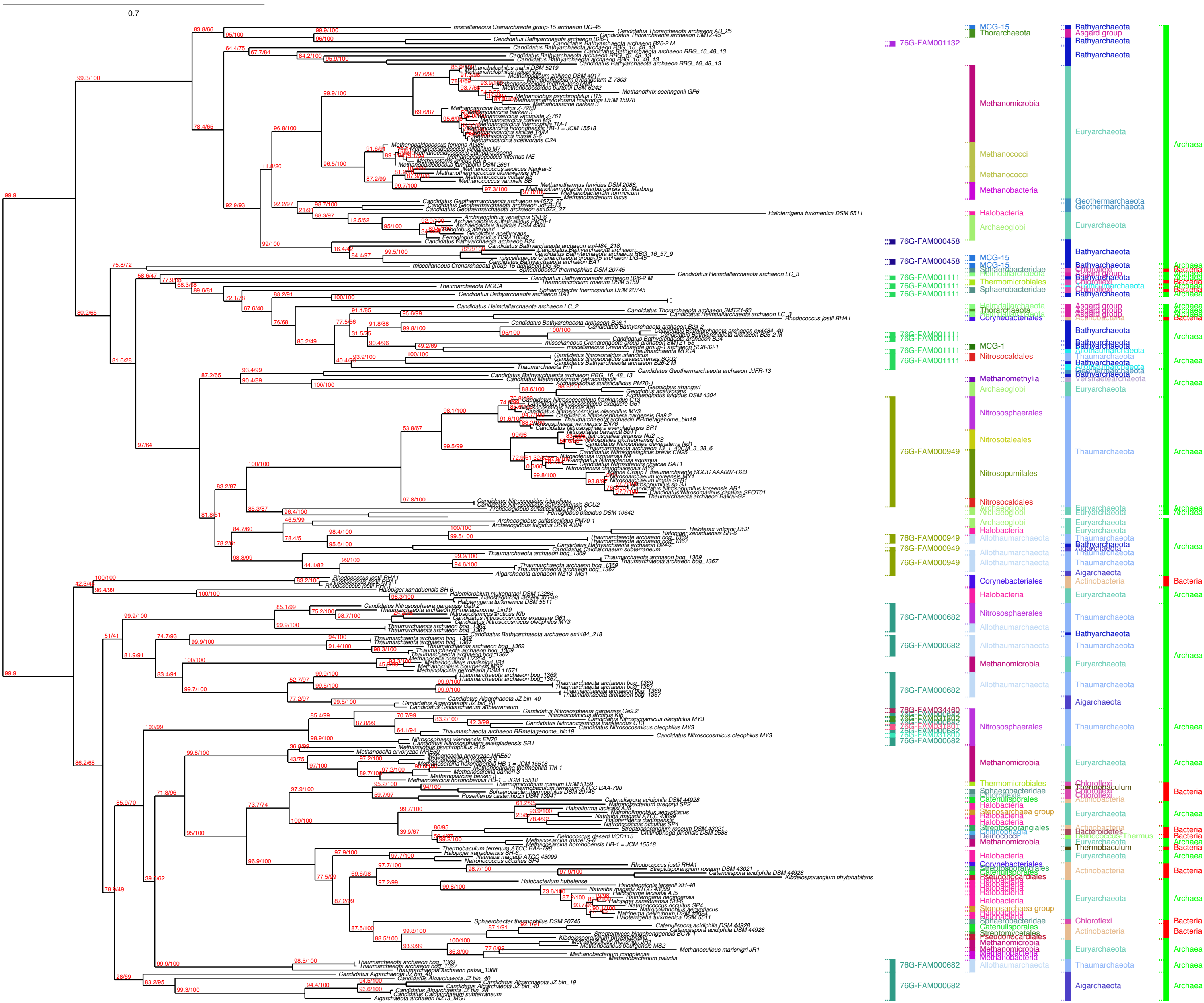

0.7
76G-FAM001132
76G-FAM000458
76G-FAM001111
76G-FAM000949
76G-FAM000682
76G-FAM034460
MCG-15
Thorarchaeota
Methanomicrobia
Methanococci
Methanobacteria
Halobacteria
Archaeoglobi
Sphaerobacteridae
Thermomicrobiales
Heimdallarchaeota
Corynebacteriales
MCG-1
Nitrosocaldales
Methanomethylia
Nitrososphaerales
Nitrosotaleales
Nitrosopumilales
Allothaumarchaeota
Catenulisporales
Stenosarchaea group
Streptosporangiales
Deinococci
Pseudonocardiales
Streptomycetales
Bathyarchaeota
Asgard group
Euryarchaeota
Geothermarchaeota
Chloroflexi
Thaumarchaeota
Verstraetearchaeota
Aigarchaeota
Actinobacteria
Thermobaculum
Bacteroidetes
Deinococcus-Thermus
Archaea
Bacteria

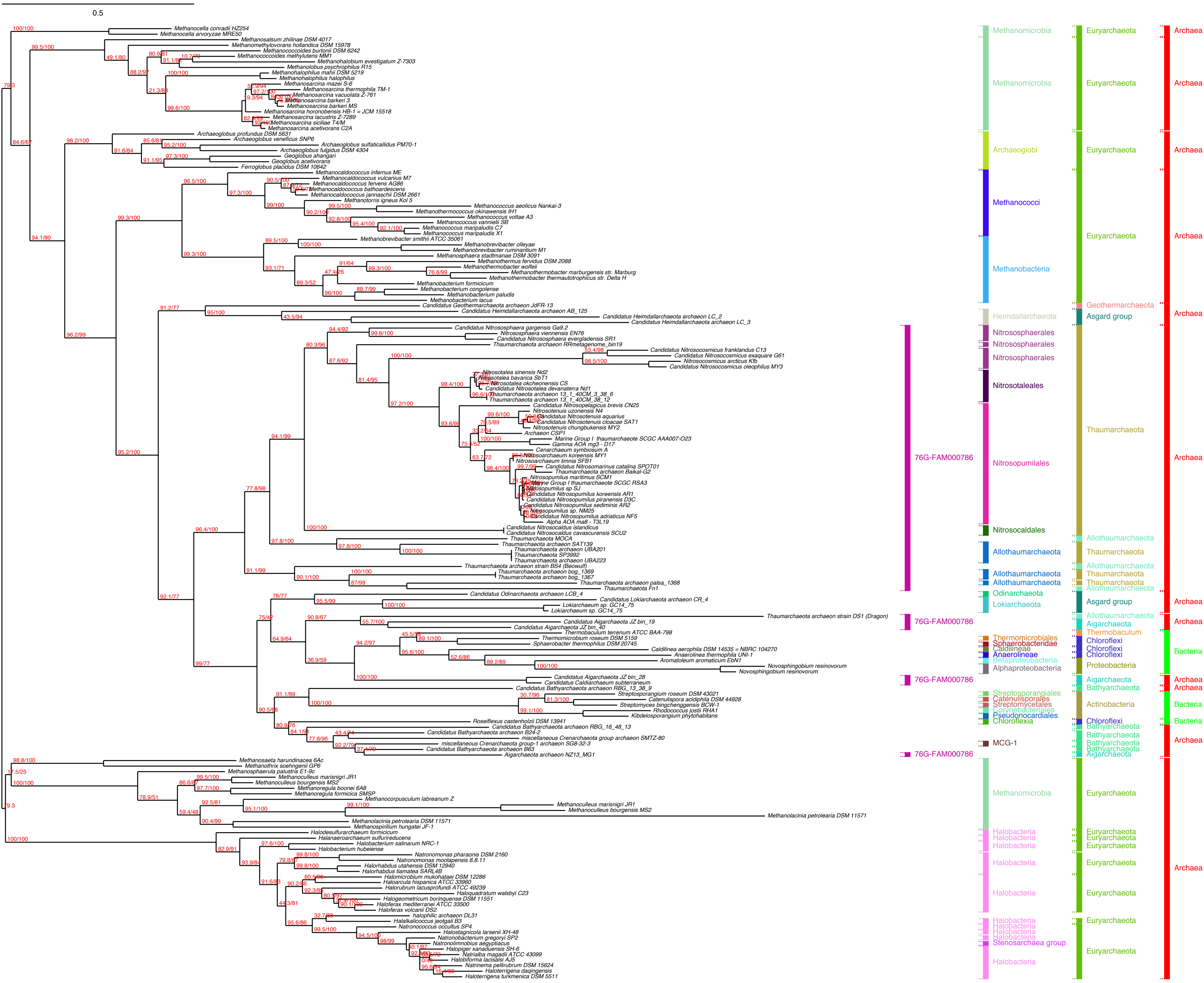
0.5
Methanocella conradii HZ254
Methanocella arvoryzae MRE50
Methanosalsum zhilinae DSM 4017
Methanomethylovorans hollandica DSM 15978
Methanococcoides burtonii DSM 6242
Methanococcoides methylutens MM1
Methanohalobium evestigatum Z-7303
Methanolobus psychrophilus R15
Methanohalophilus mahii DSM 5219
Methanohalophilus halophilus
Methanosarcina mazei S-6
Methanosarcina thermophila TM-1
Methanosarcina vacuolata Z-761
Methanosarcina barkeri 3
Methanosarcina barkeri MS
Methanosarcina horonobensis HB-1 = JCM 15518
Methanosarcina lacustris Z-7289
Methanosarcina siciliae T4/M
Methanosarcina acetivorans C2A
Archaeoglobus profundus DSM 5631
Archaeoglobus veneficus SNP6
Archaeoglobus sulfaticallidus PM70-1
Archaeoglobus fulgidus DSM 4304
Geoglobus ahangari
Geoglobus acetivorans
Ferroglobus placidus DSM 10642
Methanocaldococcus infernus ME
Methanocaldococcus vulcanius M7
Methanocaldococcus fervens AG86
Methanocaldococcus bathoardescens
Methanocaldococcus jannaschii DSM 2661
Methanotorris igneus Kol 5
Methanococcus aeolicus Nankai-3
Methanothermococcus okinawensis IH1
Methanococcus voltae A3
Methanococcus vannielii SB
Methanococcus maripaludis C7
Methanococcus maripaludis X1
Methanobrevibacter smithii ATCC 35061
Methanobrevibacter olleyae
Methanobrevibacter ruminantium M1
Methanosphaera stadtmanae DSM 3091
Methanothermus fervidus DSM 2088
Methanothermobacter wolfeii
Methanothermobacter marburgensis str. Marburg
Methanothermobacter thermautotrophicus str. Delta H
Methanobacterium formicicum
Methanobacterium congolense
Methanobacterium paludis
Methanobacterium lacus
Candidatus Geothermarchaeota archaeon JdFR-13
Candidatus Heimdallarchaeota archaeon AB_125
Candidatus Heimdallarchaeota archaeon LC_2
Candidatus Heimdallarchaeota archaeon LC_3
Candidatus Nitrososphaera gargensis Ga9.2
Nitrososphaera viennensis EN76
Candidatus Nitrososphaera evergladensis SR1
Thaumarchaeota archaeon RRmetagenome_bin19
Candidatus Nitrosocosmicus franklandus C13
Candidatus Nitrosocosmicus exaquare G61
Nitrosocosmicus arcticus Kfb
Candidatus Nitrosocosmicus oleophilus MY3
Nitrosotalea sinensis Nd2
Nitrosotalea bavarica SbT1
Nitrosotalea okcheonensis CS
Candidatus Nitrosotalea devanaterra Nd1
Thaumarchaeota archaeon 13_1_40CM_3_38_6
Thaumarchaeota archaeon 13_1_40CM_38_12
Candidatus Nitrosopelagicus brevis CN25
Nitrosotenuis uzonensis N4
Candidatus Nitrosotenuis aquarius
Candidatus Nitrosotenuis cloacae SAT1
Nitrosotenuis chungbukensis MY2
Archaeon CSP1
Marine Group I thaumarchaeote SCGC AAA007-O23
Gamma AOA mg3 - D17
Cenarchaeum symbiosum A
Nitrosoarchaeum koreensis MY1
Nitrosoarchaeum limnia SFB1
Candidatus Nitrosomarinus catalina SPOT01
Thaumarchaeota archaeon Baikal-G2
Nitrosopumilus maritimus SCM1
Marine Group I thaumarchaeote SCGC RSA3
Nitrosopumilus sp SJ
Candidatus Nitrosopumilus koreensis AR1
Candidatus Nitrosopumilus piranensis D3C
Candidatus Nitrosopumilus sediminis AR2
Nitrosopumilus sp. NM25
Candidatus Nitrosopumilus adriaticus NF5
Alpha AOA ma8 - T3L19
Candidatus Nitrosocaldus islandicus
Candidatus Nitrosocaldus cavascurensis SCU2
Thaumarchaeota MOCA
Thaumarchaeota archaeon SAT139
Thaumarchaeota archaeon UBA201
Thaumarchaeota SP3992
Thaumarchaeota archaeon UBA223
Thaumarchaeota archaeon strain BS4 (Beowulf)
Thaumarchaeota archaeon bog_1369
Thaumarchaeota archaeon bog_1367
Thaumarchaeota archaeon palsa_1368
Thaumarchaeota Fn1
Candidatus Odinarchaeota archaeon LCB_4
Candidatus Lokiarchaeota archaeon CR_4
Lokiarchaeum sp. GC14_75
Lokiarchaeum sp. GC14_75
Thaumarchaeota archaeon strain DS1 (Dragon)
Candidatus Aigarchaeota JZ bin_19
Candidatus Aigarchaeota JZ bin_40
Thermobaculum terrenum ATCC BAA-798
Thermomicrobium roseum DSM 5159
Sphaerobacter thermophilus DSM 20745
Caldilinea aerophila DSM 14535 = NBRC 104270
Anaerolinea thermophila UNI-1
Aromatoleum aromaticum EbN1
Novosphingobium resinovorum
Novosphingobium resinovorum
Candidatus Aigarchaeota JZ bin_28
Candidatus Caldiarchaeum subterraneum
Candidatus Bathyarchaeota archaeon RBG_13_38_9
Streptosporangium roseum DSM 43021
Catenulispora acidiphila DSM 44928
Streptomyces bingchenggensis BCW-1
Rhodococcus jostii RHA1
Kibdelosporangium phytohabitans
Roseiflexus castenholzii DSM 13941
Candidatus Bathyarchaeota archaeon RBG_16_48_13
Candidatus Bathyarchaeota archaeon B24-2
miscellaneous Crenarchaeota group archaeon SMTZ-80
miscellaneous Crenarchaeota group-1 archaeon SG8-32-3
Candidatus Bathyarchaeota archaeon B63
Aigarchaeota archaeon NZ13_MG1
Methanosaeta harundinacea 6Ac
Methanothrix soehngenii GP6
Methanosphaerula palustris E1-9c
Methanoculleus marisnigri JR1
Methanoculleus bourgensis MS2
Methanoregula boonei 6A8
Methanoregula formicica SMSP
Methanocorpusculum labreanum Z
Methanoculleus marisnigri JR1
Methanoculleus bourgensis MS2
Methanolacinia petrolearia DSM 11571
Methanolacinia petrolearia DSM 11571
Methanospirillum hungatei JF-1
Halodesulfurarchaeum formicicum
Halanaeroarchaeum sulfurireducens
Halobacterium salinarum NRC-1
Halobacterium hubeiense
Natronomonas pharaonis DSM 2160
Natronomonas moolapensis 8.8.11
Halorhabdus utahensis DSM 12940
Halorhabdus tiamatea SARL4B
Halomicrobium mukohataei DSM 12286
Haloarcula hispanica ATCC 33960
Halorubrum lacusprofundi ATCC 49239
Haloquadratum walsbyi C23
Halogeometricum borinquense DSM 11551
Haloferax mediterranei ATCC 33500
Haloferax volcanii DS2
halophilic archaeon DL31
Halalkalicoccus jeotgali B3
Natronococcus occultus SP4
Halostagnicola larsenii XH-48
Natronobacterium gregoryi SP2
Natronolimnobius aegyptiacus
Halopiger xanaduensis SH-6
Natrialba magadii ATCC 43099
Halobiforma lacisalsi AJ5
Natrinema pellirubrum DSM 15624
Haloterrigena daqingensis
Haloterrigena turkmenica DSM 5511
76G-FAM000786
76G-FAM000786
76G-FAM000786
76G-FAM000786
Methanomicrobia
Methanomicrobia
Archaeoglobi
Methanococci
Methanobacteria
Heimdallarchaeota
Nitrososphaerales
Nitrososphaerales
Nitrososphaerales
Nitrosotaleales
Nitrosopumilales
Nitrosocaldales
Allothaumarchaeota
Allothaumarchaeota
Allothaumarchaeota
Odinarchaeota
Lokiarchaeota
Thermomicrobiales
Sphaerobacteridae
Caldilineae
Anaerolineae
Betaproteobacteria
Alphaproteobacteria
Streptosporangiales
Catenulisporales
Streptomycetales
Corynebacteriales
Pseudonocardiales
Chloroflexia
MCG-1
Methanomicrobia
Halobacteria
Halobacteria
Halobacteria
Halobacteria
Halobacteria
Halobacteria
Halobacteria
Halobacteria
Halobacteria
Stenosarchaea group
Halobacteria
Euryarchaeota
Euryarchaeota
Euryarchaeota
Euryarchaeota
Euryarchaeota
Geothermarchaeota
Asgard group
Thaumarchaeota
Allothaumarchaeota
Thaumarchaeota
Allothaumarchaeota
Thaumarchaeota
Thaumarchaeota
Allothaumarchaeota
Asgard group
Allothaumarchaeota
Aigarchaeota
Thermobaculum
Chloroflexi
Chloroflexi
Chloroflexi
Proteobacteria
Aigarchaeota
Bathyarchaeota
Actinobacteria
Chloroflexi
Bathyarchaeota
Bathyarchaeota
Bathyarchaeota
Bathyarchaeota
Aigarchaeota
Euryarchaeota
Euryarchaeota
Euryarchaeota
Euryarchaeota
Euryarchaeota
Euryarchaeota
Euryarchaeota
Euryarchaeota
Archaea
Archaea
Archaea
Archaea
Archaea
Archaea
Archaea
Archaea
Bacteria
Archaea
Archaea
Bacteria
Bacteria
Archaea
Archaea

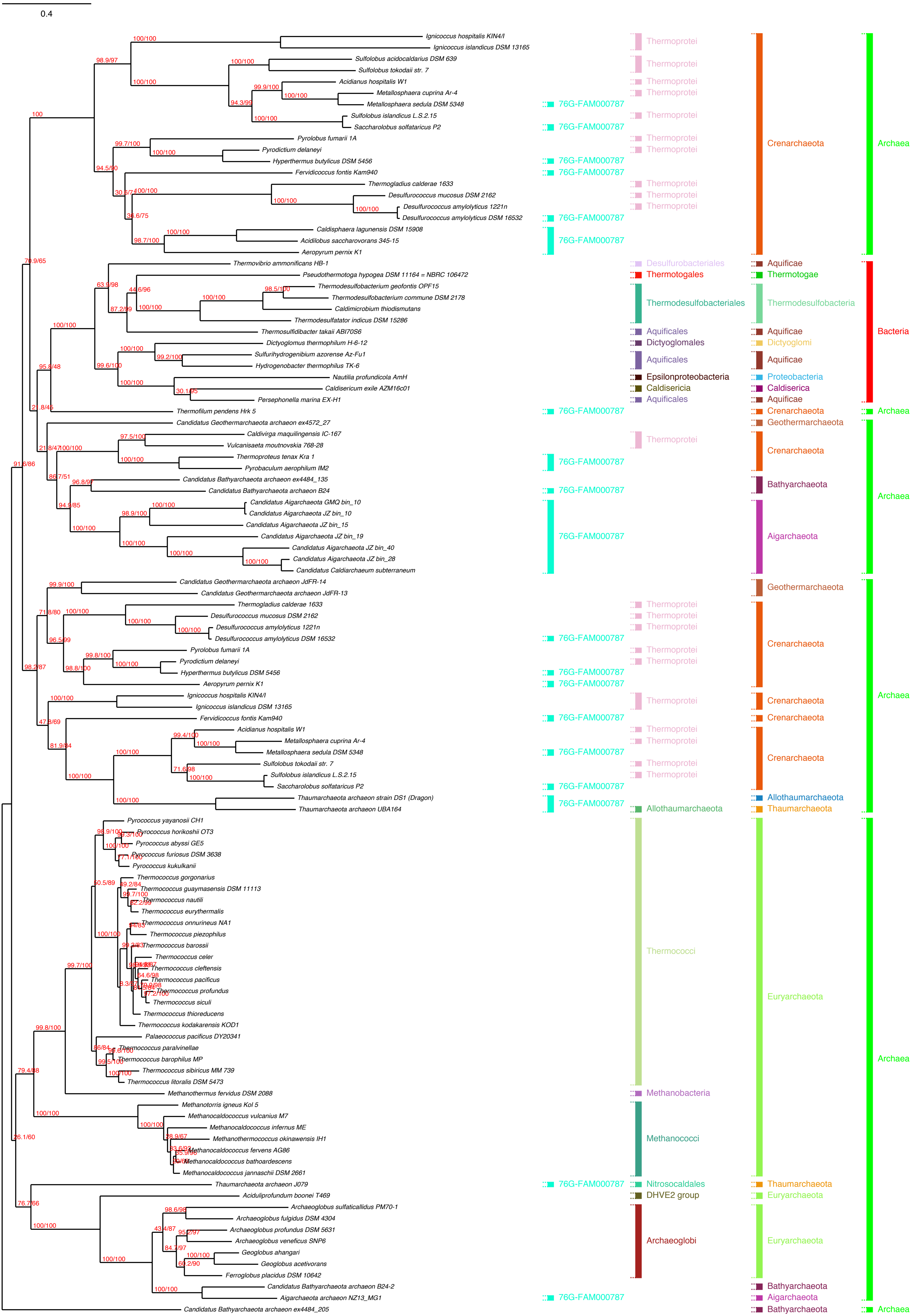

0.4
Ignicoccus hospitalis KIN4/I
Ignicoccus islandicus DSM 13165
Sulfolobus acidocaldarius DSM 639
Sulfolobus tokodaii str. 7
Acidianus hospitalis W1
Metallosphaera cuprina Ar-4
Metallosphaera sedula DSM 5348
Sulfolobus islandicus L.S.2.15
Saccharolobus solfataricus P2
Pyrolobus fumarii 1A
Pyrodictium delaneyi
Hyperthermus butylicus DSM 5456
Fervidicoccus fontis Kam940
Thermogladius calderae 1633
Desulfurococcus mucosus DSM 2162
Desulfurococcus amylolyticus 1221n
Desulfurococcus amylolyticus DSM 16532
Caldisphaera lagunensis DSM 15908
Acidilobus saccharovorans 345-15
Aeropyrum pernix K1
Thermovibrio ammonificans HB-1
Pseudothermotoga hypogea DSM 11164 = NBRC 106472
Thermodesulfobacterium geofontis OPF15
Thermodesulfobacterium commune DSM 2178
Caldimicrobium thiodismutans
Thermodesulfatator indicus DSM 15286
Thermosulfidibacter takaii ABI70S6
Dictyoglomus thermophilum H-6-12
Sulfurihydrogenibium azorense Az-Fu1
Hydrogenobacter thermophilus TK-6
Nautilia profundicola AmH
Caldisericum exile AZM16c01
Persephonella marina EX-H1
Thermofilum pendens Hrk 5
Candidatus Geothermarchaeota archaeon ex4572_27
Caldivirga maquilingensis IC-167
Vulcanisaeta moutnovskia 768-28
Thermoproteus tenax Kra 1
Pyrobaculum aerophilum IM2
Candidatus Bathyarchaeota archaeon ex4484_135
Candidatus Bathyarchaeota archaeon B24
Candidatus Aigarchaeota GMQ bin_10
Candidatus Aigarchaeota JZ bin_10
Candidatus Aigarchaeota JZ bin_15
Candidatus Aigarchaeota JZ bin_19
Candidatus Aigarchaeota JZ bin_40
Candidatus Aigarchaeota JZ bin_28
Candidatus Caldiarchaeum subterraneum
Candidatus Geothermarchaeota archaeon JdFR-14
Candidatus Geothermarchaeota archaeon JdFR-13
Thermogladius calderae 1633
Desulfurococcus mucosus DSM 2162
Desulfurococcus amylolyticus 1221n
Desulfurococcus amylolyticus DSM 16532
Pyrolobus fumarii 1A
Pyrodictium delaneyi
Hyperthermus butylicus DSM 5456
Aeropyrum pernix K1
Ignicoccus hospitalis KIN4/I
Ignicoccus islandicus DSM 13165
Fervidicoccus fontis Kam940
Acidianus hospitalis W1
Metallosphaera cuprina Ar-4
Metallosphaera sedula DSM 5348
Sulfolobus tokodaii str. 7
Sulfolobus islandicus L.S.2.15
Saccharolobus solfataricus P2
Thaumarchaeota archaeon strain DS1 (Dragon)
Thaumarchaeota archaeon UBA164
Pyrococcus yayanosii CH1
Pyrococcus horikoshii OT3
Pyrococcus abyssi GE5
Pyrococcus furiosus DSM 3638
Pyrococcus kukulkanii
Thermococcus gorgonarius
Thermococcus guaymasensis DSM 11113
Thermococcus nautili
Thermococcus eurythermalis
Thermococcus onnurineus NA1
Thermococcus piezophilus
Thermococcus barossii
Thermococcus celer
Thermococcus cleftensis
Thermococcus pacificus
Thermococcus profundus
Thermococcus siculi
Thermococcus thioreducens
Thermococcus kodakarensis KOD1
Palaeococcus pacificus DY20341
Thermococcus paralvinellae
Thermococcus barophilus MP
Thermococcus sibiricus MM 739
Thermococcus litoralis DSM 5473
Methanothermus fervidus DSM 2088
Methanotorris igneus Kol 5
Methanocaldococcus vulcanius M7
Methanocaldococcus infernus ME
Methanothermococcus okinawensis IH1
Methanocaldococcus fervens AG86
Methanocaldococcus bathoardescens
Methanocaldococcus jannaschii DSM 2661
Thaumarchaeota archaeon J079
Aciduliprofundum boonei T469
Archaeoglobus sulfaticallidus PM70-1
Archaeoglobus fulgidus DSM 4304
Archaeoglobus profundus DSM 5631
Archaeoglobus veneficus SNP6
Geoglobus ahangari
Geoglobus acetivorans
Ferroglobus placidus DSM 10642
Candidatus Bathyarchaeota archaeon B24-2
Aigarchaeota archaeon NZ13_MG1
Candidatus Bathyarchaeota archaeon ex4484_205
76G-FAM000787
Thermoprotei
Desulfurobacteriales
Thermotogales
Thermodesulfobacteriales
Aquificales
Dictyoglomales
Epsilonproteobacteria
Caldisericia
Allothaumarchaeota
Thermococci
Methanobacteria
Methanococci
Nitrosocaldales
DHVE2 group
Archaeoglobi
Crenarchaeota
Aquificae
Thermotogae
Thermodesulfobacteria
Dictyoglomi
Proteobacteria
Caldiserica
Geothermarchaeota
Bathyarchaeota
Aigarchaeota
Thaumarchaeota
Euryarchaeota
Archaea
Bacteria

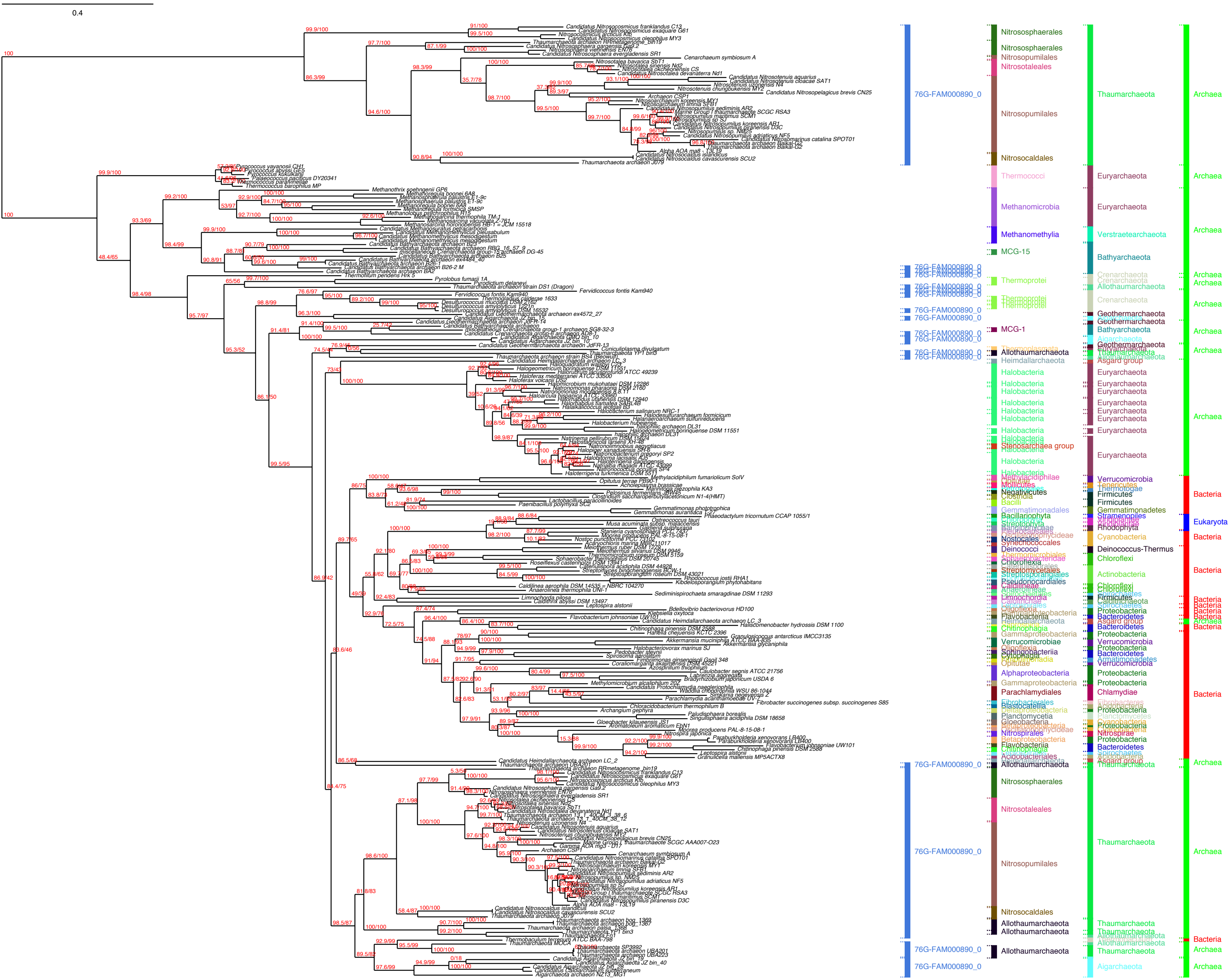

0.4
76G-FAM000890_0
Nitrososphaerales
Nitrosopumilales
Nitrosotaleales
Nitrosocaldales
Thermococci
Methanomicrobia
Methanomethylia
MCG-15
Thermoprotei
MCG-1
Allothaumarchaeota
Heimdallarchaeota
Halobacteria
Stenosarchaea group
Thaumarchaeota
Euryarchaeota
Verstraetearchaeota
Bathyarchaeota
Crenarchaeota
Geothermarchaeota
Aigarchaeota
Asgard group
Verrucomicrobia
Firmicutes
Gemmatimonadetes
Stramenopiles
Rhodophyta
Cyanobacteria
Deinococcus-Thermus
Chloroflexi
Actinobacteria
Proteobacteria
Bacteroidetes
Chlamydiae
Nitrospirae
Archaea
Bacteria
Eukaryota

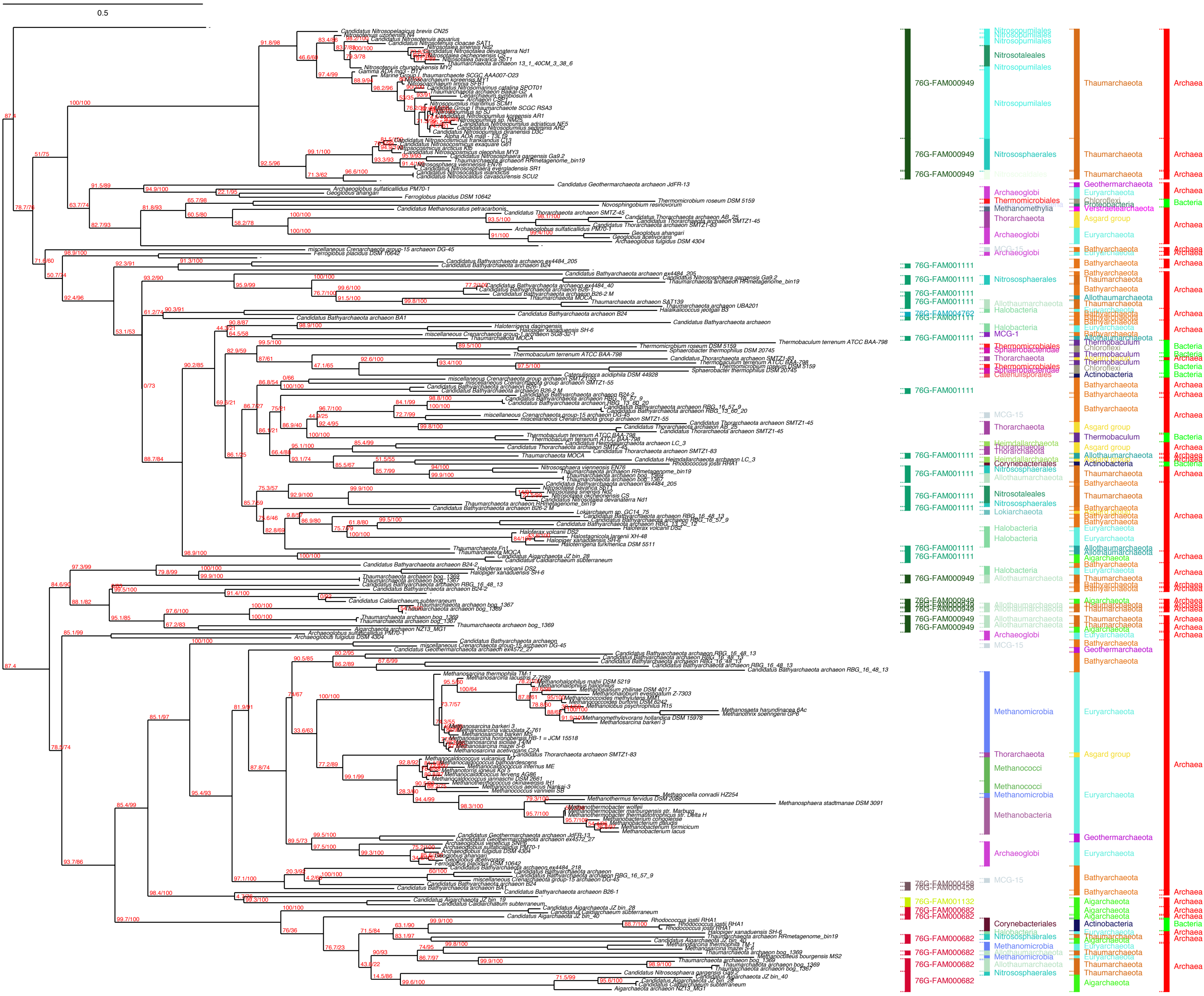

0.5
76G-FAM000949
76G-FAM001111
76G-FAM004762
76G-FAM000458
76G-FAM001132
76G-FAM000682
Nitrosopumilales
Nitrosotaleales
Nitrososphaerales
Archaeoglobi
Thermomicrobiales
Methanomethylia
Thorarchaeota
MCG-15
Allothaumarchaeota
Halobacteria
MCG-1
Sphaerobacteridae
Catenulisporales
Heimdallarchaeota
Corynebacteriales
Lokiarchaeota
Methanomicrobia
Methanococci
Methanobacteria
Thaumarchaeota
Geothermarchaeota
Euryarchaeota
Chloroflexi
Proteobacteria
Verstraetearchaeota
Asgard group
Bathyarchaeota
Thermobaculum
Actinobacteria
Aigarchaeota
Archaea
Bacteria

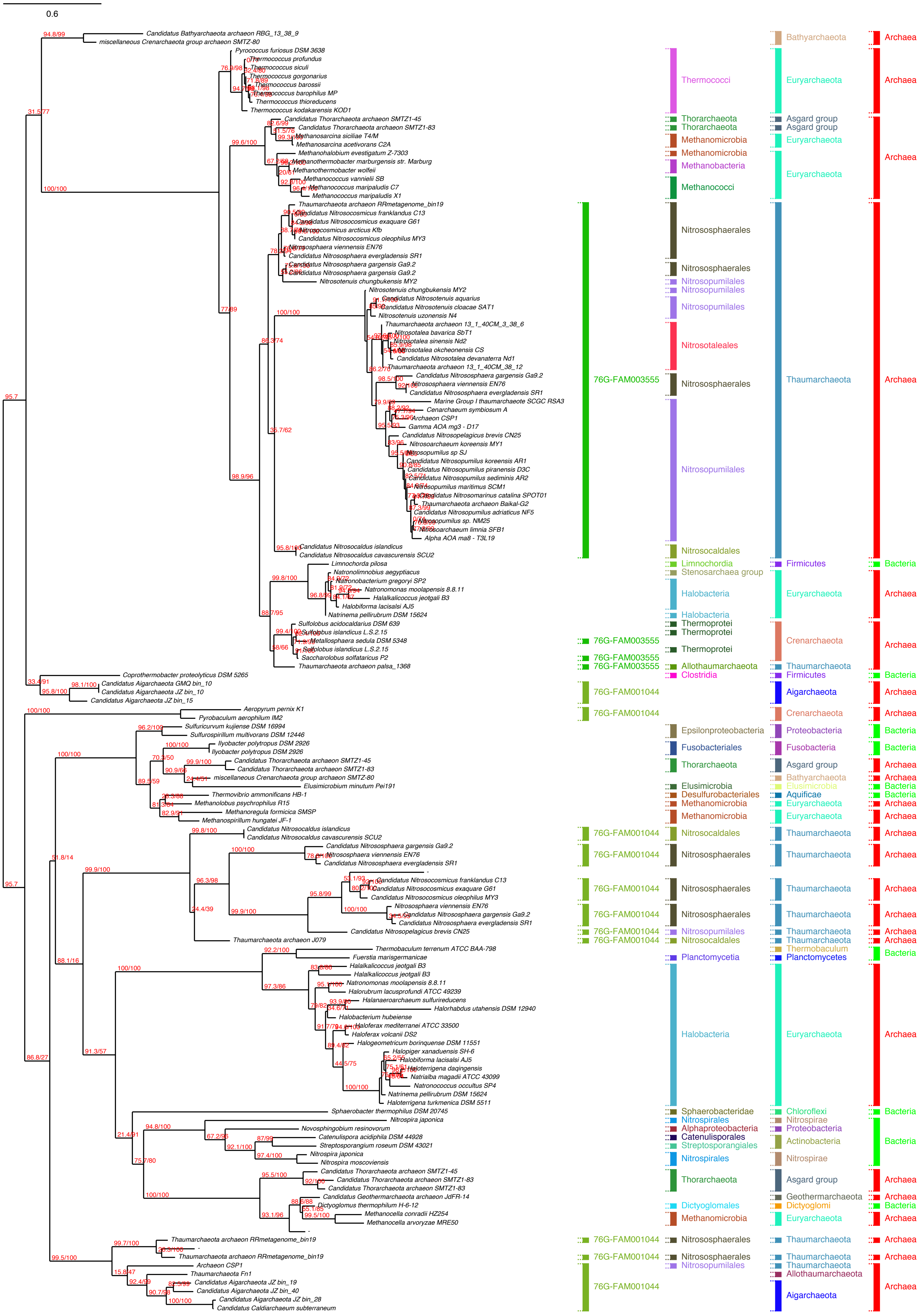
0.6
Candidatus Bathyarchaeota archaeon RBG_13_38_9
miscellaneous Crenarchaeota group archaeon SMTZ-80
Pyrococcus furiosus DSM 3638
Thermococcus profundus
Thermococcus siculi
Thermococcus gorgonarius
Thermococcus barossii
Thermococcus barophilus MP
Thermococcus thioreducens
Thermococcus kodakarensis KOD1
Candidatus Thorarchaeota archaeon SMTZ1-45
Candidatus Thorarchaeota archaeon SMTZ1-83
Methanosarcina siciliae T4/M
Methanosarcina acetivorans C2A
Methanohalobium evestigatum Z-7303
Methanothermobacter marburgensis str. Marburg
Methanothermobacter wolfeii
Methanococcus vannielii SB
Methanococcus maripaludis C7
Methanococcus maripaludis X1
Thaumarchaeota archaeon RRmetagenome_bin19
Candidatus Nitrosocosmicus franklandus C13
Candidatus Nitrosocosmicus exaquare G61
Nitrosocosmicus arcticus Kfb
Candidatus Nitrosocosmicus oleophilus MY3
Nitrososphaera viennensis EN76
Candidatus Nitrososphaera evergladensis SR1
Candidatus Nitrososphaera gargensis Ga9.2
Candidatus Nitrososphaera gargensis Ga9.2
Nitrosotenuis chungbukensis MY2
Nitrosotenuis chungbukensis MY2
Candidatus Nitrosotenuis aquarius
Candidatus Nitrosotenuis cloacae SAT1
Nitrosotenuis uzonensis N4
Thaumarchaeota archaeon 13_1_40CM_3_38_6
Nitrosotalea bavarica SbT1
Nitrosotalea sinensis Nd2
Nitrosotalea okcheonensis CS
Candidatus Nitrosotalea devanaterra Nd1
Thaumarchaeota archaeon 13_1_40CM_38_12
Candidatus Nitrososphaera gargensis Ga9.2
Nitrososphaera viennensis EN76
Candidatus Nitrososphaera evergladensis SR1
Marine Group I thaumarchaeote SCGC RSA3
Cenarchaeum symbiosum A
Archaeon CSP1
Gamma AOA mg3 - D17
Candidatus Nitrosopelagicus brevis CN25
Nitrosoarchaeum koreensis MY1
Nitrosopumilus sp SJ
Candidatus Nitrosopumilus koreensis AR1
Candidatus Nitrosopumilus piranensis D3C
Candidatus Nitrosopumilus sediminis AR2
Nitrosopumilus maritimus SCM1
Candidatus Nitrosomarinus catalina SPOT01
Thaumarchaeota archaeon Baikal-G2
Candidatus Nitrosopumilus adriaticus NF5
Nitrosopumilus sp. NM25
Nitrosoarchaeum limnia SFB1
Alpha AOA ma8 - T3L19
Candidatus Nitrosocaldus islandicus
Candidatus Nitrosocaldus cavascurensis SCU2
Limnochorda pilosa
Natronolimnobius aegyptiacus
Natronobacterium gregoryi SP2
Natronomonas moolapensis 8.8.11
Halalkalicoccus jeotgali B3
Halobiforma lacisalsi AJ5
Natrinema pellirubrum DSM 15624
Sulfolobus acidocaldarius DSM 639
Sulfolobus islandicus L.S.2.15
Metallosphaera sedula DSM 5348
Sulfolobus islandicus L.S.2.15
Saccharolobus solfataricus P2
Thaumarchaeota archaeon palsa_1368
Coprothermobacter proteolyticus DSM 5265
Candidatus Aigarchaeota GMQ bin_10
Candidatus Aigarchaeota JZ bin_10
Candidatus Aigarchaeota JZ bin_15
Aeropyrum pernix K1
Pyrobaculum aerophilum IM2
Sulfuricurvum kujiense DSM 16994
Sulfurospirillum multivorans DSM 12446
Ilyobacter polytropus DSM 2926
Ilyobacter polytropus DSM 2926
Candidatus Thorarchaeota archaeon SMTZ1-45
Candidatus Thorarchaeota archaeon SMTZ1-83
miscellaneous Crenarchaeota group archaeon SMTZ-80
Elusimicrobium minutum Pei191
Thermovibrio ammonificans HB-1
Methanolobus psychrophilus R15
Methanoregula formicica SMSP
Methanospirillum hungatei JF-1
Candidatus Nitrosocaldus islandicus
Candidatus Nitrosocaldus cavascurensis SCU2
Candidatus Nitrososphaera gargensis Ga9.2
Nitrososphaera viennensis EN76
Candidatus Nitrososphaera evergladensis SR1
Candidatus Nitrosocosmicus franklandus C13
Candidatus Nitrosocosmicus exaquare G61
Candidatus Nitrosocosmicus oleophilus MY3
Nitrososphaera viennensis EN76
Candidatus Nitrososphaera gargensis Ga9.2
Candidatus Nitrososphaera evergladensis SR1
Candidatus Nitrosopelagicus brevis CN25
Thaumarchaeota archaeon J079
Thermobaculum terrenum ATCC BAA-798
Fuerstia marisgermanicae
Halalkalicoccus jeotgali B3
Halalkalicoccus jeotgali B3
Natronomonas moolapensis 8.8.11
Halorubrum lacusprofundi ATCC 49239
Halanaeroarchaeum sulfurireducens
Halorhabdus utahensis DSM 12940
Halobacterium hubeiense
Haloferax mediterranei ATCC 33500
Haloferax volcanii DS2
Halogeometricum borinquense DSM 11551
Halopiger xanaduensis SH-6
Halobiforma lacisalsi AJ5
Haloterrigena daqingensis
Natrialba magadii ATCC 43099
Natronococcus occultus SP4
Natrinema pellirubrum DSM 15624
Haloterrigena turkmenica DSM 5511
Sphaerobacter thermophilus DSM 20745
Nitrospira japonica
Novosphingobium resinovorum
Catenulispora acidiphila DSM 44928
Streptosporangium roseum DSM 43021
Nitrospira japonica
Nitrospira moscoviensis
Candidatus Thorarchaeota archaeon SMTZ1-45
Candidatus Thorarchaeota archaeon SMTZ1-83
Candidatus Thorarchaeota archaeon SMTZ1-83
Candidatus Geothermarchaeota archaeon JdFR-14
Dictyoglomus thermophilum H-6-12
Methanocella conradii HZ254
Methanocella arvoryzae MRE50
Thaumarchaeota archaeon RRmetagenome_bin19
Thaumarchaeota archaeon RRmetagenome_bin19
Archaeon CSP1
Thaumarchaeota Fn1
Candidatus Aigarchaeota JZ bin_19
Candidatus Aigarchaeota JZ bin_40
Candidatus Aigarchaeota JZ bin_28
Candidatus Caldiarchaeum subterraneum
76G-FAM003555
76G-FAM001044
Thermococci
Thorarchaeota
Methanomicrobia
Methanobacteria
Methanococci
Nitrososphaerales
Nitrosopumilales
Nitrosotaleales
Nitrosocaldales
Limnochordia
Stenosarchaea group
Halobacteria
Thermoprotei
Allothaumarchaeota
Clostridia
Epsilonproteobacteria
Fusobacteriales
Elusimicrobia
Desulfurobacteriales
Planctomycetia
Sphaerobacteridae
Nitrospirales
Alphaproteobacteria
Catenulisporales
Streptosporangiales
Dictyoglomales
Bathyarchaeota
Euryarchaeota
Asgard group
Thaumarchaeota
Firmicutes
Crenarchaeota
Aigarchaeota
Proteobacteria
Fusobacteria
Aquificae
Thermobaculum
Planctomycetes
Chloroflexi
Nitrospirae
Actinobacteria
Geothermarchaeota
Dictyoglomi
Allothaumarchaeota
Archaea
Bacteria

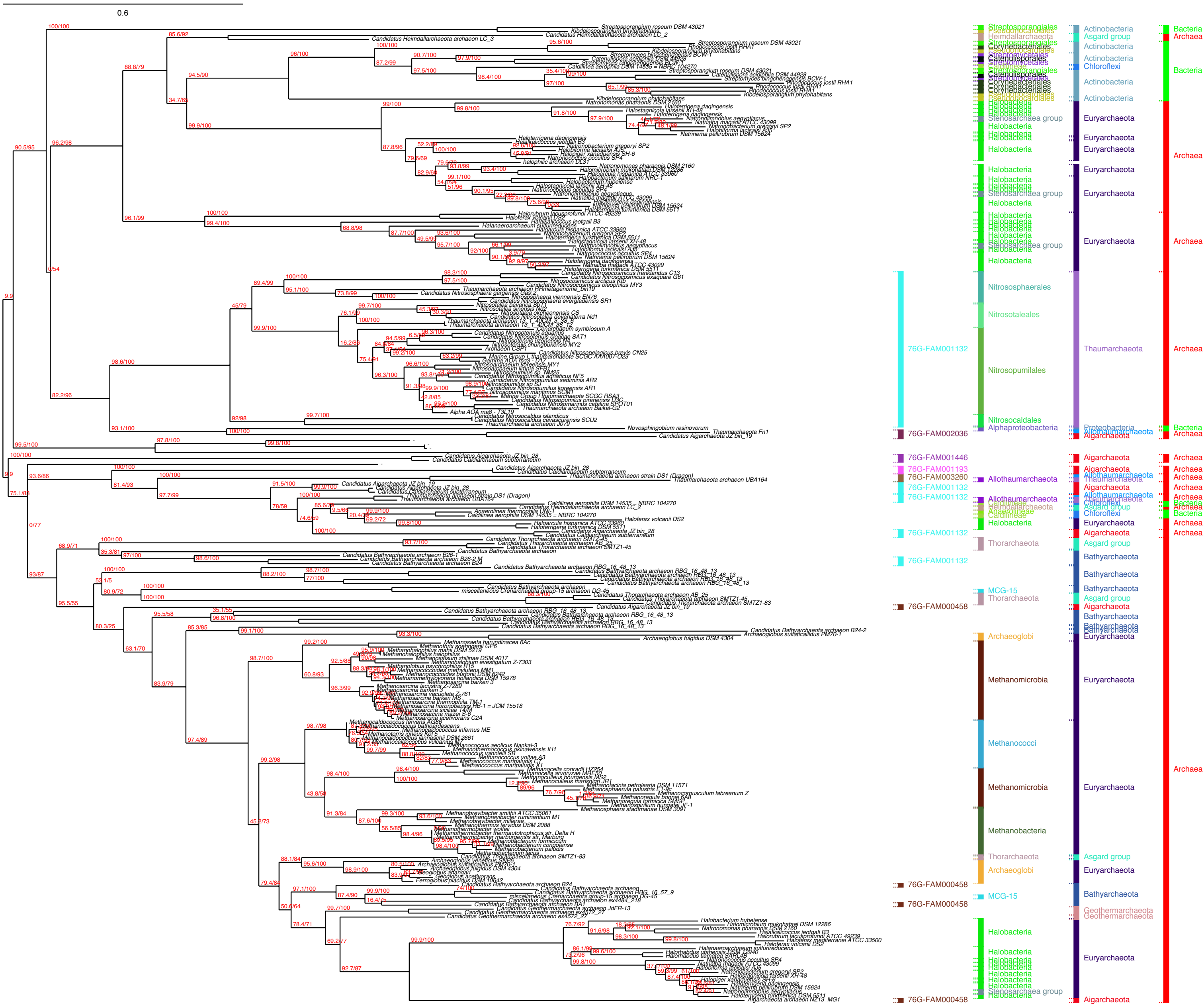
0.6
76G-FAM001132
76G-FAM002036
76G-FAM001446
76G-FAM001193
76G-FAM003260
76G-FAM000458
MCG-15
Actinobacteria
Asgard group
Chloroflexi
Euryarchaeota
Thaumarchaeota
Proteobacteria
Allothaumarchaeota
Aigarchaeota
Bathyarchaeota
Geothermarchaeota
Streptosporangiales
Heimdallarchaeota
Corynebacteriales
Catenulisporales
Streptomycetales
Halobacteria
Stenosarchaea group
Nitrososphaerales
Nitrosotaleales
Nitrosopumilales
Nitrosocaldales
Alphaproteobacteria
Thorarchaeota
Archaeoglobi
Methanomicrobia
Methanococci
Methanobacteria
Bacteria
Archaea

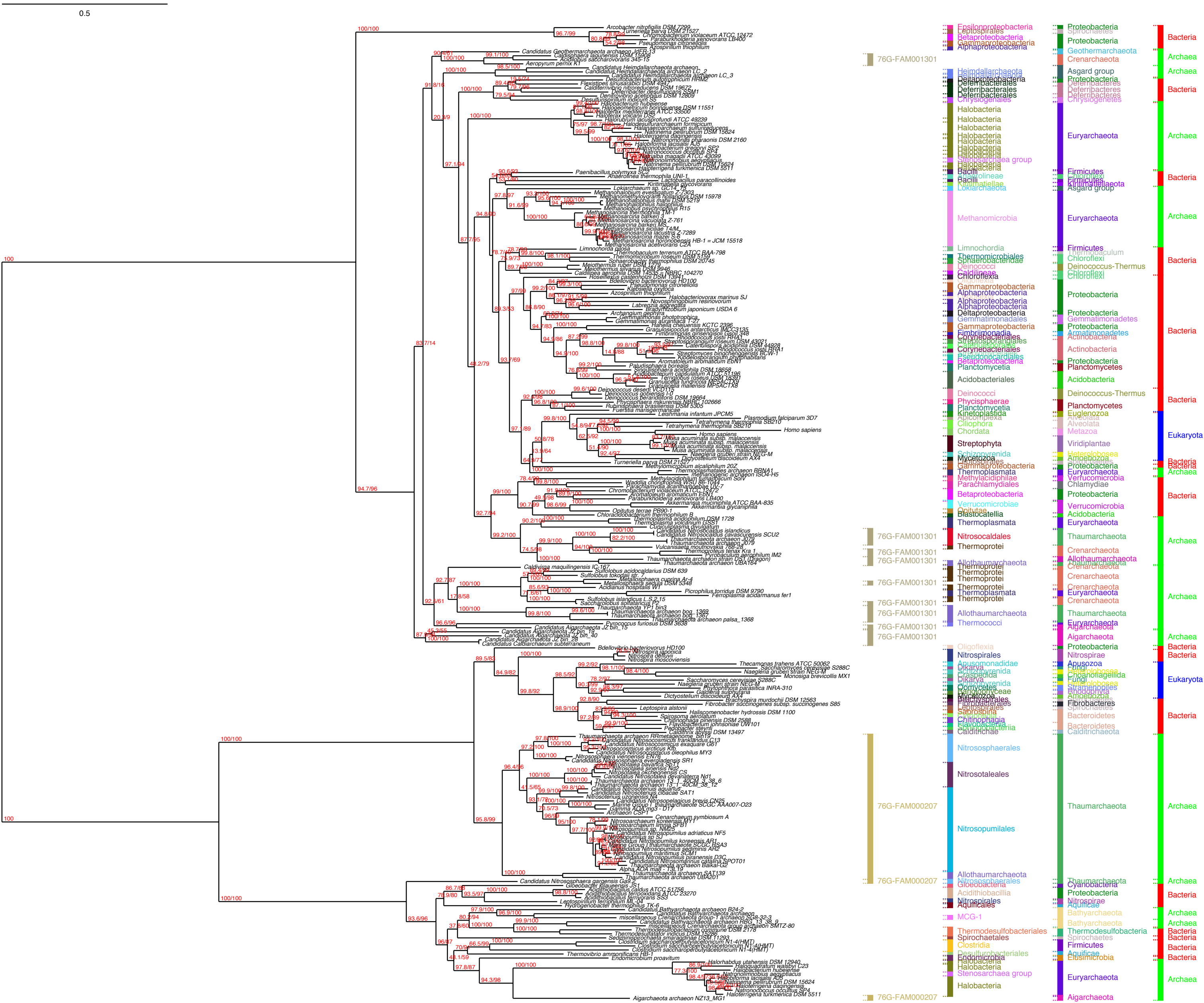
0.5
76G-FAM001301
76G-FAM000207
Bacteria
Archaea
Eukaryota

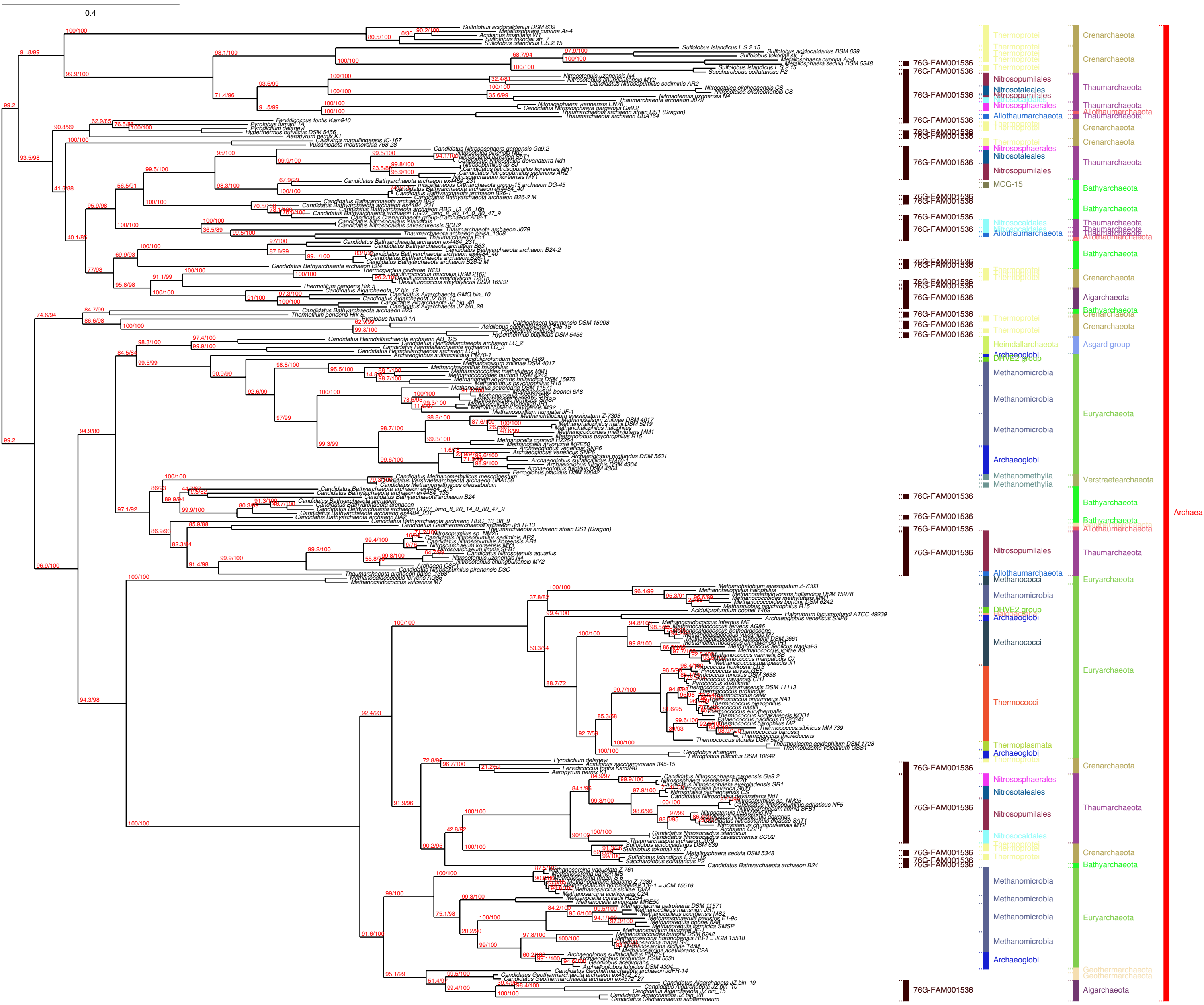

0.4
76G-FAM001536
Thermoprotei
Nitrosopumilales
Nitrosotaleales
Nitrososphaerales
Allothaumarchaeota
MCG-15
Nitrosocaldales
Heimdallarchaeota
Archaeoglobi
DHVE2 group
Methanomicrobia
Methanomethylia
Methanococci
Thermococci
Thermoplasmata
Crenarchaeota
Thaumarchaeota
Bathyarchaeota
Aigarchaeota
Asgard group
Euryarchaeota
Verstraetearchaeota
Geothermarchaeota
Archaea

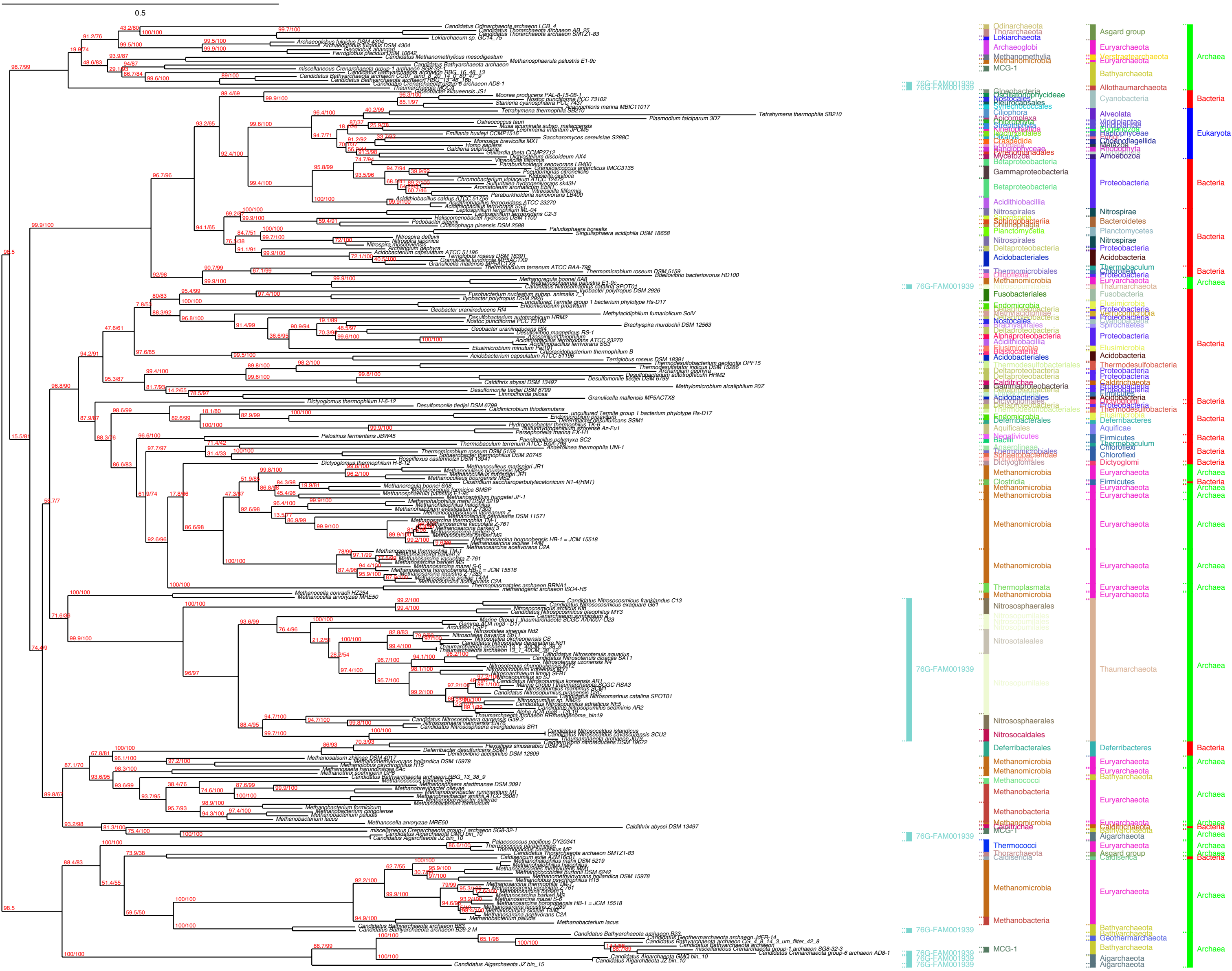
0.5
76G-FAM001939
Archaea
Bacteria
Eukaryota

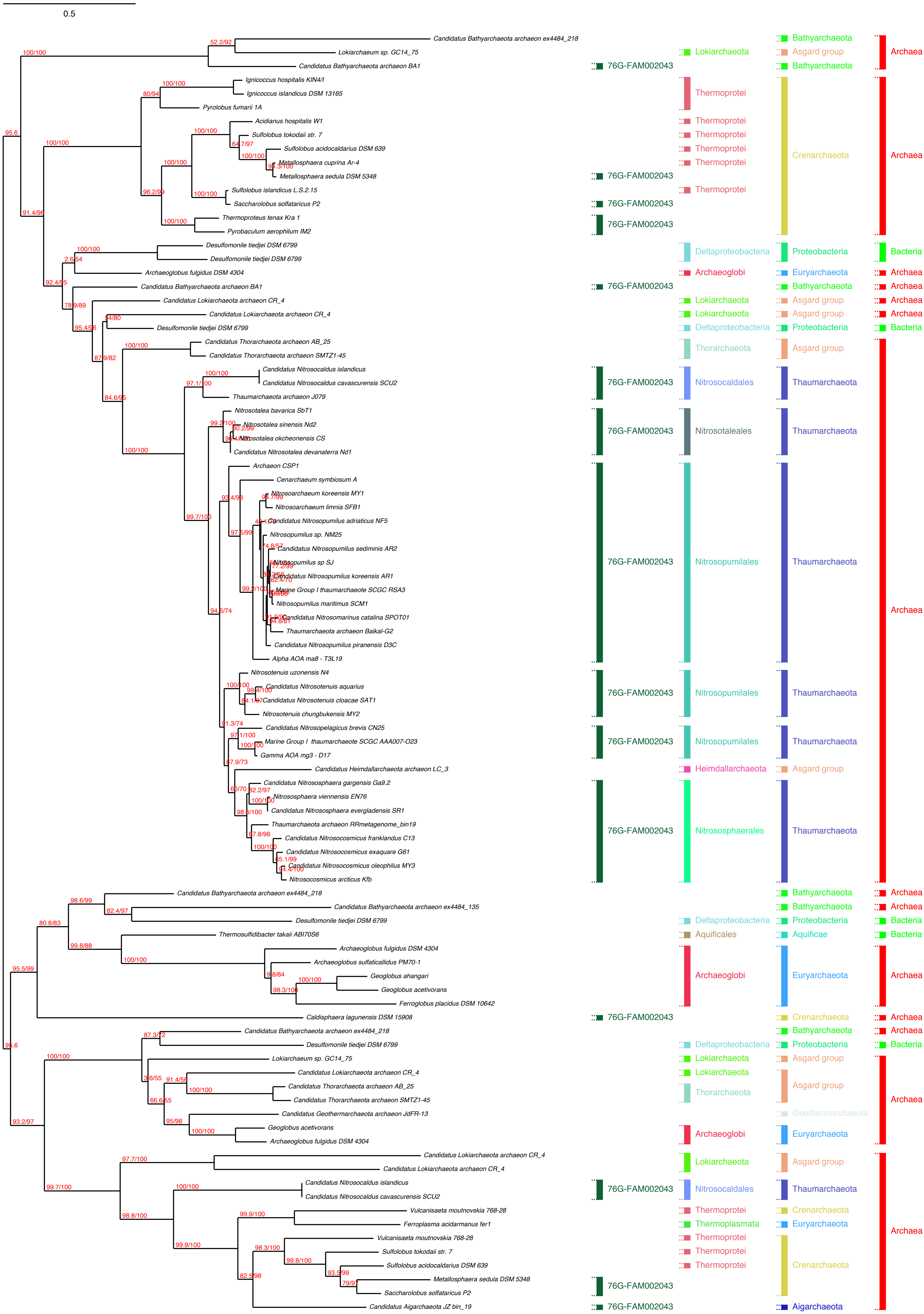

0.5
Candidatus Bathyarchaeota archaeon ex4484_218
Lokiarchaeum sp. GC14_75
Candidatus Bathyarchaeota archaeon BA1
Ignicoccus hospitalis KIN4/I
Ignicoccus islandicus DSM 13165
Pyrolobus fumarii 1A
Acidianus hospitalis W1
Sulfolobus tokodaii str. 7
Sulfolobus acidocaldarius DSM 639
Metallosphaera cuprina Ar-4
Metallosphaera sedula DSM 5348
Sulfolobus islandicus L.S.2.15
Saccharolobus solfataricus P2
Thermoproteus tenax Kra 1
Pyrobaculum aerophilum IM2
Desulfomonile tiedjei DSM 6799
Desulfomonile tiedjei DSM 6799
Archaeoglobus fulgidus DSM 4304
Candidatus Bathyarchaeota archaeon BA1
Candidatus Lokiarchaeota archaeon CR_4
Candidatus Lokiarchaeota archaeon CR_4
Desulfomonile tiedjei DSM 6799
Candidatus Thorarchaeota archaeon AB_25
Candidatus Thorarchaeota archaeon SMTZ1-45
Candidatus Nitrosocaldus islandicus
Candidatus Nitrosocaldus cavascurensis SCU2
Thaumarchaeota archaeon J079
Nitrosotalea bavarica SbT1
Nitrosotalea sinensis Nd2
Nitrosotalea okcheonensis CS
Candidatus Nitrosotalea devanaterra Nd1
Archaeon CSP1
Cenarchaeum symbiosum A
Nitrosoarchaeum koreensis MY1
Nitrosoarchaeum limnia SFB1
Candidatus Nitrosopumilus adriaticus NF5
Nitrosopumilus sp. NM25
Candidatus Nitrosopumilus sediminis AR2
Nitrosopumilus sp SJ
Candidatus Nitrosopumilus koreensis AR1
Marine Group I thaumarchaeote SCGC RSA3
Nitrosopumilus maritimus SCM1
Candidatus Nitrosomarinus catalina SPOT01
Thaumarchaeota archaeon Baikal-G2
Candidatus Nitrosopumilus piranensis D3C
Alpha AOA ma8 - T3L19
Nitrosotenuis uzonensis N4
Candidatus Nitrosotenuis aquarius
Candidatus Nitrosotenuis cloacae SAT1
Nitrosotenuis chungbukensis MY2
Candidatus Nitrosopelagicus brevis CN25
Marine Group I thaumarchaeote SCGC AAA007-O23
Gamma AOA mg3 - D17
Candidatus Heimdallarchaeota archaeon LC_3
Candidatus Nitrososphaera gargensis Ga9.2
Nitrososphaera viennensis EN76
Candidatus Nitrososphaera evergladensis SR1
Thaumarchaeota archaeon RRmetagenome_bin19
Candidatus Nitrosocosmicus franklandus C13
Candidatus Nitrosocosmicus exaquare G61
Candidatus Nitrosocosmicus oleophilus MY3
Nitrosocosmicus arcticus Kfb
Candidatus Bathyarchaeota archaeon ex4484_218
Candidatus Bathyarchaeota archaeon ex4484_135
Desulfomonile tiedjei DSM 6799
Thermosulfidibacter takaii ABI70S6
Archaeoglobus fulgidus DSM 4304
Archaeoglobus sulfaticallidus PM70-1
Geoglobus ahangari
Geoglobus acetivorans
Ferroglobus placidus DSM 10642
Caldisphaera lagunensis DSM 15908
Candidatus Bathyarchaeota archaeon ex4484_218
Desulfomonile tiedjei DSM 6799
Lokiarchaeum sp. GC14_75
Candidatus Lokiarchaeota archaeon CR_4
Candidatus Thorarchaeota archaeon AB_25
Candidatus Thorarchaeota archaeon SMTZ1-45
Candidatus Geothermarchaeota archaeon JdFR-13
Geoglobus acetivorans
Archaeoglobus fulgidus DSM 4304
Candidatus Lokiarchaeota archaeon CR_4
Candidatus Lokiarchaeota archaeon CR_4
Candidatus Nitrosocaldus islandicus
Candidatus Nitrosocaldus cavascurensis SCU2
Vulcanisaeta moutnovskia 768-28
Ferroplasma acidarmanus fer1
Vulcanisaeta moutnovskia 768-28
Sulfolobus tokodaii str. 7
Sulfolobus acidocaldarius DSM 639
Metallosphaera sedula DSM 5348
Saccharolobus solfataricus P2
Candidatus Aigarchaeota JZ bin_19
76G-FAM002043
Lokiarchaeota
Thermoprotei
Deltaproteobacteria
Archaeoglobi
Thorarchaeota
Nitrosocaldales
Nitrosotaleales
Nitrosopumilales
Heimdallarchaeota
Nitrososphaerales
Aquificales
Thermoplasmata
Bathyarchaeota
Asgard group
Crenarchaeota
Proteobacteria
Euryarchaeota
Thaumarchaeota
Aquificae
Geothermarchaeota
Aigarchaeota
Archaea
Bacteria

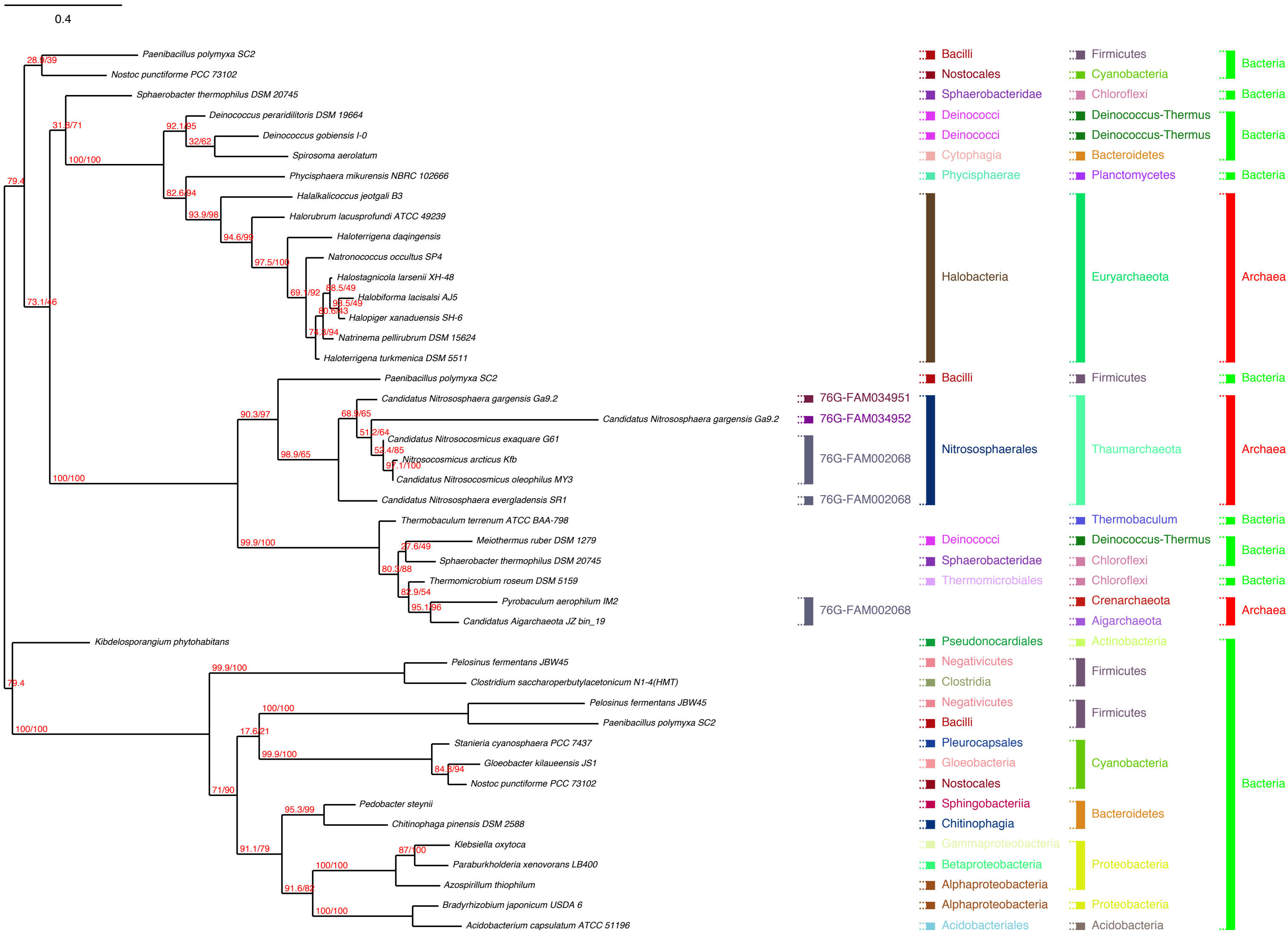
0.4
Paenibacillus polymyxa SC2
Nostoc punctiforme PCC 73102
Sphaerobacter thermophilus DSM 20745
Deinococcus peraridilitoris DSM 19664
Deinococcus gobiensis I-0
Spirosoma aerolatum
Phycisphaera mikurensis NBRC 102666
Halalkalicoccus jeotgali B3
Halorubrum lacusprofundi ATCC 49239
Haloterrigena daqingensis
Natronococcus occultus SP4
Halostagnicola larsenii XH-48
Halobiforma lacisalsi AJ5
Halopiger xanaduensis SH-6
Natrinema pellirubrum DSM 15624
Haloterrigena turkmenica DSM 5511
Paenibacillus polymyxa SC2
Candidatus Nitrososphaera gargensis Ga9.2
Candidatus Nitrososphaera gargensis Ga9.2
Candidatus Nitrosocosmicus exaquare G61
Nitrosocosmicus arcticus Kfb
Candidatus Nitrosocosmicus oleophilus MY3
Candidatus Nitrososphaera evergladensis SR1
Thermobaculum terrenum ATCC BAA-798
Meiothermus ruber DSM 1279
Sphaerobacter thermophilus DSM 20745
Thermomicrobium roseum DSM 5159
Pyrobaculum aerophilum IM2
Candidatus Aigarchaeota JZ bin_19
Kibdelosporangium phytohabitans
Pelosinus fermentans JBW45
Clostridium saccharoperbutylacetonicum N1-4(HMT)
Pelosinus fermentans JBW45
Paenibacillus polymyxa SC2
Stanieria cyanosphaera PCC 7437
Gloeobacter kilaueensis JS1
Nostoc punctiforme PCC 73102
Pedobacter steynii
Chitinophaga pinensis DSM 2588
Klebsiella oxytoca
Paraburkholderia xenovorans LB400
Azospirillum thiophilum
Bradyrhizobium japonicum USDA 6
Acidobacterium capsulatum ATCC 51196
28.9/39
31.8/71
92.1/95
32/62
100/100
79.4
82.6/94
93.9/98
94.6/99
97.5/100
69.1/92
88.5/49
95.5/49
80.6/43
74.3/94
73.1/46
90.3/97
68.9/65
51.2/64
52.4/85
97.1/100
98.9/65
100/100
99.9/100
27.6/49
80.3/88
82.9/54
95.1/96
79.4
99.9/100
100/100
17.6/21
99.9/100
84.8/94
71/90
95.3/99
87/100
100/100
91.1/79
91.6/82
100/100
100/100
76G-FAM034951
76G-FAM034952
76G-FAM002068
76G-FAM002068
76G-FAM002068
Bacilli
Nostocales
Sphaerobacteridae
Deinococci
Deinococci
Cytophagia
Phycisphaerae
Halobacteria
Bacilli
Nitrososphaerales
Deinococci
Sphaerobacteridae
Thermomicrobiales
Pseudonocardiales
Negativicutes
Clostridia
Negativicutes
Bacilli
Pleurocapsales
Gloeobacteria
Nostocales
Sphingobacteriia
Chitinophagia
Gammaproteobacteria
Betaproteobacteria
Alphaproteobacteria
Alphaproteobacteria
Acidobacteriales
Firmicutes
Cyanobacteria
Chloroflexi
Deinococcus-Thermus
Deinococcus-Thermus
Bacteroidetes
Planctomycetes
Euryarchaeota
Firmicutes
Thaumarchaeota
Thermobaculum
Deinococcus-Thermus
Chloroflexi
Chloroflexi
Crenarchaeota
Aigarchaeota
Actinobacteria
Firmicutes
Firmicutes
Cyanobacteria
Bacteroidetes
Proteobacteria
Proteobacteria
Acidobacteria
Bacteria
Bacteria
Bacteria
Bacteria
Archaea
Bacteria
Archaea
Bacteria
Bacteria
Bacteria
Archaea
Bacteria

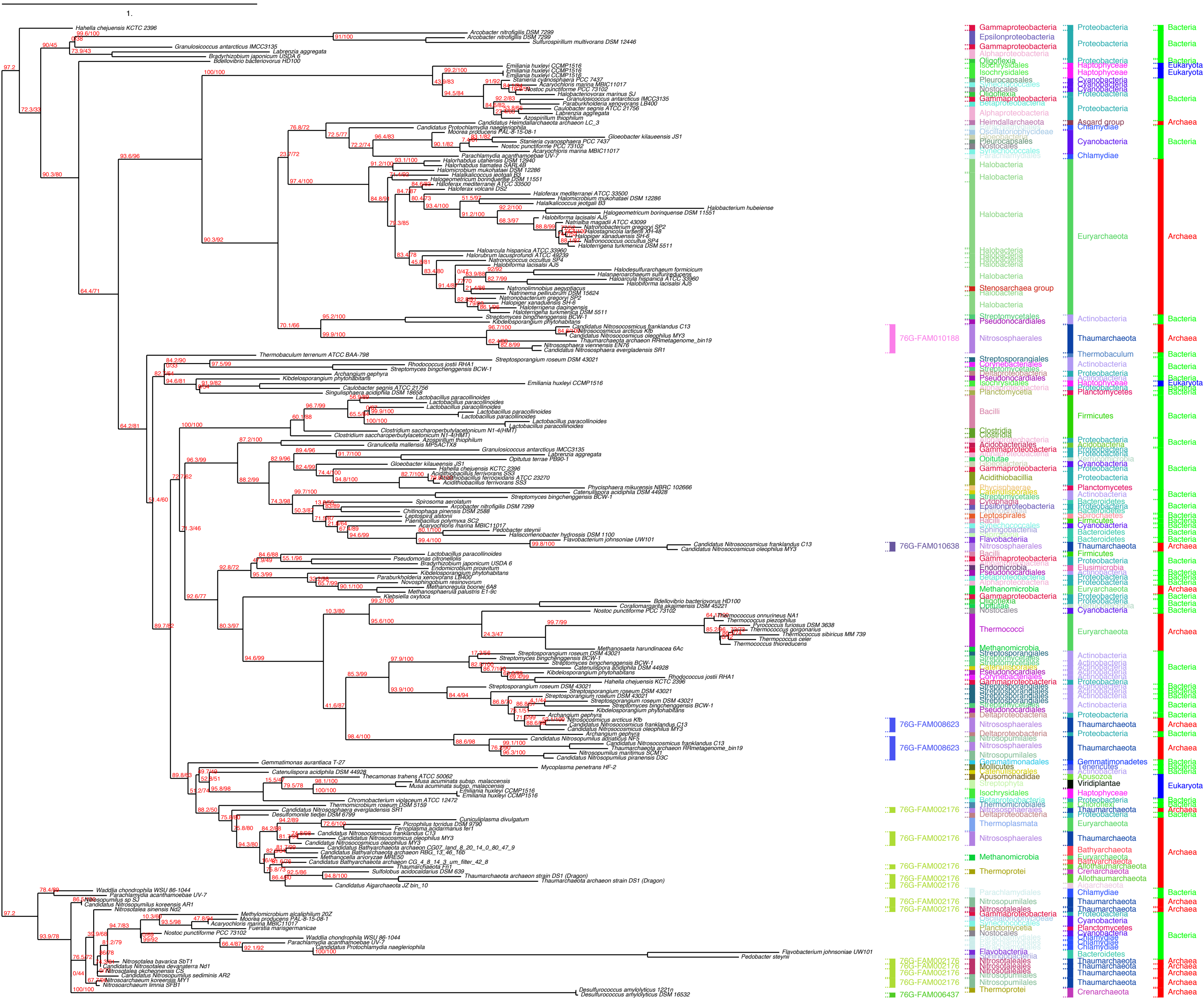
1.

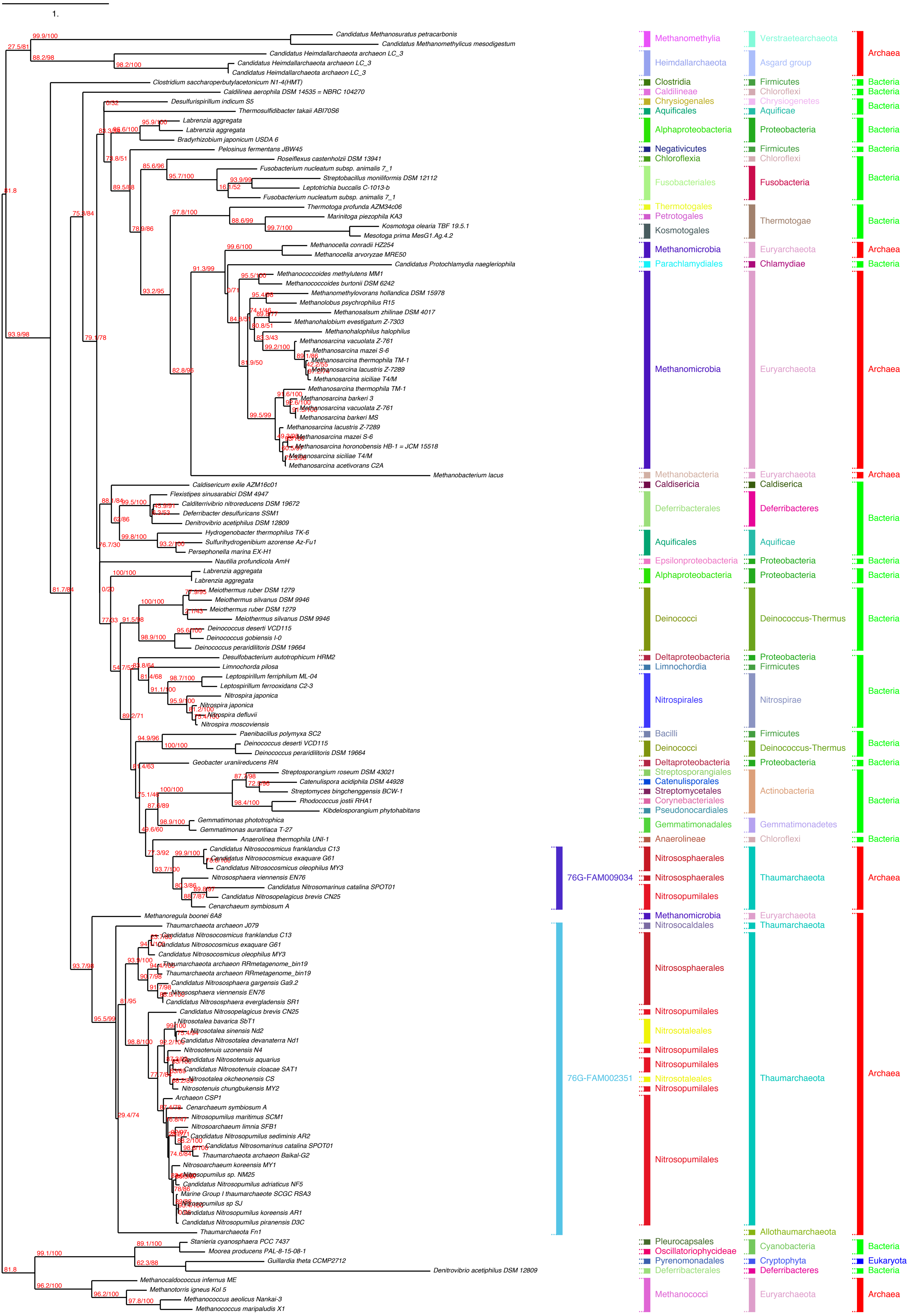
1.
Candidatus Methanosuratus petracarbonis
Candidatus Methanomethylicus mesodigestum
Candidatus Heimdallarchaeota archaeon LC_3
Candidatus Heimdallarchaeota archaeon LC_3
Candidatus Heimdallarchaeota archaeon LC_3
Clostridium saccharoperbutylacetonicum N1-4(HMT)
Caldilinea aerophila DSM 14535 = NBRC 104270
Desulfurispirillum indicum S5
Thermosulfidibacter takaii ABI70S6
Labrenzia aggregata
Labrenzia aggregata
Bradyrhizobium japonicum USDA 6
Pelosinus fermentans JBW45
Roseiflexus castenholzii DSM 13941
Fusobacterium nucleatum subsp. animalis 7_1
Streptobacillus moniliformis DSM 12112
Leptotrichia buccalis C-1013-b
Fusobacterium nucleatum subsp. animalis 7_1
Thermotoga profunda AZM34c06
Marinitoga piezophila KA3
Kosmotoga olearia TBF 19.5.1
Mesotoga prima MesG1.Ag.4.2
Methanocella conradii HZ254
Methanocella arvoryzae MRE50
Candidatus Protochlamydia naegleriophila
Methanococcoides methylutens MM1
Methanococcoides burtonii DSM 6242
Methanomethylovorans hollandica DSM 15978
Methanolobus psychrophilus R15
Methanosalsum zhilinae DSM 4017
Methanohalobium evestigatum Z-7303
Methanohalophilus halophilus
Methanosarcina vacuolata Z-761
Methanosarcina mazei S-6
Methanosarcina thermophila TM-1
Methanosarcina lacustris Z-7289
Methanosarcina siciliae T4/M
Methanosarcina thermophila TM-1
Methanosarcina barkeri 3
Methanosarcina vacuolata Z-761
Methanosarcina barkeri MS
Methanosarcina lacustris Z-7289
Methanosarcina mazei S-6
Methanosarcina horonobensis HB-1 = JCM 15518
Methanosarcina siciliae T4/M
Methanosarcina acetivorans C2A
Methanobacterium lacus
Caldisericum exile AZM16c01
Flexistipes sinusarabici DSM 4947
Calditerrivibrio nitroreducens DSM 19672
Deferribacter desulfuricans SSM1
Denitrovibrio acetiphilus DSM 12809
Hydrogenobacter thermophilus TK-6
Sulfurihydrogenibium azorense Az-Fu1
Persephonella marina EX-H1
Nautilia profundicola AmH
Labrenzia aggregata
Labrenzia aggregata
Meiothermus ruber DSM 1279
Meiothermus silvanus DSM 9946
Meiothermus ruber DSM 1279
Meiothermus silvanus DSM 9946
Deinococcus deserti VCD115
Deinococcus gobiensis I-0
Deinococcus peraridilitoris DSM 19664
Desulfobacterium autotrophicum HRM2
Limnochorda pilosa
Leptospirillum ferriphilum ML-04
Leptospirillum ferrooxidans C2-3
Nitrospira japonica
Nitrospira japonica
Nitrospira defluvii
Nitrospira moscoviensis
Paenibacillus polymyxa SC2
Deinococcus deserti VCD115
Deinococcus peraridilitoris DSM 19664
Geobacter uraniireducens Rf4
Streptosporangium roseum DSM 43021
Catenulispora acidiphila DSM 44928
Streptomyces bingchenggensis BCW-1
Rhodococcus jostii RHA1
Kibdelosporangium phytohabitans
Gemmatimonas phototrophica
Gemmatimonas aurantiaca T-27
Anaerolinea thermophila UNI-1
Candidatus Nitrosocosmicus franklandus C13
Candidatus Nitrosocosmicus exaquare G61
Candidatus Nitrosocosmicus oleophilus MY3
Nitrososphaera viennensis EN76
Candidatus Nitrosomarinus catalina SPOT01
Candidatus Nitrosopelagicus brevis CN25
Cenarchaeum symbiosum A
Methanoregula boonei 6A8
Thaumarchaeota archaeon J079
Candidatus Nitrosocosmicus franklandus C13
Candidatus Nitrosocosmicus exaquare G61
Candidatus Nitrosocosmicus oleophilus MY3
Thaumarchaeota archaeon RRmetagenome_bin19
Thaumarchaeota archaeon RRmetagenome_bin19
Candidatus Nitrososphaera gargensis Ga9.2
Nitrososphaera viennensis EN76
Candidatus Nitrososphaera evergladensis SR1
Candidatus Nitrosopelagicus brevis CN25
Nitrosotalea bavarica SbT1
Nitrosotalea sinensis Nd2
Candidatus Nitrosotalea devanaterra Nd1
Nitrosotenuis uzonensis N4
Candidatus Nitrosotenuis aquarius
Candidatus Nitrosotenuis cloacae SAT1
Nitrosotalea okcheonensis CS
Nitrosotenuis chungbukensis MY2
Archaeon CSP1
Cenarchaeum symbiosum A
Nitrosopumilus maritimus SCM1
Nitrosoarchaeum limnia SFB1
Candidatus Nitrosopumilus sediminis AR2
Candidatus Nitrosomarinus catalina SPOT01
Thaumarchaeota archaeon Baikal-G2
Nitrosoarchaeum koreensis MY1
Nitrosopumilus sp. NM25
Candidatus Nitrosopumilus adriaticus NF5
Marine Group I thaumarchaeote SCGC RSA3
Nitrosopumilus sp SJ
Candidatus Nitrosopumilus koreensis AR1
Candidatus Nitrosopumilus piranensis D3C
Thaumarchaeota Fn1
Stanieria cyanosphaera PCC 7437
Moorea producens PAL-8-15-08-1
Guillardia theta CCMP2712
Denitrovibrio acetiphilus DSM 12809
Methanocaldococcus infernus ME
Methanotorris igneus Kol 5
Methanococcus aeolicus Nankai-3
Methanococcus maripaludis X1
76G-FAM009034
76G-FAM002351
Methanomethylia
Heimdallarchaeota
Clostridia
Caldilineae
Chrysiogenales
Aquificales
Alphaproteobacteria
Negativicutes
Chloroflexia
Fusobacteriales
Thermotogales
Petrotogales
Kosmotogales
Methanomicrobia
Parachlamydiales
Methanomicrobia
Methanobacteria
Caldisericia
Deferribacterales
Aquificales
Epsilonproteobacteria
Alphaproteobacteria
Deinococci
Deltaproteobacteria
Limnochordia
Nitrospirales
Bacilli
Deinococci
Deltaproteobacteria
Streptosporangiales
Catenulisporales
Streptomycetales
Corynebacteriales
Pseudonocardiales
Gemmatimonadales
Anaerolineae
Nitrososphaerales
Nitrososphaerales
Nitrosopumilales
Methanomicrobia
Nitrosocaldales
Nitrososphaerales
Nitrosopumilales
Nitrosotaleales
Nitrosopumilales
Nitrosopumilales
Nitrosotaleales
Nitrosopumilales
Nitrosopumilales
Pleurocapsales
Oscillatoriophycideae
Pyrenomonadales
Deferribacterales
Methanococci
Verstraetearchaeota
Asgard group
Firmicutes
Chloroflexi
Chrysiogenetes
Aquificae
Proteobacteria
Firmicutes
Chloroflexi
Fusobacteria
Thermotogae
Euryarchaeota
Chlamydiae
Euryarchaeota
Euryarchaeota
Caldiserica
Deferribacteres
Aquificae
Proteobacteria
Proteobacteria
Deinococcus-Thermus
Proteobacteria
Firmicutes
Nitrospirae
Firmicutes
Deinococcus-Thermus
Proteobacteria
Actinobacteria
Gemmatimonadetes
Chloroflexi
Thaumarchaeota
Euryarchaeota
Thaumarchaeota
Thaumarchaeota
Allothaumarchaeota
Cyanobacteria
Cryptophyta
Deferribacteres
Euryarchaeota
Archaea
Bacteria
Bacteria
Bacteria
Bacteria
Bacteria
Bacteria
Bacteria
Archaea
Bacteria
Archaea
Archaea
Bacteria
Bacteria
Bacteria
Bacteria
Bacteria
Bacteria
Bacteria
Bacteria
Bacteria
Archaea
Archaea
Bacteria
Eukaryota
Bacteria
Archaea

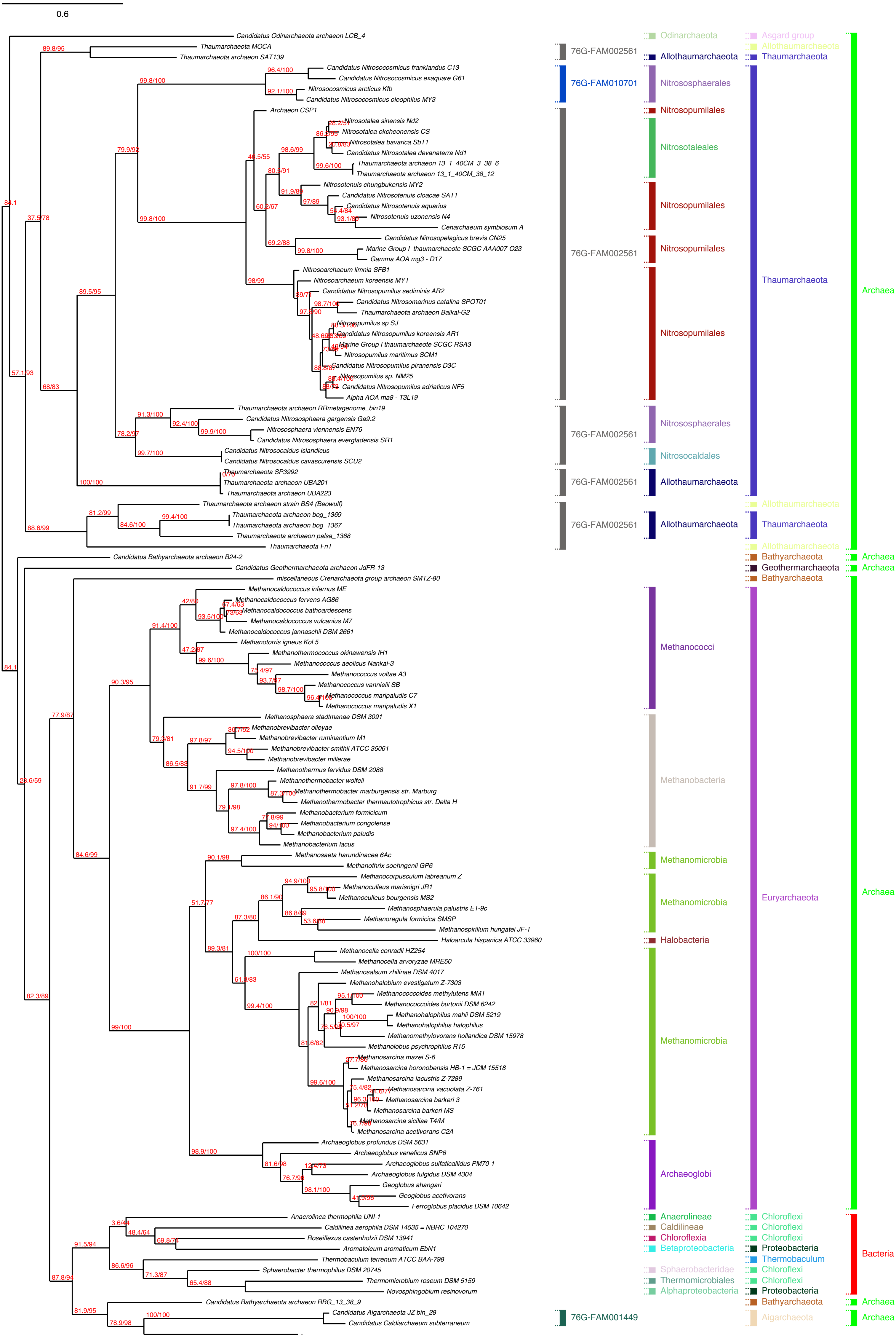

0.6
Candidatus Odinarchaeota archaeon LCB_4
Thaumarchaeota MOCA
Thaumarchaeota archaeon SAT139
Candidatus Nitrosocosmicus franklandus C13
Candidatus Nitrosocosmicus exaquare G61
Nitrosocosmicus arcticus Kfb
Candidatus Nitrosocosmicus oleophilus MY3
Archaeon CSP1
Nitrosotalea sinensis Nd2
Nitrosotalea okcheonensis CS
Nitrosotalea bavarica SbT1
Candidatus Nitrosotalea devanaterra Nd1
Thaumarchaeota archaeon 13_1_40CM_3_38_6
Thaumarchaeota archaeon 13_1_40CM_38_12
Nitrosotenuis chungbukensis MY2
Candidatus Nitrosotenuis cloacae SAT1
Candidatus Nitrosotenuis aquarius
Nitrosotenuis uzonensis N4
Cenarchaeum symbiosum A
Candidatus Nitrosopelagicus brevis CN25
Marine Group I thaumarchaeote SCGC AAA007-O23
Gamma AOA mg3 - D17
Nitrosoarchaeum limnia SFB1
Nitrosoarchaeum koreensis MY1
Candidatus Nitrosopumilus sediminis AR2
Candidatus Nitrosomarinus catalina SPOT01
Thaumarchaeota archaeon Baikal-G2
Nitrosopumilus sp SJ
Candidatus Nitrosopumilus koreensis AR1
Marine Group I thaumarchaeote SCGC RSA3
Nitrosopumilus maritimus SCM1
Candidatus Nitrosopumilus piranensis D3C
Nitrosopumilus sp. NM25
Candidatus Nitrosopumilus adriaticus NF5
Alpha AOA ma8 - T3L19
Thaumarchaeota archaeon RRmetagenome_bin19
Candidatus Nitrososphaera gargensis Ga9.2
Nitrososphaera viennensis EN76
Candidatus Nitrososphaera evergladensis SR1
Candidatus Nitrosocaldus islandicus
Candidatus Nitrosocaldus cavascurensis SCU2
Thaumarchaeota SP3992
Thaumarchaeota archaeon UBA201
Thaumarchaeota archaeon UBA223
Thaumarchaeota archaeon strain BS4 (Beowulf)
Thaumarchaeota archaeon bog_1369
Thaumarchaeota archaeon bog_1367
Thaumarchaeota archaeon palsa_1368
Thaumarchaeota Fn1
Candidatus Bathyarchaeota archaeon B24-2
Candidatus Geothermarchaeota archaeon JdFR-13
miscellaneous Crenarchaeota group archaeon SMTZ-80
Methanocaldococcus infernus ME
Methanocaldococcus fervens AG86
Methanocaldococcus bathoardescens
Methanocaldococcus vulcanius M7
Methanocaldococcus jannaschii DSM 2661
Methanotorris igneus Kol 5
Methanothermococcus okinawensis IH1
Methanococcus aeolicus Nankai-3
Methanococcus voltae A3
Methanococcus vannielii SB
Methanococcus maripaludis C7
Methanococcus maripaludis X1
Methanosphaera stadtmanae DSM 3091
Methanobrevibacter olleyae
Methanobrevibacter ruminantium M1
Methanobrevibacter smithii ATCC 35061
Methanobrevibacter millerae
Methanothermus fervidus DSM 2088
Methanothermobacter wolfeii
Methanothermobacter marburgensis str. Marburg
Methanothermobacter thermautotrophicus str. Delta H
Methanobacterium formicicum
Methanobacterium congolense
Methanobacterium paludis
Methanobacterium lacus
Methanosaeta harundinacea 6Ac
Methanothrix soehngenii GP6
Methanocorpusculum labreanum Z
Methanoculleus marisnigri JR1
Methanoculleus bourgensis MS2
Methanosphaerula palustris E1-9c
Methanoregula formicica SMSP
Methanospirillum hungatei JF-1
Haloarcula hispanica ATCC 33960
Methanocella conradii HZ254
Methanocella arvoryzae MRE50
Methanosalsum zhilinae DSM 4017
Methanohalobium evestigatum Z-7303
Methanococcoides methylutens MM1
Methanococcoides burtonii DSM 6242
Methanohalophilus mahii DSM 5219
Methanohalophilus halophilus
Methanomethylovorans hollandica DSM 15978
Methanolobus psychrophilus R15
Methanosarcina mazei S-6
Methanosarcina horonobensis HB-1 = JCM 15518
Methanosarcina lacustris Z-7289
Methanosarcina vacuolata Z-761
Methanosarcina barkeri 3
Methanosarcina barkeri MS
Methanosarcina siciliae T4/M
Methanosarcina acetivorans C2A
Archaeoglobus profundus DSM 5631
Archaeoglobus veneficus SNP6
Archaeoglobus sulfaticallidus PM70-1
Archaeoglobus fulgidus DSM 4304
Geoglobus ahangari
Geoglobus acetivorans
Ferroglobus placidus DSM 10642
Anaerolinea thermophila UNI-1
Caldilinea aerophila DSM 14535 = NBRC 104270
Roseiflexus castenholzii DSM 13941
Aromatoleum aromaticum EbN1
Thermobaculum terrenum ATCC BAA-798
Sphaerobacter thermophilus DSM 20745
Thermomicrobium roseum DSM 5159
Novosphingobium resinovorum
Candidatus Bathyarchaeota archaeon RBG_13_38_9
Candidatus Aigarchaeota JZ bin_28
Candidatus Caldiarchaeum subterraneum
76G-FAM002561
76G-FAM010701
76G-FAM002561
76G-FAM002561
76G-FAM002561
76G-FAM002561
76G-FAM001449
Odinarchaeota
Allothaumarchaeota
Nitrososphaerales
Nitrosopumilales
Nitrosotaleales
Nitrosopumilales
Nitrosopumilales
Nitrosopumilales
Nitrososphaerales
Nitrosocaldales
Allothaumarchaeota
Allothaumarchaeota
Methanococci
Methanobacteria
Methanomicrobia
Methanomicrobia
Halobacteria
Methanomicrobia
Archaeoglobi
Anaerolineae
Caldilineae
Chloroflexia
Betaproteobacteria
Sphaerobacteridae
Thermomicrobiales
Alphaproteobacteria
Asgard group
Allothaumarchaeota
Thaumarchaeota
Thaumarchaeota
Allothaumarchaeota
Thaumarchaeota
Allothaumarchaeota
Bathyarchaeota
Geothermarchaeota
Bathyarchaeota
Euryarchaeota
Chloroflexi
Chloroflexi
Chloroflexi
Proteobacteria
Thermobaculum
Chloroflexi
Chloroflexi
Proteobacteria
Bathyarchaeota
Aigarchaeota
Archaea
Archaea
Archaea
Archaea
Bacteria
Archaea
Archaea

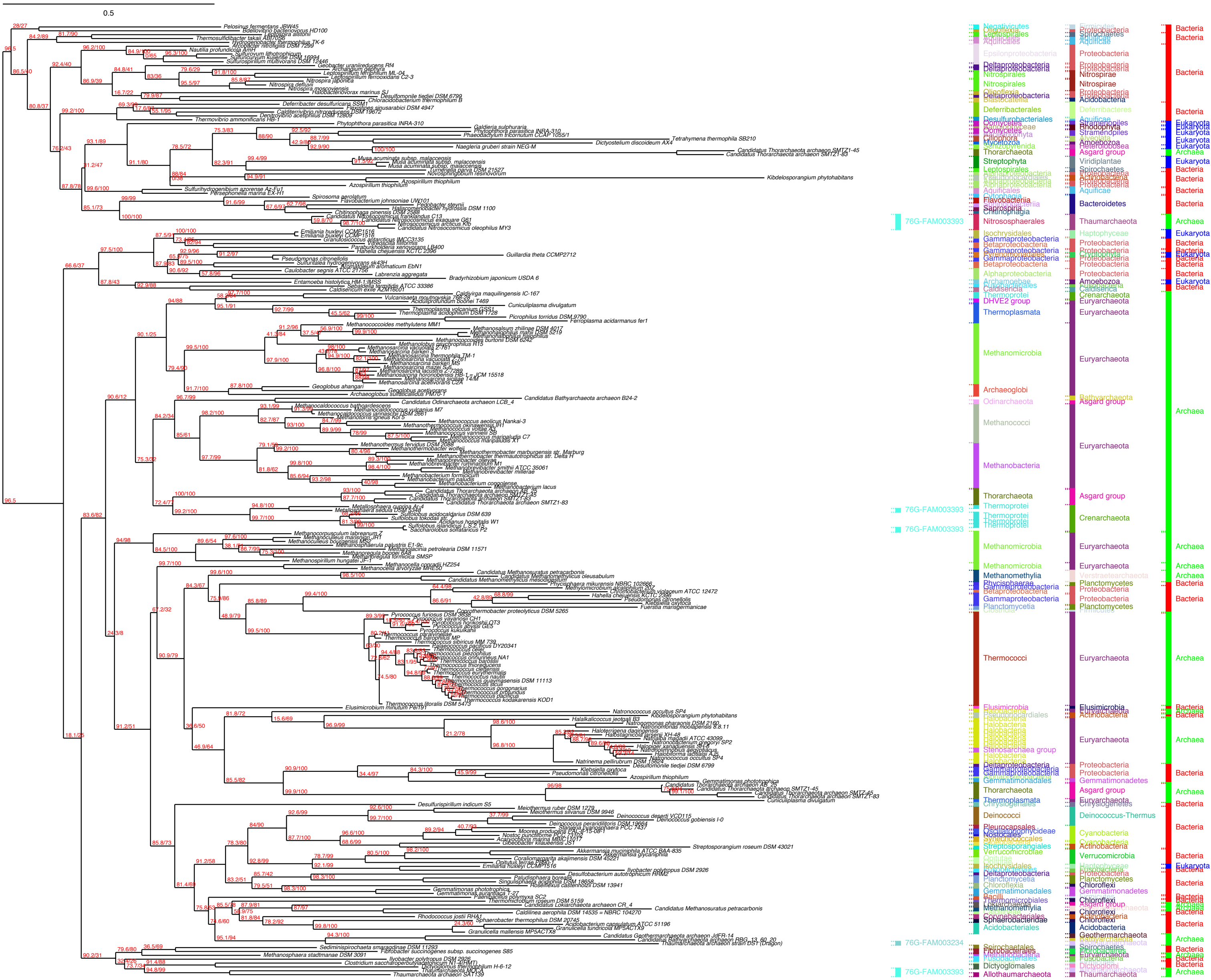

0.5
76G-FAM003393
76G-FAM003234
Bacteria
Archaea
Eukaryota
Proteobacteria
Nitrospirae
Acidobacteria
Deferribacteres
Thaumarchaeota
Bacteroidetes
Euryarchaeota
Crenarchaeota
Asgard group
Thermococci
Methanomicrobia
Methanococci
Methanobacteria
Thermoplasmata
Archaeoglobi
Thorarchaeota
Thermoprotei
Halobacteria
Deinococcus-Thermus
Cyanobacteria
Verrucomicrobia
Planctomycetes
Gemmatimonadetes
Chloroflexi
Actinobacteria
Viridiplantae
Amoebozoa
Haptophyceae
Deinococci
Acidobacteriales
Allothaumarchaeota
Dictyoglomales

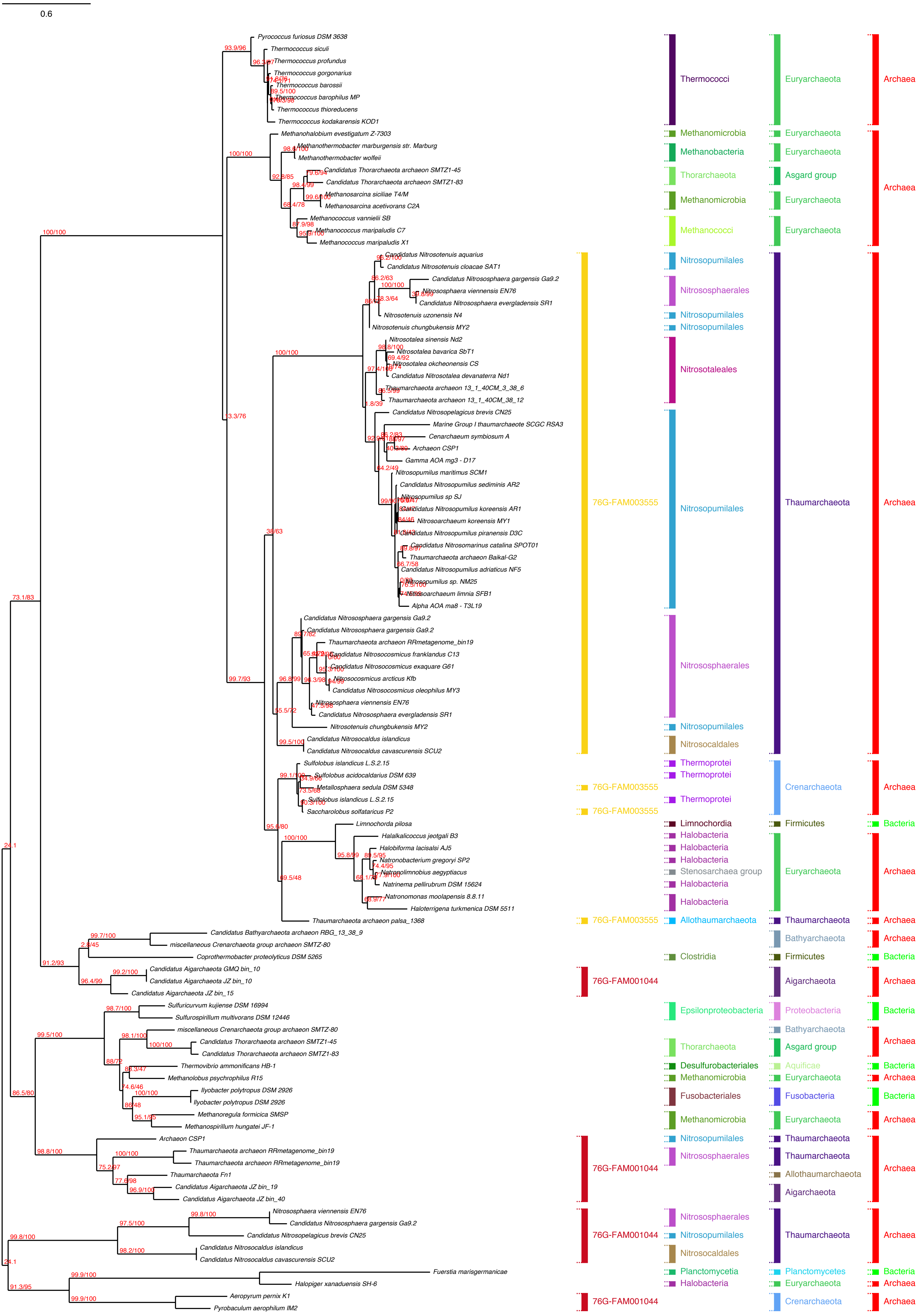

0.6
Pyrococcus furiosus DSM 3638
Thermococcus siculi
Thermococcus profundus
Thermococcus gorgonarius
Thermococcus barossii
Thermococcus barophilus MP
Thermococcus thioreducens
Thermococcus kodakarensis KOD1
Methanohalobium evestigatum Z-7303
Methanothermobacter marburgensis str. Marburg
Methanothermobacter wolfeii
Candidatus Thorarchaeota archaeon SMTZ1-45
Candidatus Thorarchaeota archaeon SMTZ1-83
Methanosarcina siciliae T4/M
Methanosarcina acetivorans C2A
Methanococcus vannielii SB
Methanococcus maripaludis C7
Methanococcus maripaludis X1
Candidatus Nitrosotenuis aquarius
Candidatus Nitrosotenuis cloacae SAT1
Candidatus Nitrososphaera gargensis Ga9.2
Nitrososphaera viennensis EN76
Candidatus Nitrososphaera evergladensis SR1
Nitrosotenuis uzonensis N4
Nitrosotenuis chungbukensis MY2
Nitrosotalea sinensis Nd2
Nitrosotalea bavarica SbT1
Nitrosotalea okcheonensis CS
Candidatus Nitrosotalea devanaterra Nd1
Thaumarchaeota archaeon 13_1_40CM_3_38_6
Thaumarchaeota archaeon 13_1_40CM_38_12
Candidatus Nitrosopelagicus brevis CN25
Marine Group I thaumarchaeote SCGC RSA3
Cenarchaeum symbiosum A
Archaeon CSP1
Gamma AOA mg3 - D17
Nitrosopumilus maritimus SCM1
Candidatus Nitrosopumilus sediminis AR2
Nitrosopumilus sp SJ
Candidatus Nitrosopumilus koreensis AR1
Nitrosoarchaeum koreensis MY1
Candidatus Nitrosopumilus piranensis D3C
Candidatus Nitrosomarinus catalina SPOT01
Thaumarchaeota archaeon Baikal-G2
Candidatus Nitrosopumilus adriaticus NF5
Nitrosopumilus sp. NM25
Nitrosoarchaeum limnia SFB1
Alpha AOA ma8 - T3L19
Candidatus Nitrososphaera gargensis Ga9.2
Candidatus Nitrososphaera gargensis Ga9.2
Thaumarchaeota archaeon RRmetagenome_bin19
Candidatus Nitrosocosmicus franklandus C13
Candidatus Nitrosocosmicus exaquare G61
Nitrosocosmicus arcticus Kfb
Candidatus Nitrosocosmicus oleophilus MY3
Nitrososphaera viennensis EN76
Candidatus Nitrososphaera evergladensis SR1
Nitrosotenuis chungbukensis MY2
Candidatus Nitrosocaldus islandicus
Candidatus Nitrosocaldus cavascurensis SCU2
Sulfolobus islandicus L.S.2.15
Sulfolobus acidocaldarius DSM 639
Metallosphaera sedula DSM 5348
Sulfolobus islandicus L.S.2.15
Saccharolobus solfataricus P2
Limnochorda pilosa
Halalkalicoccus jeotgali B3
Halobiforma lacisalsi AJ5
Natronobacterium gregoryi SP2
Natronolimnobius aegyptiacus
Natrinema pellirubrum DSM 15624
Natronomonas moolapensis 8.8.11
Haloterrigena turkmenica DSM 5511
Thaumarchaeota archaeon palsa_1368
Candidatus Bathyarchaeota archaeon RBG_13_38_9
miscellaneous Crenarchaeota group archaeon SMTZ-80
Coprothermobacter proteolyticus DSM 5265
Candidatus Aigarchaeota GMQ bin_10
Candidatus Aigarchaeota JZ bin_10
Candidatus Aigarchaeota JZ bin_15
Sulfuricurvum kujiense DSM 16994
Sulfurospirillum multivorans DSM 12446
miscellaneous Crenarchaeota group archaeon SMTZ-80
Candidatus Thorarchaeota archaeon SMTZ1-45
Candidatus Thorarchaeota archaeon SMTZ1-83
Thermovibrio ammonificans HB-1
Methanolobus psychrophilus R15
Ilyobacter polytropus DSM 2926
Ilyobacter polytropus DSM 2926
Methanoregula formicica SMSP
Methanospirillum hungatei JF-1
Archaeon CSP1
Thaumarchaeota archaeon RRmetagenome_bin19
Thaumarchaeota archaeon RRmetagenome_bin19
Thaumarchaeota Fn1
Candidatus Aigarchaeota JZ bin_19
Candidatus Aigarchaeota JZ bin_40
Nitrososphaera viennensis EN76
Candidatus Nitrososphaera gargensis Ga9.2
Candidatus Nitrosopelagicus brevis CN25
Candidatus Nitrosocaldus islandicus
Candidatus Nitrosocaldus cavascurensis SCU2
Fuerstia marisgermanicae
Halopiger xanaduensis SH-6
Aeropyrum pernix K1
Pyrobaculum aerophilum IM2
76G-FAM003555
76G-FAM003555
76G-FAM003555
76G-FAM003555
76G-FAM001044
76G-FAM001044
76G-FAM001044
76G-FAM001044
Thermococci
Methanomicrobia
Methanobacteria
Thorarchaeota
Methanomicrobia
Methanococci
Nitrosopumilales
Nitrososphaerales
Nitrosopumilales
Nitrosopumilales
Nitrosotaleales
Nitrosopumilales
Nitrososphaerales
Nitrosopumilales
Nitrosocaldales
Thermoprotei
Thermoprotei
Thermoprotei
Limnochorda
Halobacteria
Halobacteria
Halobacteria
Stenosarchaea group
Halobacteria
Halobacteria
Allothaumarchaeota
Clostridia
Epsilonproteobacteria
Thorarchaeota
Desulfurobacteriales
Methanomicrobia
Fusobacteriales
Methanomicrobia
Nitrosopumilales
Nitrososphaerales
Nitrososphaerales
Nitrosopumilales
Nitrosocaldales
Planctomycetia
Halobacteria
Euryarchaeota
Euryarchaeota
Euryarchaeota
Asgard group
Euryarchaeota
Euryarchaeota
Thaumarchaeota
Crenarchaeota
Firmicutes
Euryarchaeota
Thaumarchaeota
Bathyarchaeota
Firmicutes
Aigarchaeota
Proteobacteria
Bathyarchaeota
Asgard group
Aquificae
Euryarchaeota
Fusobacteria
Euryarchaeota
Thaumarchaeota
Thaumarchaeota
Allothaumarchaeota
Aigarchaeota
Thaumarchaeota
Planctomycetes
Euryarchaeota
Crenarchaeota
Archaea
Archaea
Archaea
Archaea
Bacteria
Archaea
Archaea
Archaea
Bacteria
Archaea
Bacteria
Archaea
Bacteria
Archaea
Bacteria
Archaea
Archaea
Archaea
Bacteria
Archaea
Archaea

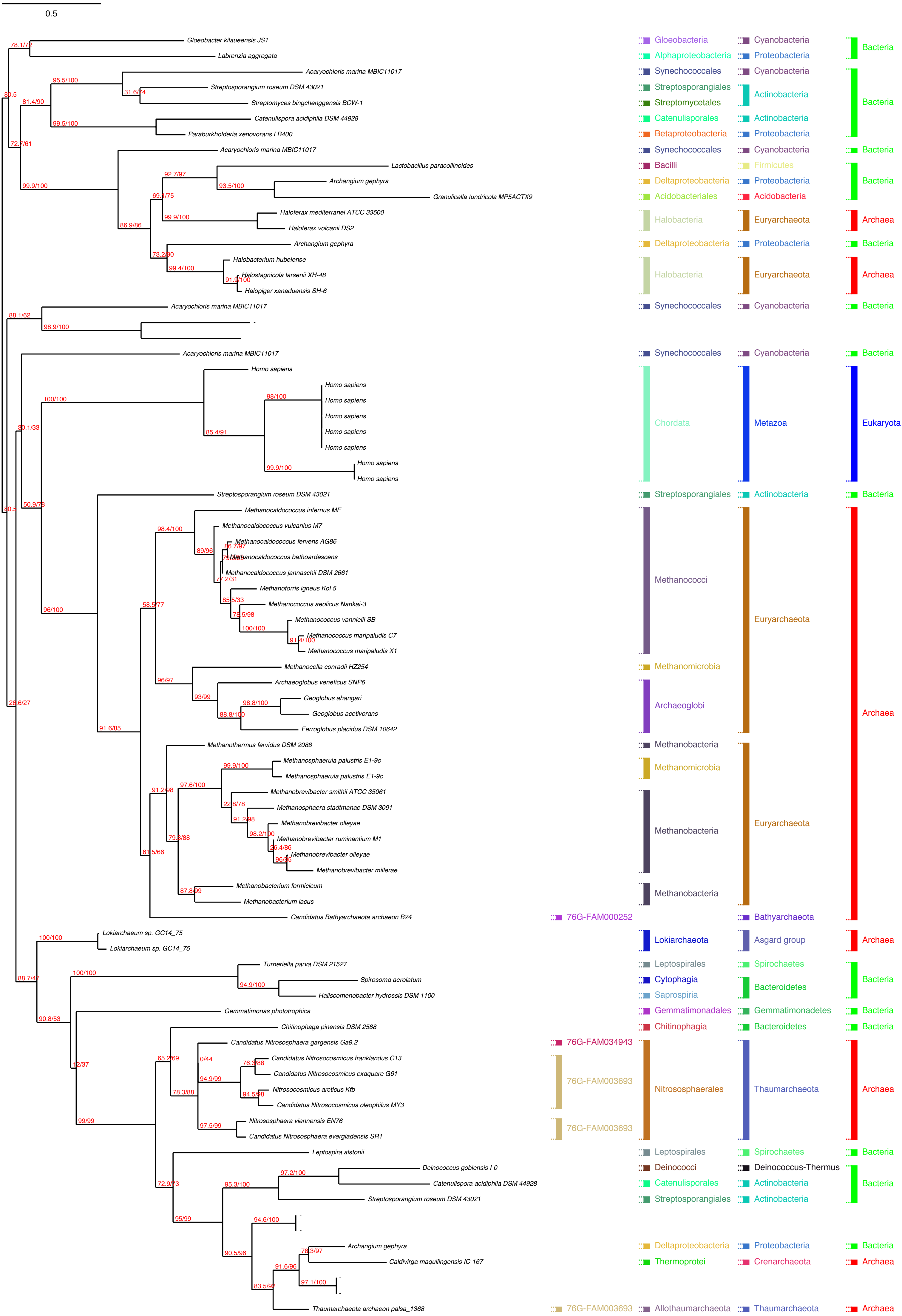
0.5
Gloeobacter kilaueensis JS1
Gloeobacteria
Cyanobacteria
Bacteria
Labrenzia aggregata
Alphaproteobacteria
Proteobacteria
Acaryochloris marina MBIC11017
Synechococcales
Cyanobacteria
Streptosporangium roseum DSM 43021
Streptosporangiales
Actinobacteria
Bacteria
Streptomyces bingchenggensis BCW-1
Streptomycetales
Catenulispora acidiphila DSM 44928
Catenulisporales
Actinobacteria
Paraburkholderia xenovorans LB400
Betaproteobacteria
Proteobacteria
Acaryochloris marina MBIC11017
Synechococcales
Cyanobacteria
Bacteria
Lactobacillus paracollinoides
Bacilli
Firmicutes
Archangium gephyra
Deltaproteobacteria
Proteobacteria
Bacteria
Granulicella tundricola MP5ACTX9
Acidobacteriales
Acidobacteria
Haloferax mediterranei ATCC 33500
Haloferax volcanii DS2
Halobacteria
Euryarchaeota
Archaea
Archangium gephyra
Deltaproteobacteria
Proteobacteria
Bacteria
Halobacterium hubeiense
Halostagnicola larsenii XH-48
Halopiger xanaduensis SH-6
Halobacteria
Euryarchaeota
Archaea
Acaryochloris marina MBIC11017
Synechococcales
Cyanobacteria
Bacteria
-
-
Acaryochloris marina MBIC11017
Synechococcales
Cyanobacteria
Bacteria
Homo sapiens
Homo sapiens
Homo sapiens
Homo sapiens
Homo sapiens
Homo sapiens
Homo sapiens
Homo sapiens
Chordata
Metazoa
Eukaryota
Streptosporangium roseum DSM 43021
Streptosporangiales
Actinobacteria
Bacteria
Methanocaldococcus infernus ME
Methanocaldococcus vulcanius M7
Methanocaldococcus fervens AG86
Methanocaldococcus bathoardescens
Methanocaldococcus jannaschii DSM 2661
Methanotorris igneus Kol 5
Methanococcus aeolicus Nankai-3
Methanococcus vannielii SB
Methanococcus maripaludis C7
Methanococcus maripaludis X1
Methanococci
Euryarchaeota
Archaea
Methanocella conradii HZ254
Methanomicrobia
Archaeoglobus veneficus SNP6
Geoglobus ahangari
Geoglobus acetivorans
Ferroglobus placidus DSM 10642
Archaeoglobi
Methanothermus fervidus DSM 2088
Methanobacteria
Methanosphaerula palustris E1-9c
Methanosphaerula palustris E1-9c
Methanomicrobia
Methanobrevibacter smithii ATCC 35061
Methanosphaera stadtmanae DSM 3091
Methanobrevibacter olleyae
Methanobrevibacter ruminantium M1
Methanobrevibacter olleyae
Methanobrevibacter millerae
Methanobacteria
Euryarchaeota
Methanobacterium formicicum
Methanobacterium lacus
Methanobacteria
Candidatus Bathyarchaeota archaeon B24
76G-FAM000252
Bathyarchaeota
Lokiarchaeum sp. GC14_75
Lokiarchaeum sp. GC14_75
Lokiarchaeota
Asgard group
Archaea
Turneriella parva DSM 21527
Leptospirales
Spirochaetes
Spirosoma aerolatum
Cytophagia
Bacteroidetes
Bacteria
Haliscomenobacter hydrossis DSM 1100
Saprospiria
Gemmatimonas phototrophica
Gemmatimonadales
Gemmatimonadetes
Bacteria
Chitinophaga pinensis DSM 2588
Chitinophagia
Bacteroidetes
Bacteria
Candidatus Nitrososphaera gargensis Ga9.2
76G-FAM034943
Candidatus Nitrosocosmicus franklandus C13
Candidatus Nitrosocosmicus exaquare G61
Nitrosocosmicus arcticus Kfb
Candidatus Nitrosocosmicus oleophilus MY3
76G-FAM003693
Nitrososphaerales
Thaumarchaeota
Archaea
Nitrososphaera viennensis EN76
Candidatus Nitrososphaera evergladensis SR1
76G-FAM003693
Leptospira alstonii
Leptospirales
Spirochaetes
Bacteria
Deinococcus gobiensis I-0
Deinococci
Deinococcus-Thermus
Catenulispora acidiphila DSM 44928
Catenulisporales
Actinobacteria
Bacteria
Streptosporangium roseum DSM 43021
Streptosporangiales
Actinobacteria
-
-
Archangium gephyra
Deltaproteobacteria
Proteobacteria
Bacteria
Caldivirga maquilingensis IC-167
Thermoprotei
Crenarchaeota
Archaea
-
-
Thaumarchaeota archaeon palsa_1368
76G-FAM003693
Allothaumarchaeota
Thaumarchaeota
Archaea

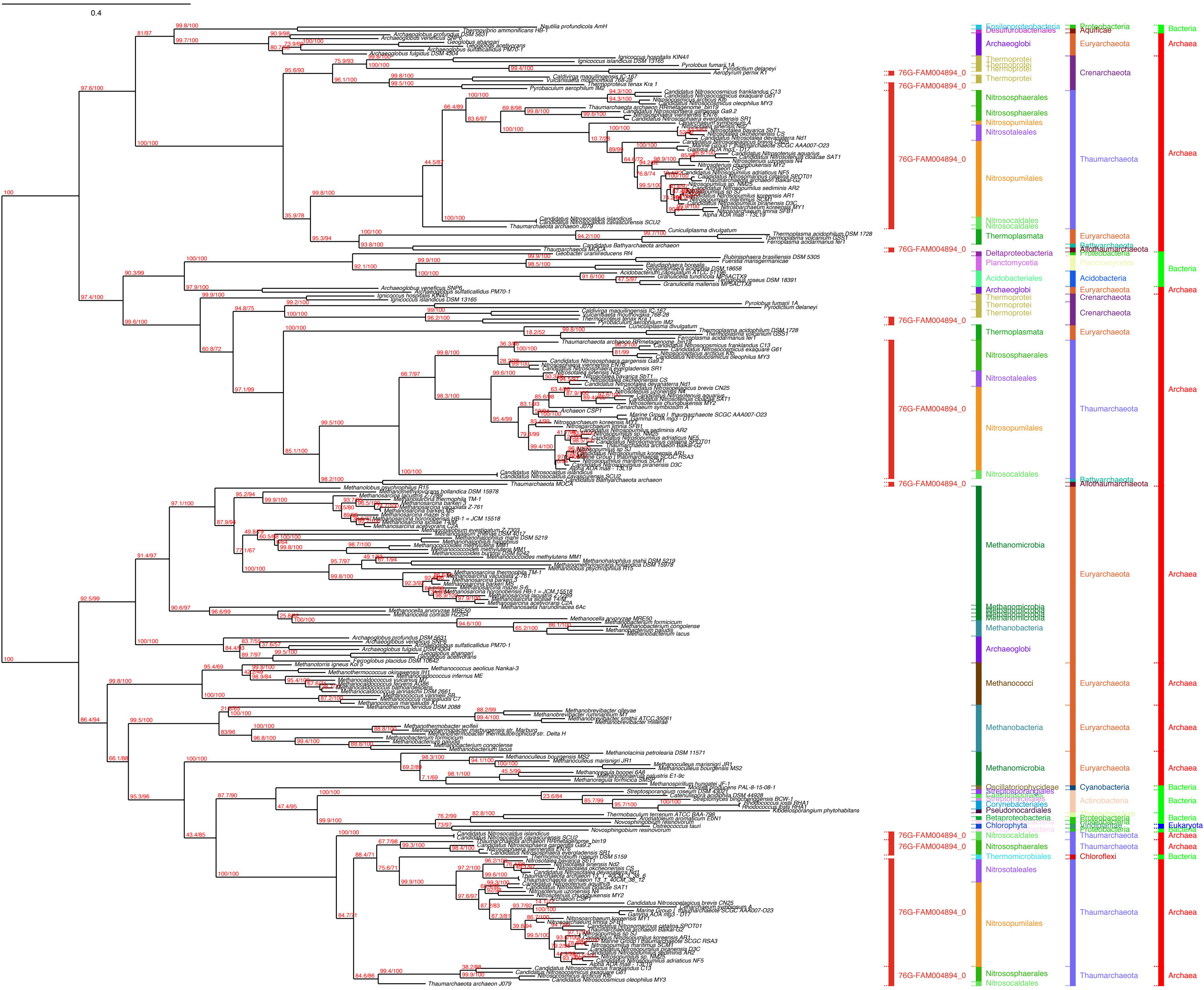
0.4
76G-FAM004894_0
Epsilonproteobacteria
Desulfurobacteriales
Archaeoglobi
Thermoprotei
Nitrososphaerales
Nitrosopumilales
Nitrosotaleales
Nitrosocaldales
Thermoplasmata
Deltaproteobacteria
Planctomycetia
Acidobacteriales
Methanomicrobia
Methanobacteria
Methanococci
Oscillatoriophycideae
Streptosporangiales
Corynebacteriales
Pseudonocardiales
Betaproteobacteria
Chlorophyta
Thermomicrobiales
Proteobacteria
Aquificae
Euryarchaeota
Crenarchaeota
Thaumarchaeota
Bathyarchaeota
Allothaumarchaeota
Planctomycetes
Acidobacteria
Cyanobacteria
Actinobacteria
Viridiplantae
Chloroflexi
Bacteria
Archaea
Eukaryota

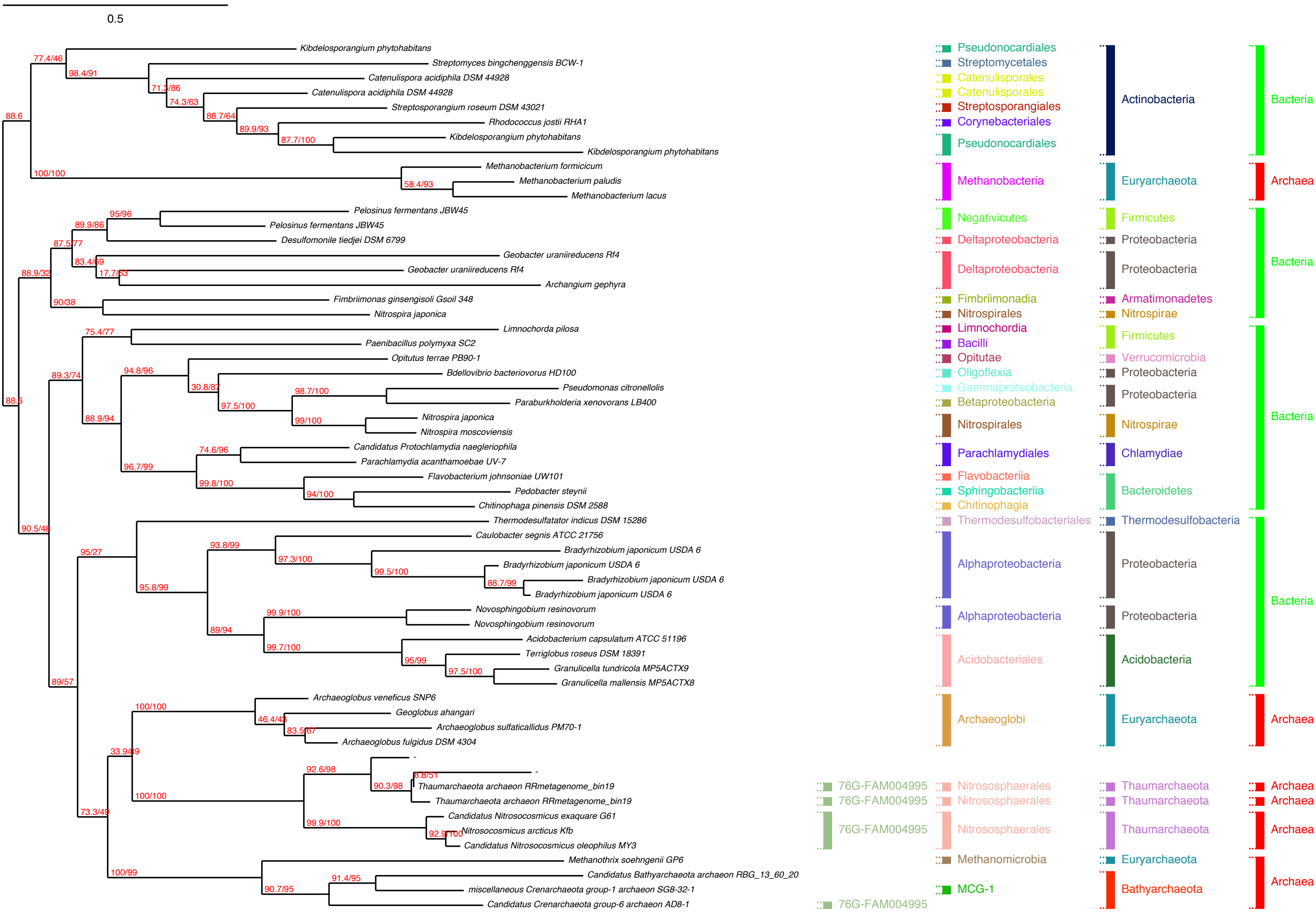

0.5
Kibdelosporangium phytohabitans
Streptomyces bingchenggensis BCW-1
Catenulispora acidiphila DSM 44928
Catenulispora acidiphila DSM 44928
Streptosporangium roseum DSM 43021
Rhodococcus jostii RHA1
Kibdelosporangium phytohabitans
Kibdelosporangium phytohabitans
Methanobacterium formicicum
Methanobacterium paludis
Methanobacterium lacus
Pelosinus fermentans JBW45
Pelosinus fermentans JBW45
Desulfomonile tiedjei DSM 6799
Geobacter uraniireducens Rf4
Geobacter uraniireducens Rf4
Archangium gephyra
Fimbriimonas ginsengisoli Gsoil 348
Nitrospira japonica
Limnochorda pilosa
Paenibacillus polymyxa SC2
Opitutus terrae PB90-1
Bdellovibrio bacteriovorus HD100
Pseudomonas citronellolis
Paraburkholderia xenovorans LB400
Nitrospira japonica
Nitrospira moscoviensis
Candidatus Protochlamydia naegleriophila
Parachlamydia acanthamoebae UV-7
Flavobacterium johnsoniae UW101
Pedobacter steynii
Chitinophaga pinensis DSM 2588
Thermodesulfatator indicus DSM 15286
Caulobacter segnis ATCC 21756
Bradyrhizobium japonicum USDA 6
Bradyrhizobium japonicum USDA 6
Bradyrhizobium japonicum USDA 6
Bradyrhizobium japonicum USDA 6
Novosphingobium resinovorum
Novosphingobium resinovorum
Acidobacterium capsulatum ATCC 51196
Terriglobus roseus DSM 18391
Granulicella tundricola MP5ACTX9
Granulicella mallensis MP5ACTX8
Archaeoglobus veneficus SNP6
Geoglobus ahangari
Archaeoglobus sulfaticallidus PM70-1
Archaeoglobus fulgidus DSM 4304
Thaumarchaeota archaeon RRmetagenome_bin19
Thaumarchaeota archaeon RRmetagenome_bin19
Candidatus Nitrosocosmicus exaquare G61
Nitrosocosmicus arcticus Kfb
Candidatus Nitrosocosmicus oleophilus MY3
Methanothrix soehngenii GP6
Candidatus Bathyarchaeota archaeon RBG_13_60_20
miscellaneous Crenarchaeota group-1 archaeon SG8-32-1
Candidatus Crenarchaeota group-6 archaeon AD8-1
Pseudonocardiales
Streptomycetales
Catenulisporales
Catenulisporales
Streptosporangiales
Corynebacteriales
Pseudonocardiales
Methanobacteria
Negativicutes
Deltaproteobacteria
Deltaproteobacteria
Fimbriimonadia
Nitrospirales
Limnochordia
Bacilli
Opitutae
Oligoflexia
Gammaproteobacteria
Betaproteobacteria
Nitrospirales
Parachlamydiales
Flavobacteriia
Sphingobacteriia
Chitinophagia
Thermodesulfobacteriales
Alphaproteobacteria
Alphaproteobacteria
Acidobacteriales
Archaeoglobi
76G-FAM004995
76G-FAM004995
76G-FAM004995
76G-FAM004995
Nitrososphaerales
Nitrososphaerales
Nitrososphaerales
Methanomicrobia
MCG-1
Actinobacteria
Euryarchaeota
Firmicutes
Proteobacteria
Proteobacteria
Armatimonadetes
Nitrospirae
Firmicutes
Verrucomicrobia
Proteobacteria
Proteobacteria
Nitrospirae
Chlamydiae
Bacteroidetes
Thermodesulfobacteria
Proteobacteria
Proteobacteria
Acidobacteria
Euryarchaeota
Thaumarchaeota
Thaumarchaeota
Thaumarchaeota
Euryarchaeota
Bathyarchaeota
Bacteria
Archaea
Bacteria
Bacteria
Bacteria
Archaea
Archaea
Archaea
Archaea
Archaea

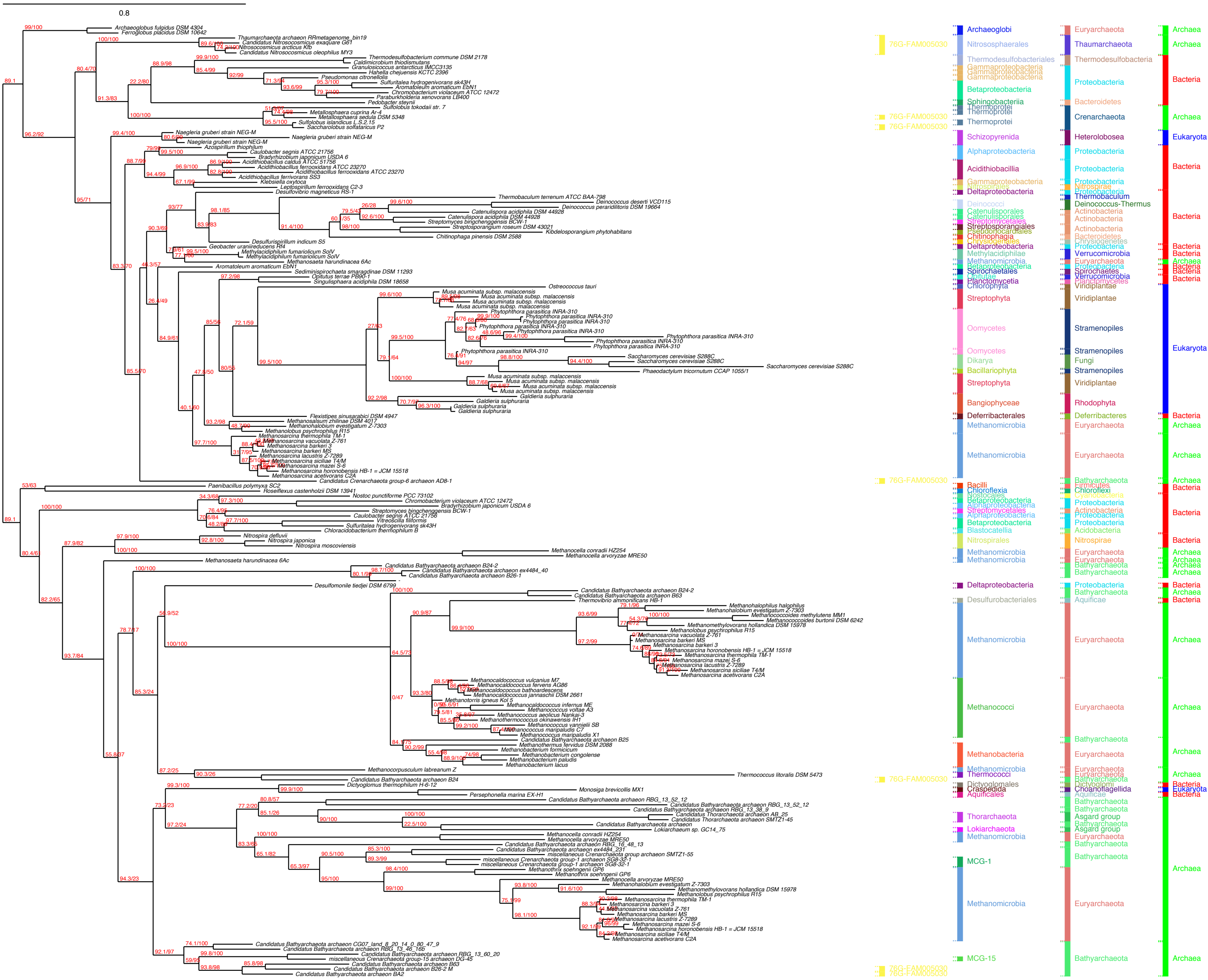

0.8
76G-FAM005030
Archaeoglobi
Nitrososphaerales
Thermodesulfobacteriales
Gammaproteobacteria
Betaproteobacteria
Sphingobacteriia
Thermoprotei
Schizopyrenida
Alphaproteobacteria
Acidithiobacillia
Deltaproteobacteria
Streptophyta
Oomycetes
Dikarya
Bacillariophyta
Bangiophyceae
Deferribacterales
Methanomicrobia
Methanococci
Methanobacteria
Thermococci
Thorarchaeota
Lokiarchaeota
MCG-1
MCG-15
Euryarchaeota
Thaumarchaeota
Thermodesulfobacteria
Proteobacteria
Bacteroidetes
Crenarchaeota
Heterolobosea
Viridiplantae
Stramenopiles
Fungi
Rhodophyta
Deferribacteres
Bathyarchaeota
Asgard group
Archaea
Bacteria
Eukaryota

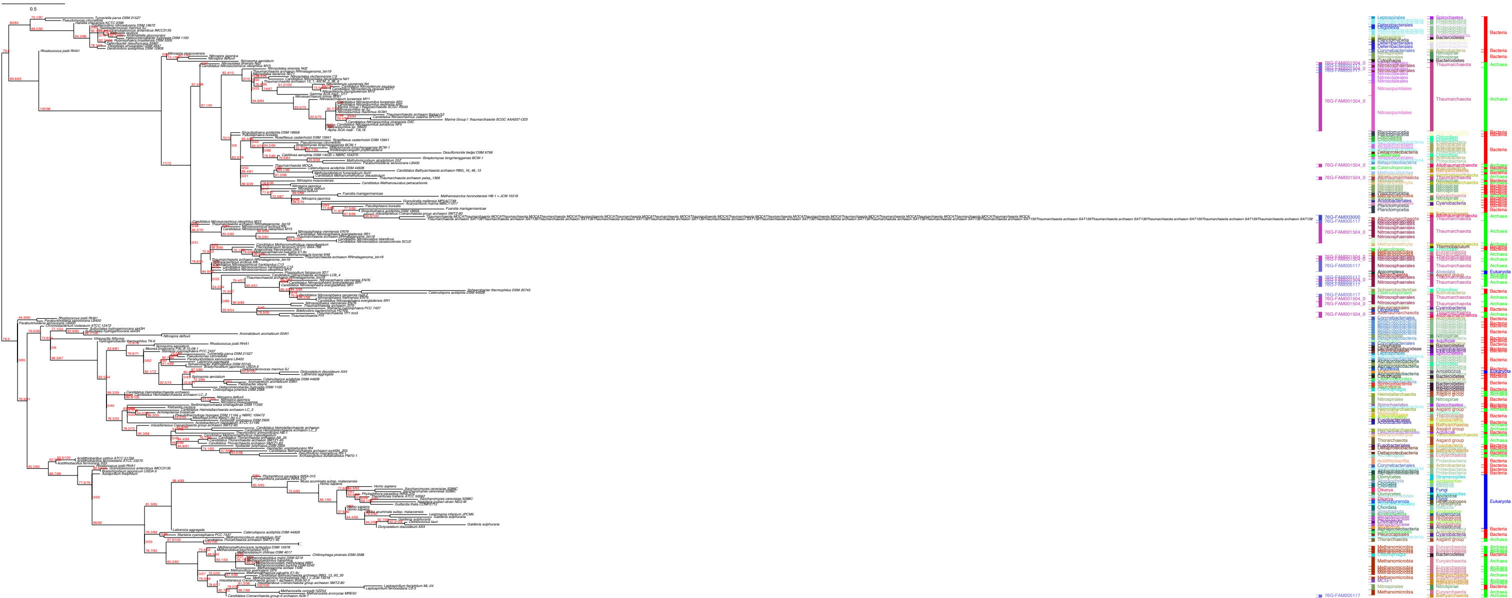

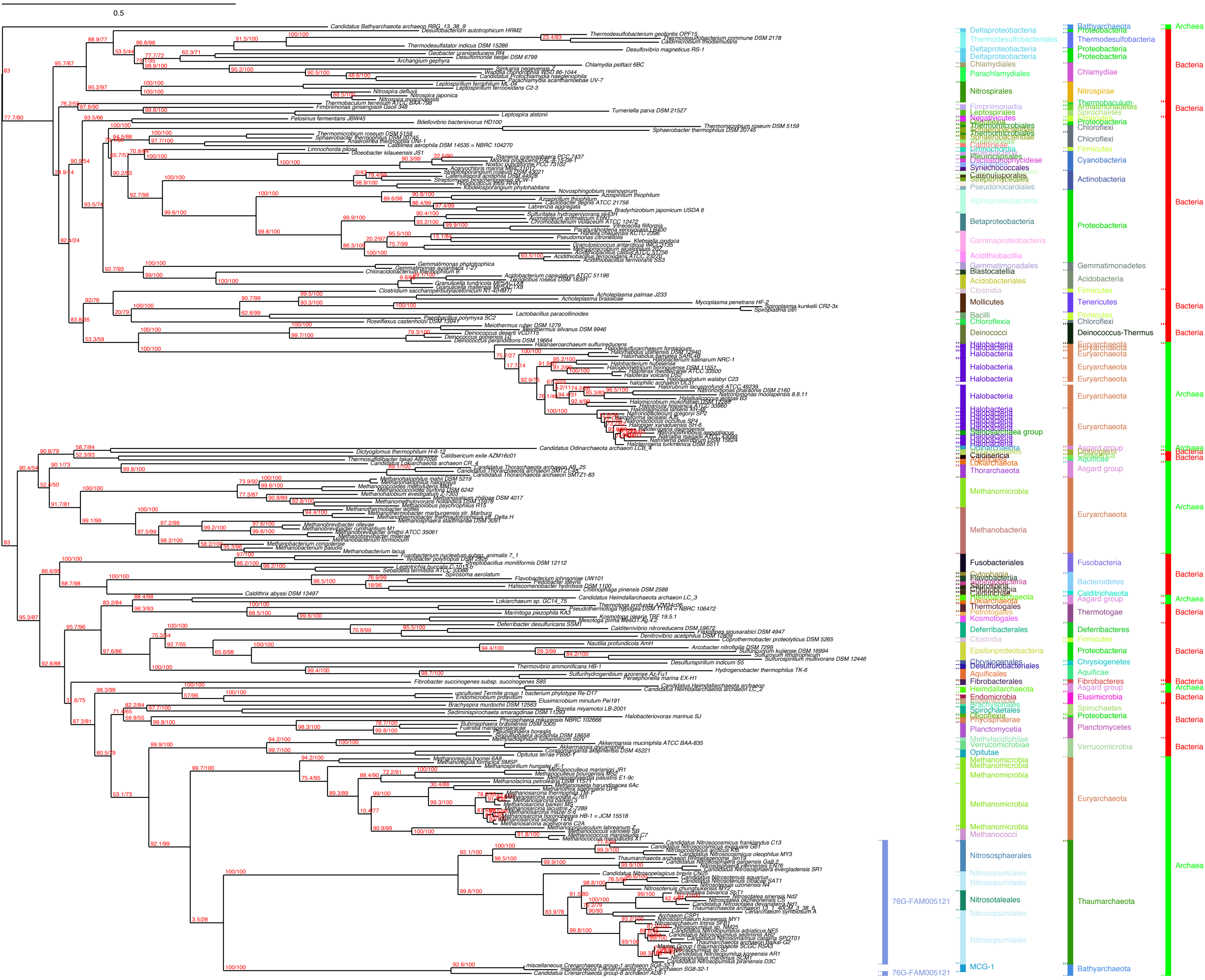

0.5
76G-FAM005121
76G-FAM005121
Bathyarchaeota
Proteobacteria
Thermodesulfobacteria
Proteobacteria
Proteobacteria
Chlamydiae
Nitrospirae
Thermobaculum
Armatimonadetes
Spirochaetes
Firmicutes
Proteobacteria
Chloroflexi
Chloroflexi
Firmicutes
Cyanobacteria
Actinobacteria
Proteobacteria
Gemmatimonadetes
Acidobacteria
Firmicutes
Tenericutes
Firmicutes
Chloroflexi
Deinococcus-Thermus
Euryarchaeota
Euryarchaeota
Euryarchaeota
Euryarchaeota
Euryarchaeota
Euryarchaeota
Asgard group
Dictyoglomi
Aquificae
Asgard group
Euryarchaeota
Fusobacteria
Bacteroidetes
Calditrichaeota
Asgard group
Thermotogae
Deferribacteres
Firmicutes
Proteobacteria
Chrysiogenetes
Aquificae
Fibrobacteres
Asgard group
Elusimicrobia
Spirochaetes
Proteobacteria
Planctomycetes
Verrucomicrobia
Euryarchaeota
Thaumarchaeota
Bathyarchaeota
Archaea
Bacteria
Bacteria
Bacteria
Bacteria
Bacteria
Archaea
Archaea
Bacteria
Bacteria
Archaea
Bacteria
Archaea
Bacteria
Bacteria
Archaea
Bacteria
Bacteria
Bacteria
Archaea

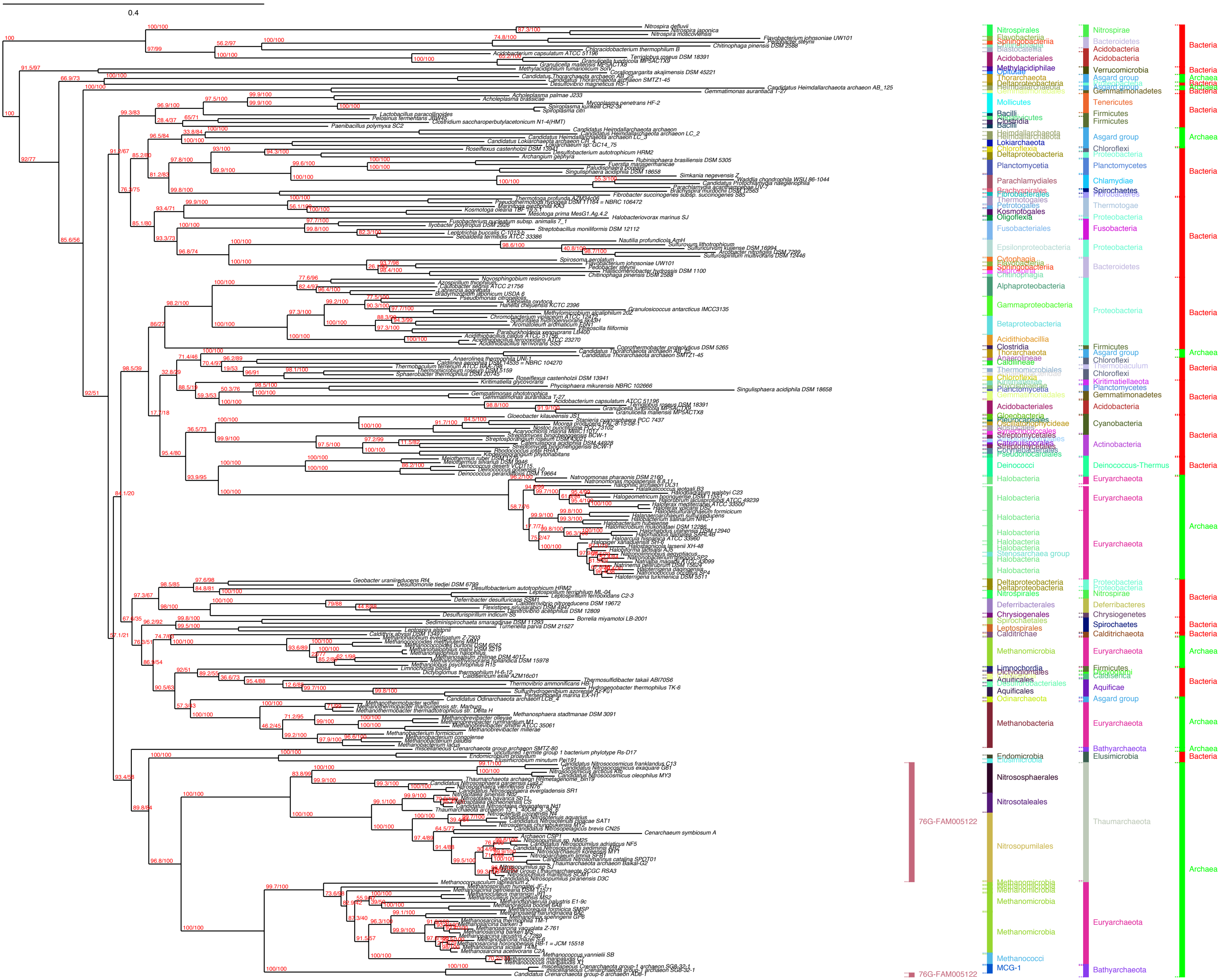
0.4
Nitrospirae
Acidobacteriales
Methylacidiphilae
Thorarchaeota
Deltaproteobacteria
Mollicutes
Bacilli
Clostridia
Heimdallarchaeota
Lokiarchaeota
Chloroflexia
Planctomycetia
Parachlamydiales
Kosmotogales
Oligoflexia
Fusobacteriales
Epsilonproteobacteria
Cytophagia
Alphaproteobacteria
Gammaproteobacteria
Betaproteobacteria
Acidithiobacillia
Anaerolineae
Caldilineae
Thermomicrobiales
Planctomycetia
Acidobacteriales
Deinococci
Halobacteria
Stenosarchaea group
Deferribacterales
Chrysiogenales
Leptospirales
Caldithrichae
Methanomicrobia
Aquificales
Odinarchaeota
Methanobacteria
Endomicrobia
Nitrososphaerales
Nitrosotaleales
Nitrosopumilales
Methanococci
MCG-1
76G-FAM005122
Nitrospirae
Bacteroidetes
Acidobacteria
Verrucomicrobia
Asgard group
Gemmatimonadetes
Tenericutes
Firmicutes
Chloroflexi
Proteobacteria
Planctomycetes
Chlamydiae
Spirochaetes
Thermotogae
Fusobacteria
Kiritimatiellaeota
Cyanobacteria
Actinobacteria
Deinococcus-Thermus
Euryarchaeota
Deferribacteres
Chrysiogenetes
Caldithrichaeota
Aquificae
Bathyarchaeota
Elusimicrobia
Thaumarchaeota
Bacteria
Archaea

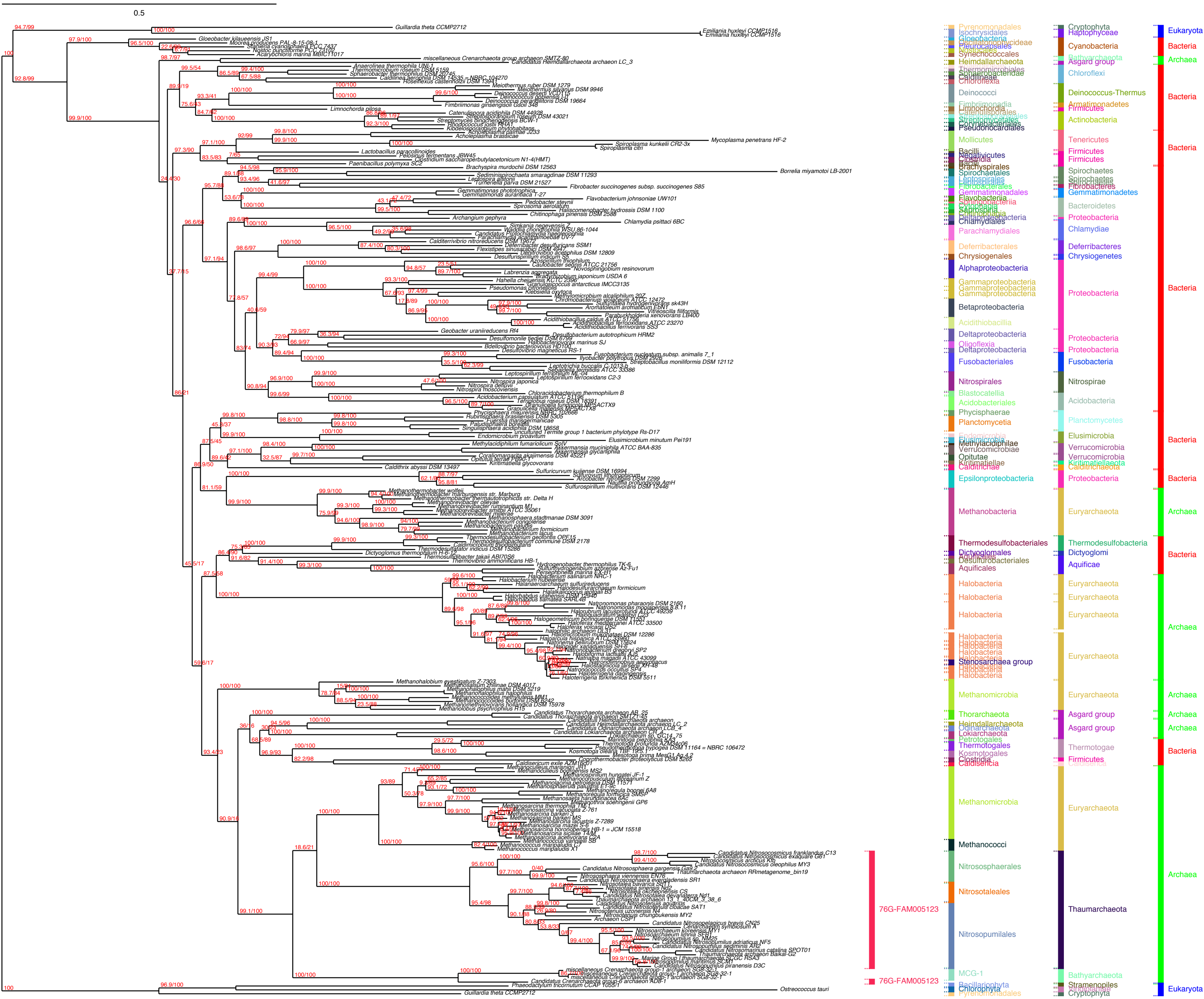
0.5
Eukaryota
Bacteria
Archaea
Cyanobacteria
Chloroflexi
Deinococcus-Thermus
Actinobacteria
Tenericutes
Firmicutes
Spirochaetes
Gemmatimonadetes
Bacteroidetes
Proteobacteria
Chlamydiae
Deferribacteres
Chrysiogenetes
Fusobacteria
Nitrospirae
Acidobacteria
Planctomycetes
Elusimicrobia
Verrucomicrobia
Euryarchaeota
Thermodesulfobacteria
Dictyoglomi
Aquificae
Asgard group
Thermotogae
Thaumarchaeota
Bathyarchaeota
Cryptophyta
76G-FAM005123
Methanobacteria
Halobacteria
Methanomicrobia
Methanococci
Nitrososphaerales
Nitrosotaleales
Nitrosopumilales
MCG-1
Alphaproteobacteria
Betaproteobacteria
Gammaproteobacteria
Deltaproteobacteria
Epsilonproteobacteria
Mollicutes
Deinococci
Thorarchaeota
Lokiarchaeota
Heimdallarchaeota

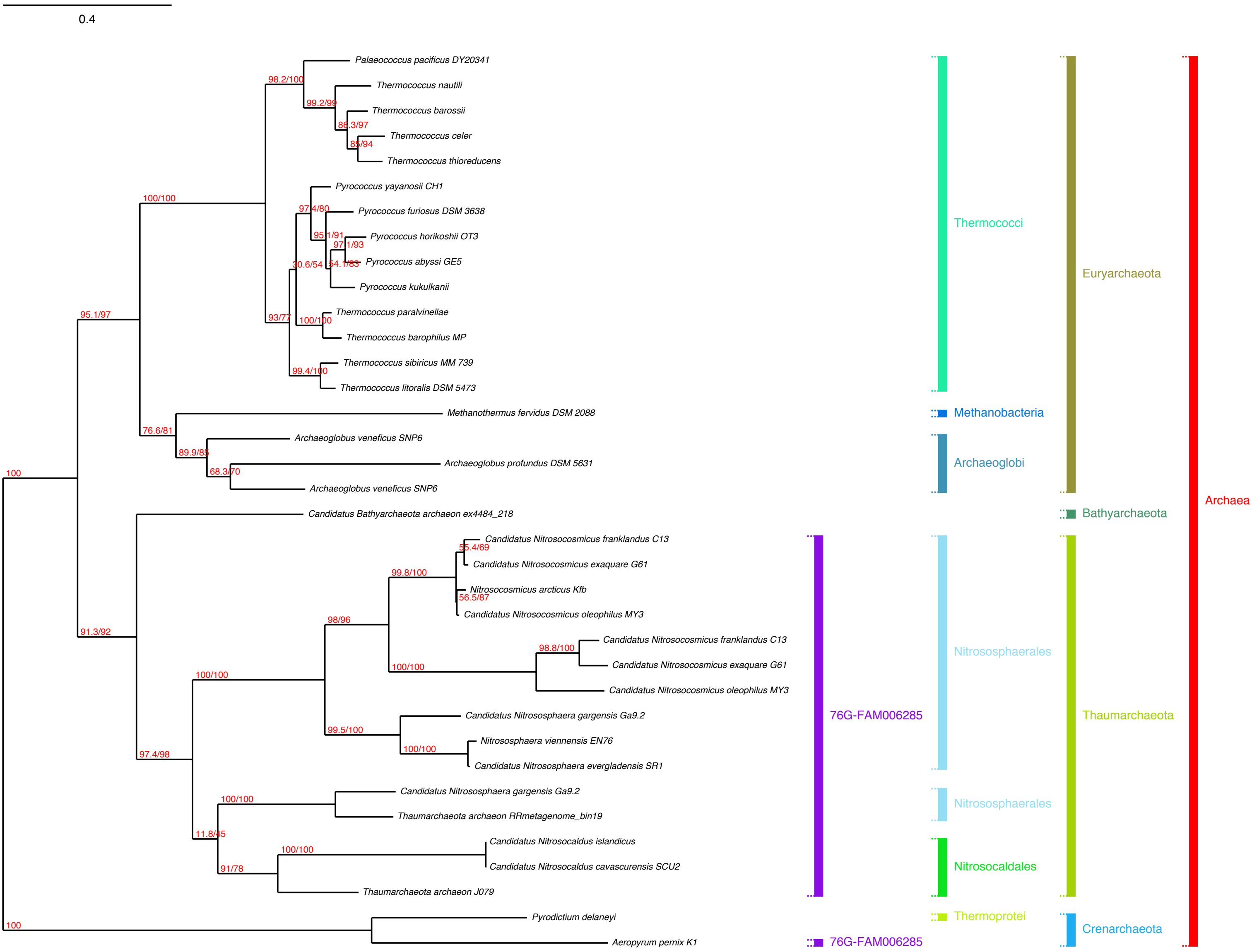

0.4
Palaeococcus pacificus DY20341
Thermococcus nautili
Thermococcus barossii
Thermococcus celer
Thermococcus thioreducens
Pyrococcus yayanosii CH1
Pyrococcus furiosus DSM 3638
Pyrococcus horikoshii OT3
Pyrococcus abyssi GE5
Pyrococcus kukulkanii
Thermococcus paralvinellae
Thermococcus barophilus MP
Thermococcus sibiricus MM 739
Thermococcus litoralis DSM 5473
Methanothermus fervidus DSM 2088
Archaeoglobus veneficus SNP6
Archaeoglobus profundus DSM 5631
Archaeoglobus veneficus SNP6
Candidatus Bathyarchaeota archaeon ex4484_218
Candidatus Nitrosocosmicus franklandus C13
Candidatus Nitrosocosmicus exaquare G61
Nitrosocosmicus arcticus Kfb
Candidatus Nitrosocosmicus oleophilus MY3
Candidatus Nitrosocosmicus franklandus C13
Candidatus Nitrosocosmicus exaquare G61
Candidatus Nitrosocosmicus oleophilus MY3
Candidatus Nitrososphaera gargensis Ga9.2
Nitrososphaera viennensis EN76
Candidatus Nitrososphaera evergladensis SR1
Candidatus Nitrososphaera gargensis Ga9.2
Thaumarchaeota archaeon RRmetagenome_bin19
Candidatus Nitrosocaldus islandicus
Candidatus Nitrosocaldus cavascurensis SCU2
Thaumarchaeota archaeon J079
Pyrodictium delaneyi
Aeropyrum pernix K1
98.2/100
99.2/99
100/100
95.1/97
93/77
100/100
99.4/100
76.6/81
89.9/85
68.3/70
100
55.4/69
99.8/100
56.5/87
98/96
98.8/100
100/100
100/100
91.3/92
99.5/100
100/100
97.4/98
100/100
11.8/45
100/100
91/78
100
Thermococci
Methanobacteria
Archaeoglobi
Euryarchaeota
Bathyarchaeota
Nitrososphaerales
Nitrososphaerales
Nitrosocaldales
Thermoprotei
76G-FAM006285
76G-FAM006285
Thaumarchaeota
Crenarchaeota
Archaea

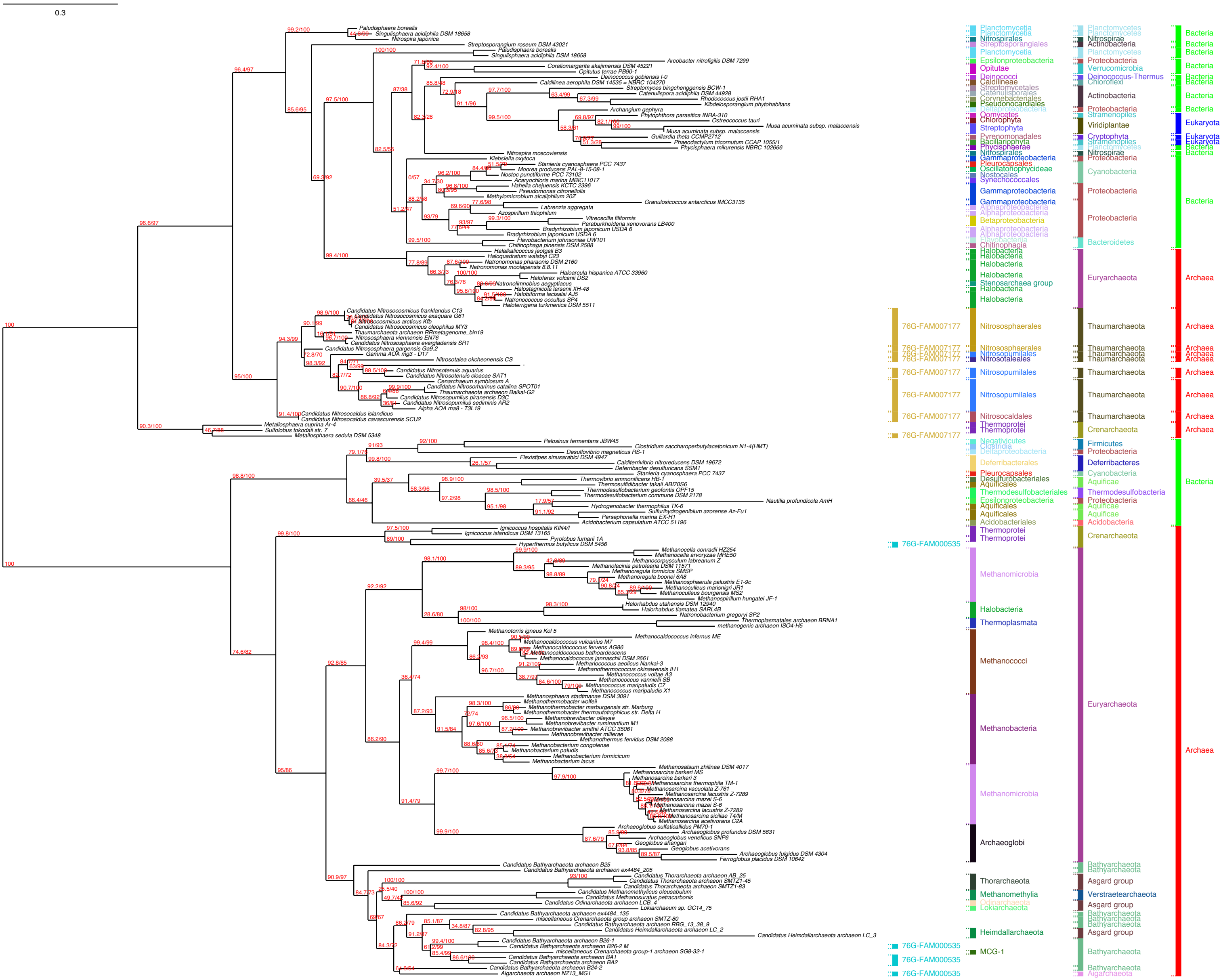

0.3
Paludisphaera borealis
Singulisphaera acidiphila DSM 18658
Nitrospira japonica
Streptosporangium roseum DSM 43021
Paludisphaera borealis
Singulisphaera acidiphila DSM 18658
Arcobacter nitrofigilis DSM 7299
Coraliomargarita akajimensis DSM 45221
Opitutus terrae PB90-1
Deinococcus gobiensis I-0
Caldilinea aerophila DSM 14535 = NBRC 104270
Streptomyces bingchenggensis BCW-1
Catenulispora acidiphila DSM 44928
Rhodococcus jostii RHA1
Kibdelosporangium phytohabitans
Archangium gephyra
Phytophthora parasitica INRA-310
Ostreococcus tauri
Musa acuminata subsp. malaccensis
Musa acuminata subsp. malaccensis
Guillardia theta CCMP2712
Phaeodactylum tricornutum CCAP 1055/1
Phycisphaera mikurensis NBRC 102666
Nitrospira moscoviensis
Klebsiella oxytoca
Stanieria cyanosphaera PCC 7437
Moorea producens PAL-8-15-08-1
Nostoc punctiforme PCC 73102
Acaryochloris marina MBIC11017
Hahella chejuensis KCTC 2396
Pseudomonas citronellolis
Methylomicrobium alcaliphilum 20Z
Granulosicoccus antarcticus IMCC3135
Labrenzia aggregata
Azospirillum thiophilum
Vitreoscilla filiformis
Paraburkholderia xenovorans LB400
Bradyrhizobium japonicum USDA 6
Bradyrhizobium japonicum USDA 6
Flavobacterium johnsoniae UW101
Chitinophaga pinensis DSM 2588
Halalkalicoccus jeotgali B3
Haloquadratum walsbyi C23
Natronomonas pharaonis DSM 2160
Natronomonas moolapensis 8.8.11
Haloarcula hispanica ATCC 33960
Haloferax volcanii DS2
Natronolimnobius aegyptiacus
Halostagnicola larsenii XH-48
Halobiforma lacisalsi AJ5
Natronococcus occultus SP4
Haloterrigena turkmenica DSM 5511
Candidatus Nitrosocosmicus franklandus C13
Candidatus Nitrosocosmicus exaquare G61
Nitrosocosmicus arcticus Kfb
Candidatus Nitrosocosmicus oleophilus MY3
Thaumarchaeota archaeon RRmetagenome_bin19
Nitrososphaera viennensis EN76
Candidatus Nitrososphaera evergladensis SR1
Candidatus Nitrososphaera gargensis Ga9.2
Gamma AOA mg3 - D17
Nitrosotalea okcheonensis CS
Candidatus Nitrosotenuis aquarius
Candidatus Nitrosotenuis cloacae SAT1
Cenarchaeum symbiosum A
Candidatus Nitrosomarinus catalina SPOT01
Thaumarchaeota archaeon Baikal-G2
Candidatus Nitrosopumilus piranensis D3C
Candidatus Nitrosopumilus sediminis AR2
Alpha AOA ma8 - T3L19
Candidatus Nitrosocaldus islandicus
Candidatus Nitrosocaldus cavascurensis SCU2
Metallosphaera cuprina Ar-4
Sulfolobus tokodaii str. 7
Metallosphaera sedula DSM 5348
Pelosinus fermentans JBW45
Clostridium saccharoperbutylacetonicum N1-4(HMT)
Desulfovibrio magneticus RS-1
Flexistipes sinusarabici DSM 4947
Calditerrivibrio nitroreducens DSM 19672
Deferribacter desulfuricans SSM1
Stanieria cyanosphaera PCC 7437
Thermovibrio ammonificans HB-1
Thermosulfidibacter takaii ABI70S6
Thermodesulfobacterium geofontis OPF15
Thermodesulfobacterium commune DSM 2178
Nautilia profundicola AmH
Hydrogenobacter thermophilus TK-6
Sulfurihydrogenibium azorense Az-Fu1
Persephonella marina EX-H1
Acidobacterium capsulatum ATCC 51196
Ignicoccus hospitalis KIN4/I
Ignicoccus islandicus DSM 13165
Pyrolobus fumarii 1A
Hyperthermus butylicus DSM 5456
Methanocella conradii HZ254
Methanocella arvoryzae MRE50
Methanocorpusculum labreanum Z
Methanolacinia petrolearia DSM 11571
Methanoregula formicica SMSP
Methanoregula boonei 6A8
Methanosphaerula palustris E1-9c
Methanoculleus marisnigri JR1
Methanoculleus bourgensis MS2
Methanospirillum hungatei JF-1
Halorhabdus utahensis DSM 12940
Halorhabdus tiamatea SARL4B
Natronobacterium gregoryi SP2
Thermoplasmatales archaeon BRNA1
methanogenic archaeon ISO4-H5
Methanotorris igneus Kol 5
Methanocaldococcus infernus ME
Methanocaldococcus vulcanius M7
Methanocaldococcus fervens AG86
Methanocaldococcus bathoardescens
Methanocaldococcus jannaschii DSM 2661
Methanococcus aeolicus Nankai-3
Methanothermococcus okinawensis IH1
Methanococcus voltae A3
Methanococcus vannielii SB
Methanococcus maripaludis C7
Methanococcus maripaludis X1
Methanosphaera stadtmanae DSM 3091
Methanothermobacter wolfeii
Methanothermobacter marburgensis str. Marburg
Methanothermobacter thermautotrophicus str. Delta H
Methanobrevibacter olleyae
Methanobrevibacter ruminantium M1
Methanobrevibacter smithii ATCC 35061
Methanobrevibacter millerae
Methanothermus fervidus DSM 2088
Methanobacterium congolense
Methanobacterium paludis
Methanobacterium formicicum
Methanobacterium lacus
Methanosalsum zhilinae DSM 4017
Methanosarcina barkeri MS
Methanosarcina barkeri 3
Methanosarcina thermophila TM-1
Methanosarcina vacuolata Z-761
Methanosarcina lacustris Z-7289
Methanosarcina mazei S-6
Methanosarcina mazei S-6
Methanosarcina lacustris Z-7289
Methanosarcina siciliae T4/M
Methanosarcina acetivorans C2A
Archaeoglobus sulfaticallidus PM70-1
Archaeoglobus profundus DSM 5631
Archaeoglobus veneficus SNP6
Geoglobus ahangari
Geoglobus acetivorans
Archaeoglobus fulgidus DSM 4304
Ferroglobus placidus DSM 10642
Candidatus Bathyarchaeota archaeon B25
Candidatus Bathyarchaeota archaeon ex4484_205
Candidatus Thorarchaeota archaeon AB_25
Candidatus Thorarchaeota archaeon SMTZ1-45
Candidatus Thorarchaeota archaeon SMTZ1-83
Candidatus Methanomethylicus oleusabulum
Candidatus Methanosuratus petracarbonis
Candidatus Odinarchaeota archaeon LCB_4
Lokiarchaeum sp. GC14_75
Candidatus Bathyarchaeota archaeon ex4484_135
miscellaneous Crenarchaeota group archaeon SMTZ-80
Candidatus Bathyarchaeota archaeon RBG_13_38_9
Candidatus Heimdallarchaeota archaeon LC_2
Candidatus Heimdallarchaeota archaeon LC_3
Candidatus Bathyarchaeota archaeon B26-1
Candidatus Bathyarchaeota archaeon B26-2 M
miscellaneous Crenarchaeota group-1 archaeon SG8-32-1
Candidatus Bathyarchaeota archaeon BA1
Candidatus Bathyarchaeota archaeon BA2
Candidatus Bathyarchaeota archaeon B24-2
Aigarchaeota archaeon NZ13_MG1
76G-FAM007177
76G-FAM000535
Planctomycetia
Nitrospirales
Streptosporangiales
Epsilonproteobacteria
Opitutae
Deinococci
Caldilineae
Streptomycetales
Catenulisporales
Corynebacteriales
Pseudonocardiales
Deltaproteobacteria
Oomycetes
Chlorophyta
Streptophyta
Pyrenomonadales
Bacillariophyta
Phycisphaerae
Gammaproteobacteria
Pleurocapsales
Oscillatoriophycideae
Nostocales
Synechococcales
Alphaproteobacteria
Betaproteobacteria
Flavobacteriia
Chitinophagia
Halobacteria
Stenosarchaea group
Nitrososphaerales
Nitrosopumilales
Nitrosotaleales
Nitrosocaldales
Thermoprotei
Negativicutes
Clostridia
Deferribacterales
Desulfurobacteriales
Aquificales
Thermodesulfobacteriales
Acidobacteriales
Methanomicrobia
Thermoplasmata
Methanococci
Methanobacteria
Archaeoglobi
Thorarchaeota
Methanomethylia
Odinarchaeota
Lokiarchaeota
Heimdallarchaeota
MCG-1
Planctomycetes
Nitrospirae
Actinobacteria
Proteobacteria
Verrucomicrobia
Deinococcus-Thermus
Chloroflexi
Stramenopiles
Viridiplantae
Cryptophyta
Cyanobacteria
Bacteroidetes
Euryarchaeota
Thaumarchaeota
Crenarchaeota
Firmicutes
Deferribacteres
Aquificae
Thermodesulfobacteria
Acidobacteria
Bathyarchaeota
Asgard group
Verstraetearchaeota
Aigarchaeota
Bacteria
Eukaryota
Archaea

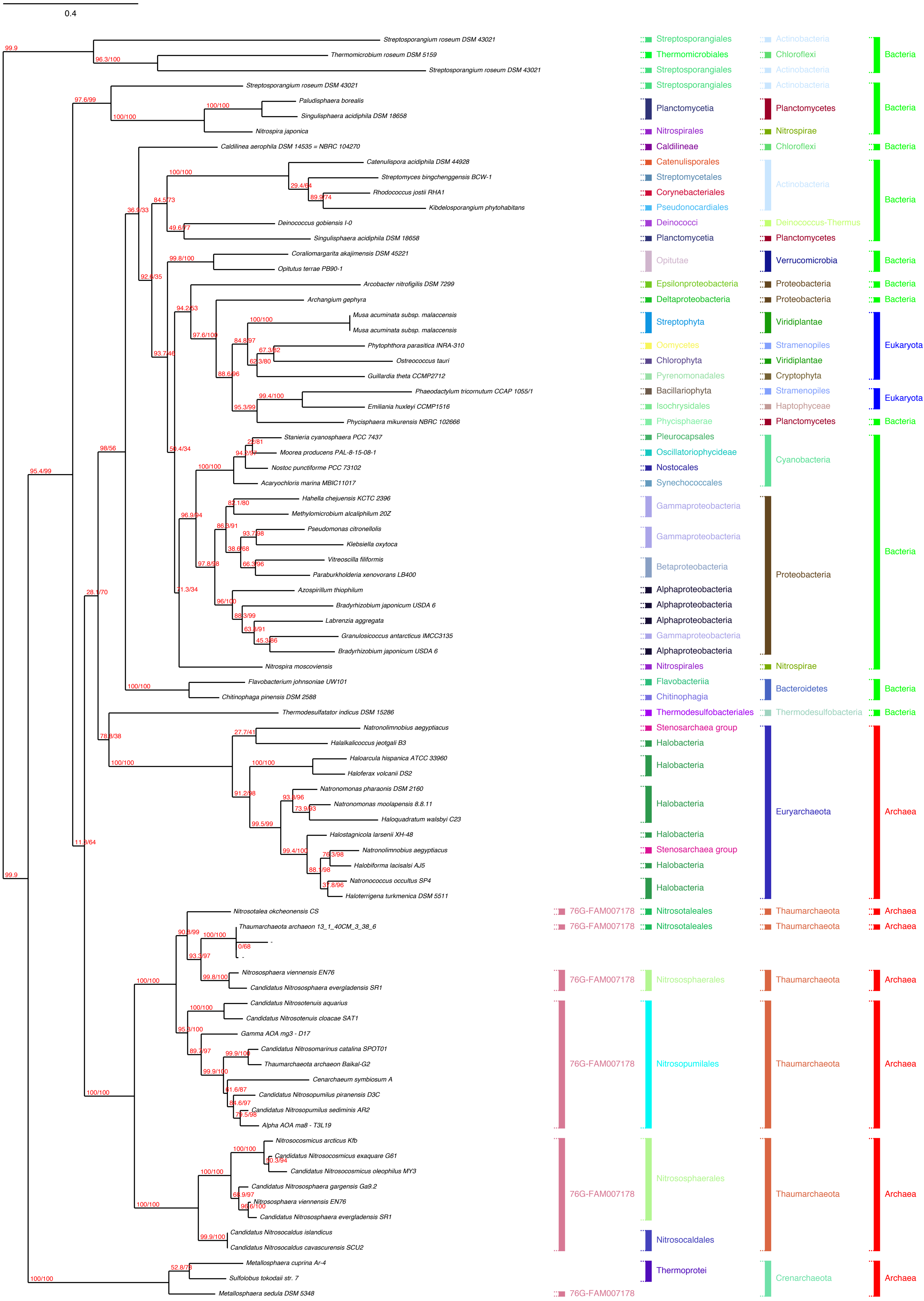
0.4
Streptosporangium roseum DSM 43021
Thermomicrobium roseum DSM 5159
Streptosporangium roseum DSM 43021
Streptosporangium roseum DSM 43021
Paludisphaera borealis
Singulisphaera acidiphila DSM 18658
Nitrospira japonica
Caldilinea aerophila DSM 14535 = NBRC 104270
Catenulispora acidiphila DSM 44928
Streptomyces bingchenggensis BCW-1
Rhodococcus jostii RHA1
Kibdelosporangium phytohabitans
Deinococcus gobiensis I-0
Singulisphaera acidiphila DSM 18658
Coraliomargarita akajimensis DSM 45221
Opitutus terrae PB90-1
Arcobacter nitrofigilis DSM 7299
Archangium gephyra
Musa acuminata subsp. malaccensis
Musa acuminata subsp. malaccensis
Phytophthora parasitica INRA-310
Ostreococcus tauri
Guillardia theta CCMP2712
Phaeodactylum tricornutum CCAP 1055/1
Emiliania huxleyi CCMP1516
Phycisphaera mikurensis NBRC 102666
Stanieria cyanosphaera PCC 7437
Moorea producens PAL-8-15-08-1
Nostoc punctiforme PCC 73102
Acaryochloris marina MBIC11017
Hahella chejuensis KCTC 2396
Methylomicrobium alcaliphilum 20Z
Pseudomonas citronellolis
Klebsiella oxytoca
Vitreoscilla filiformis
Paraburkholderia xenovorans LB400
Azospirillum thiophilum
Bradyrhizobium japonicum USDA 6
Labrenzia aggregata
Granulosicoccus antarcticus IMCC3135
Bradyrhizobium japonicum USDA 6
Nitrospira moscoviensis
Flavobacterium johnsoniae UW101
Chitinophaga pinensis DSM 2588
Thermodesulfatator indicus DSM 15286
Natronolimnobius aegyptiacus
Halalkalicoccus jeotgali B3
Haloarcula hispanica ATCC 33960
Haloferax volcanii DS2
Natronomonas pharaonis DSM 2160
Natronomonas moolapensis 8.8.11
Haloquadratum walsbyi C23
Halostagnicola larsenii XH-48
Natronolimnobius aegyptiacus
Halobiforma lacisalsi AJ5
Natronococcus occultus SP4
Haloterrigena turkmenica DSM 5511
Nitrosotalea okcheonensis CS
Thaumarchaeota archaeon 13_1_40CM_3_38_6
Nitrososphaera viennensis EN76
Candidatus Nitrososphaera evergladensis SR1
Candidatus Nitrosotenuis aquarius
Candidatus Nitrosotenuis cloacae SAT1
Gamma AOA mg3 - D17
Candidatus Nitrosomarinus catalina SPOT01
Thaumarchaeota archaeon Baikal-G2
Cenarchaeum symbiosum A
Candidatus Nitrosopumilus piranensis D3C
Candidatus Nitrosopumilus sediminis AR2
Alpha AOA ma8 - T3L19
Nitrosocosmicus arcticus Kfb
Candidatus Nitrosocosmicus exaquare G61
Candidatus Nitrosocosmicus oleophilus MY3
Candidatus Nitrososphaera gargensis Ga9.2
Nitrososphaera viennensis EN76
Candidatus Nitrososphaera evergladensis SR1
Candidatus Nitrosocaldus islandicus
Candidatus Nitrosocaldus cavascurensis SCU2
Metallosphaera cuprina Ar-4
Sulfolobus tokodaii str. 7
Metallosphaera sedula DSM 5348
Streptosporangiales
Thermomicrobiales
Streptosporangiales
Streptosporangiales
Planctomycetia
Nitrospirales
Caldilineae
Catenulisporales
Streptomycetales
Corynebacteriales
Pseudonocardiales
Deinococci
Planctomycetia
Opitutae
Epsilonproteobacteria
Deltaproteobacteria
Streptophyta
Oomycetes
Chlorophyta
Pyrenomonadales
Bacillariophyta
Isochrysidales
Phycisphaerae
Pleurocapsales
Oscillatoriophycideae
Nostocales
Synechococcales
Gammaproteobacteria
Gammaproteobacteria
Betaproteobacteria
Alphaproteobacteria
Alphaproteobacteria
Alphaproteobacteria
Gammaproteobacteria
Alphaproteobacteria
Nitrospirales
Flavobacteriia
Chitinophagia
Thermodesulfobacteriales
Stenosarchaea group
Halobacteria
Halobacteria
Halobacteria
Halobacteria
Stenosarchaea group
Halobacteria
Halobacteria
76G-FAM007178
Nitrosotaleales
76G-FAM007178
Nitrosotaleales
76G-FAM007178
Nitrososphaerales
76G-FAM007178
Nitrosopumilales
76G-FAM007178
Nitrososphaerales
Nitrosocaldales
Thermoprotei
76G-FAM007178
Actinobacteria
Chloroflexi
Actinobacteria
Actinobacteria
Planctomycetes
Nitrospirae
Chloroflexi
Actinobacteria
Deinococcus-Thermus
Planctomycetes
Verrucomicrobia
Proteobacteria
Proteobacteria
Viridiplantae
Stramenopiles
Viridiplantae
Cryptophyta
Stramenopiles
Haptophyceae
Planctomycetes
Cyanobacteria
Proteobacteria
Nitrospirae
Bacteroidetes
Thermodesulfobacteria
Euryarchaeota
Thaumarchaeota
Thaumarchaeota
Thaumarchaeota
Thaumarchaeota
Thaumarchaeota
Crenarchaeota
Bacteria
Bacteria
Bacteria
Bacteria
Bacteria
Bacteria
Bacteria
Eukaryota
Eukaryota
Bacteria
Bacteria
Bacteria
Bacteria
Archaea
Archaea
Archaea
Archaea
Archaea
Archaea
Archaea

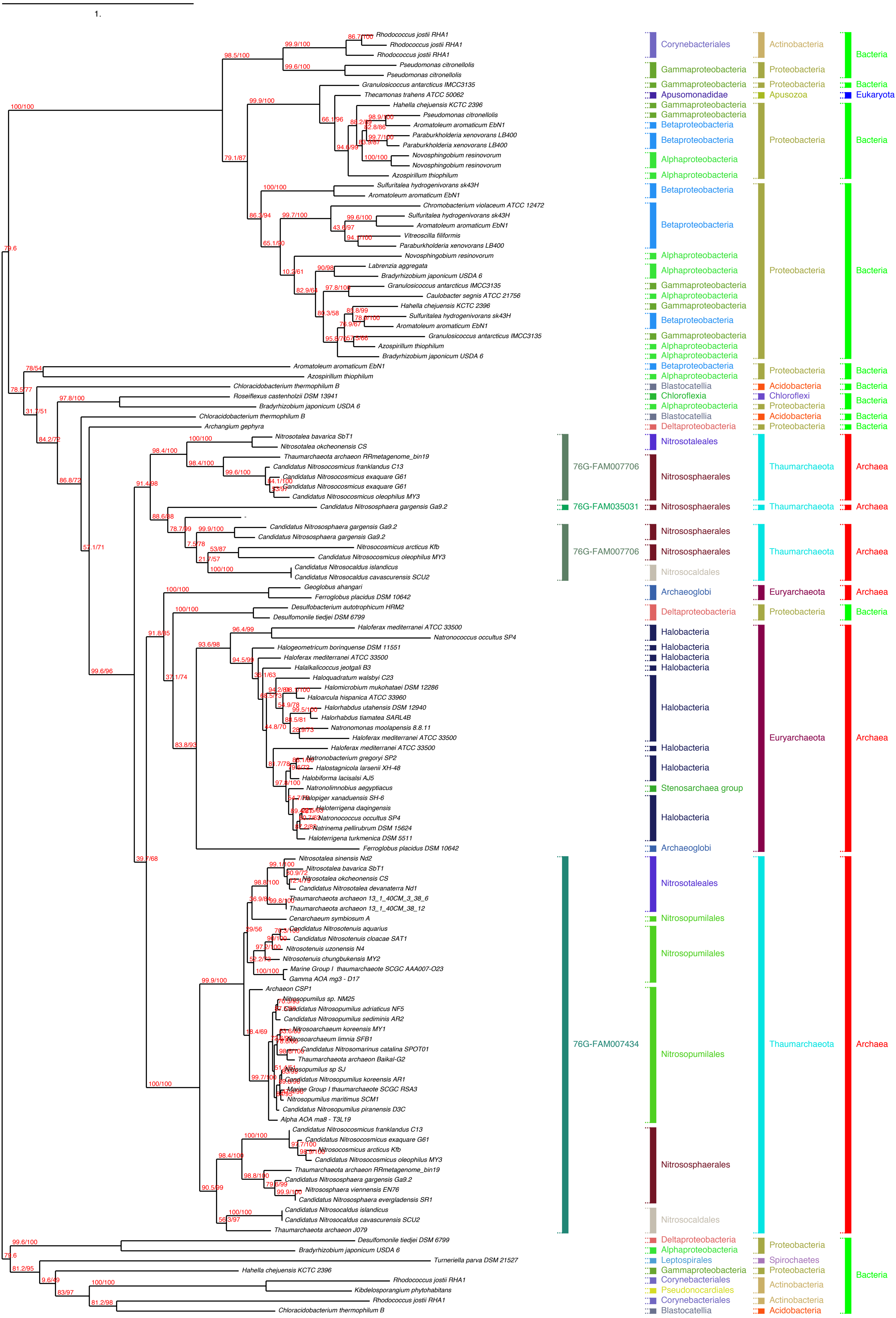

1.
Rhodococcus jostii RHA1
Pseudomonas citronellolis
Granulosicoccus antarcticus IMCC3135
Thecamonas trahens ATCC 50062
Hahella chejuensis KCTC 2396
Aromatoleum aromaticum EbN1
Paraburkholderia xenovorans LB400
Novosphingobium resinovorum
Azospirillum thiophilum
Sulfuritalea hydrogenivorans sk43H
Chromobacterium violaceum ATCC 12472
Vitreoscilla filiformis
Labrenzia aggregata
Bradyrhizobium japonicum USDA 6
Caulobacter segnis ATCC 21756
Chloracidobacterium thermophilum B
Roseiflexus castenholzii DSM 13941
Archangium gephyra
Nitrosotalea bavarica SbT1
Nitrosotalea okcheonensis CS
Thaumarchaeota archaeon RRmetagenome_bin19
Candidatus Nitrosocosmicus franklandus C13
Candidatus Nitrosocosmicus exaquare G61
Candidatus Nitrosocosmicus oleophilus MY3
Candidatus Nitrososphaera gargensis Ga9.2
Nitrosocosmicus arcticus Kfb
Candidatus Nitrosocaldus islandicus
Candidatus Nitrosocaldus cavascurensis SCU2
Geoglobus ahangari
Ferroglobus placidus DSM 10642
Desulfobacterium autotrophicum HRM2
Desulfomonile tiedjei DSM 6799
Haloferax mediterranei ATCC 33500
Natronococcus occultus SP4
Halogeometricum borinquense DSM 11551
Halalkalicoccus jeotgali B3
Haloquadratum walsbyi C23
Halomicrobium mukohataei DSM 12286
Haloarcula hispanica ATCC 33960
Halorhabdus utahensis DSM 12940
Halorhabdus tiamatea SARL4B
Natronomonas moolapensis 8.8.11
Natronobacterium gregoryi SP2
Halostagnicola larsenii XH-48
Halobiforma lacisalsi AJ5
Natronolimnobius aegyptiacus
Halopiger xanaduensis SH-6
Haloterrigena daqingensis
Natrinema pellirubrum DSM 15624
Haloterrigena turkmenica DSM 5511
Nitrosotalea sinensis Nd2
Candidatus Nitrosotalea devanaterra Nd1
Thaumarchaeota archaeon 13_1_40CM_3_38_6
Thaumarchaeota archaeon 13_1_40CM_38_12
Cenarchaeum symbiosum A
Candidatus Nitrosotenuis aquarius
Candidatus Nitrosotenuis cloacae SAT1
Nitrosotenuis uzonensis N4
Nitrosotenuis chungbukensis MY2
Marine Group I thaumarchaeote SCGC AAA007-O23
Gamma AOA mg3 - D17
Archaeon CSP1
Nitrosopumilus sp. NM25
Candidatus Nitrosopumilus adriaticus NF5
Candidatus Nitrosopumilus sediminis AR2
Nitrosoarchaeum koreensis MY1
Nitrosoarchaeum limnia SFB1
Candidatus Nitrosomarinus catalina SPOT01
Thaumarchaeota archaeon Baikal-G2
Nitrosopumilus sp SJ
Candidatus Nitrosopumilus koreensis AR1
Marine Group I thaumarchaeote SCGC RSA3
Nitrosopumilus maritimus SCM1
Candidatus Nitrosopumilus piranensis D3C
Alpha AOA ma8 - T3L19
Nitrososphaera viennensis EN76
Candidatus Nitrososphaera evergladensis SR1
Thaumarchaeota archaeon J079
Turneriella parva DSM 21527
Kibdelosporangium phytohabitans
76G-FAM007706
76G-FAM035031
76G-FAM007434
Corynebacteriales
Gammaproteobacteria
Apusomonadidae
Betaproteobacteria
Alphaproteobacteria
Blastocatellia
Chloroflexia
Deltaproteobacteria
Nitrosotaleales
Nitrososphaerales
Nitrosocaldales
Archaeoglobi
Halobacteria
Stenosarchaea group
Nitrosopumilales
Leptospirales
Pseudonocardiales
Actinobacteria
Proteobacteria
Apusozoa
Acidobacteria
Chloroflexi
Thaumarchaeota
Euryarchaeota
Spirochaetes
Bacteria
Eukaryota
Archaea

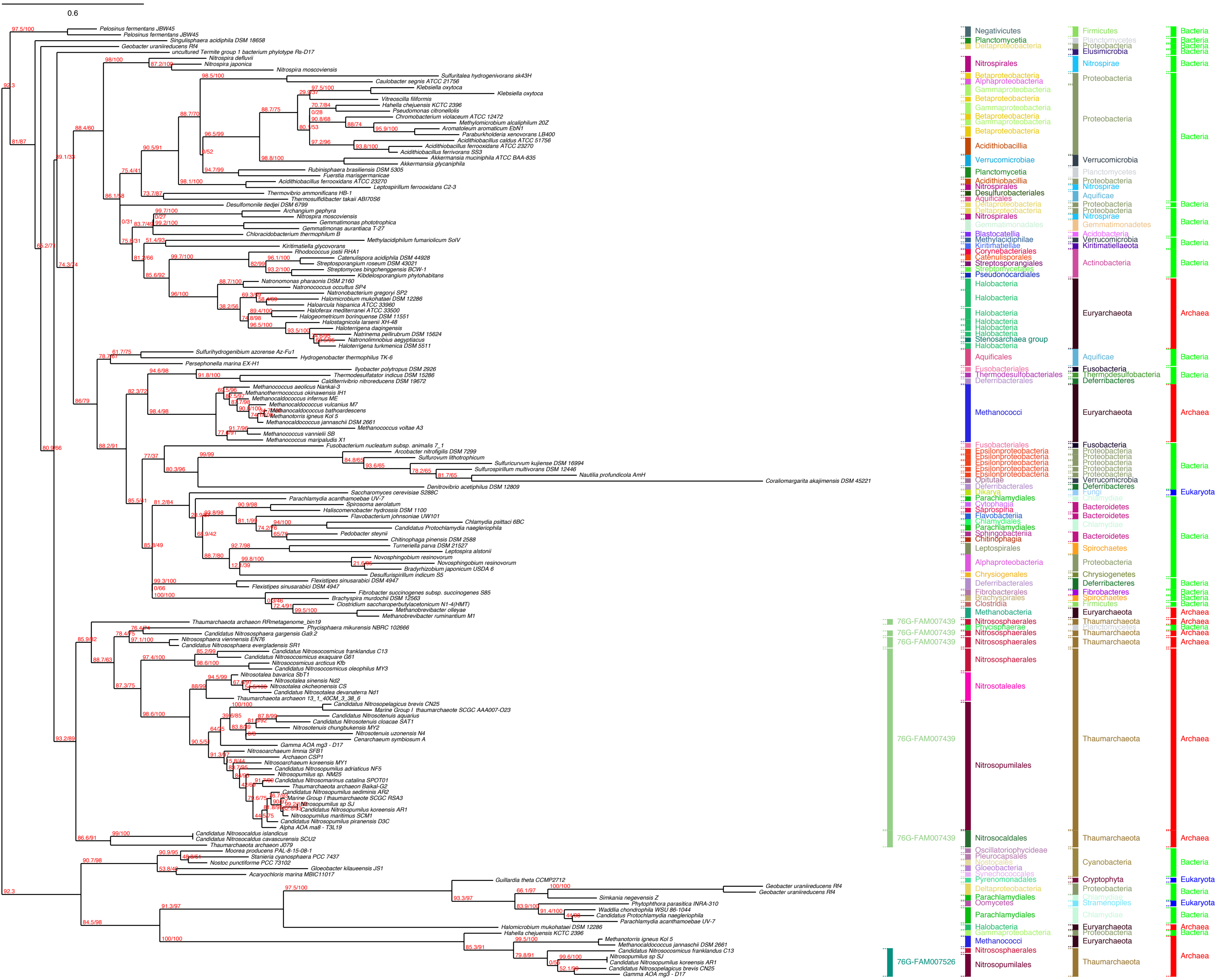

0.6
Pelosinus fermentans JBW45
Pelosinus fermentans JBW45
Singulisphaera acidiphila DSM 18658
Geobacter uraniireducens Rf4
uncultured Termite group 1 bacterium phylotype Rs-D17
Nitrospira defluvii
Nitrospira japonica
Nitrospira moscoviensis
Sulfuritalea hydrogenivorans sk43H
Caulobacter segnis ATCC 21756
Klebsiella oxytoca
Klebsiella oxytoca
Vitreoscilla filiformis
Hahella chejuensis KCTC 2396
Pseudomonas citronellolis
Chromobacterium violaceum ATCC 12472
Methylomicrobium alcaliphilum 20Z
Aromatoleum aromaticum EbN1
Paraburkholderia xenovorans LB400
Acidithiobacillus caldus ATCC 51756
Acidithiobacillus ferrooxidans ATCC 23270
Acidithiobacillus ferrivorans SS3
Akkermansia muciniphila ATCC BAA-835
Akkermansia glycaniphila
Rubinisphaera brasiliensis DSM 5305
Fuerstia marisgermanicae
Acidithiobacillus ferrooxidans ATCC 23270
Leptospirillum ferrooxidans C2-3
Thermovibrio ammonificans HB-1
Thermosulfidibacter takaii ABI70S6
Desulfomonile tiedjei DSM 6799
Archangium gephyra
Nitrospira moscoviensis
Gemmatimonas phototrophica
Gemmatimonas aurantiaca T-27
Chloracidobacterium thermophilum B
Methylacidiphilum fumariolicum SolV
Kiritimatiella glycovorans
Rhodococcus jostii RHA1
Catenulispora acidiphila DSM 44928
Streptosporangium roseum DSM 43021
Streptomyces bingchenggensis BCW-1
Kibdelosporangium phytohabitans
Natronomonas pharaonis DSM 2160
Natronococcus occultus SP4
Natronobacterium gregoryi SP2
Halomicrobium mukohataei DSM 12286
Haloarcula hispanica ATCC 33960
Haloferax mediterranei ATCC 33500
Halogeometricum borinquense DSM 11551
Halostagnicola larsenii XH-48
Haloterrigena daqingensis
Natrinema pellirubrum DSM 15624
Natronolimnobius aegyptiacus
Haloterrigena turkmenica DSM 5511
Sulfurihydrogenibium azorense Az-Fu1
Hydrogenobacter thermophilus TK-6
Persephonella marina EX-H1
Ilyobacter polytropus DSM 2926
Thermodesulfatator indicus DSM 15286
Calditerrivibrio nitroreducens DSM 19672
Methanococcus aeolicus Nankai-3
Methanothermococcus okinawensis IH1
Methanocaldococcus infernus ME
Methanocaldococcus vulcanius M7
Methanocaldococcus bathoardescens
Methanotorris igneus Kol 5
Methanocaldococcus jannaschii DSM 2661
Methanococcus voltae A3
Methanococcus vannielii SB
Methanococcus maripaludis X1
Fusobacterium nucleatum subsp. animalis 7_1
Arcobacter nitrofigilis DSM 7299
Sulfurovum lithotrophicum
Sulfuricurvum kujiense DSM 16994
Sulfurospirillum multivorans DSM 12446
Nautilia profundicola AmH
Coraliomargarita akajimensis DSM 45221
Denitrovibrio acetiphilus DSM 12809
Saccharomyces cerevisiae S288C
Parachlamydia acanthamoebae UV-7
Spirosoma aerolatum
Haliscomenobacter hydrossis DSM 1100
Flavobacterium johnsoniae UW101
Chlamydia psittaci 6BC
Candidatus Protochlamydia naegleriophila
Pedobacter steynii
Chitinophaga pinensis DSM 2588
Turneriella parva DSM 21527
Leptospira alstonii
Novosphingobium resinovorum
Novosphingobium resinovorum
Bradyrhizobium japonicum USDA 6
Desulfurispirillum indicum S5
Flexistipes sinusarabici DSM 4947
Flexistipes sinusarabici DSM 4947
Fibrobacter succinogenes subsp. succinogenes S85
Brachyspira murdochii DSM 12563
Clostridium saccharoperbutylacetonicum N1-4(HMT)
Methanobrevibacter olleyae
Methanobrevibacter ruminantium M1
Thaumarchaeota archaeon RRmetagenome_bin19
Phycisphaera mikurensis NBRC 102666
Candidatus Nitrososphaera gargensis Ga9.2
Nitrososphaera viennensis EN76
Candidatus Nitrososphaera evergladensis SR1
Candidatus Nitrosocosmicus franklandus C13
Candidatus Nitrosocosmicus exaquare G61
Nitrosocosmicus arcticus Kfb
Candidatus Nitrosocosmicus oleophilus MY3
Nitrosotalea bavarica SbT1
Nitrosotalea sinensis Nd2
Nitrosotalea okcheonensis CS
Candidatus Nitrosotalea devanaterra Nd1
Thaumarchaeota archaeon 13_1_40CM_3_38_6
Candidatus Nitrosopelagicus brevis CN25
Marine Group I thaumarchaeote SCGC AAA007-O23
Candidatus Nitrosotenuis aquarius
Candidatus Nitrosotenuis cloacae SAT1
Nitrosotenuis chungbukensis MY2
Nitrosotenuis uzonensis N4
Cenarchaeum symbiosum A
Gamma AOA mg3 - D17
Nitrosoarchaeum limnia SFB1
Archaeon CSP1
Nitrosoarchaeum koreensis MY1
Candidatus Nitrosopumilus adriaticus NF5
Nitrosopumilus sp. NM25
Candidatus Nitrosomarinus catalina SPOT01
Thaumarchaeota archaeon Baikal-G2
Candidatus Nitrosopumilus sediminis AR2
Marine Group I thaumarchaeote SCGC RSA3
Nitrosopumilus sp SJ
Candidatus Nitrosopumilus koreensis AR1
Nitrosopumilus maritimus SCM1
Candidatus Nitrosopumilus piranensis D3C
Alpha AOA ma8 - T3L19
Candidatus Nitrosocaldus islandicus
Candidatus Nitrosocaldus cavascurensis SCU2
Thaumarchaeota archaeon J079
Moorea producens PAL-8-15-08-1
Stanieria cyanosphaera PCC 7437
Nostoc punctiforme PCC 73102
Gloeobacter kilaueensis JS1
Acaryochloris marina MBIC11017
Guillardia theta CCMP2712
Geobacter uraniireducens Rf4
Geobacter uraniireducens Rf4
Simkania negevensis Z
Phytophthora parasitica INRA-310
Waddlia chondrophila WSU 86-1044
Candidatus Protochlamydia naegleriophila
Parachlamydia acanthamoebae UV-7
Halomicrobium mukohataei DSM 12286
Hahella chejuensis KCTC 2396
Methanotorris igneus Kol 5
Methanocaldococcus jannaschii DSM 2661
Candidatus Nitrosocosmicus franklandus C13
Nitrosopumilus sp SJ
Candidatus Nitrosopumilus koreensis AR1
Candidatus Nitrosopelagicus brevis CN25
Gamma AOA mg3 - D17
76G-FAM007439
76G-FAM007526

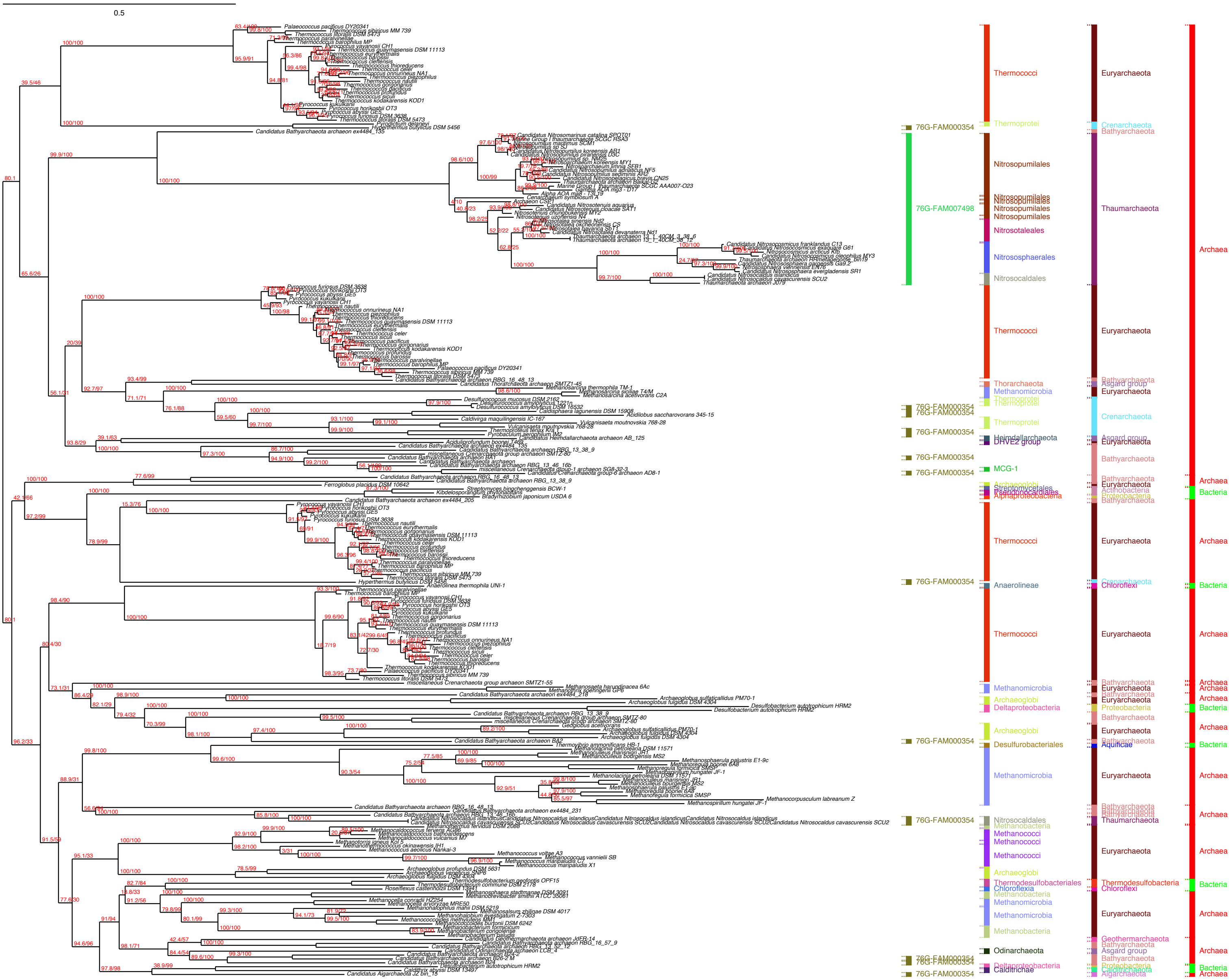
0.5
Thermococci
Euryarchaeota
Thermoprotei
Crenarchaeota
Bathyarchaeota
76G-FAM000354
Nitrosopumilales
76G-FAM007498
Thaumarchaeota
Archaea
Nitrosotaleales
Nitrososphaerales
Nitrosocaldales
Thorarchaeota
Asgard group
Methanomicrobia
Heimdallarchaeota
DPANN group
MCG-1
Archaeoglobi
Streptomycetales
Pseudonocardiales
Alphaproteobacteria
Actinobacteria
Proteobacteria
Bacteria
Anaerolineae
Chloroflexi
Deltaproteobacteria
Desulfurobacteriales
Aquificae
Methanobacteria
Methanococci
Thermodesulfobacteriales
Thermodesulfobacteria
Chloroflexia
Geothermarchaeota
Odinarchaeota
Calditrichae
Aigarchaeota

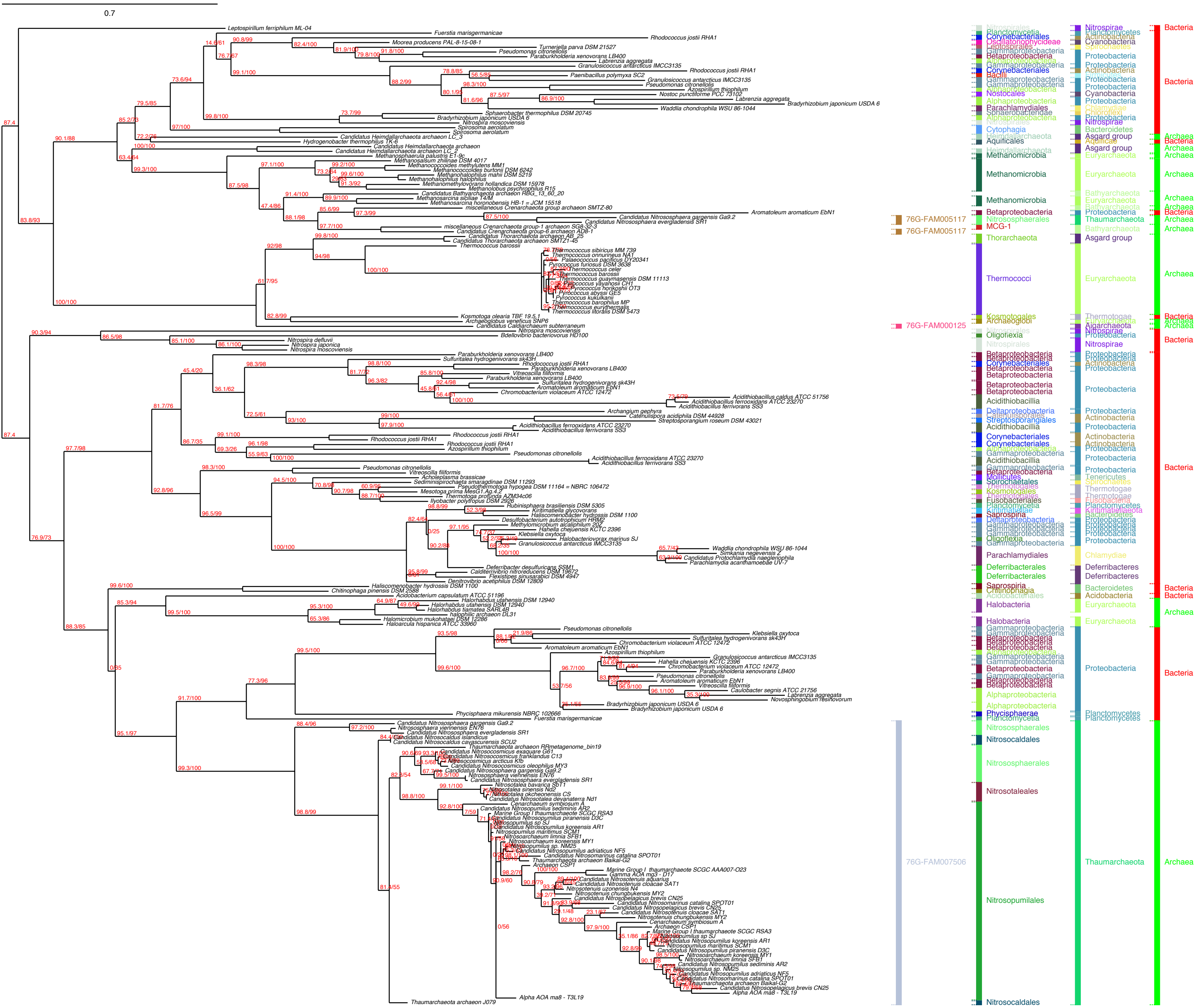
0.7
Leptospirillum ferriphilum ML-04
Fuerstia marisgermanicae
Rhodococcus jostii RHA1
Moorea producens PAL-8-15-08-1
Turneriella parva DSM 21527
Pseudomonas citronellolis
Paraburkholderia xenovorans LB400
Aromatoleum aromaticum EbN1
Candidatus Nitrososphaera gargensis Ga9.2
Candidatus Nitrososphaera evergladensis SR1
Kosmotoga olearia TBF 19.5.1
Archaeoglobus veneficus SNP6
Candidatus Caldiarchaeum subterraneum
Nitrospira moscoviensis
Bdellovibrio bacteriovorus HD100
Nitrospira defluvii
Nitrospira japonica
Waddlia chondrophila WSU 86-1044
Simkania negevensis Z
Candidatus Protochlamydia naegleriophila
Parachlamydia acanthamoebae UV-7
Haliscomenobacter hydrossis DSM 1100
Chitinophaga pinensis DSM 2588
Phycisphaera mikurensis NBRC 102666
Thaumarchaeota archaeon J079
Alpha AOA ma8 - T3L19
76G-FAM005117
76G-FAM005117
76G-FAM000125
76G-FAM007506
Nitrospirales
Planctomycetia
Corynebacteriales
Oscillatoriophycideae
Leptospirales
Gammaproteobacteria
Betaproteobacteria
Alphaproteobacteria
Bacilli
Nostocales
Parachlamydiales
Sphaerobacteridae
Cytophagia
Heimdallarchaeota
Aquificales
Methanomicrobia
MCG-1
Nitrososphaerales
Thorarchaeota
Thermococci
Kosmotogales
Archaeoglobi
Oligoflexia
Acidithiobacillia
Deltaproteobacteria
Catenulisporales
Streptosporangiales
Mollicutes
Spirochaetales
Thermotogales
Fusobacteriales
Kiritimatiellae
Saprospiria
Deferribacterales
Chitinophagia
Acidobacteriales
Halobacteria
Phycisphaerae
Nitrosocaldales
Nitrosotaleales
Nitrosopumilales
Nitrospirae
Planctomycetes
Actinobacteria
Cyanobacteria
Spirochaetes
Proteobacteria
Chloroflexi
Chlamydiae
Bacteroidetes
Asgard group
Aquificae
Euryarchaeota
Bathyarchaeota
Thaumarchaeota
Thermotogae
Aigarchaeota
Tenericutes
Fusobacteria
Kiritimatiellaeota
Deferribacteres
Acidobacteria
Bacteria
Archaea

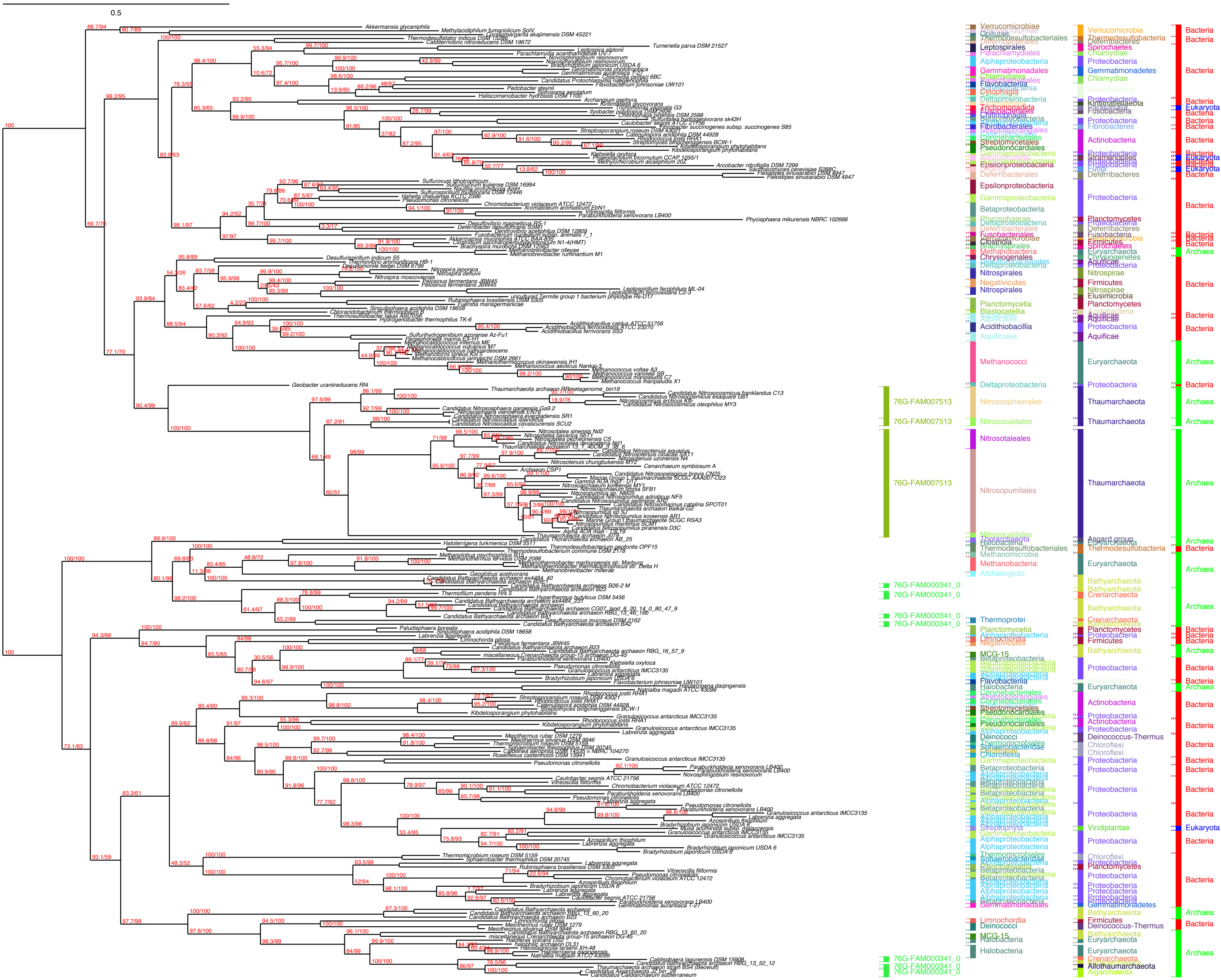
0.5
76G-FAM007513
76G-FAM000341_0
Bacteria
Archaea
Eukaryota
Thaumarchaeota
Euryarchaeota
Bathyarchaeota
Proteobacteria
Actinobacteria
Methanococci
Nitrosopumilales
Nitrosotaleales
Halobacteria

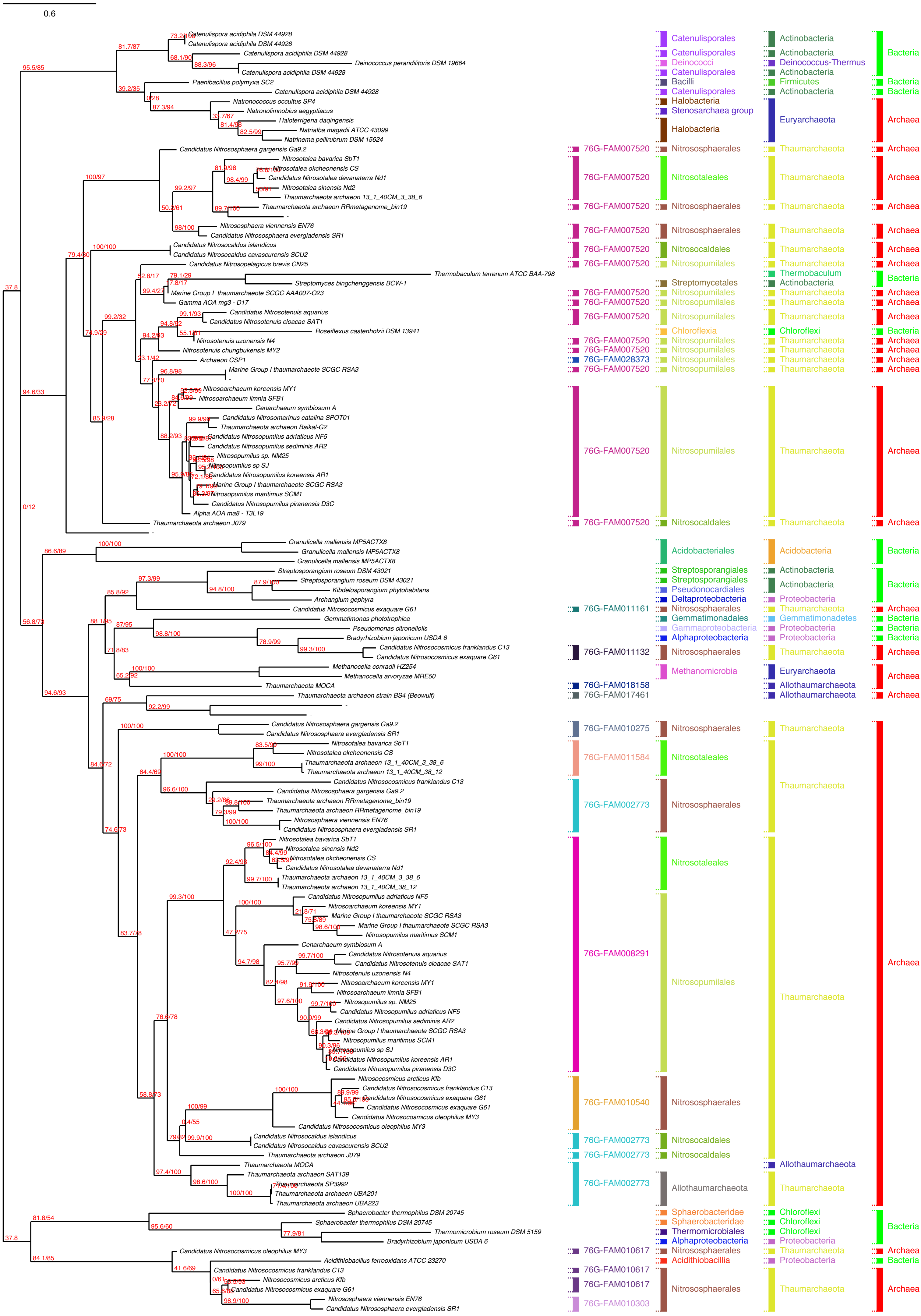

0.6
Catenulispora acidiphila DSM 44928
Catenulispora acidiphila DSM 44928
Deinococcus peraridilitoris DSM 19664
Catenulispora acidiphila DSM 44928
Paenibacillus polymyxa SC2
Catenulispora acidiphila DSM 44928
Natronococcus occultus SP4
Natronolimnobius aegyptiacus
Haloterrigena daqingensis
Natrialba magadii ATCC 43099
Natrinema pellirubrum DSM 15624
Candidatus Nitrososphaera gargensis Ga9.2
Nitrosotalea bavarica SbT1
Nitrosotalea okcheonensis CS
Candidatus Nitrosotalea devanaterra Nd1
Nitrosotalea sinensis Nd2
Thaumarchaeota archaeon 13_1_40CM_3_38_6
Thaumarchaeota archaeon RRmetagenome_bin19
Nitrososphaera viennensis EN76
Candidatus Nitrososphaera evergladensis SR1
Candidatus Nitrosocaldus islandicus
Candidatus Nitrosocaldus cavascurensis SCU2
Candidatus Nitrosopelagicus brevis CN25
Thermobaculum terrenum ATCC BAA-798
Streptomyces bingchenggensis BCW-1
Marine Group I thaumarchaeote SCGC AAA007-O23
Gamma AOA mg3 - D17
Candidatus Nitrosotenuis aquarius
Candidatus Nitrosotenuis cloacae SAT1
Roseiflexus castenholzii DSM 13941
Nitrosotenuis uzonensis N4
Nitrosotenuis chungbukensis MY2
Archaeon CSP1
Marine Group I thaumarchaeote SCGC RSA3
Nitrosoarchaeum koreensis MY1
Nitrosoarchaeum limnia SFB1
Cenarchaeum symbiosum A
Candidatus Nitrosomarinus catalina SPOT01
Thaumarchaeota archaeon Baikal-G2
Candidatus Nitrosopumilus adriaticus NF5
Candidatus Nitrosopumilus sediminis AR2
Nitrosopumilus sp. NM25
Nitrosopumilus sp SJ
Candidatus Nitrosopumilus koreensis AR1
Marine Group I thaumarchaeote SCGC RSA3
Nitrosopumilus maritimus SCM1
Candidatus Nitrosopumilus piranensis D3C
Alpha AOA ma8 - T3L19
Thaumarchaeota archaeon J079
Granulicella mallensis MP5ACTX8
Granulicella mallensis MP5ACTX8
Granulicella mallensis MP5ACTX8
Streptosporangium roseum DSM 43021
Streptosporangium roseum DSM 43021
Kibdelosporangium phytohabitans
Archangium gephyra
Candidatus Nitrosocosmicus exaquare G61
Gemmatimonas phototrophica
Pseudomonas citronellolis
Bradyrhizobium japonicum USDA 6
Candidatus Nitrosocosmicus franklandus C13
Candidatus Nitrosocosmicus exaquare G61
Methanocella conradii HZ254
Methanocella arvoryzae MRE50
Thaumarchaeota MOCA
Thaumarchaeota archaeon strain BS4 (Beowulf)
Candidatus Nitrososphaera gargensis Ga9.2
Candidatus Nitrososphaera evergladensis SR1
Nitrosotalea bavarica SbT1
Nitrosotalea okcheonensis CS
Thaumarchaeota archaeon 13_1_40CM_3_38_6
Thaumarchaeota archaeon 13_1_40CM_38_12
Candidatus Nitrosocosmicus franklandus C13
Candidatus Nitrososphaera gargensis Ga9.2
Thaumarchaeota archaeon RRmetagenome_bin19
Thaumarchaeota archaeon RRmetagenome_bin19
Nitrososphaera viennensis EN76
Candidatus Nitrososphaera evergladensis SR1
Nitrosotalea bavarica SbT1
Nitrosotalea sinensis Nd2
Nitrosotalea okcheonensis CS
Candidatus Nitrosotalea devanaterra Nd1
Thaumarchaeota archaeon 13_1_40CM_3_38_6
Thaumarchaeota archaeon 13_1_40CM_38_12
Candidatus Nitrosopumilus adriaticus NF5
Nitrosoarchaeum koreensis MY1
Marine Group I thaumarchaeote SCGC RSA3
Marine Group I thaumarchaeote SCGC RSA3
Nitrosopumilus maritimus SCM1
Cenarchaeum symbiosum A
Candidatus Nitrosotenuis aquarius
Candidatus Nitrosotenuis cloacae SAT1
Nitrosotenuis uzonensis N4
Nitrosoarchaeum koreensis MY1
Nitrosoarchaeum limnia SFB1
Nitrosopumilus sp. NM25
Candidatus Nitrosopumilus adriaticus NF5
Candidatus Nitrosopumilus sediminis AR2
Marine Group I thaumarchaeote SCGC RSA3
Nitrosopumilus maritimus SCM1
Nitrosopumilus sp SJ
Candidatus Nitrosopumilus koreensis AR1
Candidatus Nitrosopumilus piranensis D3C
Nitrosocosmicus arcticus Kfb
Candidatus Nitrosocosmicus franklandus C13
Candidatus Nitrosocosmicus exaquare G61
Candidatus Nitrosocosmicus exaquare G61
Candidatus Nitrosocosmicus oleophilus MY3
Candidatus Nitrosocosmicus oleophilus MY3
Candidatus Nitrosocaldus islandicus
Candidatus Nitrosocaldus cavascurensis SCU2
Thaumarchaeota archaeon J079
Thaumarchaeota MOCA
Thaumarchaeota archaeon SAT139
Thaumarchaeota SP3992
Thaumarchaeota archaeon UBA201
Thaumarchaeota archaeon UBA223
Sphaerobacter thermophilus DSM 20745
Sphaerobacter thermophilus DSM 20745
Thermomicrobium roseum DSM 5159
Bradyrhizobium japonicum USDA 6
Candidatus Nitrosocosmicus oleophilus MY3
Acidithiobacillus ferrooxidans ATCC 23270
Candidatus Nitrosocosmicus franklandus C13
Nitrosocosmicus arcticus Kfb
Candidatus Nitrosocosmicus exaquare G61
Nitrososphaera viennensis EN76
Candidatus Nitrososphaera evergladensis SR1
76G-FAM007520
76G-FAM028373
76G-FAM011161
76G-FAM011132
76G-FAM018158
76G-FAM017461
76G-FAM010275
76G-FAM011584
76G-FAM002773
76G-FAM008291
76G-FAM010540
76G-FAM010617
76G-FAM010303
Catenulisporales
Deinococci
Bacilli
Halobacteria
Stenosarchaea group
Nitrososphaerales
Nitrosotaleales
Nitrosocaldales
Nitrosopumilales
Streptomycetales
Chloroflexia
Acidobacteriales
Streptosporangiales
Pseudonocardiales
Deltaproteobacteria
Gemmatimonadales
Gammaproteobacteria
Alphaproteobacteria
Methanomicrobia
Allothaumarchaeota
Sphaerobacteridae
Thermomicrobiales
Acidithiobacillia
Actinobacteria
Deinococcus-Thermus
Firmicutes
Euryarchaeota
Thaumarchaeota
Thermobaculum
Chloroflexi
Acidobacteria
Proteobacteria
Gemmatimonadetes
Bacteria
Archaea

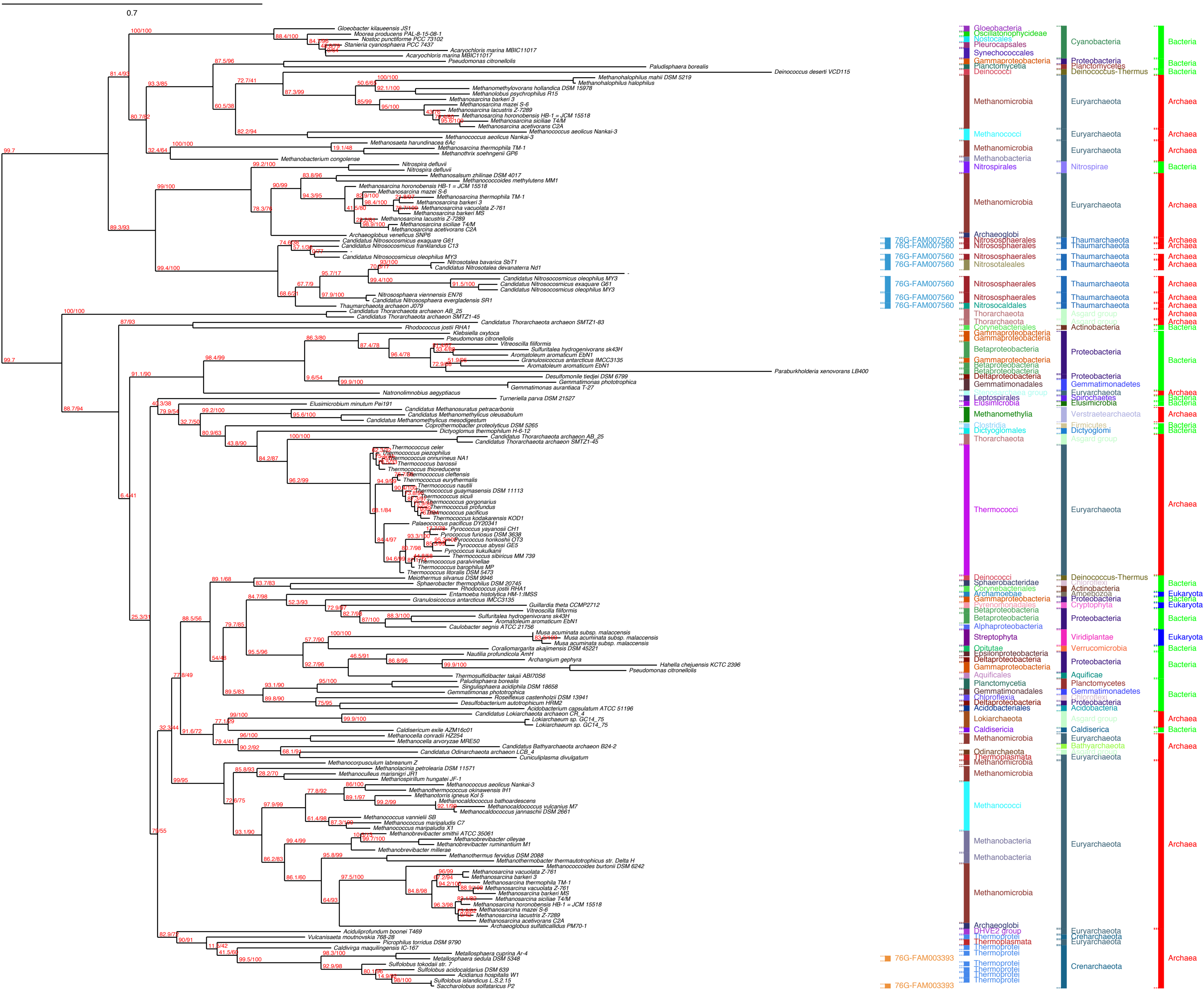

0.7
Gloeobacter kilaueensis JS1
Moorea producens PAL-8-15-08-1
Nostoc punctiforme PCC 73102
Stanieria cyanosphaera PCC 7437
Acaryochloris marina MBIC11017
Acaryochloris marina MBIC11017
Pseudomonas citronellolis
Paludisphaera borealis
Deinococcus deserti VCD115
Methanohalophilus mahii DSM 5219
Methanohalophilus halophilus
Methanomethylovorans hollandica DSM 15978
Methanolobus psychrophilus R15
Methanosarcina barkeri 3
Methanosarcina mazei S-6
Methanosarcina lacustris Z-7289
Methanosarcina horonobensis HB-1 = JCM 15518
Methanosarcina siciliae T4/M
Methanosarcina acetivorans C2A
Methanococcus aeolicus Nankai-3
Methanococcus aeolicus Nankai-3
Methanosaeta harundinacea 6Ac
Methanothrix thermophila TM-1
Methanothrix soehngenii GP6
Methanobacterium congolense
Nitrospira defluvii
Nitrospira defluvii
Methanosalsum zhilinae DSM 4017
Methanococcoides methylutens MM1
Methanosarcina horonobensis HB-1 = JCM 15518
Methanosarcina mazei S-6
Methanosarcina thermophila TM-1
Methanosarcina barkeri 3
Methanosarcina vacuolata Z-761
Methanosarcina barkeri MS
Methanosarcina lacustris Z-7289
Methanosarcina siciliae T4/M
Methanosarcina acetivorans C2A
Archaeoglobus veneficus SNP6
Candidatus Nitrosocosmicus exaquare G61
Candidatus Nitrosocosmicus franklandus C13
Candidatus Nitrosocosmicus oleophilus MY3
Nitrosotalea bavarica SbT1
Candidatus Nitrosotalea devanaterra Nd1
Candidatus Nitrosocosmicus oleophilus MY3
Candidatus Nitrosocosmicus exaquare G61
Candidatus Nitrosocosmicus oleophilus MY3
Nitrososphaera viennensis EN76
Candidatus Nitrososphaera evergladensis SR1
Thaumarchaeota archaeon J079
Candidatus Thorarchaeota archaeon AB_25
Candidatus Thorarchaeota archaeon SMTZ1-45
Candidatus Thorarchaeota archaeon SMTZ1-83
Rhodococcus jostii RHA1
Klebsiella oxytoca
Pseudomonas citronellolis
Vitreoscilla filiformis
Sulfuritalea hydrogenivorans sk43H
Aromatoleum aromaticum EbN1
Granulosicoccus antarcticus IMCC3135
Aromatoleum aromaticum EbN1
Paraburkholderia xenovorans LB400
Desulfomonile tiedjei DSM 6799
Gemmatimonas phototrophica
Gemmatimonas aurantiaca T-27
Natronolimnobius aegyptiacus
Turneriella parva DSM 21527
Elusimicrobium minutum Pei191
Candidatus Methanosuratus petracarbonis
Candidatus Methanomethylicus oleusabulum
Candidatus Methanomethylicus mesodigestum
Coprothermobacter proteolyticus DSM 5265
Dictyoglomus thermophilum H-6-12
Candidatus Thorarchaeota archaeon AB_25
Candidatus Thorarchaeota archaeon SMTZ1-45
Thermococcus celer
Thermococcus piezophilus
Thermococcus onnurineus NA1
Thermococcus barossii
Thermococcus thioreducens
Thermococcus cleftensis
Thermococcus eurythermalis
Thermococcus nautili
Thermococcus guaymasensis DSM 11113
Thermococcus siculi
Thermococcus gorgonarius
Thermococcus profundus
Thermococcus pacificus
Thermococcus kodakarensis KOD1
Palaeococcus pacificus DY20341
Pyrococcus yayanosii CH1
Pyrococcus furiosus DSM 3638
Pyrococcus horikoshii OT3
Pyrococcus abyssi GE5
Pyrococcus kukulkanii
Thermococcus sibiricus MM 739
Thermococcus paralvinellae
Thermococcus barophilus MP
Thermococcus litoralis DSM 5473
Meiothermus silvanus DSM 9946
Sphaerobacter thermophilus DSM 20745
Rhodococcus jostii RHA1
Entamoeba histolytica HM-1:IMSS
Granulosicoccus antarcticus IMCC3135
Guillardia theta CCMP2712
Vitreoscilla filiformis
Sulfuritalea hydrogenivorans sk43H
Aromatoleum aromaticum EbN1
Caulobacter segnis ATCC 21756
Musa acuminata subsp. malaccensis
Musa acuminata subsp. malaccensis
Musa acuminata subsp. malaccensis
Coraliomargarita akajimensis DSM 45221
Nautilia profundicola AmH
Archangium gephyra
Hahella chejuensis KCTC 2396
Pseudomonas citronellolis
Thermosulfidibacter takaii ABI70S6
Paludisphaera borealis
Singulisphaera acidiphila DSM 18658
Gemmatimonas phototrophica
Roseiflexus castenholzii DSM 13941
Desulfobacterium autotrophicum HRM2
Acidobacterium capsulatum ATCC 51196
Candidatus Lokiarchaeota archaeon CR_4
Lokiarchaeum sp. GC14_75
Lokiarchaeum sp. GC14_75
Caldisericum exile AZM16c01
Methanocella conradii HZ254
Methanocella arvoryzae MRE50
Candidatus Bathyarchaeota archaeon B24-2
Candidatus Odinarchaeota archaeon LCB_4
Cuniculiplasma divulgatum
Methanocorpusculum labreanum Z
Methanolacinia petrolearia DSM 11571
Methanoculleus marisnigri JR1
Methanospirillum hungatei JF-1
Methanococcus aeolicus Nankai-3
Methanothermococcus okinawensis IH1
Methanotorris igneus Kol 5
Methanocaldococcus bathoardescens
Methanocaldococcus vulcanius M7
Methanocaldococcus jannaschii DSM 2661
Methanococcus vannielii SB
Methanococcus maripaludis C7
Methanococcus maripaludis X1
Methanobrevibacter smithii ATCC 35061
Methanobrevibacter olleyae
Methanobrevibacter ruminantium M1
Methanobrevibacter millerae
Methanothermus fervidus DSM 2088
Methanothermobacter thermautotrophicus str. Delta H
Methanococcoides burtonii DSM 6242
Methanosarcina vacuolata Z-761
Methanosarcina barkeri 3
Methanosarcina thermophila TM-1
Methanosarcina vacuolata Z-761
Methanosarcina barkeri MS
Methanosarcina siciliae T4/M
Methanosarcina horonobensis HB-1 = JCM 15518
Methanosarcina mazei S-6
Methanosarcina lacustris Z-7289
Methanosarcina acetivorans C2A
Archaeoglobus sulfaticallidus PM70-1
Aciduliprofundum boonei T469
Vulcanisaeta moutnovskia 768-28
Picrophilus torridus DSM 9790
Caldivirga maquilingensis IC-167
Metallosphaera cuprina Ar-4
Metallosphaera sedula DSM 5348
Sulfolobus tokodaii str. 7
Sulfolobus acidocaldarius DSM 639
Acidianus hospitalis W1
Sulfolobus islandicus L.S.2.15
Saccharolobus solfataricus P2
76G-FAM007560
76G-FAM007560
76G-FAM007560
76G-FAM007560
76G-FAM007560
76G-FAM007560
76G-FAM007560
76G-FAM003393
76G-FAM003393
Gloeobacteria
Oscillatoriophycideae
Nostocales
Pleurocapsales
Synechococcales
Gammaproteobacteria
Planctomycetia
Deinococci
Methanomicrobia
Methanococci
Methanomicrobia
Methanobacteria
Nitrospirales
Methanomicrobia
Archaeoglobi
Nitrososphaerales
Nitrososphaerales
Nitrososphaerales
Nitrosotaleales
Nitrososphaerales
Nitrososphaerales
Nitrosocaldales
Thorarchaeota
Thorarchaeota
Corynebacteriales
Gammaproteobacteria
Gammaproteobacteria
Betaproteobacteria
Gammaproteobacteria
Betaproteobacteria
Betaproteobacteria
Deltaproteobacteria
Gemmatimonadales
Leptospirales
Elusimicrobia
Methanomethylia
Clostridia
Dictyoglomales
Thorarchaeota
Thermococci
Deinococci
Sphaerobacteridae
Corynebacteriales
Archamoebae
Gammaproteobacteria
Pyrenomonadales
Betaproteobacteria
Betaproteobacteria
Alphaproteobacteria
Streptophyta
Opitutae
Epsilonproteobacteria
Deltaproteobacteria
Gammaproteobacteria
Aquificales
Planctomycetia
Gemmatimonadales
Chloroflexia
Deltaproteobacteria
Acidobacteriales
Lokiarchaeota
Caldisericia
Methanomicrobia
Odinarchaeota
Thermoplasmata
Methanomicrobia
Methanomicrobia
Methanococci
Methanobacteria
Methanobacteria
Methanomicrobia
Archaeoglobi
DHVE2 group
Thermoprotei
Thermoplasmata
Thermoprotei
Thermoprotei
Thermoprotei
Thermoprotei
Thermoprotei
Thermoprotei
Cyanobacteria
Proteobacteria
Planctomycetes
Deinococcus-Thermus
Euryarchaeota
Euryarchaeota
Euryarchaeota
Nitrospirae
Euryarchaeota
Thaumarchaeota
Thaumarchaeota
Thaumarchaeota
Thaumarchaeota
Thaumarchaeota
Thaumarchaeota
Thaumarchaeota
Asgard group
Asgard group
Actinobacteria
Proteobacteria
Proteobacteria
Gemmatimonadetes
Euryarchaeota
Spirochaetes
Elusimicrobia
Verstraetearchaeota
Firmicutes
Dictyoglomi
Asgard group
Euryarchaeota
Deinococcus-Thermus
Chloroflexi
Actinobacteria
Amoebozoa
Proteobacteria
Cryptophyta
Proteobacteria
Viridiplantae
Verrucomicrobia
Proteobacteria
Aquificae
Planctomycetes
Gemmatimonadetes
Chloroflexi
Proteobacteria
Acidobacteria
Asgard group
Caldiserica
Euryarchaeota
Bathyarchaeota
Euryarchaeota
Euryarchaeota
Euryarchaeota
Crenarchaeota
Euryarchaeota
Crenarchaeota
Bacteria
Bacteria
Bacteria
Archaea
Archaea
Archaea
Bacteria
Archaea
Archaea
Archaea
Archaea
Archaea
Archaea
Archaea
Archaea
Archaea
Archaea
Bacteria
Bacteria
Archaea
Bacteria
Bacteria
Archaea
Bacteria
Bacteria
Archaea
Bacteria
Eukaryota
Bacteria
Eukaryota
Bacteria
Eukaryota
Bacteria
Bacteria
Bacteria
Archaea
Bacteria
Archaea
Archaea
Archaea

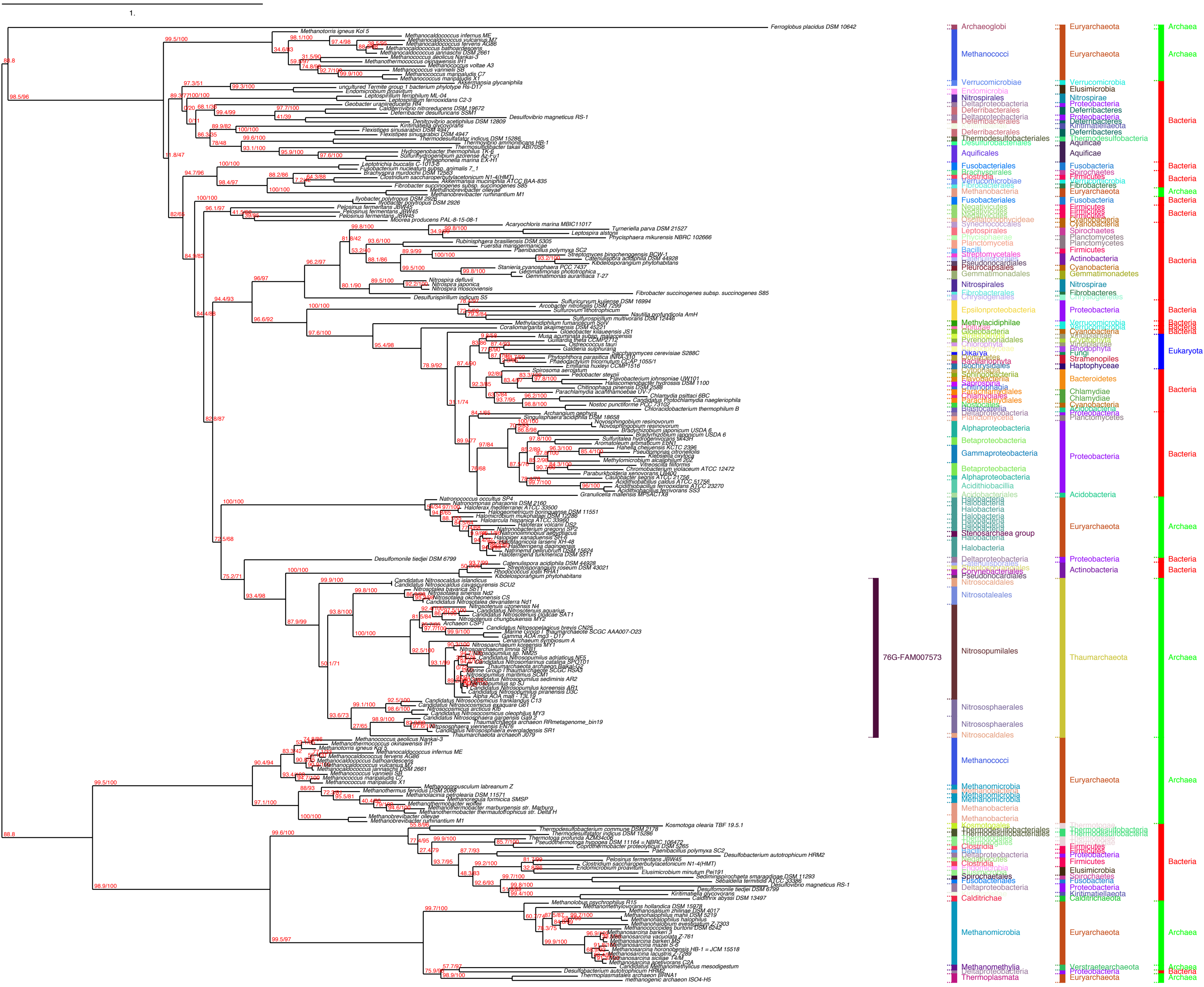
1.
76G-FAM007573
Ferroglobus placidus DSM 10642
Methanotorris igneus Kol 5
Methanocaldococcus infernus ME
Methanocaldococcus vulcanius M7
Methanocaldococcus fervens AG86
Methanocaldococcus bathoardescens
Methanocaldococcus jannaschii DSM 2661
Methanococcus aeolicus Nankai-3
Methanothermococcus okinawensis IH1
Methanococcus voltae A3
Methanococcus vannielii SB
Methanococcus maripaludis C7
Methanococcus maripaludis X1
Akkermansia glycaniphila
uncultured Termite group 1 bacterium phylotype Rs-D17
Endomicrobium proavitum
Leptospirillum ferriphilum ML-04
Leptospirillum ferrooxidans C2-3
Geobacter uraniireducens Rf4
Calditerrivibrio nitroreducens DSM 19672
Deferribacter desulfuricans SSM1
Desulfovibrio magneticus RS-1
Denitrovibrio acetiphilus DSM 12809
Kiritimatiella glycovorans
Flexistipes sinusarabici DSM 4947
Thermodesulfatator indicus DSM 15286
Thermovibrio ammonificans HB-1
Thermosulfidibacter takaii ABI70S6
Hydrogenobacter thermophilus TK-6
Sulfurihydrogenibium azorense Az-Fu1
Persephonella marina EX-H1
Leptotrichia buccalis C-1013-b
Fusobacterium nucleatum subsp. animalis 7_1
Brachyspira murdochii DSM 12563
Clostridium saccharoperbutylacetonicum N1-4(HMT)
Akkermansia muciniphila ATCC BAA-835
Fibrobacter succinogenes subsp. succinogenes S85
Methanobrevibacter olleyae
Methanobrevibacter ruminantium M1
Ilyobacter polytropus DSM 2926
Pelosinus fermentans JBW45
Moorea producens PAL-8-15-08-1
Acaryochloris marina MBIC11017
Turneriella parva DSM 21527
Leptospira alstonii
Phycisphaera mikurensis NBRC 102666
Rubinisphaera brasiliensis DSM 5305
Fuerstia marisgermanicae
Paenibacillus polymyxa SC2
Streptomyces bingchenggensis BCW-1
Catenulispora acidiphila DSM 44928
Kibdelosporangium phytohabitans
Stanieria cyanosphaera PCC 7437
Gemmatimonas phototrophica
Gemmatimonas aurantiaca T-27
Nitrospira defluvii
Nitrospira japonica
Nitrospira moscoviensis
Fibrobacter succinogenes subsp. succinogenes S85
Desulfurispirillum indicum S5
Sulfuricurvum kujiense DSM 16994
Arcobacter nitrofigilis DSM 7299
Sulfurovum lithotrophicum
Nautilia profundicola AmH
Sulfurospirillum multivorans DSM 12446
Methylacidiphilum fumariolicum SolV
Coraliomargarita akajimensis DSM 45221
Gloeobacter kilaueensis JS1
Musa acuminata subsp. malaccensis
Guillardia theta CCMP2712
Ostreococcus tauri
Galdieria sulphuraria
Saccharomyces cerevisiae S288C
Phytophthora parasitica INRA-310
Phaeodactylum tricornutum CCAP 1055/1
Emiliania huxleyi CCMP1516
Spirosoma aerolatum
Pedobacter stevpii
Flavobacterium johnsoniae UW101
Haliscomenobacter hydrossis DSM 1100
Chitinophaga pinensis DSM 2588
Parachlamydia acanthamoebae UV-7
Chlamydia psittaci 6BC
Candidatus Protochlamydia naegleriophila
Nostoc punctiforme PCC 73102
Chloracidobacterium thermophilum B
Archangium gephyra
Singulisphaera acidiphila DSM 18658
Novosphingobium resinovorum
Bradyrhizobium japonicum USDA 6
Sulfuritalea hydrogenivorans sk43H
Aromatoleum aromaticum EbN1
Hahella chejuensis KCTC 2396
Pseudomonas citronellolis
Klebsiella oxytoca
Methylomicrobium alcaliphilum 20Z
Vitreoscilla filiformis
Chromobacterium violaceum ATCC 12472
Paraburkholderia xenovorans LB400
Caulobacter segnis ATCC 21756
Acidithiobacillus caldus ATCC 51756
Acidithiobacillus ferrooxidans ATCC 23270
Acidithiobacillus ferrivorans SS3
Granulicella mallensis MP5ACTX8
Natronococcus occultus SP4
Natronomonas pharaonis DSM 2160
Haloferax mediterranei ATCC 33500
Halogeometricum borinquense DSM 11551
Halomicrobium mukohataei DSM 12286
Haloarcula hispanica ATCC 33960
Haloferax volcanii DS2
Natronobacterium gregoryi SP2
Natronolimnobius aegyptiacus
Halopiger xanaduensis SH-6
Halostagnicola larsenii XH-48
Haloterrigena daqingensis
Natrinema pellirubrum DSM 15624
Haloterrigena turkmenica DSM 5511
Desulfomonile tiedjei DSM 6799
Catenulispora acidiphila DSM 44928
Streptosporangium roseum DSM 43021
Rhodococcus jostii RHA1
Kibdelosporangium phytohabitans
Candidatus Nitrosocaldus islandicus
Candidatus Nitrosocaldus cavascurensis SCU2
Nitrosotalea bavarica SbT1
Nitrosotalea sinensis Nd2
Nitrosotalea okcheonensis CS
Candidatus Nitrosotalea devanaterra Nd1
Nitrosotenuis uzonensis N4
Candidatus Nitrosotenuis aquarius
Candidatus Nitrosotenuis cloacae SAT1
Nitrosotenuis chungbukensis MY2
Archaeon CSP1
Candidatus Nitrosopelagicus brevis CN25
Marine Group I thaumarchaeote SCGC AAA007-O23
Gamma AOA mg3 - D17
Cenarchaeum symbiosum A
Nitrosoarchaeum koreensis MY1
Nitrosoarchaeum limnia SFB1
Nitrosopumilus sp. NM25
Candidatus Nitrosopumilus adriaticus NF5
Candidatus Nitrosomarinus catalina SPOT01
Thaumarchaeota archaeon Baikal-G2
Marine Group I thaumarchaeote SCGC RSA3
Nitrosopumilus maritimus SCM1
Candidatus Nitrosopumilus sediminis AR2
Nitrosopumilus sp SJ
Candidatus Nitrosopumilus koreensis AR1
Candidatus Nitrosopumilus piranensis D3C
Alpha AOA ma8 - T3L15
Candidatus Nitrosocosmicus franklandus C13
Candidatus Nitrosocosmicus exaquare G61
Nitrosocosmicus arcticus Kfb
Candidatus Nitrosocosmicus oleophilus MY3
Candidatus Nitrososphaera gargensis Ga9.2
Thaumarchaeota archaeon RRmetagenome_bin19
Nitrososphaera viennensis EN76
Candidatus Nitrososphaera evergladensis SR1
Thaumarchaeota archaeon J079
Methanococcus aeolicus Nankai-3
Methanothermococcus okinawensis IH1
Methanotorris igneus Kol 5
Methanocaldococcus infernus ME
Methanocaldococcus fervens AG86
Methanocaldococcus bathoardescens
Methanocaldococcus vulcanius M7
Methanocaldococcus jannaschii DSM 2661
Methanococcus vannielii SB
Methanococcus maripaludis C7
Methanococcus maripaludis X1
Methanocorpusculum labreanum Z
Methanothermus fervidus DSM 2088
Methanolacinia petrolearia DSM 11571
Methanoregula formicica SMSP
Methanothermobacter wolfeii
Methanothermobacter marburgensis str. Marburg
Methanothermobacter thermautotrophicus str. Delta H
Methanobrevibacter olleyae
Methanobrevibacter ruminantium M1
Kosmotoga olearia TBF 19.5.1
Thermodesulfobacterium commune DSM 2178
Thermodesulfatator indicus DSM 15286
Thermotoga profunda AZM34c06
Pseudothermotoga hypogea DSM 11164 = NBRC 106472
Coprothermobacter proteolyticus DSM 5265
Paenibacillus polymyxa SC2
Desulfobacterium autotrophicum HRM2
Pelosinus fermentans JBW45
Clostridium saccharoperbutylacetonicum N1-4(HMT)
Endomicrobium proavitum
Elusimicrobium minutum Pei191
Sediminispirochaeta smaragdinae DSM 11293
Sebaldella termitidis ATCC 33386
Desulfovibrio magneticus RS-1
Desulfomonile tiedjei DSM 6799
Kiritimatiella glycovorans
Caldithrix abyssi DSM 13497
Methanolobus psychrophilus R15
Methanomethylovorans hollandica DSM 15978
Methanosalsum zhilinae DSM 4017
Methanohalophilus mahii DSM 5219
Methanohalophilus halophilus
Methanohalobium evestigatum Z-7303
Methanococcoides burtonii DSM 6242
Methanosarcina barkeri 3
Methanosarcina vacuolata Z-761
Methanosarcina barkeri MS
Methanosarcina mazei S-6
Methanosarcina horonobensis HB-1 = JCM 15518
Methanosarcina lacustris Z-7289
Methanosarcina siciliae T4/M
Methanosarcina acetivorans C2A
Candidatus Methanomethylicus mesodigestum
Desulfobacterium autotrophicum HRM2
Thermoplasmatales archaeon BRNA1
methanogenic archaeon ISO4-H5
Archaeoglobi
Methanococci
Verrucomicrobiae
Endomicrobia
Nitrospirales
Deltaproteobacteria
Deferribacterales
Thermodesulfobacteriales
Desulfurobacteriales
Aquificales
Fusobacteriales
Brachyspirales
Clostridia
Fibrobacterales
Methanobacteria
Negativicutes
Oscillatoriophycideae
Synechococcales
Leptospirales
Phycisphaerae
Planctomycetia
Bacilli
Streptomycetales
Pseudonocardiales
Pleurocapsales
Gemmatimonadales
Chrysiogenales
Epsilonproteobacteria
Methylacidiphilae
Opitutae
Gloeobacteria
Chlorophyta
Dikarya
Bacillariophyta
Isochrysidales
Cytophagia
Sphingobacteriia
Flavobacteriia
Saprospiria
Chitinophagia
Parachlamydiales
Chlamydiales
Nostocales
Blastocatellia
Alphaproteobacteria
Betaproteobacteria
Gammaproteobacteria
Acidithiobacillia
Acidobacteriales
Halobacteria
Stenosarchaea group
Catenulisporales
Corynebacteriales
Nitrosocaldales
Nitrosotaleales
Nitrosopumilales
Nitrososphaerales
Methanomicrobia
Kosmotogales
Thermotogales
Spirochaetales
Calditrichae
Methanomethylia
Thermoplasmata
Euryarchaeota
Elusimicrobia
Nitrospirae
Proteobacteria
Deferribacteres
Kiritimatiellaeota
Thermodesulfobacteria
Aquificae
Fusobacteria
Spirochaetes
Verrucomicrobia
Fibrobacteres
Firmicutes
Cyanobacteria
Planctomycetes
Actinobacteria
Gemmatimonadetes
Chrysiogenetes
Viridiplantae
Rhodophyta
Fungi
Stramenopiles
Haptophyceae
Bacteroidetes
Chlamydiae
Acidobacteria
Thaumarchaeota
Thermotogae
Verstraetearchaeota
Archaea
Bacteria
Eukaryota

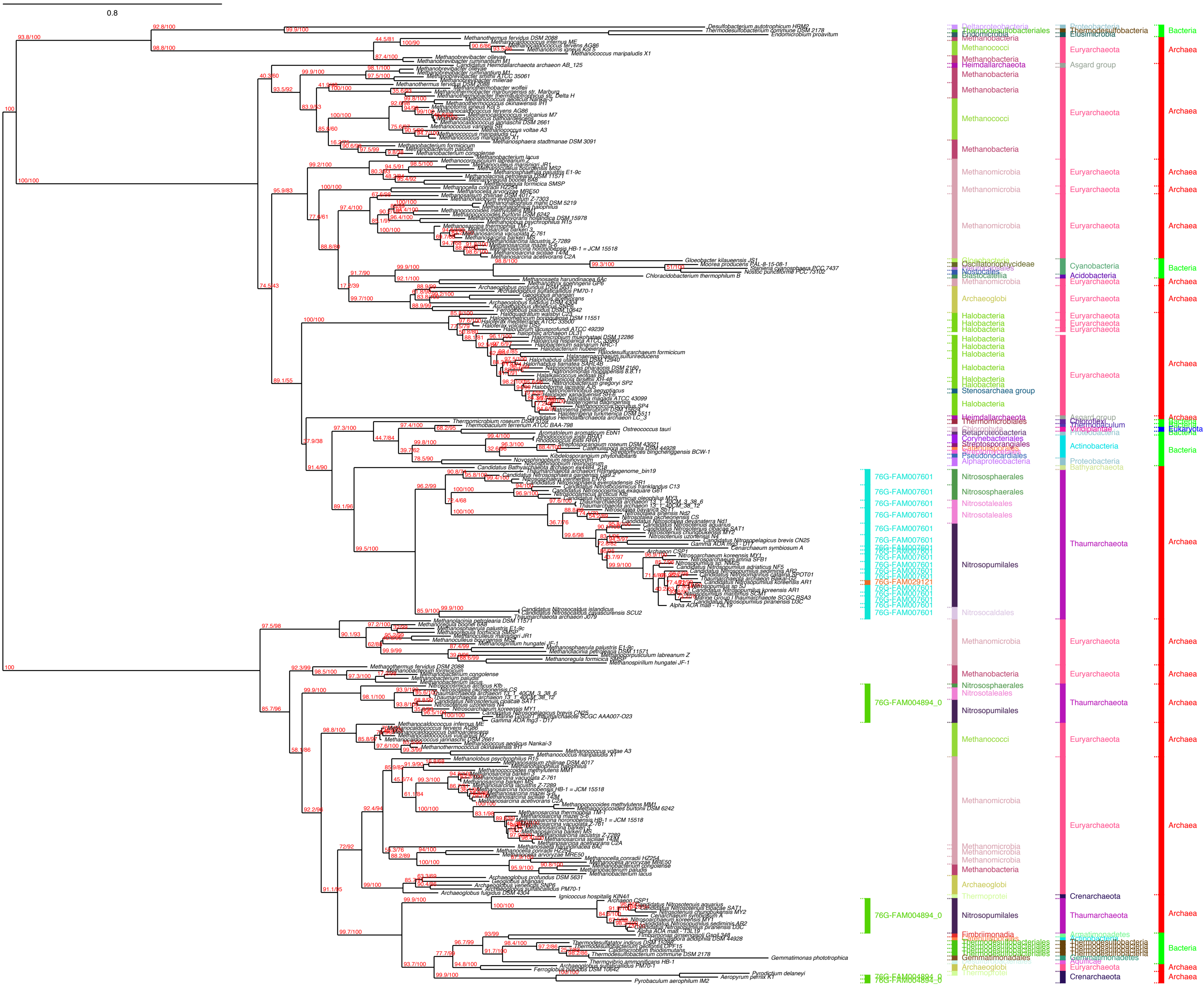

0.8
76G-FAM007601
76G-FAM029121
76G-FAM004894_0
Nitrososphaerales
Nitrosotaleales
Nitrosopumilales
Nitrosocaldales
Methanococci
Methanobacteria
Methanomicrobia
Halobacteria
Archaeoglobi
Thermoprotei
Heimdallarchaeota
Stenosarchaea group
Euryarchaeota
Thaumarchaeota
Crenarchaeota
Asgard group
Cyanobacteria
Actinobacteria
Proteobacteria
Acidobacteria
Bathyarchaeota
Thermodesulfobacteria
Bacteria
Archaea
Eukaryota

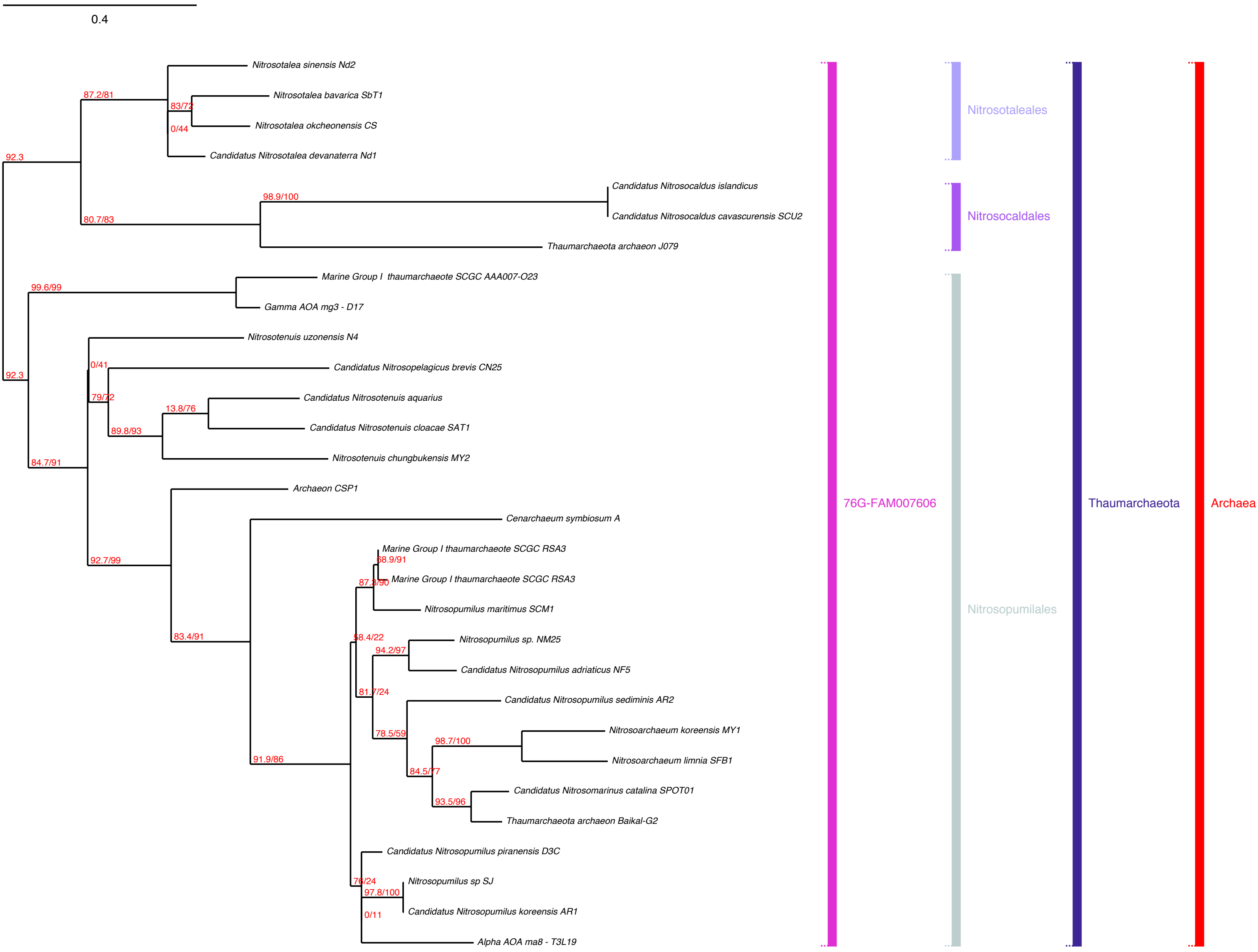
0.4
Nitrosotalea sinensis Nd2
Nitrosotalea bavarica SbT1
Nitrosotalea okcheonensis CS
Candidatus Nitrosotalea devanaterra Nd1
Candidatus Nitrosocaldus islandicus
Candidatus Nitrosocaldus cavascurensis SCU2
Thaumarchaeota archaeon J079
Marine Group I thaumarchaeote SCGC AAA007-O23
Gamma AOA mg3 - D17
Nitrosotenuis uzonensis N4
Candidatus Nitrosopelagicus brevis CN25
Candidatus Nitrosotenuis aquarius
Candidatus Nitrosotenuis cloacae SAT1
Nitrosotenuis chungbukensis MY2
Archaeon CSP1
Cenarchaeum symbiosum A
Marine Group I thaumarchaeote SCGC RSA3
Marine Group I thaumarchaeote SCGC RSA3
Nitrosopumilus maritimus SCM1
Nitrosopumilus sp. NM25
Candidatus Nitrosopumilus adriaticus NF5
Candidatus Nitrosopumilus sediminis AR2
Nitrosoarchaeum koreensis MY1
Nitrosoarchaeum limnia SFB1
Candidatus Nitrosomarinus catalina SPOT01
Thaumarchaeota archaeon Baikal-G2
Candidatus Nitrosopumilus piranensis D3C
Nitrosopumilus sp SJ
Candidatus Nitrosopumilus koreensis AR1
Alpha AOA ma8 - T3L19
92.3
87.2/81
83/72
0/44
80.7/83
98.9/100
99.6/99
92.3
0/41
79/72
13.8/76
89.8/93
84.7/91
92.7/99
68.9/91
87.3/90
58.4/22
94.2/97
81.7/24
78.5/59
98.7/100
84.5/77
93.5/96
83.4/91
91.9/86
76/24
97.8/100
0/11
76G-FAM007606
Nitrosotaleales
Nitrosocaldales
Nitrosopumilales
Thaumarchaeota
Archaea

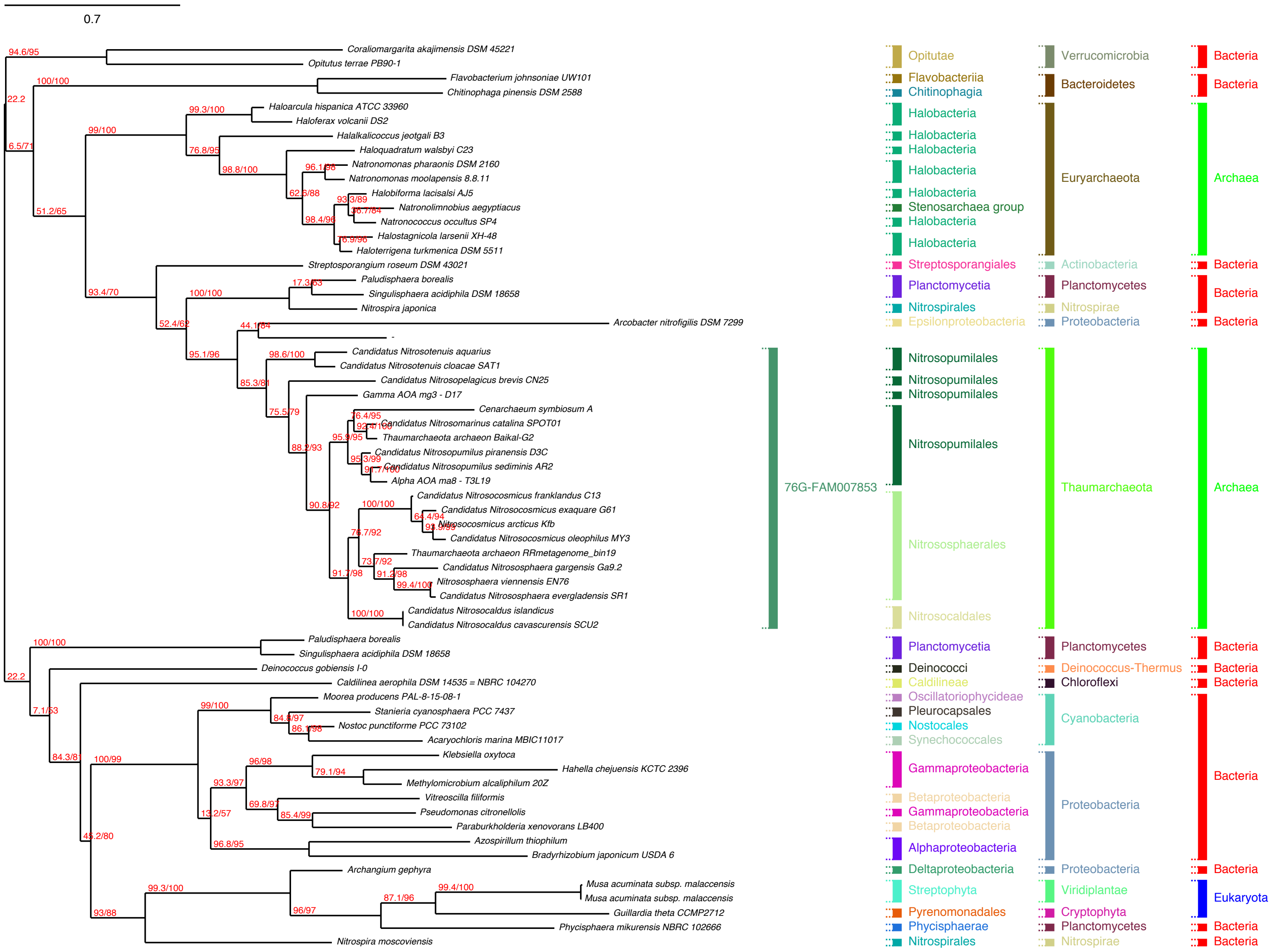
0.7
Coraliomargarita akajimensis DSM 45221
Opitutus terrae PB90-1
Flavobacterium johnsoniae UW101
Chitinophaga pinensis DSM 2588
Haloarcula hispanica ATCC 33960
Haloferax volcanii DS2
Halalkalicoccus jeotgali B3
Haloquadratum walsbyi C23
Natronomonas pharaonis DSM 2160
Natronomonas moolapensis 8.8.11
Halobiforma lacisalsi AJ5
Natronolimnobius aegyptiacus
Natronococcus occultus SP4
Halostagnicola larsenii XH-48
Haloterrigena turkmenica DSM 5511
Streptosporangium roseum DSM 43021
Paludisphaera borealis
Singulisphaera acidiphila DSM 18658
Nitrospira japonica
Arcobacter nitrofigilis DSM 7299
Candidatus Nitrosotenuis aquarius
Candidatus Nitrosotenuis cloacae SAT1
Candidatus Nitrosopelagicus brevis CN25
Gamma AOA mg3 - D17
Cenarchaeum symbiosum A
Candidatus Nitrosomarinus catalina SPOT01
Thaumarchaeota archaeon Baikal-G2
Candidatus Nitrosopumilus piranensis D3C
Candidatus Nitrosopumilus sediminis AR2
Alpha AOA ma8 - T3L19
Candidatus Nitrosocosmicus franklandus C13
Candidatus Nitrosocosmicus exaquare G61
Nitrosocosmicus arcticus Kfb
Candidatus Nitrosocosmicus oleophilus MY3
Thaumarchaeota archaeon RRmetagenome_bin19
Candidatus Nitrososphaera gargensis Ga9.2
Nitrososphaera viennensis EN76
Candidatus Nitrososphaera evergladensis SR1
Candidatus Nitrosocaldus islandicus
Candidatus Nitrosocaldus cavascurensis SCU2
Paludisphaera borealis
Singulisphaera acidiphila DSM 18658
Deinococcus gobiensis I-0
Caldilinea aerophila DSM 14535 = NBRC 104270
Moorea producens PAL-8-15-08-1
Stanieria cyanosphaera PCC 7437
Nostoc punctiforme PCC 73102
Acaryochloris marina MBIC11017
Klebsiella oxytoca
Hahella chejuensis KCTC 2396
Methylomicrobium alcaliphilum 20Z
Vitreoscilla filiformis
Pseudomonas citronellolis
Paraburkholderia xenovorans LB400
Azospirillum thiophilum
Bradyrhizobium japonicum USDA 6
Archangium gephyra
Musa acuminata subsp. malaccensis
Musa acuminata subsp. malaccensis
Guillardia theta CCMP2712
Phycisphaera mikurensis NBRC 102666
Nitrospira moscoviensis
76G-FAM007853
Opitutae
Flavobacteriia
Chitinophagia
Halobacteria
Stenosarchaea group
Streptosporangiales
Planctomycetia
Nitrospirales
Epsilonproteobacteria
Nitrosopumilales
Nitrososphaerales
Nitrosocaldales
Deinococci
Caldilineae
Oscillatoriophycideae
Pleurocapsales
Nostocales
Synechococcales
Gammaproteobacteria
Betaproteobacteria
Alphaproteobacteria
Deltaproteobacteria
Streptophyta
Pyrenomonadales
Phycisphaerae
Verrucomicrobia
Bacteroidetes
Euryarchaeota
Actinobacteria
Planctomycetes
Nitrospirae
Proteobacteria
Thaumarchaeota
Deinococcus-Thermus
Chloroflexi
Cyanobacteria
Viridiplantae
Cryptophyta
Bacteria
Archaea
Eukaryota

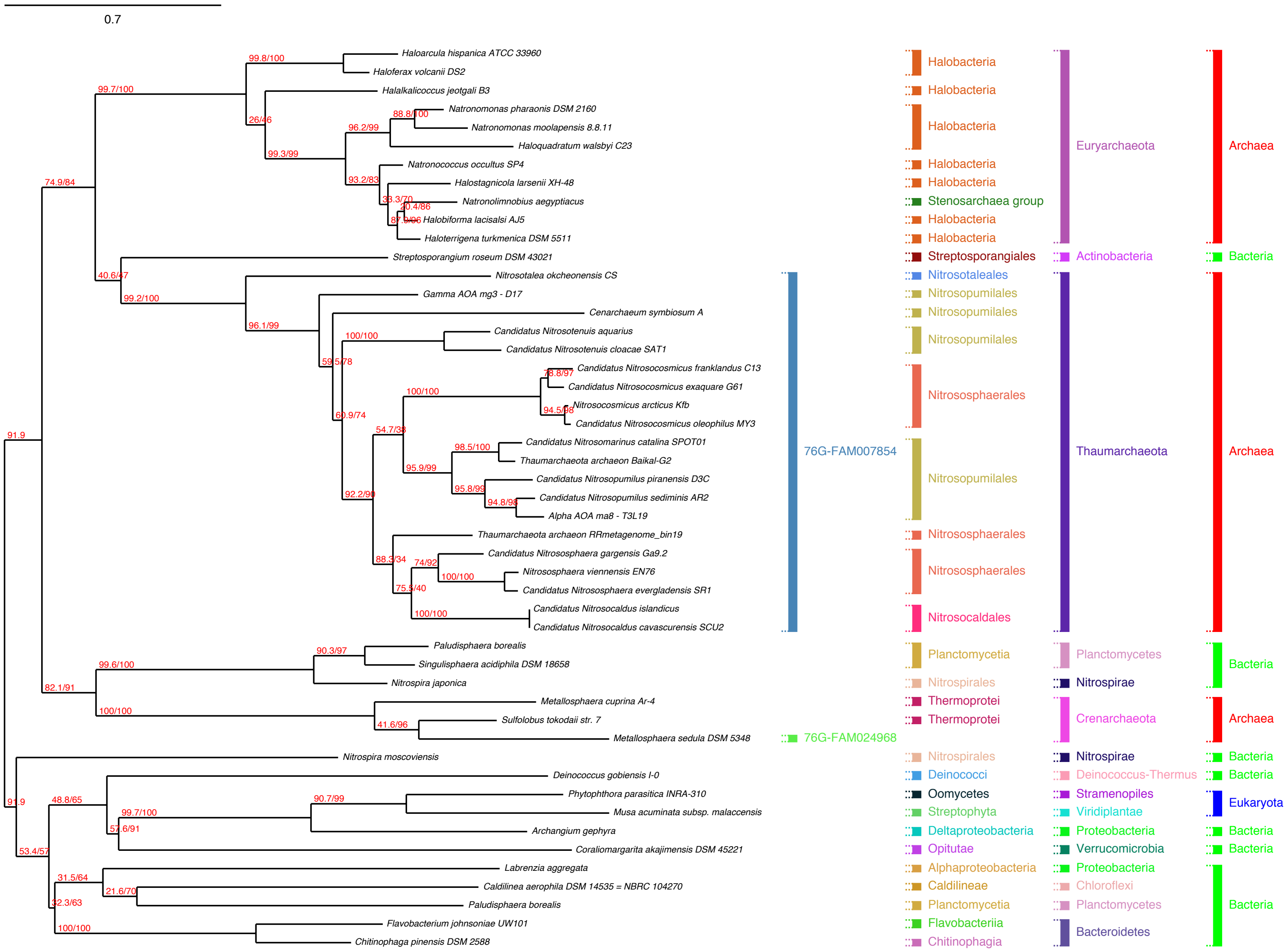

0.7
Haloarcula hispanica ATCC 33960
Haloferax volcanii DS2
Halalkalicoccus jeotgali B3
Natronomonas pharaonis DSM 2160
Natronomonas moolapensis 8.8.11
Haloquadratum walsbyi C23
Natronococcus occultus SP4
Halostagnicola larsenii XH-48
Natronolimnobius aegyptiacus
Halobiforma lacisalsi AJ5
Haloterrigena turkmenica DSM 5511
Streptosporangium roseum DSM 43021
Nitrosotalea okcheonensis CS
Gamma AOA mg3 - D17
Cenarchaeum symbiosum A
Candidatus Nitrosotenuis aquarius
Candidatus Nitrosotenuis cloacae SAT1
Candidatus Nitrosocosmicus franklandus C13
Candidatus Nitrosocosmicus exaquare G61
Nitrosocosmicus arcticus Kfb
Candidatus Nitrosocosmicus oleophilus MY3
Candidatus Nitrosomarinus catalina SPOT01
Thaumarchaeota archaeon Baikal-G2
Candidatus Nitrosopumilus piranensis D3C
Candidatus Nitrosopumilus sediminis AR2
Alpha AOA ma8 - T3L19
Thaumarchaeota archaeon RRmetagenome_bin19
Candidatus Nitrososphaera gargensis Ga9.2
Nitrososphaera viennensis EN76
Candidatus Nitrososphaera evergladensis SR1
Candidatus Nitrosocaldus islandicus
Candidatus Nitrosocaldus cavascurensis SCU2
Paludisphaera borealis
Singulisphaera acidiphila DSM 18658
Nitrospira japonica
Metallosphaera cuprina Ar-4
Sulfolobus tokodaii str. 7
Metallosphaera sedula DSM 5348
Nitrospira moscoviensis
Deinococcus gobiensis I-0
Phytophthora parasitica INRA-310
Musa acuminata subsp. malaccensis
Archangium gephyra
Coraliomargarita akajimensis DSM 45221
Labrenzia aggregata
Caldilinea aerophila DSM 14535 = NBRC 104270
Paludisphaera borealis
Flavobacterium johnsoniae UW101
Chitinophaga pinensis DSM 2588
99.8/100
99.7/100
26/46
88.8/100
96.2/99
99.3/99
93.2/83
33.3/70
20.4/86
87.9/96
74.9/84
40.6/47
99.2/100
96.1/99
100/100
59.5/78
78.8/97
100/100
94.5/98
60.9/74
54.7/38
98.5/100
95.9/99
95.8/99
94.8/98
92.2/90
88.3/34
74/92
100/100
75.5/40
100/100
91.9
90.3/97
99.6/100
82.1/91
100/100
41.6/96
91.9
48.8/65
90.7/99
99.7/100
57.6/91
53.4/57
31.5/64
21.6/70
32.3/63
100/100
76G-FAM007854
76G-FAM024968
Halobacteria
Halobacteria
Halobacteria
Halobacteria
Halobacteria
Stenosarchaea group
Halobacteria
Halobacteria
Streptosporangiales
Nitrosotaleales
Nitrosopumilales
Nitrosopumilales
Nitrosopumilales
Nitrososphaerales
Nitrosopumilales
Nitrososphaerales
Nitrososphaerales
Nitrosocaldales
Planctomycetia
Nitrospirales
Thermoprotei
Thermoprotei
Nitrospirales
Deinococci
Oomycetes
Streptophyta
Deltaproteobacteria
Opitutae
Alphaproteobacteria
Caldilineae
Planctomycetia
Flavobacteriia
Chitinophagia
Euryarchaeota
Actinobacteria
Thaumarchaeota
Planctomycetes
Nitrospirae
Crenarchaeota
Nitrospirae
Deinococcus-Thermus
Stramenopiles
Viridiplantae
Proteobacteria
Verrucomicrobia
Proteobacteria
Chloroflexi
Planctomycetes
Bacteroidetes
Archaea
Bacteria
Archaea
Bacteria
Archaea
Bacteria
Bacteria
Eukaryota
Bacteria
Bacteria
Bacteria

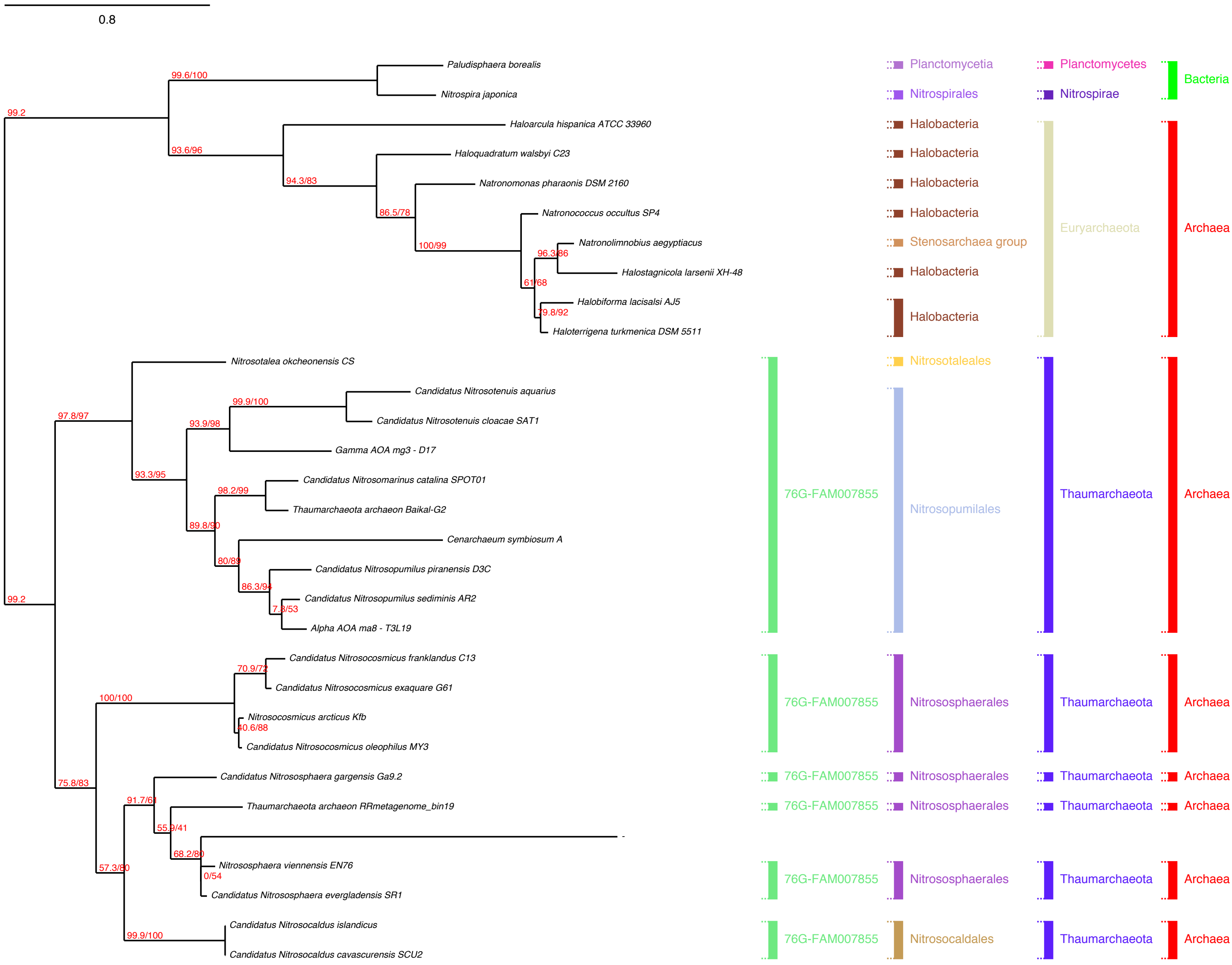
0.8
99.2
99.6/100
93.6/96
94.3/83
86.5/78
100/99
96.3/86
61/68
79.8/92
97.8/97
99.9/100
93.9/98
93.3/95
98.2/99
89.8/90
80/89
86.3/94
7.8/53
99.2
100/100
70.9/72
40.6/88
75.8/83
91.7/61
55.9/41
68.2/80
0/54
57.3/80
99.9/100
Paludisphaera borealis
Nitrospira japonica
Haloarcula hispanica ATCC 33960
Haloquadratum walsbyi C23
Natronomonas pharaonis DSM 2160
Natronococcus occultus SP4
Natronolimnobius aegyptiacus
Halostagnicola larsenii XH-48
Halobiforma lacisalsi AJ5
Haloterrigena turkmenica DSM 5511
Nitrosotalea okcheonensis CS
Candidatus Nitrosotenuis aquarius
Candidatus Nitrosotenuis cloacae SAT1
Gamma AOA mg3 - D17
Candidatus Nitrosomarinus catalina SPOT01
Thaumarchaeota archaeon Baikal-G2
Cenarchaeum symbiosum A
Candidatus Nitrosopumilus piranensis D3C
Candidatus Nitrosopumilus sediminis AR2
Alpha AOA ma8 - T3L19
Candidatus Nitrosocosmicus franklandus C13
Candidatus Nitrosocosmicus exaquare G61
Nitrosocosmicus arcticus Kfb
Candidatus Nitrosocosmicus oleophilus MY3
Candidatus Nitrososphaera gargensis Ga9.2
Thaumarchaeota archaeon RRmetagenome_bin19
-
Nitrososphaera viennensis EN76
Candidatus Nitrososphaera evergladensis SR1
Candidatus Nitrosocaldus islandicus
Candidatus Nitrosocaldus cavascurensis SCU2
Planctomycetia
Nitrospirales
Halobacteria
Halobacteria
Halobacteria
Halobacteria
Stenosarchaea group
Halobacteria
Halobacteria
Nitrosotaleales
Nitrosopumilales
Nitrososphaerales
Nitrososphaerales
Nitrososphaerales
Nitrososphaerales
Nitrosocaldales
Planctomycetes
Nitrospirae
Euryarchaeota
Thaumarchaeota
Thaumarchaeota
Thaumarchaeota
Thaumarchaeota
Thaumarchaeota
Thaumarchaeota
Bacteria
Archaea
Archaea
Archaea
Archaea
Archaea
Archaea
Archaea
76G-FAM007855
76G-FAM007855
76G-FAM007855
76G-FAM007855
76G-FAM007855
76G-FAM007855

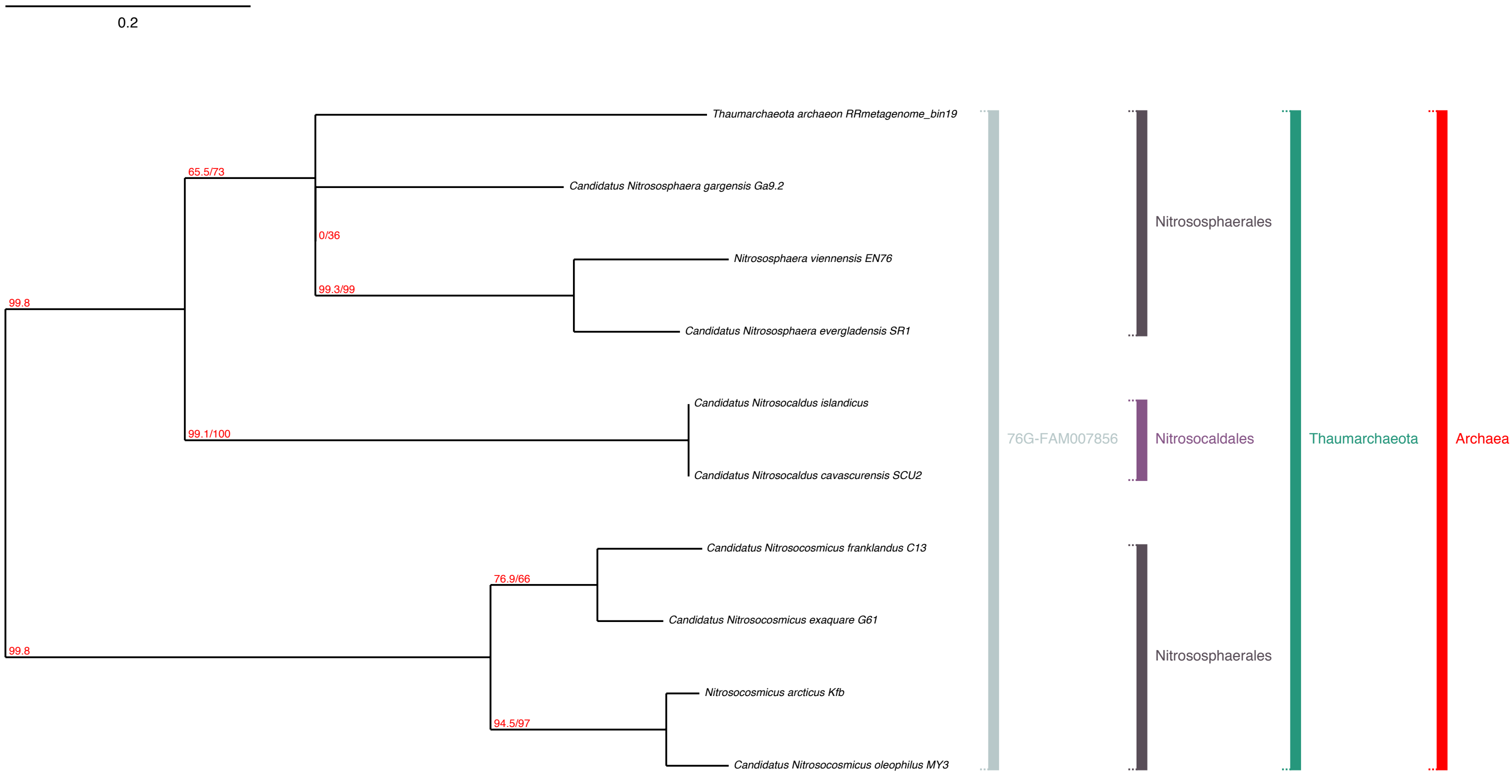
0.2
Thaumarchaeota archaeon RRmetagenome_bin19
65.5/73
Candidatus Nitrososphaera gargensis Ga9.2
0/36
Nitrososphaera viennensis EN76
99.3/99
99.8
Candidatus Nitrososphaera evergladensis SR1
Candidatus Nitrosocaldus islandicus
99.1/100
Candidatus Nitrosocaldus cavascurensis SCU2
Candidatus Nitrosocosmicus franklandus C13
76.9/66
Candidatus Nitrosocosmicus exaquare G61
99.8
Nitrosocosmicus arcticus Kfb
94.5/97
Candidatus Nitrosocosmicus oleophilus MY3
76G-FAM007856
Nitrososphaerales
Nitrosocaldales
Nitrososphaerales
Thaumarchaeota
Archaea

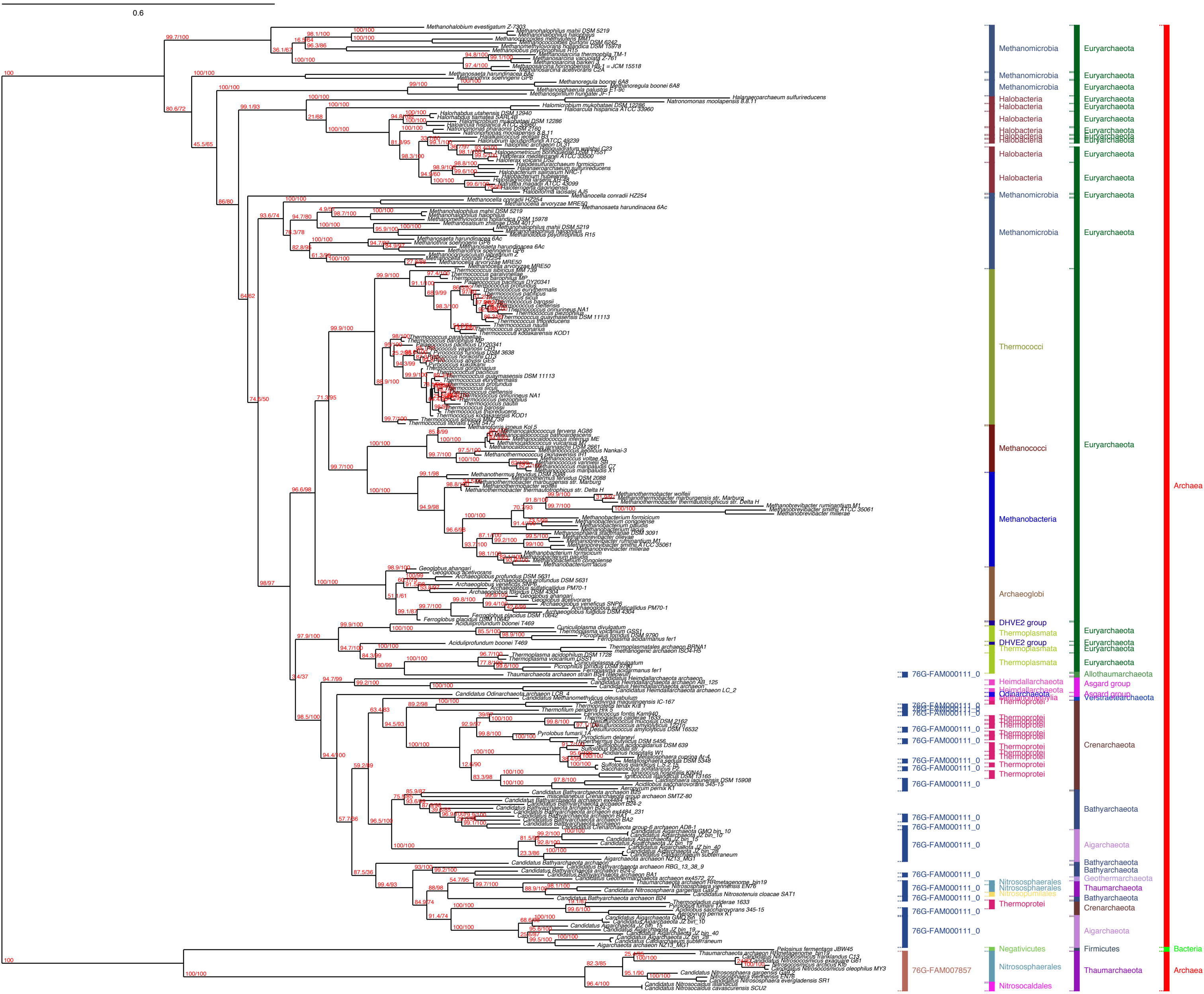
0.6
Methanomicrobia
Halobacteria
Thermococci
Methanococci
Methanobacteria
Archaeoglobi
DHVE2 group
Thermoplasmata
Heimdallarchaeota
Odinarchaeota
Methanomethylicia
Thermoprotei
Nitrososphaerales
Nitrosopumilales
Negativicutes
Nitrosocaldales
76G-FAM000111_0
76G-FAM007857
Euryarchaeota
Allothaumarchaeota
Asgard group
Verstraetearchaeota
Crenarchaeota
Bathyarchaeota
Aigarchaeota
Geothermarchaeota
Thaumarchaeota
Firmicutes
Archaea
Bacteria

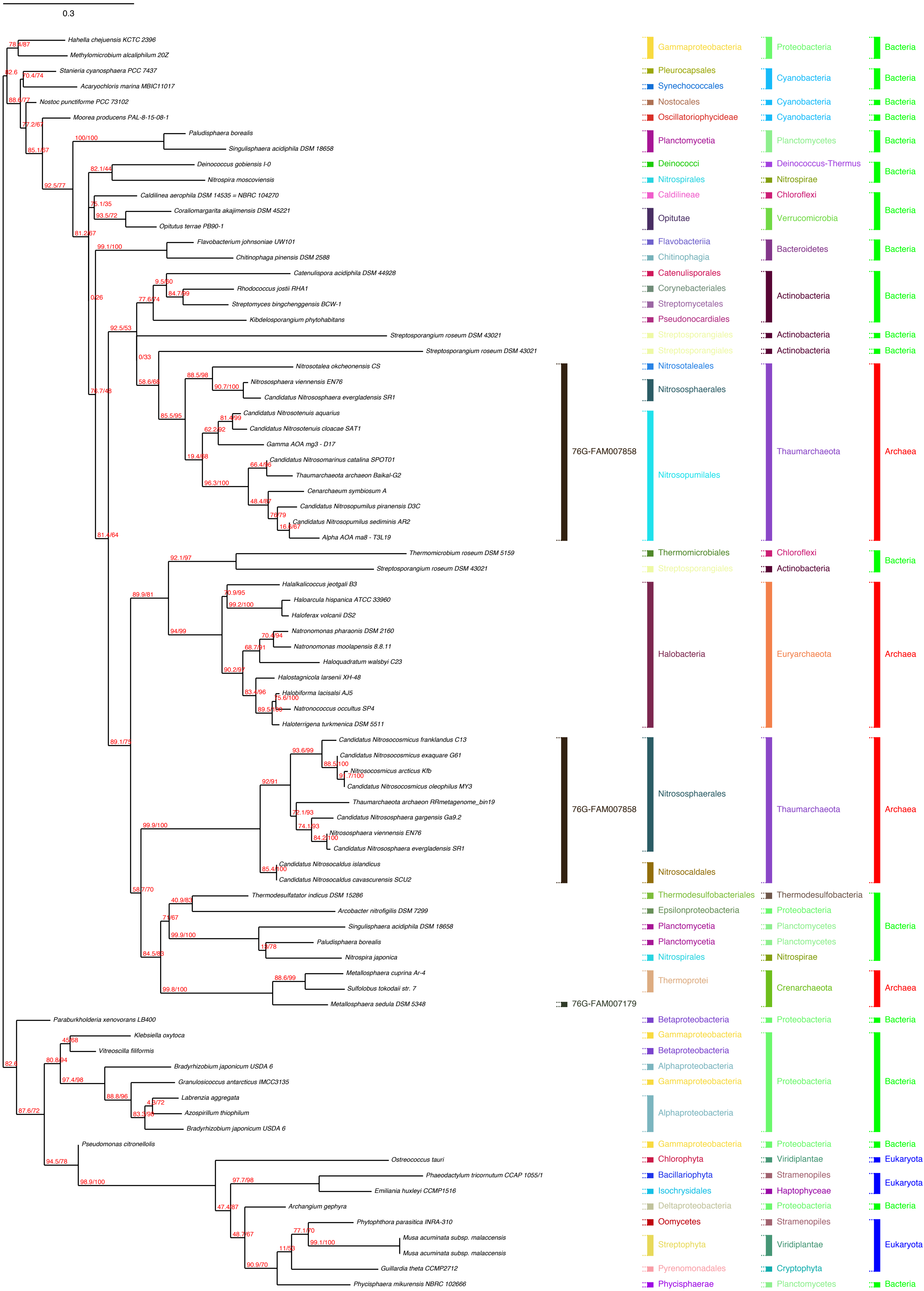
0.3
Hahella chejuensis KCTC 2396
Methylomicrobium alcaliphilum 20Z
Stanieria cyanosphaera PCC 7437
Acaryochloris marina MBIC11017
Nostoc punctiforme PCC 73102
Moorea producens PAL-8-15-08-1
Paludisphaera borealis
Singulisphaera acidiphila DSM 18658
Deinococcus gobiensis I-0
Nitrospira moscoviensis
Caldilinea aerophila DSM 14535 = NBRC 104270
Coraliomargarita akajimensis DSM 45221
Opitutus terrae PB90-1
Flavobacterium johnsoniae UW101
Chitinophaga pinensis DSM 2588
Catenulispora acidiphila DSM 44928
Rhodococcus jostii RHA1
Streptomyces bingchenggensis BCW-1
Kibdelosporangium phytohabitans
Streptosporangium roseum DSM 43021
Streptosporangium roseum DSM 43021
Nitrosotalea okcheonensis CS
Nitrososphaera viennensis EN76
Candidatus Nitrososphaera evergladensis SR1
Candidatus Nitrosotenuis aquarius
Candidatus Nitrosotenuis cloacae SAT1
Gamma AOA mg3 - D17
Candidatus Nitrosomarinus catalina SPOT01
Thaumarchaeota archaeon Baikal-G2
Cenarchaeum symbiosum A
Candidatus Nitrosopumilus piranensis D3C
Candidatus Nitrosopumilus sediminis AR2
Alpha AOA ma8 - T3L19
Thermomicrobium roseum DSM 5159
Streptosporangium roseum DSM 43021
Halalkalicoccus jeotgali B3
Haloarcula hispanica ATCC 33960
Haloferax volcanii DS2
Natronomonas pharaonis DSM 2160
Natronomonas moolapensis 8.8.11
Haloquadratum walsbyi C23
Halostagnicola larsenii XH-48
Halobiforma lacisalsi AJ5
Natronococcus occultus SP4
Haloterrigena turkmenica DSM 5511
Candidatus Nitrosocosmicus franklandus C13
Candidatus Nitrosocosmicus exaquare G61
Nitrosocosmicus arcticus Kfb
Candidatus Nitrosocosmicus oleophilus MY3
Thaumarchaeota archaeon RRmetagenome_bin19
Candidatus Nitrososphaera gargensis Ga9.2
Nitrososphaera viennensis EN76
Candidatus Nitrososphaera evergladensis SR1
Candidatus Nitrosocaldus islandicus
Candidatus Nitrosocaldus cavascurensis SCU2
Thermodesulfatator indicus DSM 15286
Arcobacter nitrofigilis DSM 7299
Singulisphaera acidiphila DSM 18658
Paludisphaera borealis
Nitrospira japonica
Metallosphaera cuprina Ar-4
Sulfolobus tokodaii str. 7
Metallosphaera sedula DSM 5348
Paraburkholderia xenovorans LB400
Klebsiella oxytoca
Vitreoscilla filiformis
Bradyrhizobium japonicum USDA 6
Granulosicoccus antarcticus IMCC3135
Labrenzia aggregata
Azospirillum thiophilum
Bradyrhizobium japonicum USDA 6
Pseudomonas citronellolis
Ostreococcus tauri
Phaeodactylum tricornutum CCAP 1055/1
Emiliania huxleyi CCMP1516
Archangium gephyra
Phytophthora parasitica INRA-310
Musa acuminata subsp. malaccensis
Musa acuminata subsp. malaccensis
Guillardia theta CCMP2712
Phycisphaera mikurensis NBRC 102666
76G-FAM007858
76G-FAM007858
76G-FAM007179
Gammaproteobacteria
Pleurocapsales
Synechococcales
Nostocales
Oscillatoriophycideae
Planctomycetia
Deinococci
Nitrospirales
Caldilineae
Opitutae
Flavobacteriia
Chitinophagia
Catenulisporales
Corynebacteriales
Streptomycetales
Pseudonocardiales
Streptosporangiales
Streptosporangiales
Nitrosotaleales
Nitrososphaerales
Nitrosopumilales
Thermomicrobiales
Streptosporangiales
Halobacteria
Nitrososphaerales
Nitrosocaldales
Thermodesulfobacteriales
Epsilonproteobacteria
Planctomycetia
Planctomycetia
Nitrospirales
Thermoprotei
Betaproteobacteria
Gammaproteobacteria
Betaproteobacteria
Alphaproteobacteria
Gammaproteobacteria
Alphaproteobacteria
Gammaproteobacteria
Chlorophyta
Bacillariophyta
Isochrysidales
Deltaproteobacteria
Oomycetes
Streptophyta
Pyrenomonadales
Phycisphaerae
Proteobacteria
Cyanobacteria
Cyanobacteria
Cyanobacteria
Planctomycetes
Deinococcus-Thermus
Nitrospirae
Chloroflexi
Verrucomicrobia
Bacteroidetes
Actinobacteria
Actinobacteria
Actinobacteria
Thaumarchaeota
Chloroflexi
Actinobacteria
Euryarchaeota
Thaumarchaeota
Thermodesulfobacteria
Proteobacteria
Planctomycetes
Planctomycetes
Nitrospirae
Crenarchaeota
Proteobacteria
Proteobacteria
Proteobacteria
Viridiplantae
Stramenopiles
Haptophyceae
Proteobacteria
Stramenopiles
Viridiplantae
Cryptophyta
Planctomycetes
Bacteria
Archaea
Eukaryota

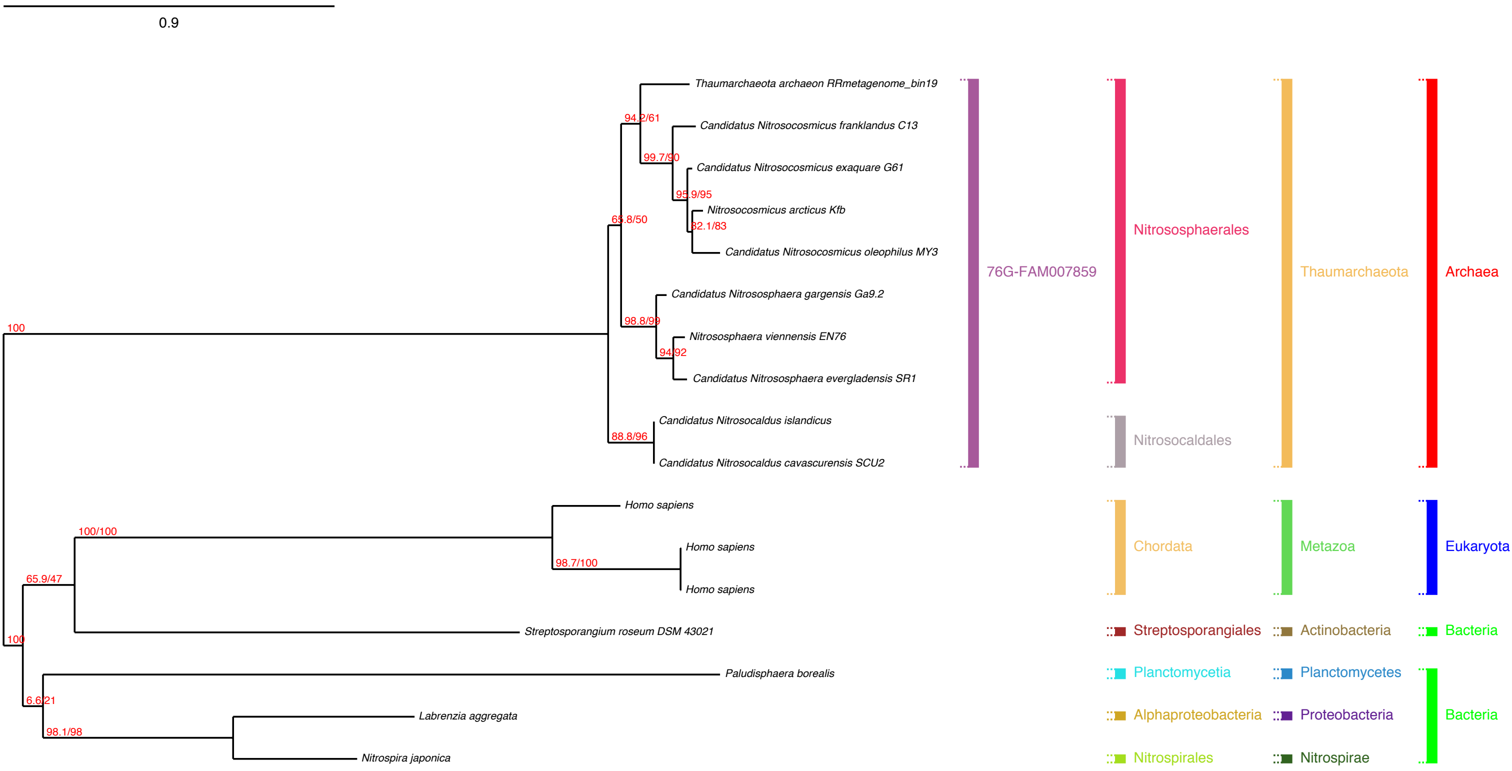

0.9
Thaumarchaeota archaeon RRmetagenome_bin19
Candidatus Nitrosocosmicus franklandus C13
Candidatus Nitrosocosmicus exaquare G61
Nitrosocosmicus arcticus Kfb
Candidatus Nitrosocosmicus oleophilus MY3
Candidatus Nitrososphaera gargensis Ga9.2
Nitrososphaera viennensis EN76
Candidatus Nitrososphaera evergladensis SR1
Candidatus Nitrosocaldus islandicus
Candidatus Nitrosocaldus cavascurensis SCU2
Homo sapiens
Homo sapiens
Homo sapiens
Streptosporangium roseum DSM 43021
Paludisphaera borealis
Labrenzia aggregata
Nitrospira japonica
100
94.2/61
99.7/90
95.9/95
82.1/83
65.8/50
98.8/99
94/92
88.8/96
100/100
98.7/100
65.9/47
100
6.6/21
98.1/98
76G-FAM007859
Nitrososphaerales
Nitrosocaldales
Chordata
Streptosporangiales
Planctomycetia
Alphaproteobacteria
Nitrospirales
Thaumarchaeota
Metazoa
Actinobacteria
Planctomycetes
Proteobacteria
Nitrospirae
Archaea
Eukaryota
Bacteria
Bacteria

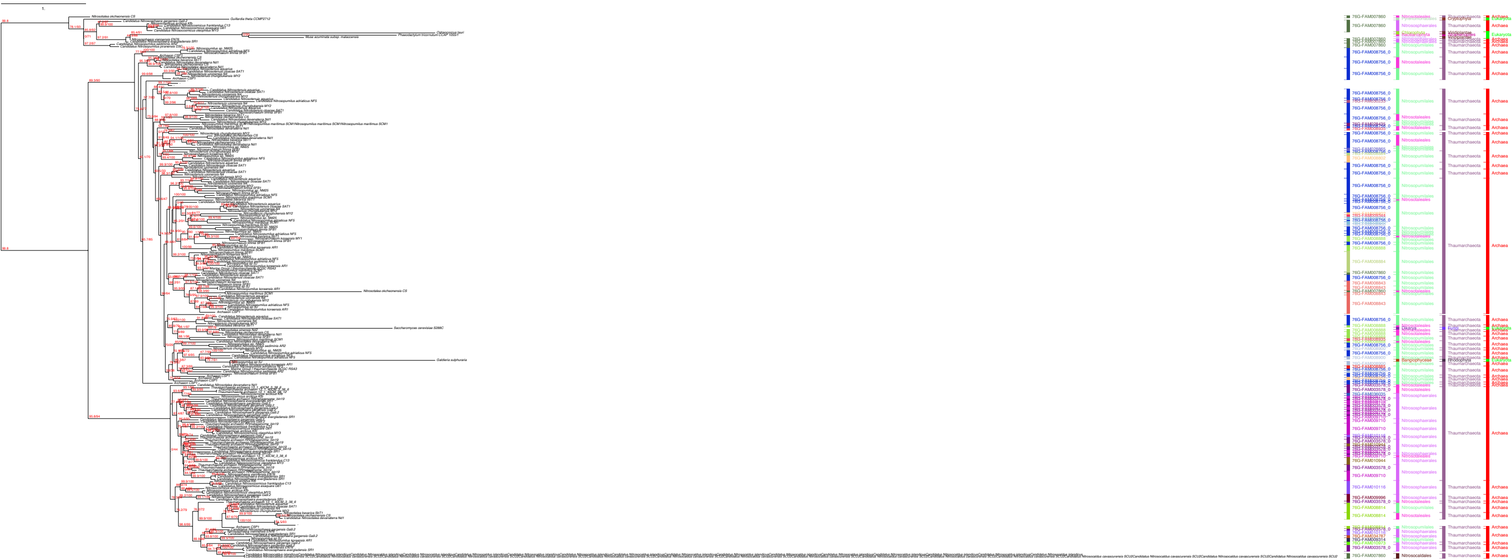

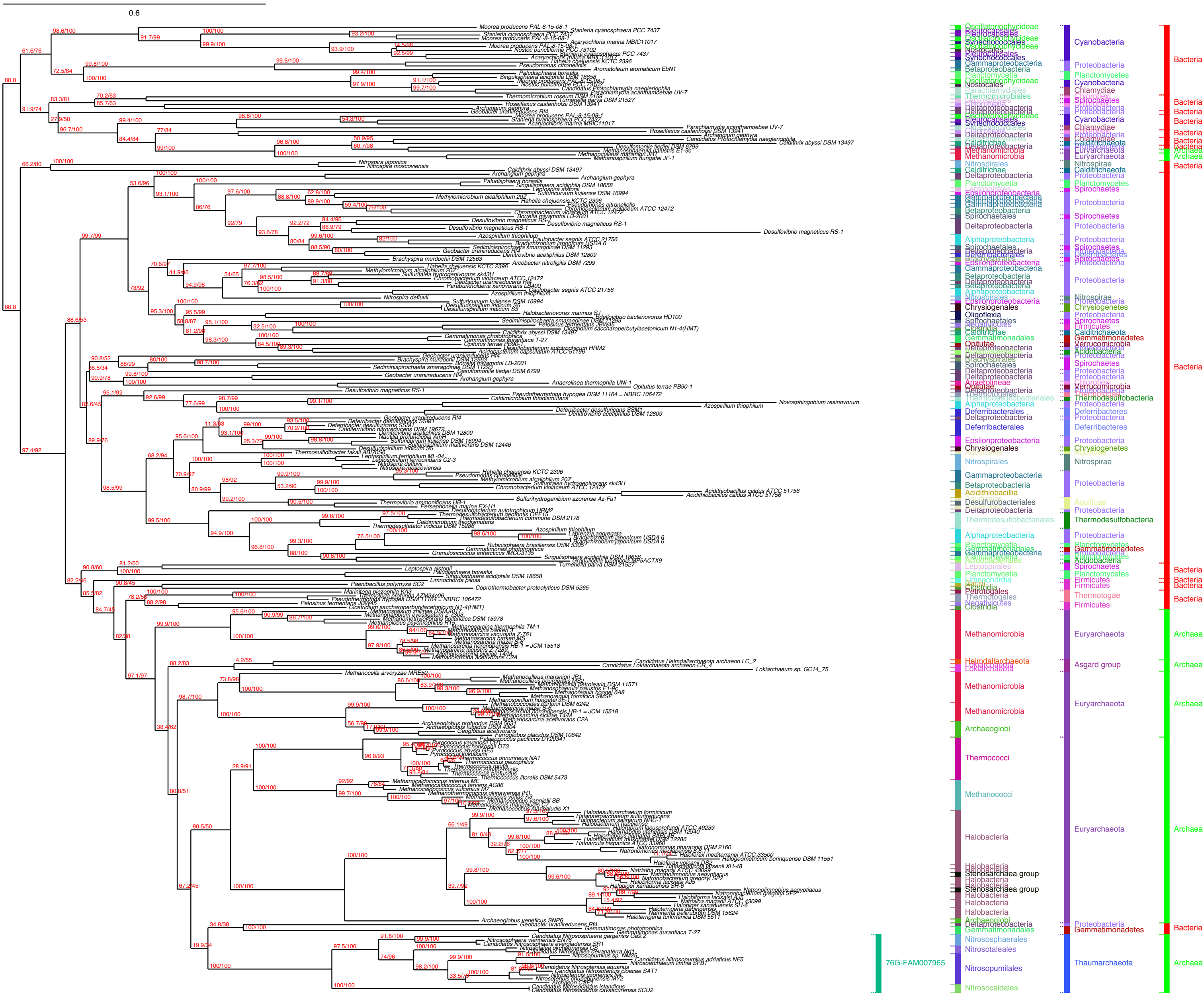

0.6
Cyanobacteria
Proteobacteria
Planctomycetes
Chlamydiae
Spirochaetes
Nitrospirae
Caldithrichaeota
Chrysiogenetes
Firmicutes
Gemmatimonadetes
Verrucomicrobia
Acidobacteria
Thermodesulfobacteria
Deferribacteres
Aquificae
Thermotogae
Euryarchaeota
Asgard group
Thaumarchaeota
76G-FAM007965
Bacteria
Archaea
Methanomicrobia
Heimdallarchaeota
Lokiarchaeota
Archaeoglobi
Thermococci
Methanococci
Halobacteria
Stenosarchaea group
Nitrososphaerales
Nitrosotaleales
Nitrosopumilales
Nitrosocaldales

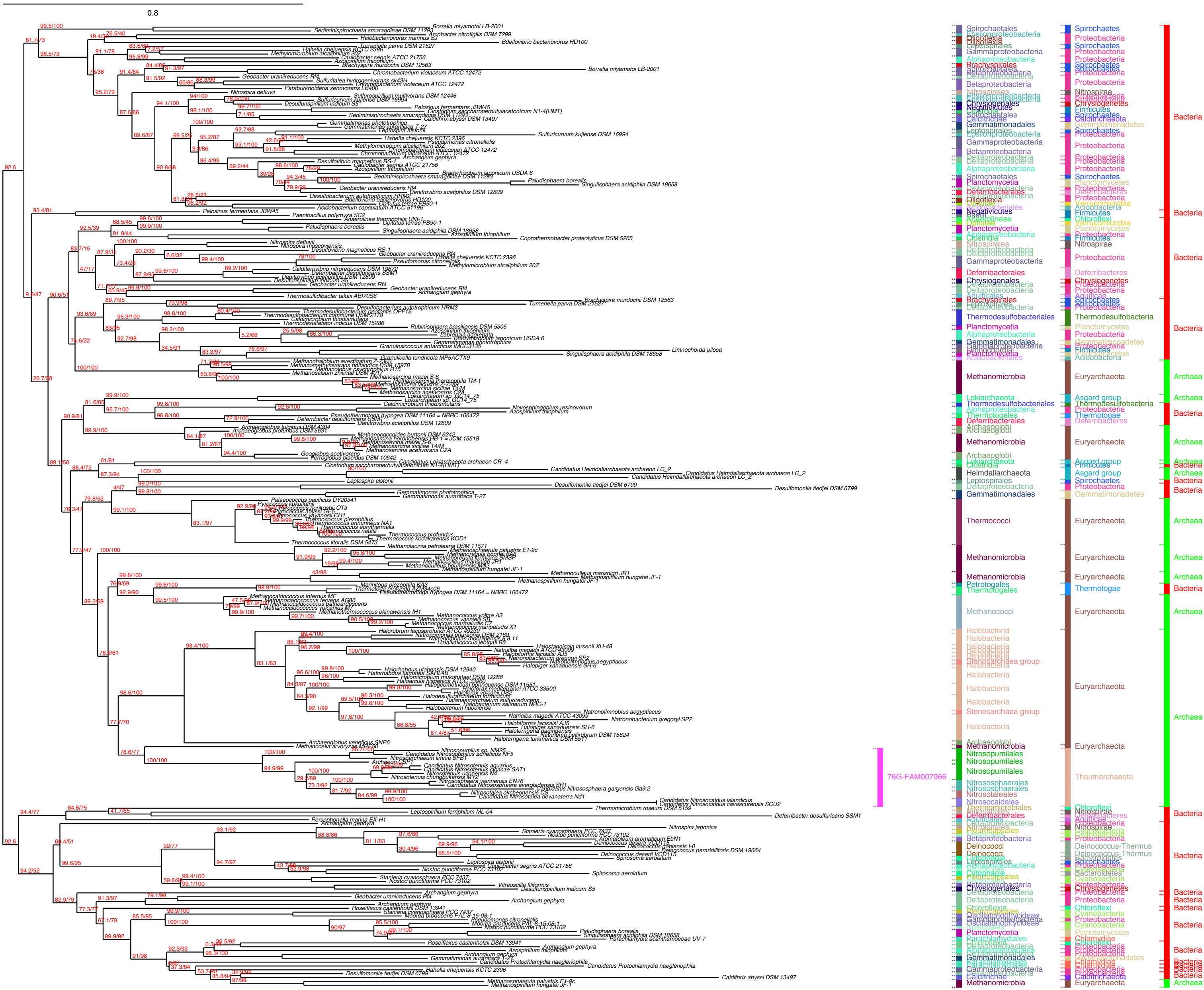

0.8
76G-FAM007966
Bacteria
Archaea
Spirochaetes
Proteobacteria
Nitrospirae
Firmicutes
Chloroflexi
Thermodesulfobacteria
Euryarchaeota
Asgard group
Thermotogae
Thaumarchaeota
Methanomicrobia
Thermococci
Methanococci
Halobacteria
Stenosarchaea group
Nitrosopumilales
Nitrososphaerales
Nitrosotaleales
Nitrosocaldales
Gammaproteobacteria
Betaproteobacteria
Alphaproteobacteria
Deltaproteobacteria
Deferribacterales
Chrysiogenetes
Gemmatimonadales
Lokiarchaeota
Heimdallarchaeota
Archaeoglobi
Petrotogales
Thermotogales
Deinococci
Deinococcus-Thermus
Bacteroidetes
Cyanobacteria
Planctomycetia
Chlamydiae
Caldithrichaeota

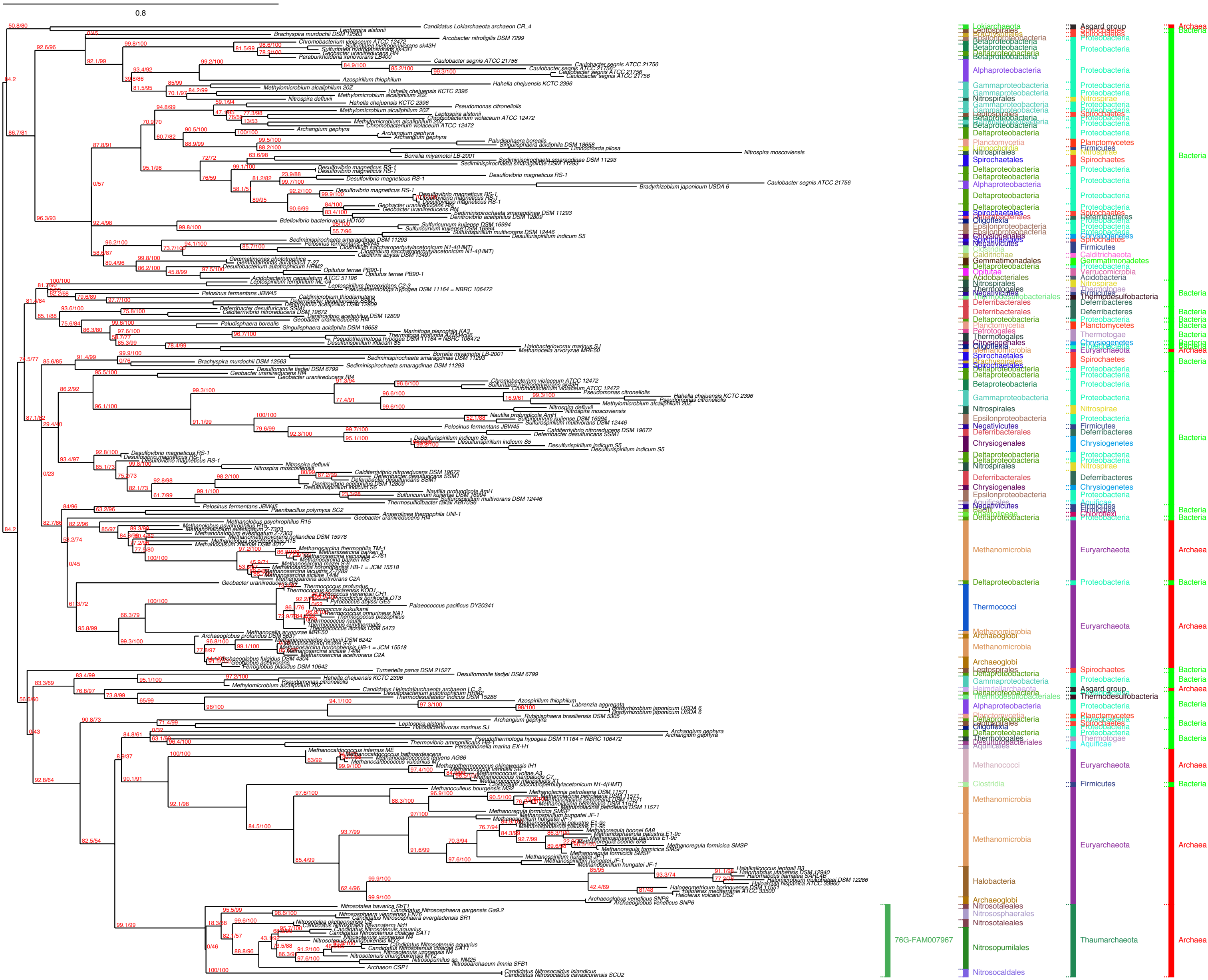
0.8
76G-FAM007967
Bacteria
Archaea
Asgard group
Proteobacteria
Euryarchaeota
Thaumarchaeota
Methanomicrobia
Thermococci
Archaeoglobi
Halobacteria
Methanococci
Nitrosotaleales
Nitrososphaerales
Nitrosopumilales
Nitrosocaldales

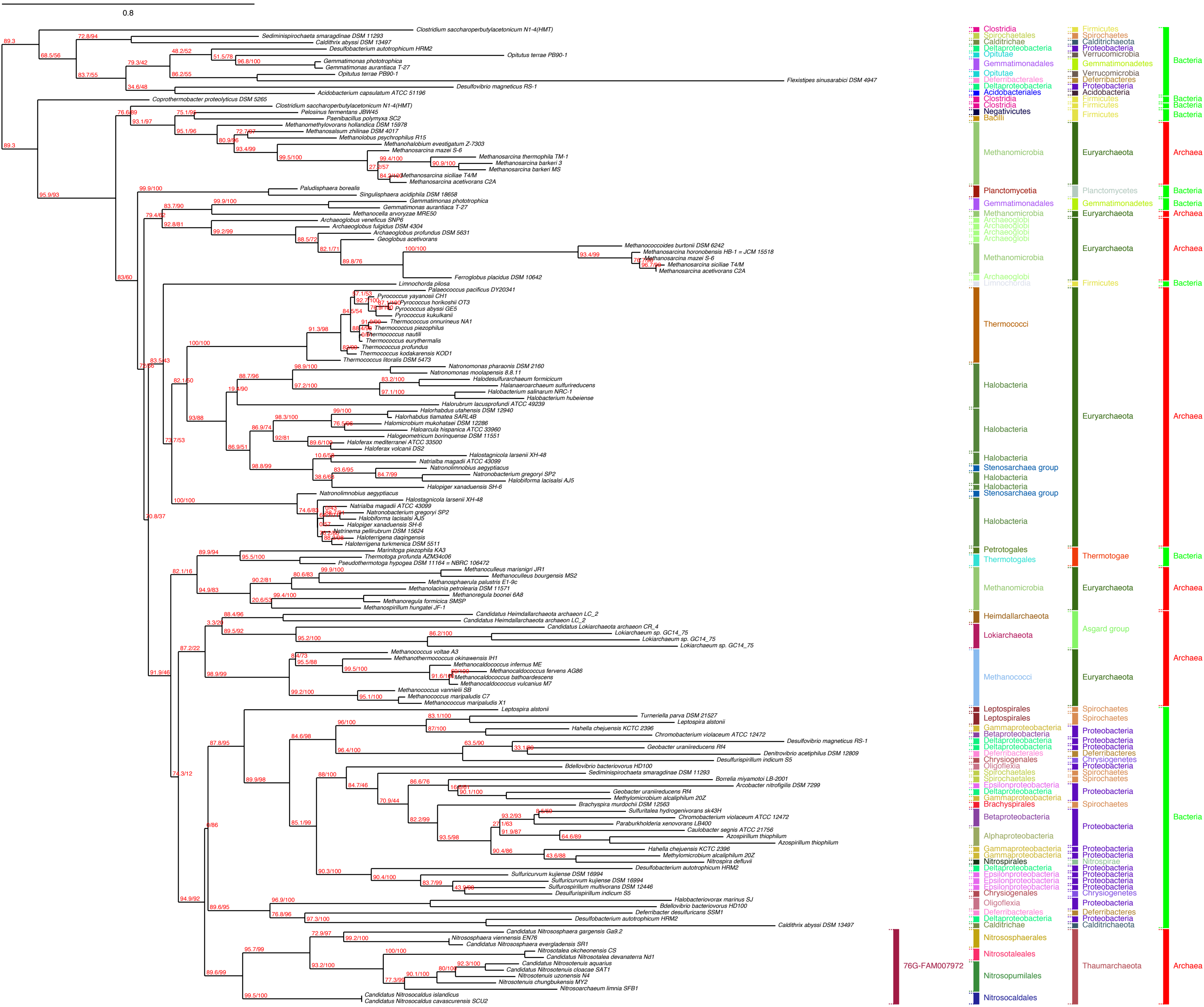

0.8
Clostridium saccharoperbutylacetonicum N1-4(HMT)
Sediminispirochaeta smaragdinae DSM 11293
Caldithrix abyssi DSM 13497
Desulfobacterium autotrophicum HRM2
Opitutus terrae PB90-1
Gemmatimonas phototrophica
Gemmatimonas aurantiaca T-27
Opitutus terrae PB90-1
Flexistipes sinusarabici DSM 4947
Desulfovibrio magneticus RS-1
Acidobacterium capsulatum ATCC 51196
Coprothermobacter proteolyticus DSM 5265
Clostridium saccharoperbutylacetonicum N1-4(HMT)
Pelosinus fermentans JBW45
Paenibacillus polymyxa SC2
Methanomethylovorans hollandica DSM 15978
Methanosalsum zhilinae DSM 4017
Methanolobus psychrophilus R15
Methanohalobium evestigatum Z-7303
Methanosarcina mazei S-6
Methanosarcina thermophila TM-1
Methanosarcina barkeri 3
Methanosarcina barkeri MS
Methanosarcina siciliae T4/M
Methanosarcina acetivorans C2A
Paludisphaera borealis
Singulisphaera acidiphila DSM 18658
Gemmatimonas phototrophica
Gemmatimonas aurantiaca T-27
Methanocella arvoryzae MRE50
Archaeoglobus veneficus SNP6
Archaeoglobus fulgidus DSM 4304
Archaeoglobus profundus DSM 5631
Geoglobus acetivorans
Methanococcoides burtonii DSM 6242
Methanosarcina horonobensis HB-1 = JCM 15518
Methanosarcina mazei S-6
Methanosarcina siciliae T4/M
Methanosarcina acetivorans C2A
Ferroglobus placidus DSM 10642
Limnochorda pilosa
Palaeococcus pacificus DY20341
Pyrococcus yayanosii CH1
Pyrococcus horikoshii OT3
Pyrococcus abyssi GE5
Pyrococcus kukulkanii
Thermococcus onnurineus NA1
Thermococcus piezophilus
Thermococcus nautili
Thermococcus eurythermalis
Thermococcus profundus
Thermococcus kodakarensis KOD1
Thermococcus litoralis DSM 5473
Natronomonas pharaonis DSM 2160
Natronomonas moolapensis 8.8.11
Halodesulfurarchaeum formicicum
Halanaeroarchaeum sulfurireducens
Halobacterium salinarum NRC-1
Halobacterium hubeiense
Halorubrum lacusprofundi ATCC 49239
Halorhabdus utahensis DSM 12940
Halorhabdus tiamatea SARL4B
Halomicrobium mukohataei DSM 12286
Haloarcula hispanica ATCC 33960
Halogeometricum borinquense DSM 11551
Haloferax mediterranei ATCC 33500
Haloferax volcanii DS2
Halostagnicola larsenii XH-48
Natrialba magadii ATCC 43099
Natronolimnobius aegyptiacus
Natronobacterium gregoryi SP2
Halobiforma lacisalsi AJ5
Halopiger xanaduensis SH-6
Natronolimnobius aegyptiacus
Halostagnicola larsenii XH-48
Natrialba magadii ATCC 43099
Natronobacterium gregoryi SP2
Halobiforma lacisalsi AJ5
Halopiger xanaduensis SH-6
Natrinema pellirubrum DSM 15624
Haloterrigena daqingensis
Haloterrigena turkmenica DSM 5511
Marinitoga piezophila KA3
Thermotoga profunda AZM34c06
Pseudothermotoga hypogea DSM 11164 = NBRC 106472
Methanoculleus marisnigri JR1
Methanoculleus bourgensis MS2
Methanosphaerula palustris E1-9c
Methanolacinia petrolearia DSM 11571
Methanoregula boonei 6A8
Methanoregula formicica SMSP
Methanospirillum hungatei JF-1
Candidatus Heimdallarchaeota archaeon LC_2
Candidatus Heimdallarchaeota archaeon LC_2
Candidatus Lokiarchaeota archaeon CR_4
Lokiarchaeum sp. GC14_75
Lokiarchaeum sp. GC14_75
Lokiarchaeum sp. GC14_75
Methanococcus voltae A3
Methanothermococcus okinawensis IH1
Methanocaldococcus infernus ME
Methanocaldococcus fervens AG86
Methanocaldococcus bathoardescens
Methanocaldococcus vulcanius M7
Methanococcus vannielii SB
Methanococcus maripaludis C7
Methanococcus maripaludis X1
Leptospira alstonii
Turneriella parva DSM 21527
Leptospira alstonii
Hahella chejuensis KCTC 2396
Chromobacterium violaceum ATCC 12472
Desulfovibrio magneticus RS-1
Geobacter uraniireducens Rf4
Denitrovibrio acetiphilus DSM 12809
Desulfurispirillum indicum S5
Bdellovibrio bacteriovorus HD100
Sediminispirochaeta smaragdinae DSM 11293
Borrelia miyamotoi LB-2001
Arcobacter nitrofigilis DSM 7299
Geobacter uraniireducens Rf4
Methylomicrobium alcaliphilum 20Z
Brachyspira murdochii DSM 12563
Sulfuritalea hydrogenivorans sk43H
Chromobacterium violaceum ATCC 12472
Paraburkholderia xenovorans LB400
Caulobacter segnis ATCC 21756
Azospirillum thiophilum
Azospirillum thiophilum
Hahella chejuensis KCTC 2396
Methylomicrobium alcaliphilum 20Z
Nitrospira defluvii
Desulfobacterium autotrophicum HRM2
Sulfuricurvum kujiense DSM 16994
Sulfuricurvum kujiense DSM 16994
Sulfurospirillum multivorans DSM 12446
Desulfurispirillum indicum S5
Halobacteriovorax marinus SJ
Bdellovibrio bacteriovorus HD100
Deferribacter desulfuricans SSM1
Desulfobacterium autotrophicum HRM2
Caldithrix abyssi DSM 13497
Candidatus Nitrososphaera gargensis Ga9.2
Nitrososphaera viennensis EN76
Candidatus Nitrososphaera evergladensis SR1
Nitrosotalea okcheonensis CS
Candidatus Nitrosotalea devanaterra Nd1
Candidatus Nitrosotenuis aquarius
Candidatus Nitrosotenuis cloacae SAT1
Nitrosotenuis uzonensis N4
Nitrosotenuis chungbukensis MY2
Nitrosoarchaeum limnia SFB1
Candidatus Nitrosocaldus islandicus
Candidatus Nitrosocaldus cavascurensis SCU2
76G-FAM007972
Clostridia
Spirochaetales
Caldithrichae
Deltaproteobacteria
Opitutae
Gemmatimonadales
Opitutae
Deferribacterales
Deltaproteobacteria
Acidobacteriales
Clostridia
Clostridia
Negativicutes
Bacilli
Methanomicrobia
Planctomycetia
Gemmatimonadales
Methanomicrobia
Archaeoglobi
Archaeoglobi
Archaeoglobi
Archaeoglobi
Methanomicrobia
Archaeoglobi
Limnochordia
Thermococci
Halobacteria
Halobacteria
Halobacteria
Stenosarchaea group
Halobacteria
Halobacteria
Stenosarchaea group
Halobacteria
Petrotogales
Thermotogales
Methanomicrobia
Heimdallarchaeota
Lokiarchaeota
Methanococci
Leptospirales
Leptospirales
Gammaproteobacteria
Betaproteobacteria
Deltaproteobacteria
Deltaproteobacteria
Deferribacterales
Chrysiogenales
Oligoflexia
Spirochaetales
Spirochaetales
Epsilonproteobacteria
Deltaproteobacteria
Gammaproteobacteria
Brachyspirales
Betaproteobacteria
Alphaproteobacteria
Gammaproteobacteria
Gammaproteobacteria
Nitrospirales
Deltaproteobacteria
Epsilonproteobacteria
Epsilonproteobacteria
Epsilonproteobacteria
Epsilonproteobacteria
Chrysiogenales
Oligoflexia
Deferribacterales
Deltaproteobacteria
Caldithrichae
Nitrososphaerales
Nitrosotaleales
Nitrosopumilales
Nitrosocaldales
Firmicutes
Spirochaetes
Calditrichaeota
Proteobacteria
Verrucomicrobia
Gemmatimonadetes
Verrucomicrobia
Deferribacteres
Proteobacteria
Acidobacteria
Firmicutes
Firmicutes
Firmicutes
Euryarchaeota
Planctomycetes
Gemmatimonadetes
Euryarchaeota
Euryarchaeota
Firmicutes
Euryarchaeota
Thermotogae
Euryarchaeota
Asgard group
Euryarchaeota
Spirochaetes
Spirochaetes
Proteobacteria
Proteobacteria
Proteobacteria
Deferribacteres
Chrysiogenetes
Proteobacteria
Spirochaetes
Spirochaetes
Proteobacteria
Spirochaetes
Proteobacteria
Proteobacteria
Proteobacteria
Nitrospirae
Proteobacteria
Proteobacteria
Proteobacteria
Proteobacteria
Chrysiogenetes
Proteobacteria
Deferribacteres
Proteobacteria
Calditrichaeota
Thaumarchaeota
Bacteria
Bacteria
Bacteria
Bacteria
Archaea
Bacteria
Bacteria
Archaea
Archaea
Bacteria
Archaea
Bacteria
Archaea
Archaea
Bacteria
Archaea

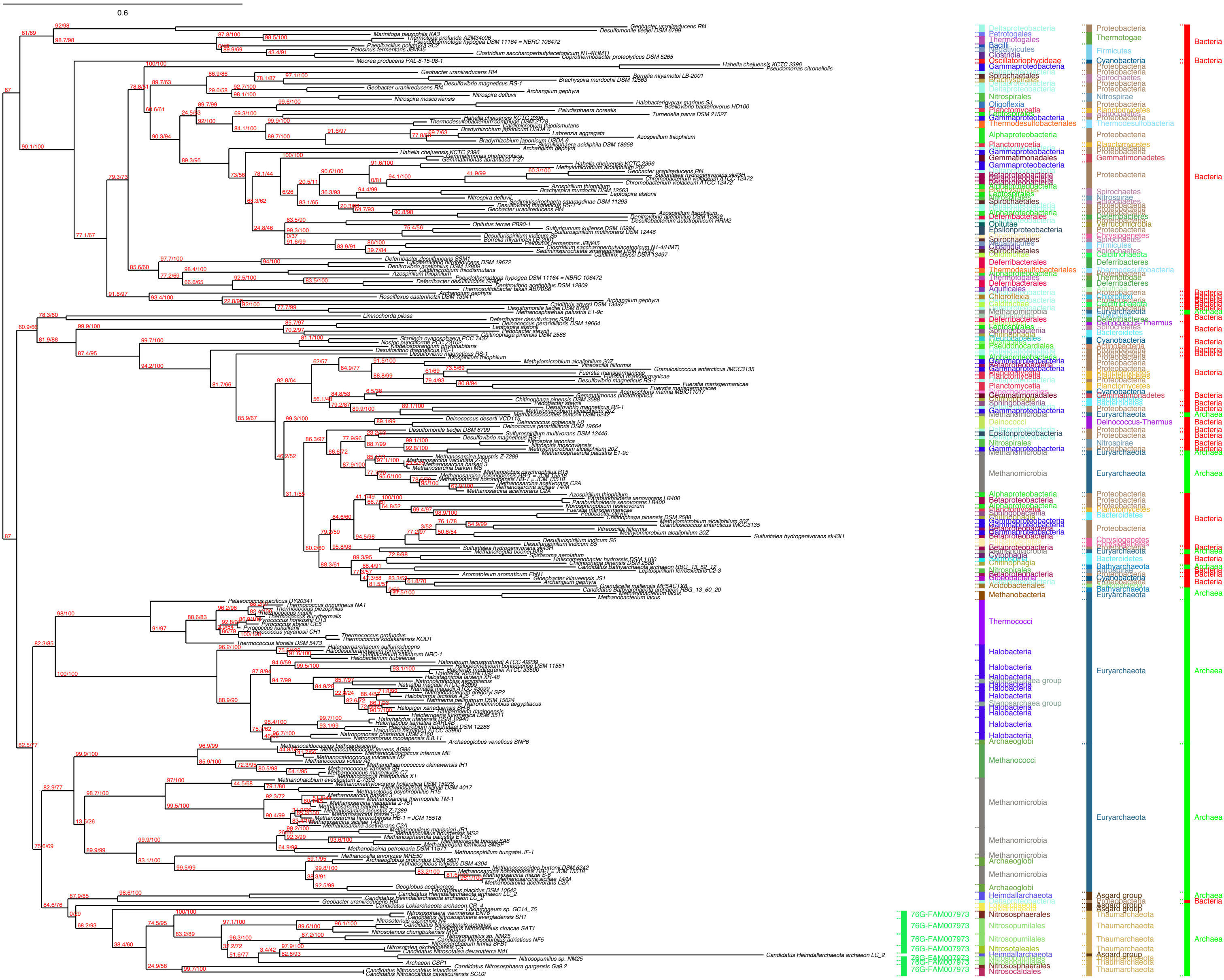
0.6
Methanomicrobia
Thermococci
Halobacteria
Methanococci
Euryarchaeota
Bacteria
Archaea

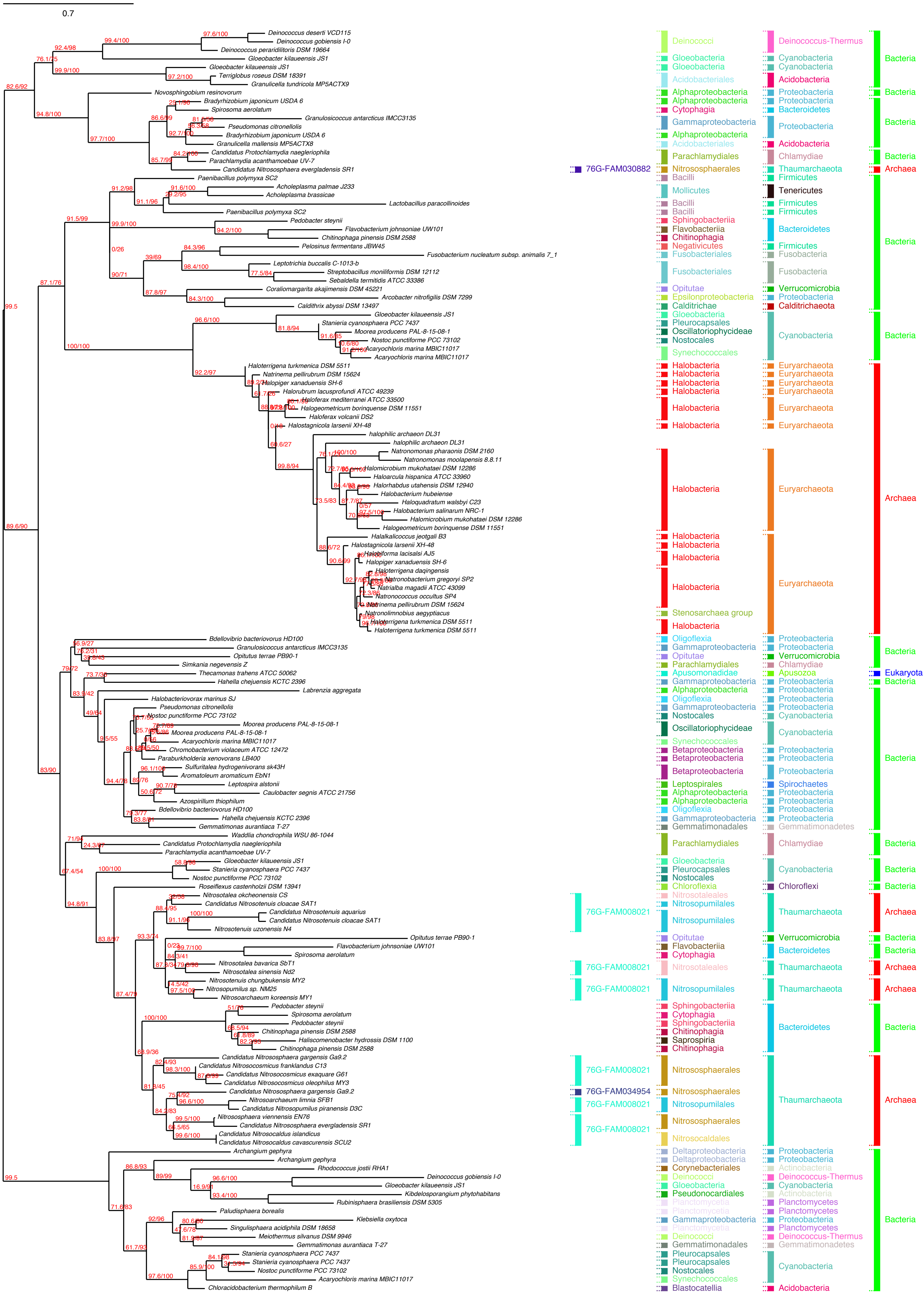

0.7
Deinococcus deserti VCD115
Deinococcus gobiensis I-0
Deinococcus peraridilitoris DSM 19664
Gloeobacter kilaueensis JS1
Gloeobacter kilaueensis JS1
Terriglobus roseus DSM 18391
Granulicella tundricola MP5ACTX9
Novosphingobium resinovorum
Bradyrhizobium japonicum USDA 6
Spirosoma aerolatum
Granulosicoccus antarcticus IMCC3135
Pseudomonas citronellolis
Bradyrhizobium japonicum USDA 6
Granulicella mallensis MP5ACTX8
Candidatus Protochlamydia naegleriophila
Parachlamydia acanthamoebae UV-7
Candidatus Nitrososphaera evergladensis SR1
Paenibacillus polymyxa SC2
Acholeplasma palmae J233
Acholeplasma brassicae
Lactobacillus paracollinoides
Paenibacillus polymyxa SC2
Pedobacter steynii
Flavobacterium johnsoniae UW101
Chitinophaga pinensis DSM 2588
Pelosinus fermentans JBW45
Fusobacterium nucleatum subsp. animalis 7_1
Leptotrichia buccalis C-1013-b
Streptobacillus moniliformis DSM 12112
Sebaldella termitidis ATCC 33386
Coraliomargarita akajimensis DSM 45221
Arcobacter nitrofigilis DSM 7299
Caldithrix abyssi DSM 13497
Gloeobacter kilaueensis JS1
Stanieria cyanosphaera PCC 7437
Moorea producens PAL-8-15-08-1
Nostoc punctiforme PCC 73102
Acaryochloris marina MBIC11017
Acaryochloris marina MBIC11017
Haloterrigena turkmenica DSM 5511
Natrinema pellirubrum DSM 15624
Halopiger xanaduensis SH-6
Halorubrum lacusprofundi ATCC 49239
Haloferax mediterranei ATCC 33500
Halogeometricum borinquense DSM 11551
Haloferax volcanii DS2
Halostagnicola larsenii XH-48
halophilic archaeon DL31
halophilic archaeon DL31
Natronomonas pharaonis DSM 2160
Natronomonas moolapensis 8.8.11
Halomicrobium mukohataei DSM 12286
Haloarcula hispanica ATCC 33960
Halorhabdus utahensis DSM 12940
Halobacterium hubeiense
Haloquadratum walsbyi C23
Halobacterium salinarum NRC-1
Halomicrobium mukohataei DSM 12286
Halogeometricum borinquense DSM 11551
Halalkalicoccus jeotgali B3
Halostagnicola larsenii XH-48
Halobiforma lacisalsi AJ5
Halopiger xanaduensis SH-6
Haloterrigena daqingensis
Natronobacterium gregoryi SP2
Natrialba magadii ATCC 43099
Natronococcus occultus SP4
Natrinema pellirubrum DSM 15624
Natronolimnobius aegyptiacus
Haloterrigena turkmenica DSM 5511
Haloterrigena turkmenica DSM 5511
Bdellovibrio bacteriovorus HD100
Granulosicoccus antarcticus IMCC3135
Opitutus terrae PB90-1
Simkania negevensis Z
Thecamonas trahens ATCC 50062
Hahella chejuensis KCTC 2396
Labrenzia aggregata
Halobacteriovorax marinus SJ
Pseudomonas citronellolis
Nostoc punctiforme PCC 73102
Moorea producens PAL-8-15-08-1
Moorea producens PAL-8-15-08-1
Acaryochloris marina MBIC11017
Chromobacterium violaceum ATCC 12472
Paraburkholderia xenovorans LB400
Sulfuritalea hydrogenivorans sk43H
Aromatoleum aromaticum EbN1
Leptospira alstonii
Caulobacter segnis ATCC 21756
Azospirillum thiophilum
Bdellovibrio bacteriovorus HD100
Hahella chejuensis KCTC 2396
Gemmatimonas aurantiaca T-27
Waddlia chondrophila WSU 86-1044
Candidatus Protochlamydia naegleriophila
Parachlamydia acanthamoebae UV-7
Gloeobacter kilaueensis JS1
Stanieria cyanosphaera PCC 7437
Nostoc punctiforme PCC 73102
Roseiflexus castenholzii DSM 13941
Nitrosotalea okcheonensis CS
Candidatus Nitrosotenuis cloacae SAT1
Candidatus Nitrosotenuis aquarius
Candidatus Nitrosotenuis cloacae SAT1
Nitrosotenuis uzonensis N4
Opitutus terrae PB90-1
Flavobacterium johnsoniae UW101
Spirosoma aerolatum
Nitrosotalea bavarica SbT1
Nitrosotalea sinensis Nd2
Nitrosotenuis chungbukensis MY2
Nitrosopumilus sp. NM25
Nitrosoarchaeum koreensis MY1
Pedobacter steynii
Spirosoma aerolatum
Pedobacter steynii
Chitinophaga pinensis DSM 2588
Haliscomenobacter hydrossis DSM 1100
Chitinophaga pinensis DSM 2588
Candidatus Nitrososphaera gargensis Ga9.2
Candidatus Nitrosocosmicus franklandus C13
Candidatus Nitrosocosmicus exaquare G61
Candidatus Nitrosocosmicus oleophilus MY3
Candidatus Nitrososphaera gargensis Ga9.2
Nitrosoarchaeum limnia SFB1
Candidatus Nitrosopumilus piranensis D3C
Nitrososphaera viennensis EN76
Candidatus Nitrososphaera evergladensis SR1
Candidatus Nitrosocaldus islandicus
Candidatus Nitrosocaldus cavascurensis SCU2
Archangium gephyra
Archangium gephyra
Rhodococcus jostii RHA1
Deinococcus gobiensis I-0
Gloeobacter kilaueensis JS1
Kibdelosporangium phytohabitans
Rubinisphaera brasiliensis DSM 5305
Paludisphaera borealis
Klebsiella oxytoca
Singulisphaera acidiphila DSM 18658
Meiothermus silvanus DSM 9946
Gemmatimonas aurantiaca T-27
Stanieria cyanosphaera PCC 7437
Stanieria cyanosphaera PCC 7437
Nostoc punctiforme PCC 73102
Acaryochloris marina MBIC11017
Chloracidobacterium thermophilum B
76G-FAM030882
76G-FAM008021
76G-FAM008021
76G-FAM008021
76G-FAM008021
76G-FAM034954
76G-FAM008021
76G-FAM008021
Deinococci
Gloeobacteria
Gloeobacteria
Acidobacteriales
Alphaproteobacteria
Alphaproteobacteria
Cytophagia
Gammaproteobacteria
Alphaproteobacteria
Acidobacteriales
Parachlamydiales
Nitrososphaerales
Bacilli
Mollicutes
Bacilli
Bacilli
Sphingobacteriia
Flavobacteriia
Chitinophagia
Negativicutes
Fusobacteriales
Fusobacteriales
Opitutae
Epsilonproteobacteria
Calditrichae
Gloeobacteria
Pleurocapsales
Oscillatoriophycideae
Nostocales
Synechococcales
Halobacteria
Halobacteria
Halobacteria
Halobacteria
Halobacteria
Halobacteria
Halobacteria
Halobacteria
Halobacteria
Halobacteria
Halobacteria
Stenosarchaea group
Halobacteria
Oligoflexia
Gammaproteobacteria
Opitutae
Parachlamydiales
Apusomonadidae
Gammaproteobacteria
Alphaproteobacteria
Oligoflexia
Gammaproteobacteria
Nostocales
Oscillatoriophycideae
Synechococcales
Betaproteobacteria
Betaproteobacteria
Betaproteobacteria
Leptospirales
Alphaproteobacteria
Alphaproteobacteria
Oligoflexia
Gammaproteobacteria
Gemmatimonadales
Parachlamydiales
Gloeobacteria
Pleurocapsales
Nostocales
Chloroflexia
Nitrosotaleales
Nitrosopumilales
Nitrosopumilales
Opitutae
Flavobacteriia
Cytophagia
Nitrosotaleales
Nitrosopumilales
Sphingobacteriia
Cytophagia
Sphingobacteriia
Chitinophagia
Saprospiria
Chitinophagia
Nitrososphaerales
Nitrososphaerales
Nitrosopumilales
Nitrososphaerales
Nitrosocaldales
Deltaproteobacteria
Deltaproteobacteria
Corynebacteriales
Deinococci
Gloeobacteria
Pseudonocardiales
Planctomycetia
Planctomycetia
Gammaproteobacteria
Planctomycetia
Deinococci
Gemmatimonadales
Pleurocapsales
Pleurocapsales
Nostocales
Synechococcales
Blastocatellia
Deinococcus-Thermus
Cyanobacteria
Cyanobacteria
Acidobacteria
Proteobacteria
Proteobacteria
Bacteroidetes
Proteobacteria
Acidobacteria
Chlamydiae
Thaumarchaeota
Firmicutes
Tenericutes
Firmicutes
Firmicutes
Bacteroidetes
Firmicutes
Fusobacteria
Fusobacteria
Verrucomicrobia
Proteobacteria
Calditrichaeota
Cyanobacteria
Euryarchaeota
Euryarchaeota
Euryarchaeota
Euryarchaeota
Euryarchaeota
Euryarchaeota
Euryarchaeota
Euryarchaeota
Proteobacteria
Proteobacteria
Verrucomicrobia
Chlamydiae
Apusozoa
Proteobacteria
Proteobacteria
Proteobacteria
Proteobacteria
Cyanobacteria
Cyanobacteria
Proteobacteria
Proteobacteria
Proteobacteria
Spirochaetes
Proteobacteria
Proteobacteria
Proteobacteria
Proteobacteria
Gemmatimonadetes
Chlamydiae
Cyanobacteria
Chloroflexi
Thaumarchaeota
Verrucomicrobia
Bacteroidetes
Thaumarchaeota
Thaumarchaeota
Bacteroidetes
Thaumarchaeota
Proteobacteria
Proteobacteria
Actinobacteria
Deinococcus-Thermus
Cyanobacteria
Actinobacteria
Planctomycetes
Planctomycetes
Proteobacteria
Planctomycetes
Deinococcus-Thermus
Gemmatimonadetes
Cyanobacteria
Acidobacteria
Bacteria
Bacteria
Bacteria
Bacteria
Archaea
Bacteria
Bacteria
Archaea
Bacteria
Eukaryota
Bacteria
Bacteria
Bacteria
Bacteria
Bacteria
Archaea
Bacteria
Bacteria
Archaea
Archaea
Bacteria
Archaea
Bacteria

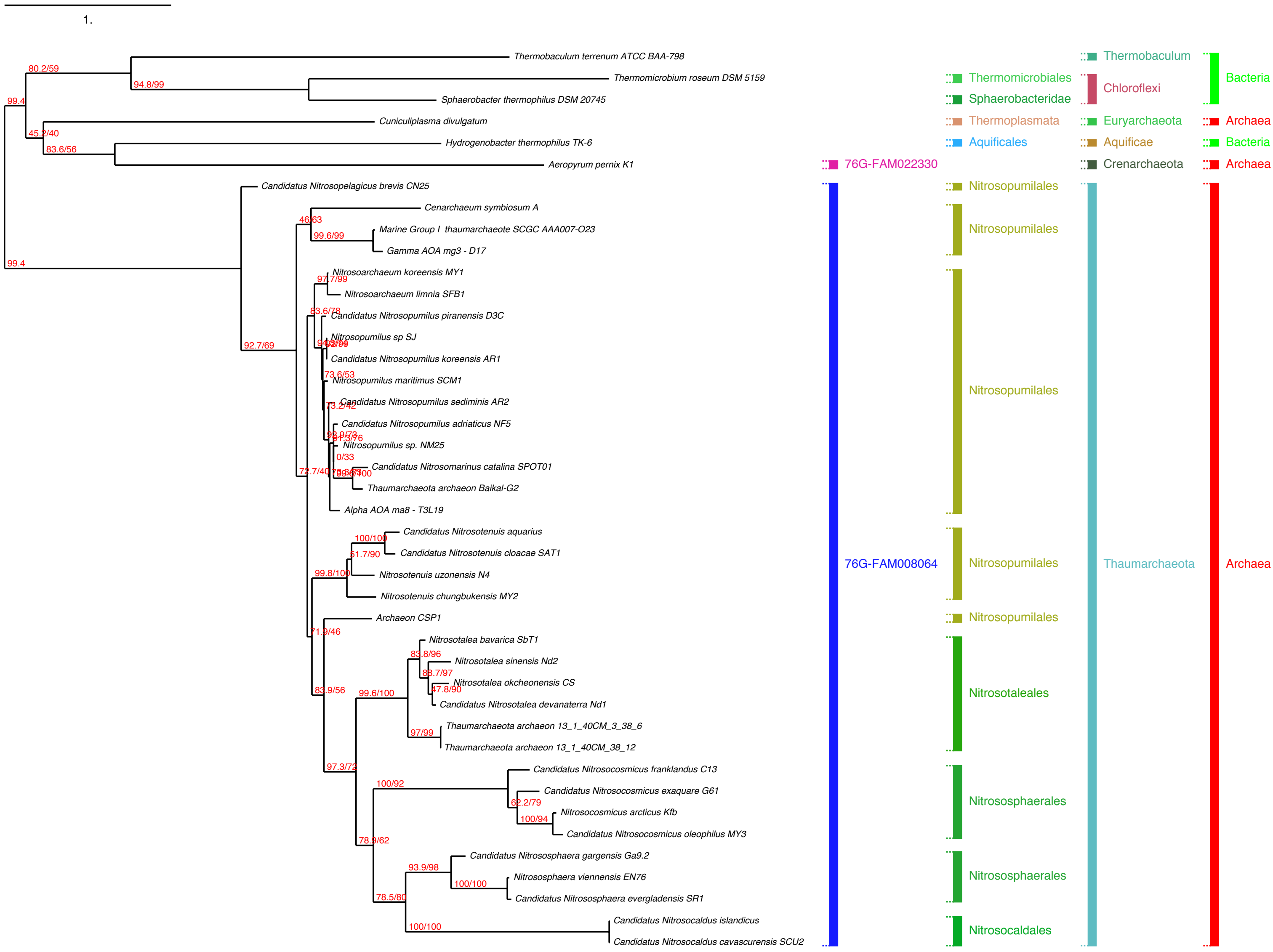
1.
Thermobaculum terrenum ATCC BAA-798
Thermomicrobium roseum DSM 5159
Sphaerobacter thermophilus DSM 20745
Cuniculiplasma divulgatum
Hydrogenobacter thermophilus TK-6
Aeropyrum pernix K1
Candidatus Nitrosopelagicus brevis CN25
Cenarchaeum symbiosum A
Marine Group I thaumarchaeote SCGC AAA007-O23
Gamma AOA mg3 - D17
Nitrosoarchaeum koreensis MY1
Nitrosoarchaeum limnia SFB1
Candidatus Nitrosopumilus piranensis D3C
Nitrosopumilus sp SJ
Candidatus Nitrosopumilus koreensis AR1
Nitrosopumilus maritimus SCM1
Candidatus Nitrosopumilus sediminis AR2
Candidatus Nitrosopumilus adriaticus NF5
Nitrosopumilus sp. NM25
Candidatus Nitrosomarinus catalina SPOT01
Thaumarchaeota archaeon Baikal-G2
Alpha AOA ma8 - T3L19
Candidatus Nitrosotenuis aquarius
Candidatus Nitrosotenuis cloacae SAT1
Nitrosotenuis uzonensis N4
Nitrosotenuis chungbukensis MY2
Archaeon CSP1
Nitrosotalea bavarica SbT1
Nitrosotalea sinensis Nd2
Nitrosotalea okcheonensis CS
Candidatus Nitrosotalea devanaterra Nd1
Thaumarchaeota archaeon 13_1_40CM_3_38_6
Thaumarchaeota archaeon 13_1_40CM_38_12
Candidatus Nitrosocosmicus franklandus C13
Candidatus Nitrosocosmicus exaquare G61
Nitrosocosmicus arcticus Kfb
Candidatus Nitrosocosmicus oleophilus MY3
Candidatus Nitrososphaera gargensis Ga9.2
Nitrososphaera viennensis EN76
Candidatus Nitrososphaera evergladensis SR1
Candidatus Nitrosocaldus islandicus
Candidatus Nitrosocaldus cavascurensis SCU2
80.2/59
94.8/99
99.4
45.2/40
83.6/56
99.4
92.7/69
46/63
99.6/99
97.7/99
83.6/78
73.6/53
73.2/42
0/33
72.7/40
100/100
51.7/90
99.8/100
71.9/46
83.8/96
88.7/97
47.8/90
83.9/56
99.6/100
97/99
97.3/72
100/92
62.2/79
100/94
78.9/62
93.9/98
100/100
78.5/80
100/100
76G-FAM022330
76G-FAM008064
Thermomicrobiales
Sphaerobacteridae
Thermoplasmata
Aquificales
Nitrosopumilales
Nitrosopumilales
Nitrosopumilales
Nitrosopumilales
Nitrosopumilales
Nitrosotaleales
Nitrososphaerales
Nitrososphaerales
Nitrosocaldales
Thermobaculum
Chloroflexi
Euryarchaeota
Aquificae
Crenarchaeota
Thaumarchaeota
Bacteria
Archaea
Bacteria
Archaea
Archaea

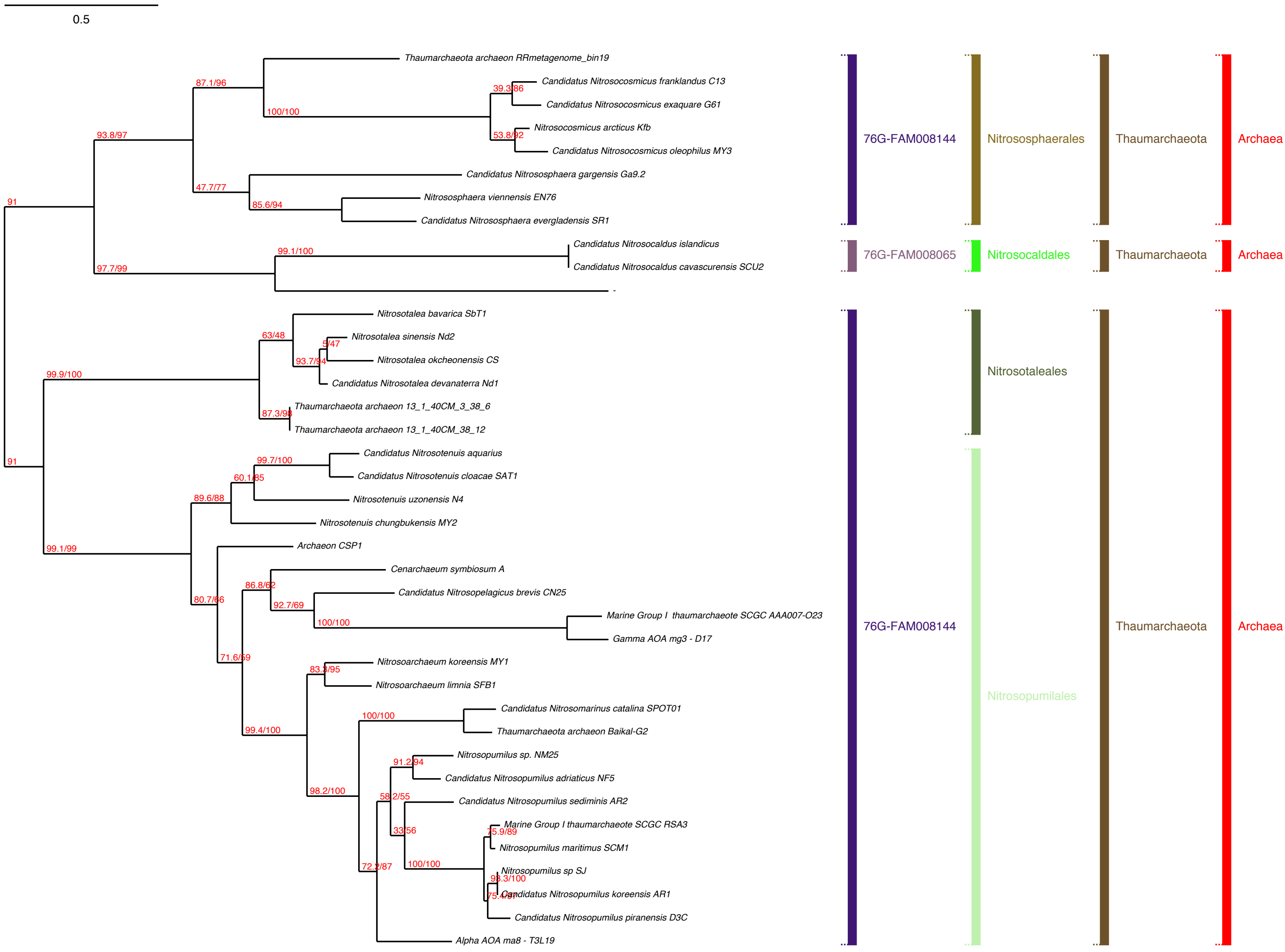

0.5
Thaumarchaeota archaeon RRmetagenome_bin19
Candidatus Nitrosocosmicus franklandus C13
Candidatus Nitrosocosmicus exaquare G61
Nitrosocosmicus arcticus Kfb
Candidatus Nitrosocosmicus oleophilus MY3
Candidatus Nitrososphaera gargensis Ga9.2
Nitrososphaera viennensis EN76
Candidatus Nitrososphaera evergladensis SR1
Candidatus Nitrosocaldus islandicus
Candidatus Nitrosocaldus cavascurensis SCU2
Nitrosotalea bavarica SbT1
Nitrosotalea sinensis Nd2
Nitrosotalea okcheonensis CS
Candidatus Nitrosotalea devanaterra Nd1
Thaumarchaeota archaeon 13_1_40CM_3_38_6
Thaumarchaeota archaeon 13_1_40CM_38_12
Candidatus Nitrosotenuis aquarius
Candidatus Nitrosotenuis cloacae SAT1
Nitrosotenuis uzonensis N4
Nitrosotenuis chungbukensis MY2
Archaeon CSP1
Cenarchaeum symbiosum A
Candidatus Nitrosopelagicus brevis CN25
Marine Group I thaumarchaeote SCGC AAA007-O23
Gamma AOA mg3 - D17
Nitrosoarchaeum koreensis MY1
Nitrosoarchaeum limnia SFB1
Candidatus Nitrosomarinus catalina SPOT01
Thaumarchaeota archaeon Baikal-G2
Nitrosopumilus sp. NM25
Candidatus Nitrosopumilus adriaticus NF5
Candidatus Nitrosopumilus sediminis AR2
Marine Group I thaumarchaeote SCGC RSA3
Nitrosopumilus maritimus SCM1
Nitrosopumilus sp SJ
Candidatus Nitrosopumilus koreensis AR1
Candidatus Nitrosopumilus piranensis D3C
Alpha AOA ma8 - T3L19
76G-FAM008144
Nitrososphaerales
Thaumarchaeota
Archaea
76G-FAM008065
Nitrosocaldales
Thaumarchaeota
Archaea
76G-FAM008144
Nitrosotaleales
Nitrosopumilales
Thaumarchaeota
Archaea

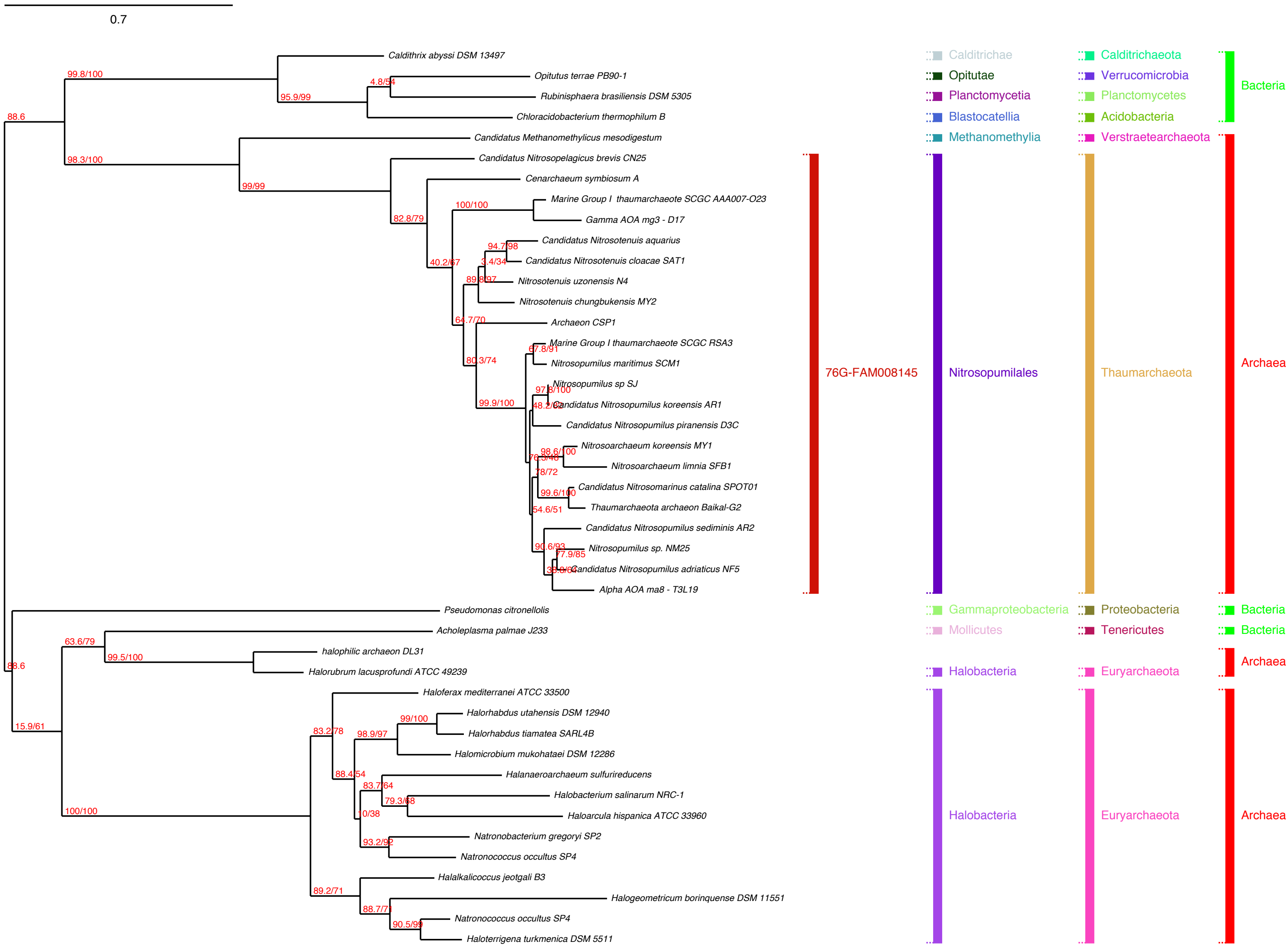

0.7
Caldithrix abyssi DSM 13497
Opitutus terrae PB90-1
Rubinisphaera brasiliensis DSM 5305
Chloracidobacterium thermophilum B
Candidatus Methanomethylicus mesodigestum
Candidatus Nitrosopelagicus brevis CN25
Cenarchaeum symbiosum A
Marine Group I thaumarchaeote SCGC AAA007-O23
Gamma AOA mg3 - D17
Candidatus Nitrosotenuis aquarius
Candidatus Nitrosotenuis cloacae SAT1
Nitrosotenuis uzonensis N4
Nitrosotenuis chungbukensis MY2
Archaeon CSP1
Marine Group I thaumarchaeote SCGC RSA3
Nitrosopumilus maritimus SCM1
Nitrosopumilus sp SJ
Candidatus Nitrosopumilus koreensis AR1
Candidatus Nitrosopumilus piranensis D3C
Nitrosoarchaeum koreensis MY1
Nitrosoarchaeum limnia SFB1
Candidatus Nitrosomarinus catalina SPOT01
Thaumarchaeota archaeon Baikal-G2
Candidatus Nitrosopumilus sediminis AR2
Nitrosopumilus sp. NM25
Candidatus Nitrosopumilus adriaticus NF5
Alpha AOA ma8 - T3L19
Pseudomonas citronellolis
Acholeplasma palmae J233
halophilic archaeon DL31
Halorubrum lacusprofundi ATCC 49239
Haloferax mediterranei ATCC 33500
Halorhabdus utahensis DSM 12940
Halorhabdus tiamatea SARL4B
Halomicrobium mukohataei DSM 12286
Halanaeroarchaeum sulfurireducens
Halobacterium salinarum NRC-1
Haloarcula hispanica ATCC 33960
Natronobacterium gregoryi SP2
Natronococcus occultus SP4
Halalkalicoccus jeotgali B3
Halogeometricum borinquense DSM 11551
Natronococcus occultus SP4
Haloterrigena turkmenica DSM 5511
76G-FAM008145
Calditrichae
Opitutae
Planctomycetia
Blastocatellia
Methanomethylia
Nitrosopumilales
Gammaproteobacteria
Mollicutes
Halobacteria
Halobacteria
Calditrichaeota
Verrucomicrobia
Planctomycetes
Acidobacteria
Verstraetearchaeota
Thaumarchaeota
Proteobacteria
Tenericutes
Euryarchaeota
Euryarchaeota
Bacteria
Archaea
Bacteria
Bacteria
Archaea
Archaea

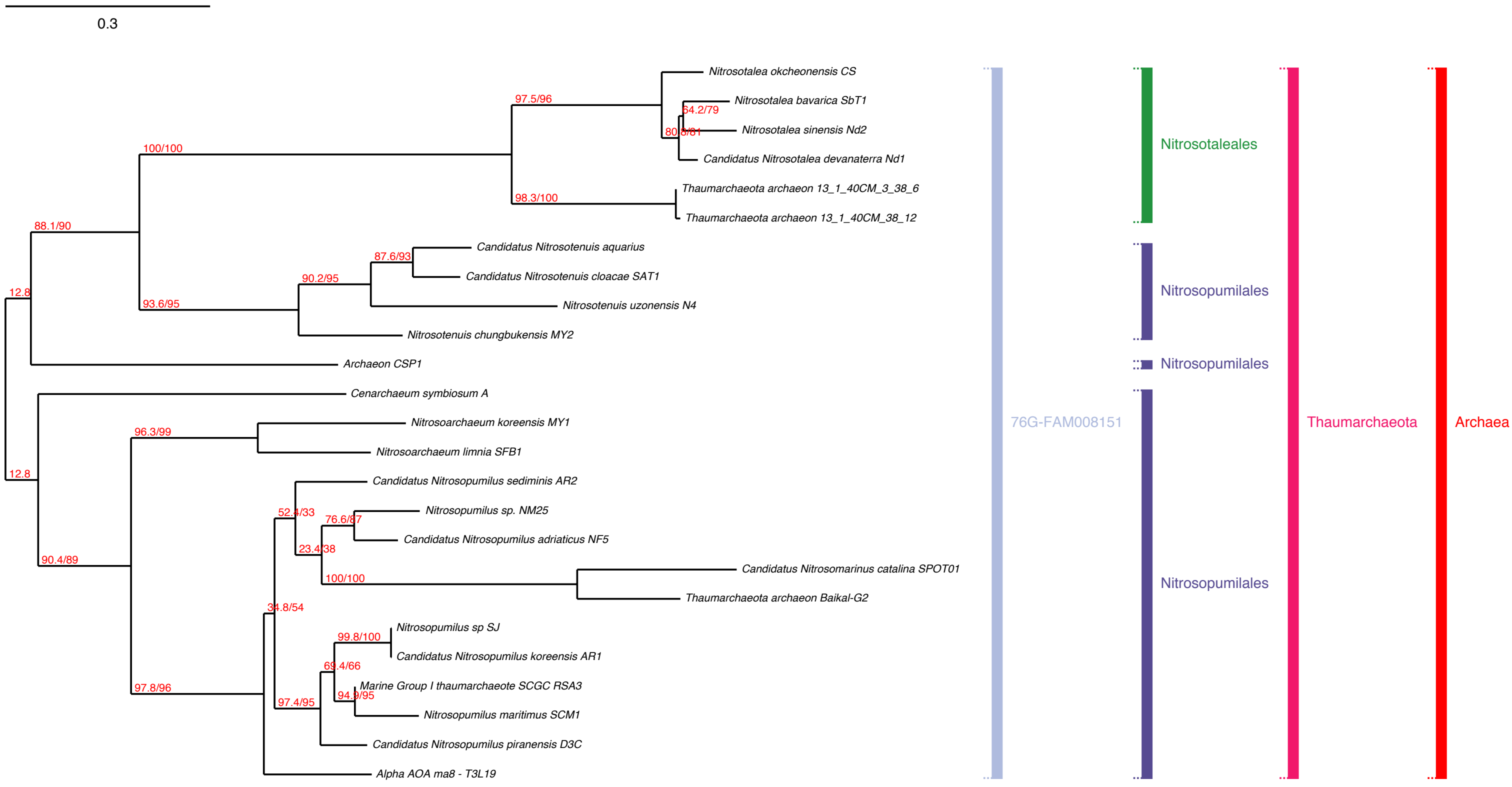
0.3
Nitrosotalea okcheonensis CS
Nitrosotalea bavarica SbT1
Nitrosotalea sinensis Nd2
Candidatus Nitrosotalea devanaterra Nd1
Thaumarchaeota archaeon 13_1_40CM_3_38_6
Thaumarchaeota archaeon 13_1_40CM_38_12
Candidatus Nitrosotenuis aquarius
Candidatus Nitrosotenuis cloacae SAT1
Nitrosotenuis uzonensis N4
Nitrosotenuis chungbukensis MY2
Archaeon CSP1
Cenarchaeum symbiosum A
Nitrosoarchaeum koreensis MY1
Nitrosoarchaeum limnia SFB1
Candidatus Nitrosopumilus sediminis AR2
Nitrosopumilus sp. NM25
Candidatus Nitrosopumilus adriaticus NF5
Candidatus Nitrosomarinus catalina SPOT01
Thaumarchaeota archaeon Baikal-G2
Nitrosopumilus sp SJ
Candidatus Nitrosopumilus koreensis AR1
Marine Group I thaumarchaeote SCGC RSA3
Nitrosopumilus maritimus SCM1
Candidatus Nitrosopumilus piranensis D3C
Alpha AOA ma8 - T3L19
97.5/96
64.2/79
80.8/81
100/100
98.3/100
88.1/90
87.6/93
90.2/95
12.8
93.6/95
96.3/99
12.8
52.4/33
76.6/87
23.4/38
90.4/89
100/100
34.8/54
99.8/100
69.4/66
94.9/95
97.8/96
97.4/95
76G-FAM008151
Nitrosotaleales
Nitrosopumilales
Nitrosopumilales
Nitrosopumilales
Thaumarchaeota
Archaea

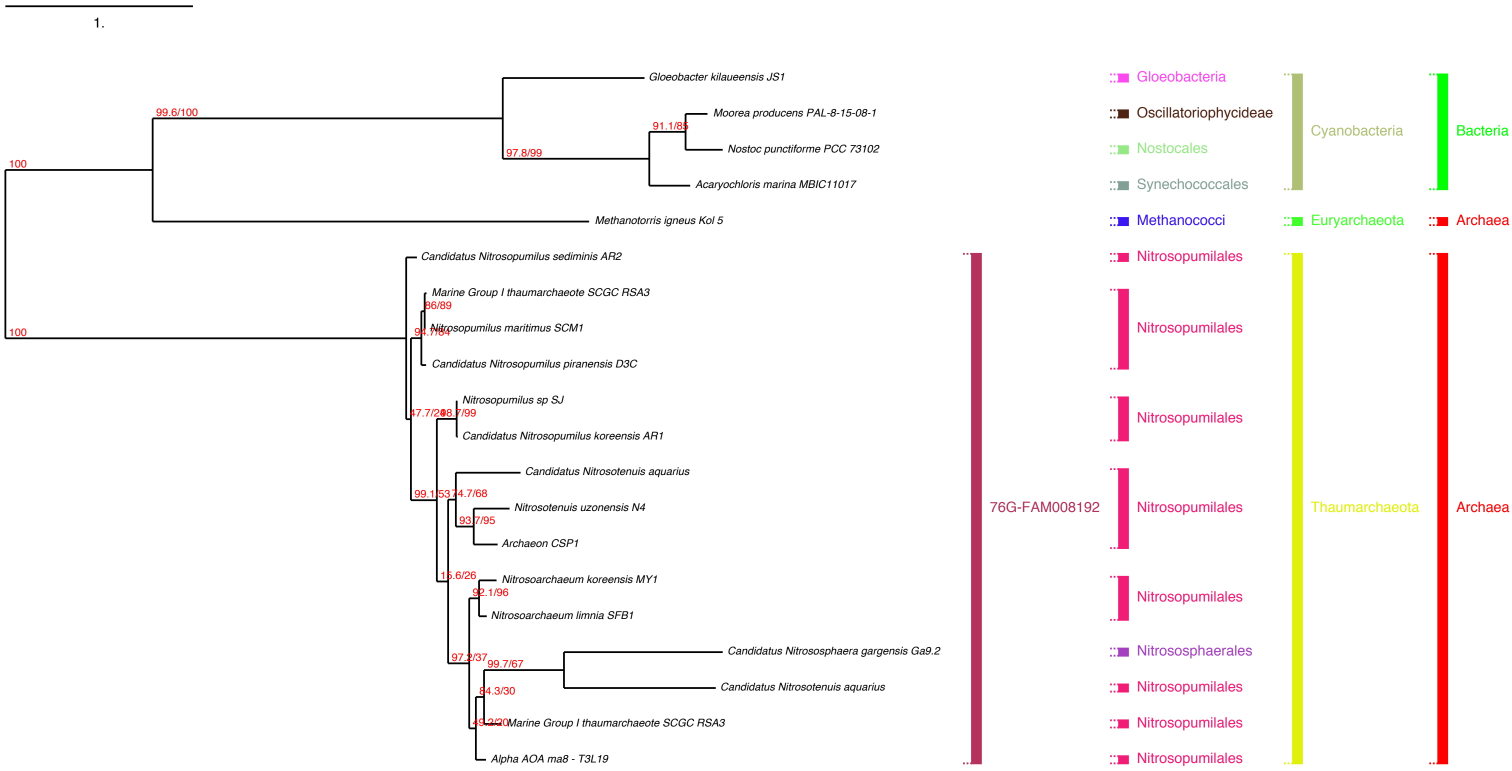

1.
Gloeobacter kilaueensis JS1
99.6/100
Moorea producens PAL-8-15-08-1
91.1/85
100
97.8/99
Nostoc punctiforme PCC 73102
Acaryochloris marina MBIC11017
Methanotorris igneus Kol 5
Candidatus Nitrosopumilus sediminis AR2
Marine Group I thaumarchaeote SCGC RSA3
86/89
100
94.7/84
Nitrosopumilus maritimus SCM1
Candidatus Nitrosopumilus piranensis D3C
Nitrosopumilus sp SJ
47.7/20
98.7/99
Candidatus Nitrosopumilus koreensis AR1
Candidatus Nitrosotenuis aquarius
99.1/53
74.7/68
Nitrosotenuis uzonensis N4
93.7/95
Archaeon CSP1
15.6/26
Nitrosoarchaeum koreensis MY1
92.1/96
Nitrosoarchaeum limnia SFB1
Candidatus Nitrososphaera gargensis Ga9.2
97.2/37
99.7/67
Candidatus Nitrosotenuis aquarius
84.3/30
49.2/20
Marine Group I thaumarchaeote SCGC RSA3
Alpha AOA ma8 - T3L19
76G-FAM008192
Gloeobacteria
Oscillatoriophycideae
Nostocales
Synechococcales
Methanococci
Nitrosopumilales
Nitrosopumilales
Nitrosopumilales
Nitrosopumilales
Nitrosopumilales
Nitrososphaerales
Nitrosopumilales
Nitrosopumilales
Nitrosopumilales
Cyanobacteria
Euryarchaeota
Thaumarchaeota
Bacteria
Archaea
Archaea

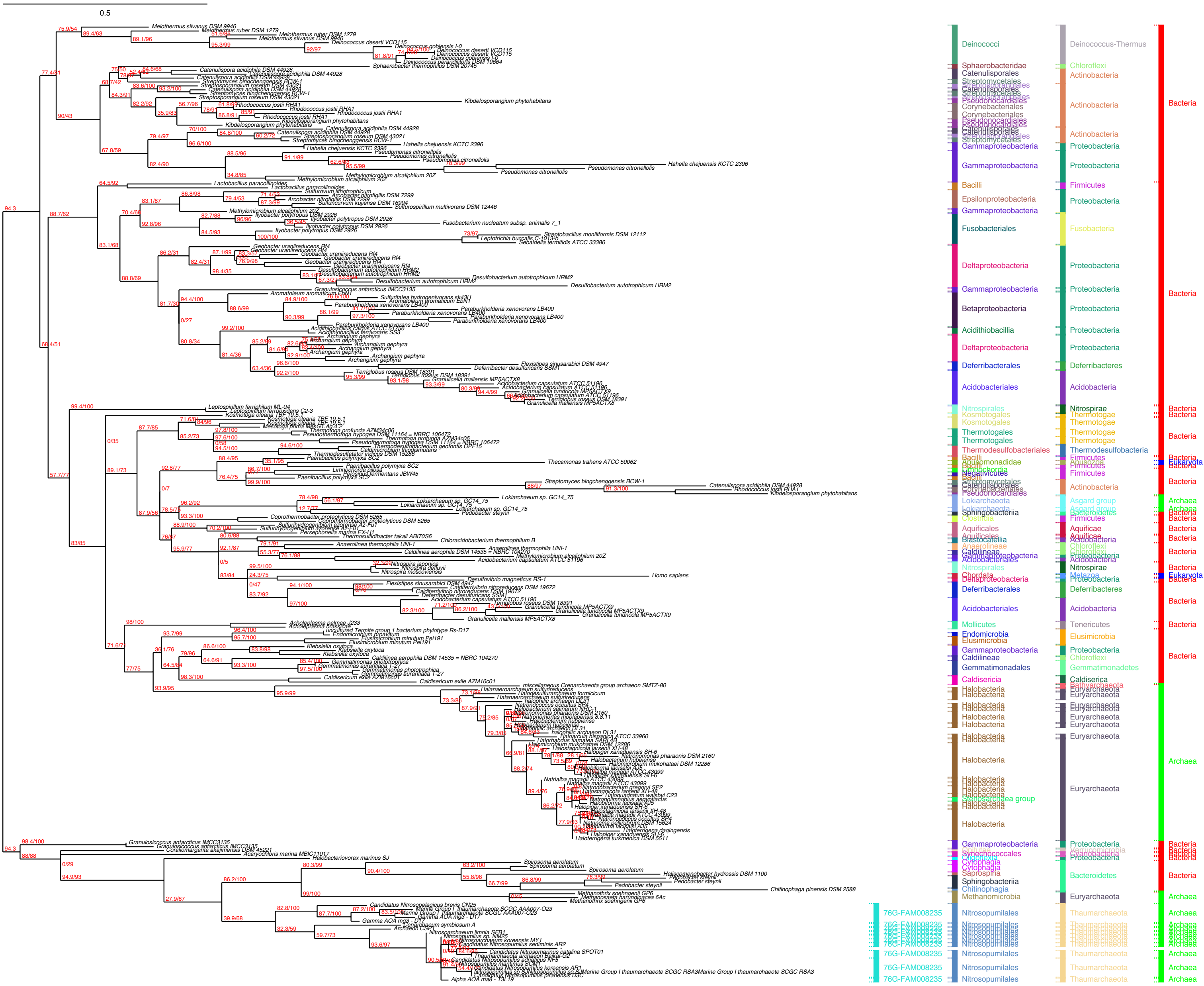
0.5
Deinococci
Deinococcus-Thermus
Sphaerobacteridae
Chloroflexi
Catenulisporales
Actinobacteria
Streptomycetales
Corynebacteriales
Pseudonocardiales
Gammaproteobacteria
Proteobacteria
Bacilli
Firmicutes
Epsilonproteobacteria
Fusobacteriales
Fusobacteria
Deltaproteobacteria
Betaproteobacteria
Acidithiobacillia
Deferribacterales
Deferribacteres
Acidobacteriales
Acidobacteria
Nitrospirales
Nitrospirae
Kosmotogales
Thermotogae
Thermotogales
Thermodesulfobacteriales
Thermodesulfobacteria
Apusomonadidae
Apusozoa
Eukaryota
Negativicutes
Lokiarchaeota
Asgard group
Sphingobacteriia
Bacteroidetes
Clostridia
Aquificales
Aquificae
Blastocatellia
Anaerolineae
Caldilineae
Chordata
Metazoa
Mollicutes
Tenericutes
Endomicrobia
Elusimicrobia
Gemmatimonadales
Gemmatimonadetes
Caldisericia
Bathyarchaeota
Halobacteria
Euryarchaeota
Stenosarchaea group
Synechococcales
Cyanobacteria
Cytophagia
Saprospiria
Chitinophagia
Methanomicrobia
76G-FAM008235
Nitrosopumilales
Thaumarchaeota
Bacteria
Archaea

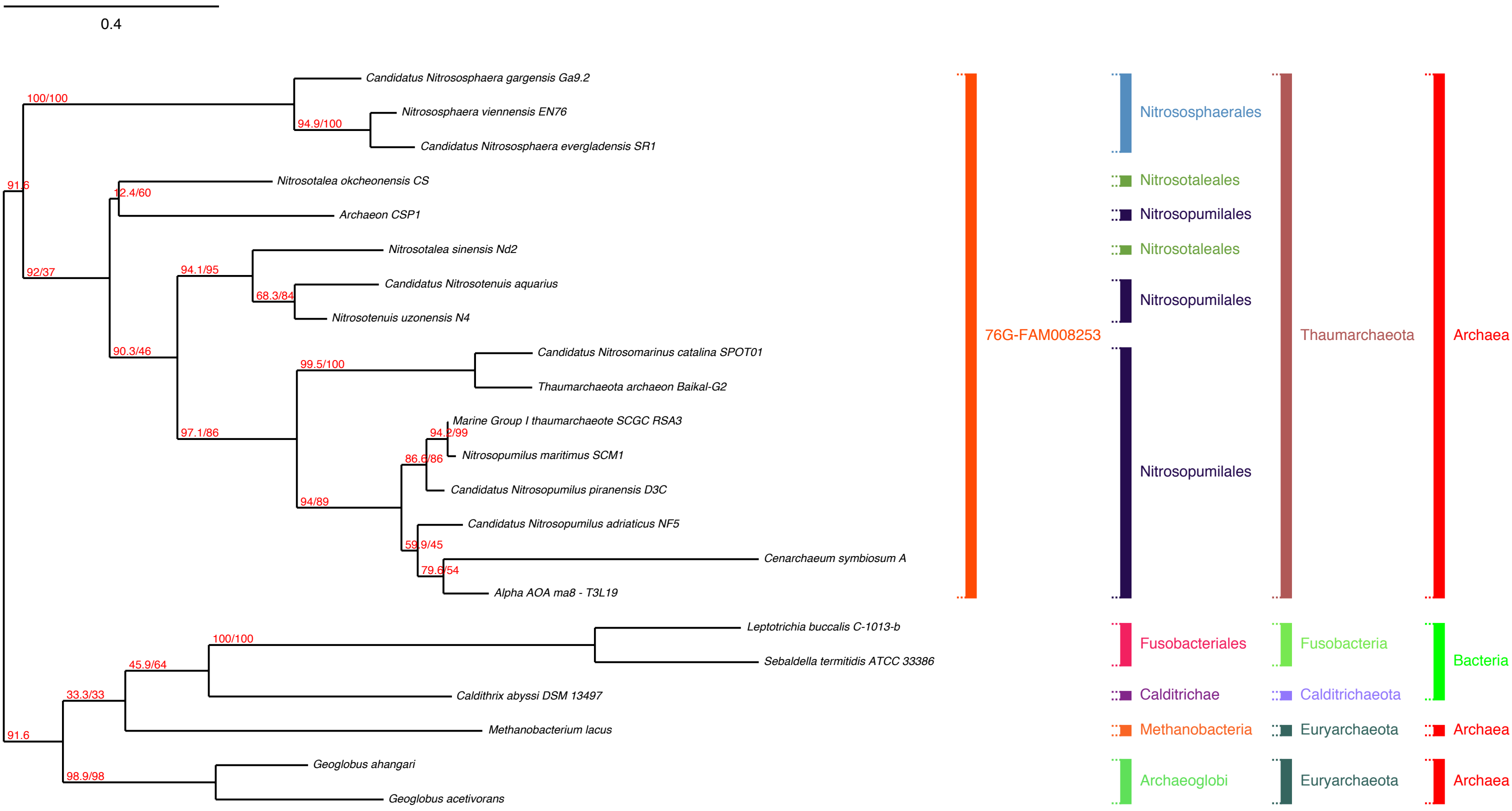
0.4
Candidatus Nitrososphaera gargensis Ga9.2
Nitrososphaera viennensis EN76
Candidatus Nitrososphaera evergladensis SR1
Nitrosotalea okcheonensis CS
Archaeon CSP1
Nitrosotalea sinensis Nd2
Candidatus Nitrosotenuis aquarius
Nitrosotenuis uzonensis N4
Candidatus Nitrosomarinus catalina SPOT01
Thaumarchaeota archaeon Baikal-G2
Marine Group I thaumarchaeote SCGC RSA3
Nitrosopumilus maritimus SCM1
Candidatus Nitrosopumilus piranensis D3C
Candidatus Nitrosopumilus adriaticus NF5
Cenarchaeum symbiosum A
Alpha AOA ma8 - T3L19
Leptotrichia buccalis C-1013-b
Sebaldella termitidis ATCC 33386
Caldithrix abyssi DSM 13497
Methanobacterium lacus
Geoglobus ahangari
Geoglobus acetivorans
100/100
94.9/100
91.6
12.4/60
92/37
94.1/95
68.3/84
90.3/46
99.5/100
97.1/86
94.2/99
86.6/86
94/89
59.9/45
79.6/54
100/100
45.9/64
33.3/33
91.6
98.9/98
76G-FAM008253
Nitrososphaerales
Nitrosotaleales
Nitrosopumilales
Nitrosotaleales
Nitrosopumilales
Nitrosopumilales
Fusobacteriales
Calditrichae
Methanobacteria
Archaeoglobi
Thaumarchaeota
Fusobacteria
Calditrichaeota
Euryarchaeota
Euryarchaeota
Archaea
Bacteria
Archaea
Archaea

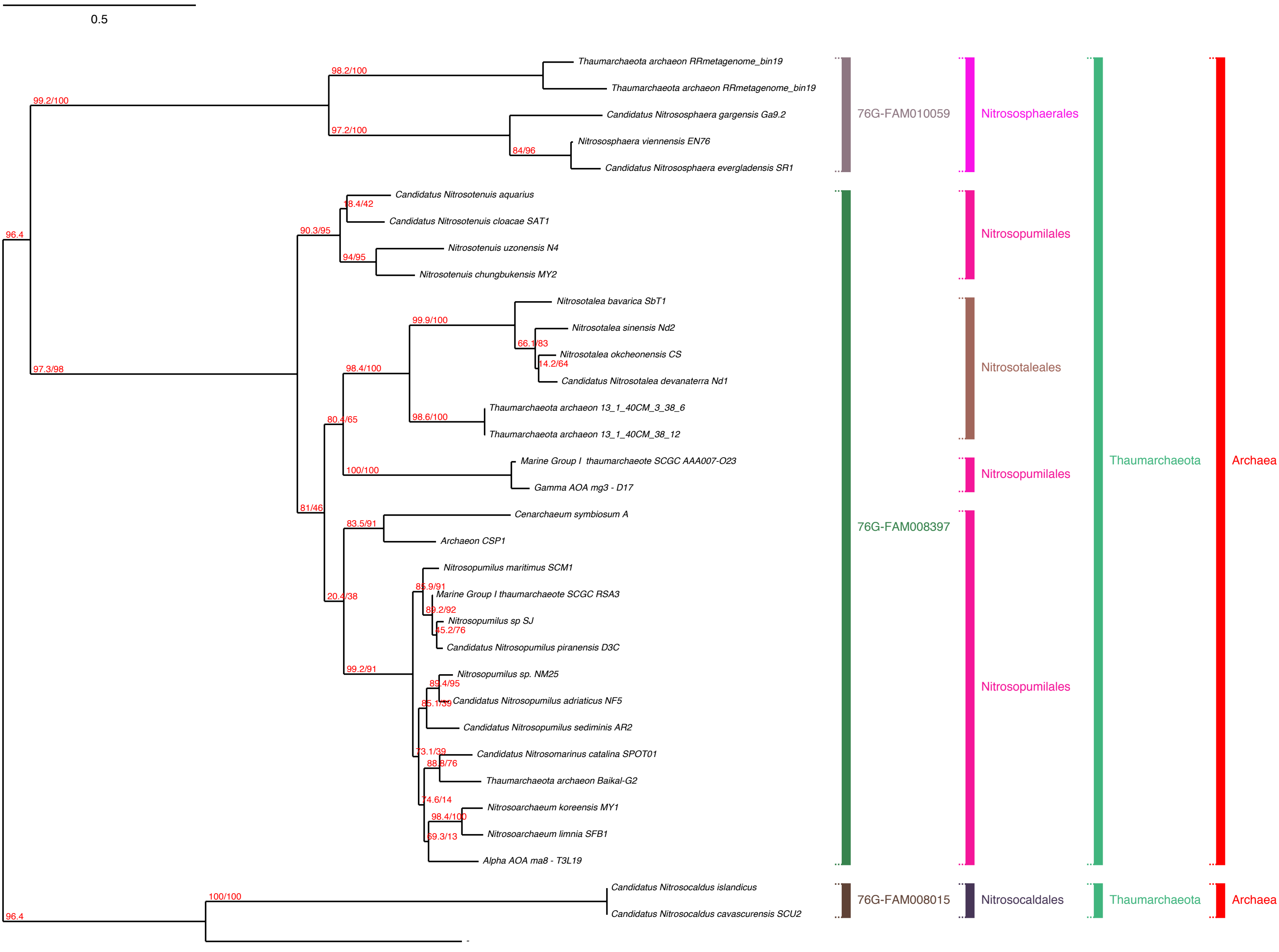
0.5
Thaumarchaeota archaeon RRmetagenome_bin19
Thaumarchaeota archaeon RRmetagenome_bin19
Candidatus Nitrososphaera gargensis Ga9.2
Nitrososphaera viennensis EN76
Candidatus Nitrososphaera evergladensis SR1
Candidatus Nitrosotenuis aquarius
Candidatus Nitrosotenuis cloacae SAT1
Nitrosotenuis uzonensis N4
Nitrosotenuis chungbukensis MY2
Nitrosotalea bavarica SbT1
Nitrosotalea sinensis Nd2
Nitrosotalea okcheonensis CS
Candidatus Nitrosotalea devanaterra Nd1
Thaumarchaeota archaeon 13_1_40CM_3_38_6
Thaumarchaeota archaeon 13_1_40CM_38_12
Marine Group I thaumarchaeote SCGC AAA007-O23
Gamma AOA mg3 - D17
Cenarchaeum symbiosum A
Archaeon CSP1
Nitrosopumilus maritimus SCM1
Marine Group I thaumarchaeote SCGC RSA3
Nitrosopumilus sp SJ
Candidatus Nitrosopumilus piranensis D3C
Nitrosopumilus sp. NM25
Candidatus Nitrosopumilus adriaticus NF5
Candidatus Nitrosopumilus sediminis AR2
Candidatus Nitrosomarinus catalina SPOT01
Thaumarchaeota archaeon Baikal-G2
Nitrosoarchaeum koreensis MY1
Nitrosoarchaeum limnia SFB1
Alpha AOA ma8 - T3L19
Candidatus Nitrosocaldus islandicus
Candidatus Nitrosocaldus cavascurensis SCU2
-
96.4
99.2/100
98.2/100
97.2/100
84/96
97.3/98
90.3/95
18.4/42
94/95
81/46
99.9/100
66.1/83
14.2/64
98.4/100
98.6/100
80.4/65
100/100
83.5/91
20.4/38
85.9/91
89.2/92
45.2/76
99.2/91
89.4/95
85.1/39
73.1/39
88.8/76
74.6/14
98.4/100
69.3/13
100/100
96.4
76G-FAM010059
Nitrososphaerales
76G-FAM008397
Nitrosopumilales
Nitrosotaleales
Nitrosopumilales
Nitrosopumilales
Thaumarchaeota
Archaea
76G-FAM008015
Nitrosocaldales
Thaumarchaeota
Archaea

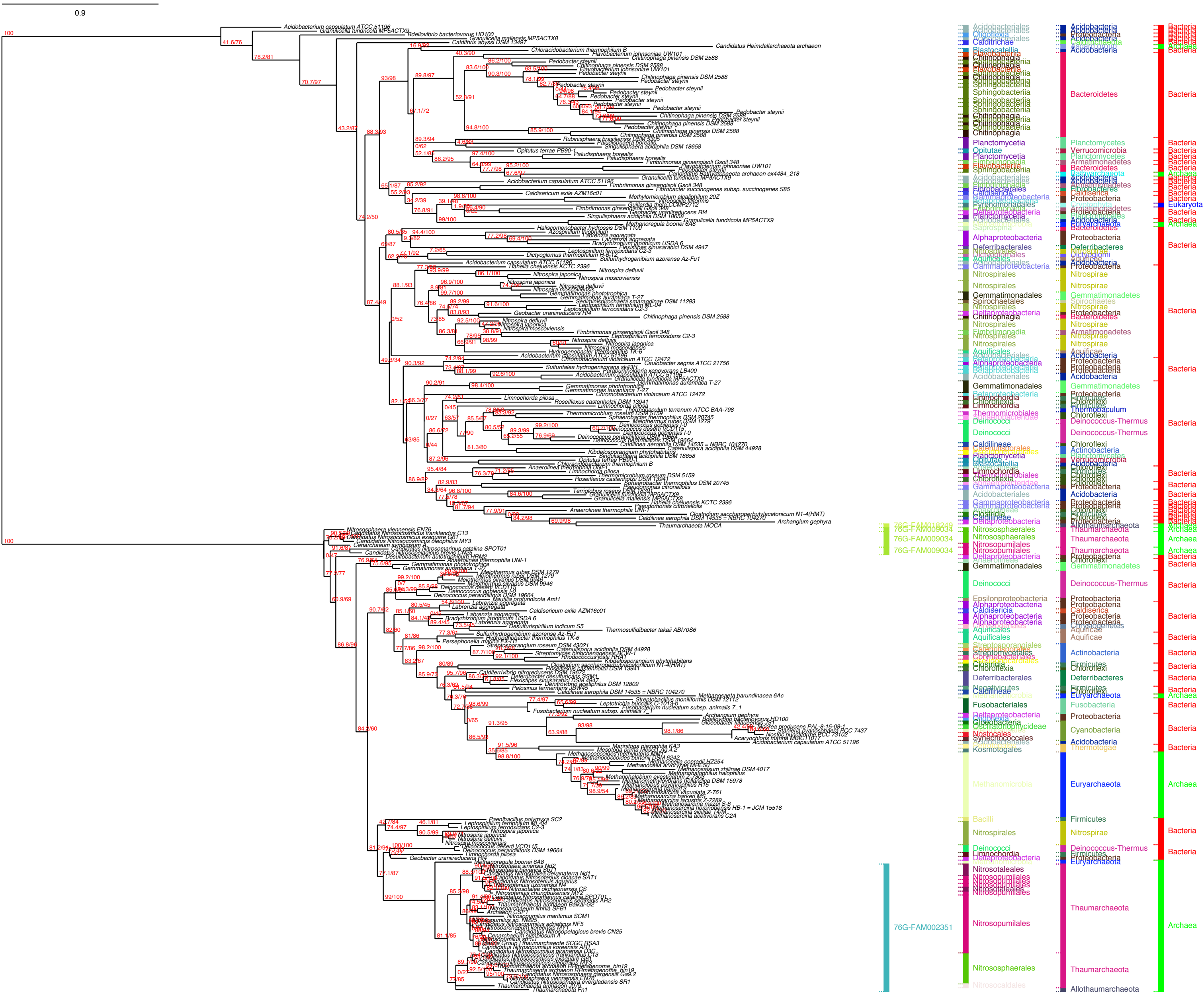

0.9
76G-FAM009034
76G-FAM002351
Bacteroidetes
Thaumarchaeota
Euryarchaeota
Deinococcus-Thermus
Methanomicrobia
Nitrosopumilales
Nitrososphaerales
Bacteria
Archaea
Eukaryota

2.

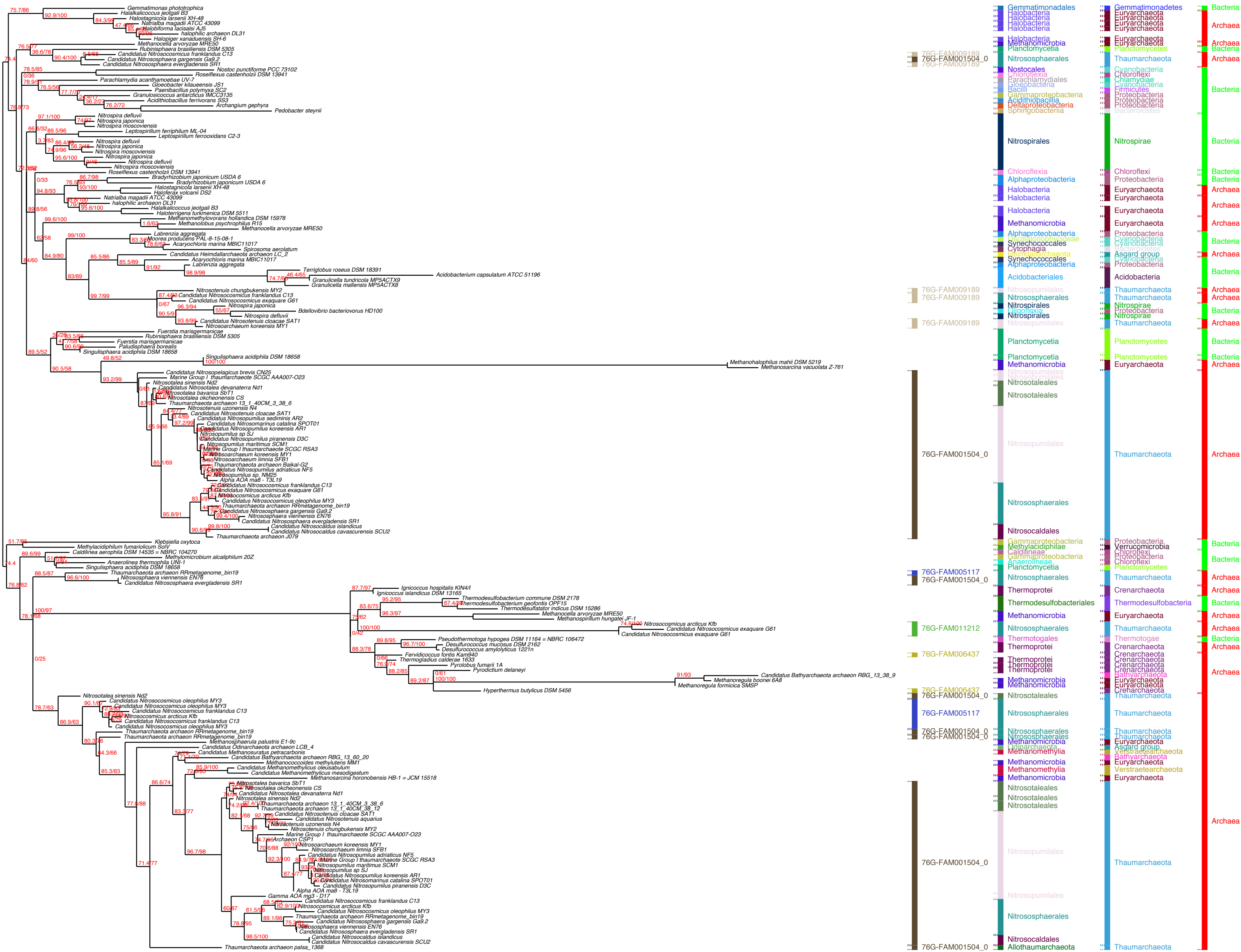

Gemmatimonas phototrophica
Halalkalicoccus jeotgali B3
Halostagnicola larsenii XH-48
Natrialba magadii ATCC 43099
Halobiforma lacisalsi AJ5
halophilic archaeon DL31
Halopiger xanaduensis SH-6
Methanocella arvoryzae MRE50
Rubinisphaera brasiliensis DSM 5305
Candidatus Nitrosocosmicus franklandus C13
Candidatus Nitrososphaera gargensis Ga9.2
Candidatus Nitrososphaera evergladensis SR1
Nostoc punctiforme PCC 73102
Roseiflexus castenholzii DSM 13941
Parachlamydia acanthamoebae UV-7
Gloeobacter kilaueensis JS1
Paenibacillus polymyxa SC2
Granulosicoccus antarcticus IMCC3135
Acidithiobacillus ferrivorans SS3
Archangium gephyra
Pedobacter steynii
Nitrospira defluvii
Nitrospira japonica
Nitrospira moscoviensis
Leptospirillum ferriphilum ML-04
Leptospirillum ferrooxidans C2-3
Bradyrhizobium japonicum USDA 6
Haloferax volcanii DS2
Haloterrigena turkmenica DSM 5511
Methanomethylovorans hollandica DSM 15978
Methanolobus psychrophilus R15
Labrenzia aggregata
Moorea producens PAL-8-15-08-1
Acaryochloris marina MBIC11017
Spirosoma aerolatum
Candidatus Heimdallarchaeota archaeon LC_2
Terriglobus roseus DSM 18391
Acidobacterium capsulatum ATCC 51196
Granulicella tundricola MP5ACTX9
Granulicella mallensis MP5ACTX8
Nitrosotenuis chungbukensis MY2
Candidatus Nitrosocosmicus exaquare G61
Bdellovibrio bacteriovorus HD100
Candidatus Nitrosotenuis cloacae SAT1
Nitrosoarchaeum koreensis MY1
Fuerstia marisgermanicae
Paludisphaera borealis
Singulisphaera acidiphila DSM 18658
Methanohalophilus mahii DSM 5219
Methanosarcina vacuolata Z-761
Candidatus Nitrosopelagicus brevis CN25
Marine Group I thaumarchaeote SCGC AAA007-O23
Nitrosotalea sinensis Nd2
Candidatus Nitrosotalea devanaterra Nd1
Nitrosotalea bavarica SbT1
Nitrosotalea okcheonensis CS
Thaumarchaeota archaeon 13_1_40CM_3_38_6
Nitrosotenuis uzonensis N4
Candidatus Nitrosopumilus sediminis AR2
Candidatus Nitrosomarinus catalina SPOT01
Candidatus Nitrosopumilus koreensis AR1
Nitrosopumilus sp SJ
Candidatus Nitrosopumilus piranensis D3C
Nitrosopumilus maritimus SCM1
Marine Group I thaumarchaeote SCGC RSA3
Nitrosoarchaeum limnia SFB1
Thaumarchaeota archaeon Baikal-G2
Candidatus Nitrosopumilus adriaticus NF5
Nitrosopumilus sp. NM25
Alpha AOA ma8 - T3L19
Nitrosocosmicus arcticus Kfb
Candidatus Nitrosocosmicus oleophilus MY3
Thaumarchaeota archaeon RRmetagenome_bin19
Nitrososphaera viennensis EN76
Candidatus Nitrosocaldus islandicus
Candidatus Nitrosocaldus cavascurensis SCU2
Thaumarchaeota archaeon J079
Klebsiella oxytoca
Methylacidiphilum fumariolicum SolV
Caldilinea aerophila DSM 14535 = NBRC 104270
Methylomicrobium alcaliphilum 20Z
Anaerolinea thermophila UNI-1
Ignicoccus hospitalis KIN4/I
Ignicoccus islandicus DSM 13165
Thermodesulfobacterium commune DSM 2178
Thermodesulfobacterium geofontis OPF15
Thermodesulfatator indicus DSM 15286
Methanospirillum hungatei JF-1
Candidatus Nitrosocosmicus exaquare G61
Pseudothermotoga hypogea DSM 11164 = NBRC 106472
Desulfurococcus mucosus DSM 2162
Desulfurococcus amylolyticus 1221n
Fervidicoccus fontis Kam940
Thermogladius calderae 1633
Pyrolobus fumarii 1A
Pyrodictium delaneyi
Candidatus Bathyarchaeota archaeon RBG_13_38_9
Methanoregula boonei 6A8
Methanoregula formicica SMSP
Hyperthermus butylicus DSM 5456
Methanosphaerula palustris E1-9c
Candidatus Odinarchaeota archaeon LCB_4
Candidatus Methanosuratus petracarbonis
Candidatus Bathyarchaeota archaeon RBG_13_60_20
Methanococcoides methylutens MM1
Candidatus Methanomethylicus oleusabulum
Candidatus Methanomethylicus mesodigestum
Methanosarcina horonobensis HB-1 = JCM 15518
Candidatus Nitrosotalea devanaterra Nd1
Thaumarchaeota archaeon 13_1_40CM_38_12
Candidatus Nitrosotenuis aquarius
Archaeon CSP1
Gamma AOA mg3 - D17
Thaumarchaeota archaeon palsa_1368
76G-FAM009189
76G-FAM001504_0
76G-FAM005117
76G-FAM011212
76G-FAM006437
Gemmatimonadales
Halobacteria
Methanomicrobia
Planctomycetia
Nitrososphaerales
Nostocales
Chloroflexia
Parachlamydiales
Gloeobacteria
Bacilli
Gammaproteobacteria
Acidithiobacillia
Deltaproteobacteria
Sphingobacteriia
Nitrospirales
Alphaproteobacteria
Oscillatoriophycideae
Synechococcales
Cytophagia
Heimdallarchaeota
Acidobacteriales
Oligoflexia
Nitrosopumilales
Nitrosotaleales
Nitrosocaldales
Methylacidiphilae
Caldilineae
Anaerolineae
Thermoprotei
Thermodesulfobacteriales
Thermotogales
Odinarchaeota
Methanomethylia
Allothaumarchaeota
Gemmatimonadetes
Euryarchaeota
Planctomycetes
Thaumarchaeota
Cyanobacteria
Chloroflexi
Chlamydiae
Firmicutes
Proteobacteria
Bacteroidetes
Nitrospirae
Asgard group
Acidobacteria
Verrucomicrobia
Crenarchaeota
Thermodesulfobacteria
Thermotogae
Bathyarchaeota
Verstraetearchaeota
Bacteria
Archaea

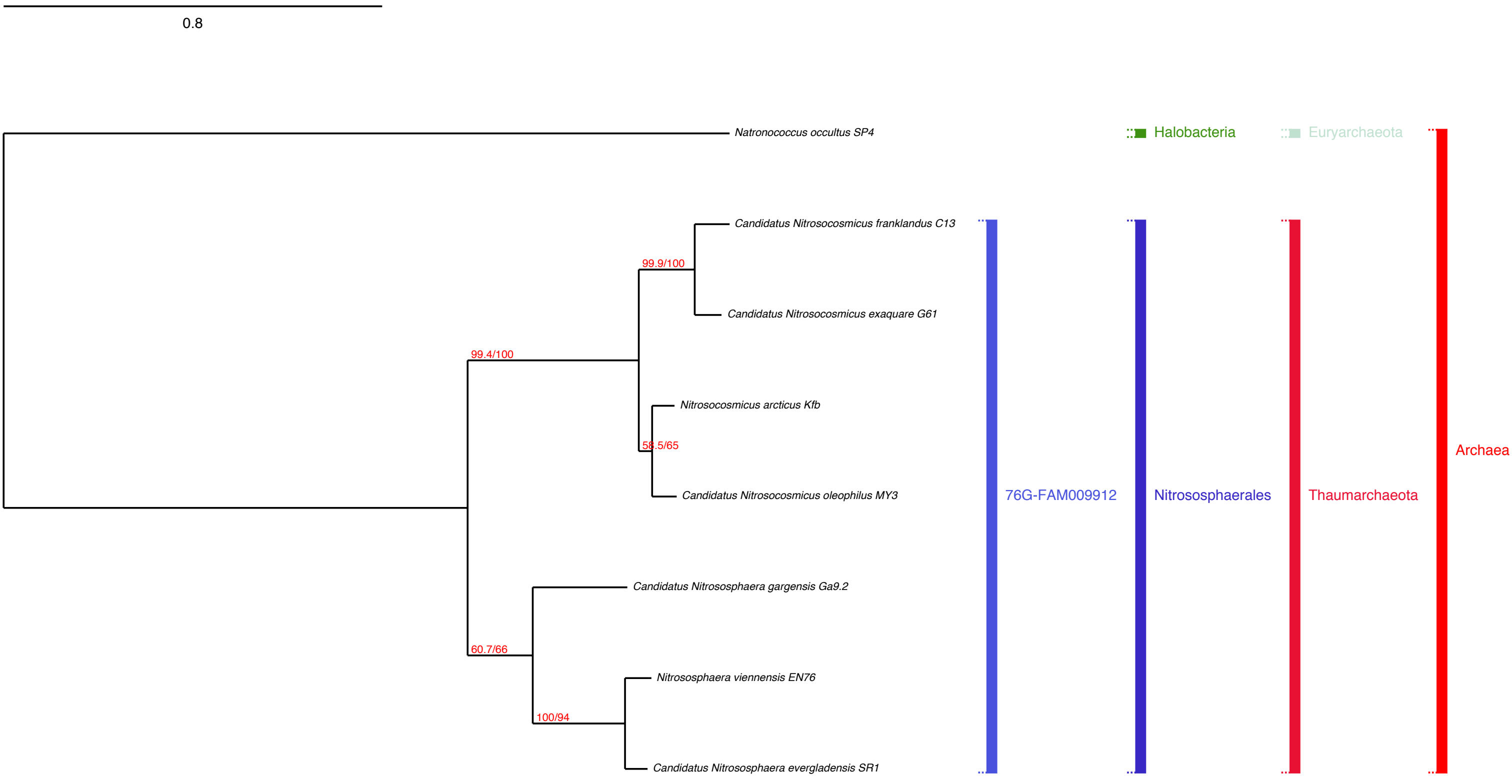

0.8
Natronococcus occultus SP4
Halobacteria
Euryarchaeota
Candidatus Nitrosocosmicus franklandus C13
99.9/100
Candidatus Nitrosocosmicus exaquare G61
99.4/100
Nitrosocosmicus arcticus Kfb
58.5/65
Candidatus Nitrosocosmicus oleophilus MY3
76G-FAM009912
Nitrososphaerales
Thaumarchaeota
Archaea
Candidatus Nitrososphaera gargensis Ga9.2
60.7/66
Nitrososphaera viennensis EN76
100/94
Candidatus Nitrososphaera evergladensis SR1

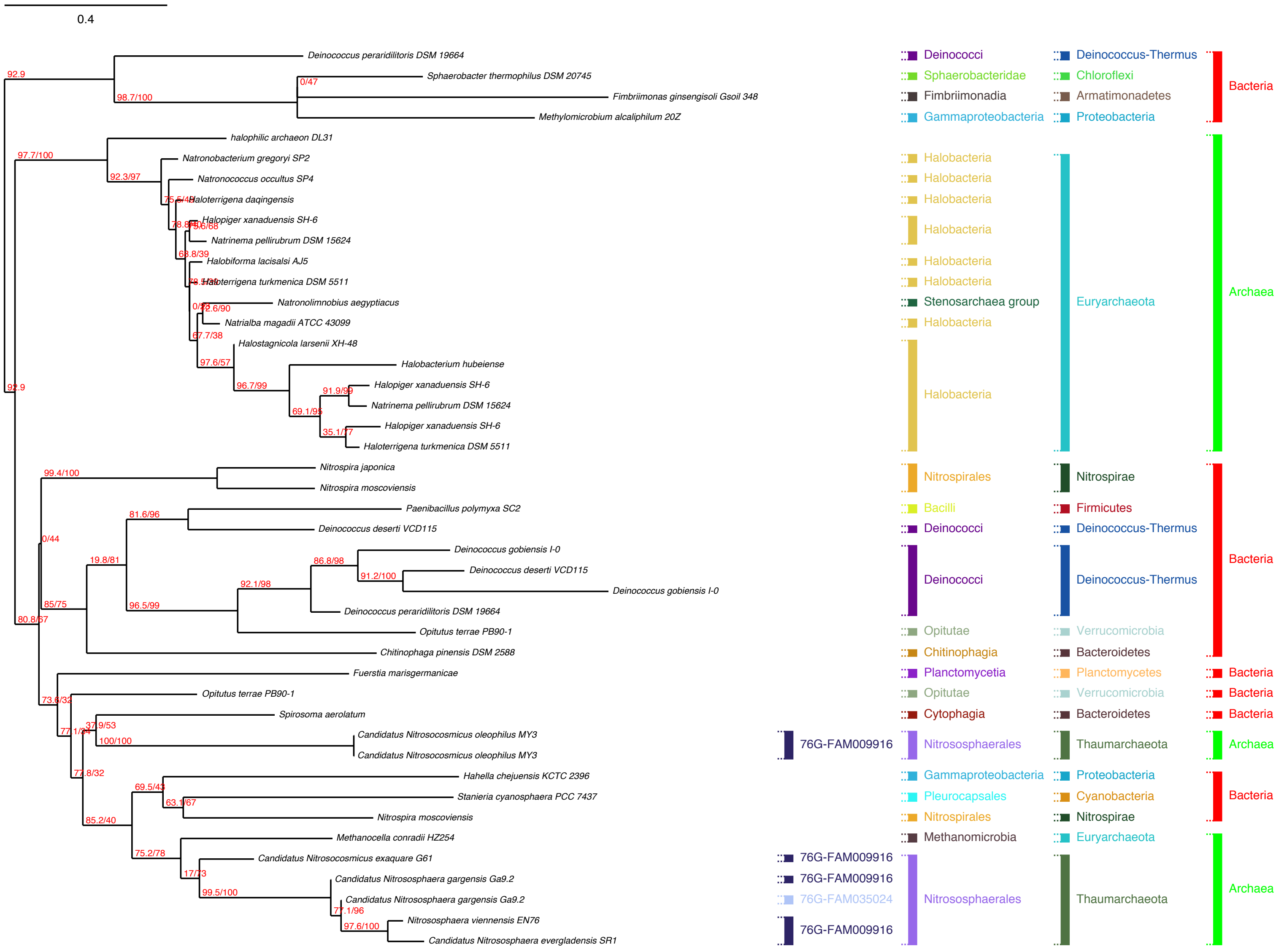

0.4
Deinococcus perarídilitoris DSM 19664
Sphaerobacter thermophilus DSM 20745
Fimbriimonas ginsengisoli Gsoil 348
Methylomicrobium alcaliphilum 20Z
halophilic archaeon DL31
Natronobacterium gregoryi SP2
Natronococcus occultus SP4
Haloterrigena daqingensis
Halopiger xanaduensis SH-6
Natrinema pellirubrum DSM 15624
Halobiforma lacisalsi AJ5
Haloterrigena turkmenica DSM 5511
Natronolimnobius aegyptiacus
Natrialba magadii ATCC 43099
Halostagnicola larsenii XH-48
Halobacterium hubeiense
Halopiger xanaduensis SH-6
Natrinema pellirubrum DSM 15624
Halopiger xanaduensis SH-6
Haloterrigena turkmenica DSM 5511
Nitrospira japonica
Nitrospira moscoviensis
Paenibacillus polymyxa SC2
Deinococcus deserti VCD115
Deinococcus gobiensis I-0
Deinococcus deserti VCD115
Deinococcus gobiensis I-0
Deinococcus peraridilitoris DSM 19664
Opitutus terrae PB90-1
Chitinophaga pinensis DSM 2588
Fuerstia marisgermanicae
Opitutus terrae PB90-1
Spirosoma aerolatum
Candidatus Nitrosocosmicus oleophilus MY3
Candidatus Nitrosocosmicus oleophilus MY3
Hahella chejuensis KCTC 2396
Stanieria cyanosphaera PCC 7437
Nitrospira moscoviensis
Methanocella conradii HZ254
Candidatus Nitrosocosmicus exaquare G61
Candidatus Nitrososphaera gargensis Ga9.2
Candidatus Nitrososphaera gargensis Ga9.2
Nitrososphaera viennensis EN76
Candidatus Nitrososphaera evergladensis SR1
Deinococci
Deinococcus-Thermus
Sphaerobacteridae
Chloroflexi
Fimbriimonadia
Armatimonadetes
Gammaproteobacteria
Proteobacteria
Bacteria
Halobacteria
Halobacteria
Halobacteria
Halobacteria
Halobacteria
Halobacteria
Stenosarchaea group
Euryarchaeota
Halobacteria
Halobacteria
Archaea
Nitrospirales
Nitrospirae
Bacilli
Firmicutes
Deinococci
Deinococcus-Thermus
Deinococci
Deinococcus-Thermus
Bacteria
Opitutae
Verrucomicrobia
Chitinophagia
Bacteroidetes
Planctomycetia
Planctomycetes
Bacteria
Opitutae
Verrucomicrobia
Bacteria
Cytophagia
Bacteroidetes
Bacteria
76G-FAM009916
Nitrososphaerales
Thaumarchaeota
Archaea
Gammaproteobacteria
Proteobacteria
Pleurocapsales
Cyanobacteria
Bacteria
Nitrospirales
Nitrospirae
Methanomicrobia
Euryarchaeota
76G-FAM009916
76G-FAM009916
76G-FAM035024
76G-FAM009916
Nitrososphaerales
Thaumarchaeota
Archaea

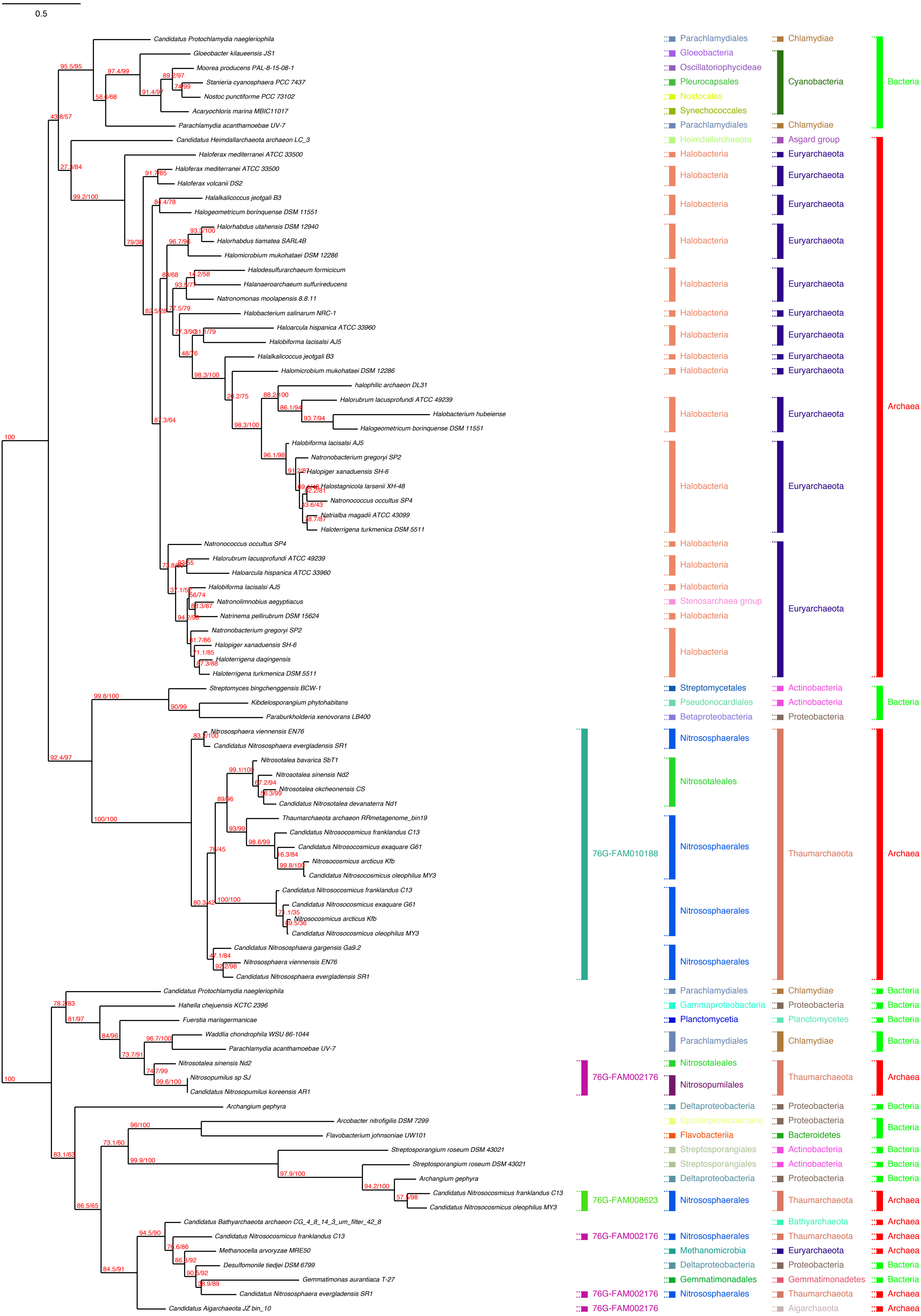

0.5
Candidatus Protochlamydia naegleriophila
Gloeobacter kilaueensis JS1
Moorea producens PAL-8-15-08-1
Stanieria cyanosphaera PCC 7437
Nostoc punctiforme PCC 73102
Acaryochloris marina MBIC11017
Parachlamydia acanthamoebae UV-7
Candidatus Heimdallarchaeota archaeon LC_3
Haloferax mediterranei ATCC 33500
Haloferax mediterranei ATCC 33500
Haloferax volcanii DS2
Halalkalicoccus jeotgali B3
Halogeometricum borinquense DSM 11551
Halorhabdus utahensis DSM 12940
Halorhabdus tiamatea SARL4B
Halomicrobium mukohataei DSM 12286
Halodesulfurarchaeum formicicum
Halanaeroarchaeum sulfurireducens
Natronomonas moolapensis 8.8.11
Halobacterium salinarum NRC-1
Haloarcula hispanica ATCC 33960
Halobiforma lacisalsi AJ5
Halalkalicoccus jeotgali B3
Halomicrobium mukohataei DSM 12286
halophilic archaeon DL31
Halorubrum lacusprofundi ATCC 49239
Halobacterium hubeiense
Halogeometricum borinquense DSM 11551
Halobiforma lacisalsi AJ5
Natronobacterium gregoryi SP2
Halopiger xanaduensis SH-6
Halostagnicola larsenii XH-48
Natronococcus occultus SP4
Natrialba magadii ATCC 43099
Haloterrigena turkmenica DSM 5511
Natronococcus occultus SP4
Halorubrum lacusprofundi ATCC 49239
Haloarcula hispanica ATCC 33960
Halobiforma lacisalsi AJ5
Natronolimnobius aegyptiacus
Natrinema pellirubrum DSM 15624
Natronobacterium gregoryi SP2
Halopiger xanaduensis SH-6
Haloterrigena daqingensis
Haloterrigena turkmenica DSM 5511
Streptomyces bingchenggensis BCW-1
Kibdelosporangium phytohabitans
Paraburkholderia xenovorans LB400
Nitrososphaera viennensis EN76
Candidatus Nitrososphaera evergladensis SR1
Nitrosotalea bavarica SbT1
Nitrosotalea sinensis Nd2
Nitrosotalea okcheonensis CS
Candidatus Nitrosotalea devanaterra Nd1
Thaumarchaeota archaeon RRmetagenome_bin19
Candidatus Nitrosocosmicus franklandus C13
Candidatus Nitrosocosmicus exaquare G61
Nitrosocosmicus arcticus Kfb
Candidatus Nitrosocosmicus oleophilus MY3
Candidatus Nitrosocosmicus franklandus C13
Candidatus Nitrosocosmicus exaquare G61
Nitrosocosmicus arcticus Kfb
Candidatus Nitrosocosmicus oleophilus MY3
Candidatus Nitrososphaera gargensis Ga9.2
Nitrososphaera viennensis EN76
Candidatus Nitrososphaera evergladensis SR1
Candidatus Protochlamydia naegleriophila
Hahella chejuensis KCTC 2396
Fuerstia marisgermanicae
Waddlia chondrophila WSU 86-1044
Parachlamydia acanthamoebae UV-7
Nitrosotalea sinensis Nd2
Nitrosopumilus sp SJ
Candidatus Nitrosopumilus koreensis AR1
Archangium gephyra
Arcobacter nitrofigilis DSM 7299
Flavobacterium johnsoniae UW101
Streptosporangium roseum DSM 43021
Streptosporangium roseum DSM 43021
Archangium gephyra
Candidatus Nitrosocosmicus franklandus C13
Candidatus Nitrosocosmicus oleophilus MY3
Candidatus Bathyarchaeota archaeon CG_4_8_14_3_um_filter_42_8
Candidatus Nitrosocosmicus franklandus C13
Methanocella arvoryzae MRE50
Desulfomonile tiedjei DSM 6799
Gemmatimonas aurantiaca T-27
Candidatus Nitrososphaera evergladensis SR1
Candidatus Aigarchaeota JZ bin_10
76G-FAM010188
76G-FAM002176
76G-FAM008623
76G-FAM002176
76G-FAM002176
76G-FAM002176
Parachlamydiales
Gloeobacteria
Oscillatoriophycideae
Pleurocapsales
Nostocales
Synechococcales
Parachlamydiales
Heimdallarchaeota
Halobacteria
Stenosarchaea group
Streptomycetales
Pseudonocardiales
Betaproteobacteria
Nitrososphaerales
Nitrosotaleales
Gammaproteobacteria
Planctomycetia
Nitrosopumilales
Deltaproteobacteria
Epsilonproteobacteria
Flavobacteriia
Streptosporangiales
Bathyarchaeota
Methanomicrobia
Gemmatimonadales
Chlamydiae
Cyanobacteria
Asgard group
Euryarchaeota
Actinobacteria
Proteobacteria
Thaumarchaeota
Planctomycetes
Bacteroidetes
Gemmatimonadetes
Aigarchaeota
Bacteria
Archaea

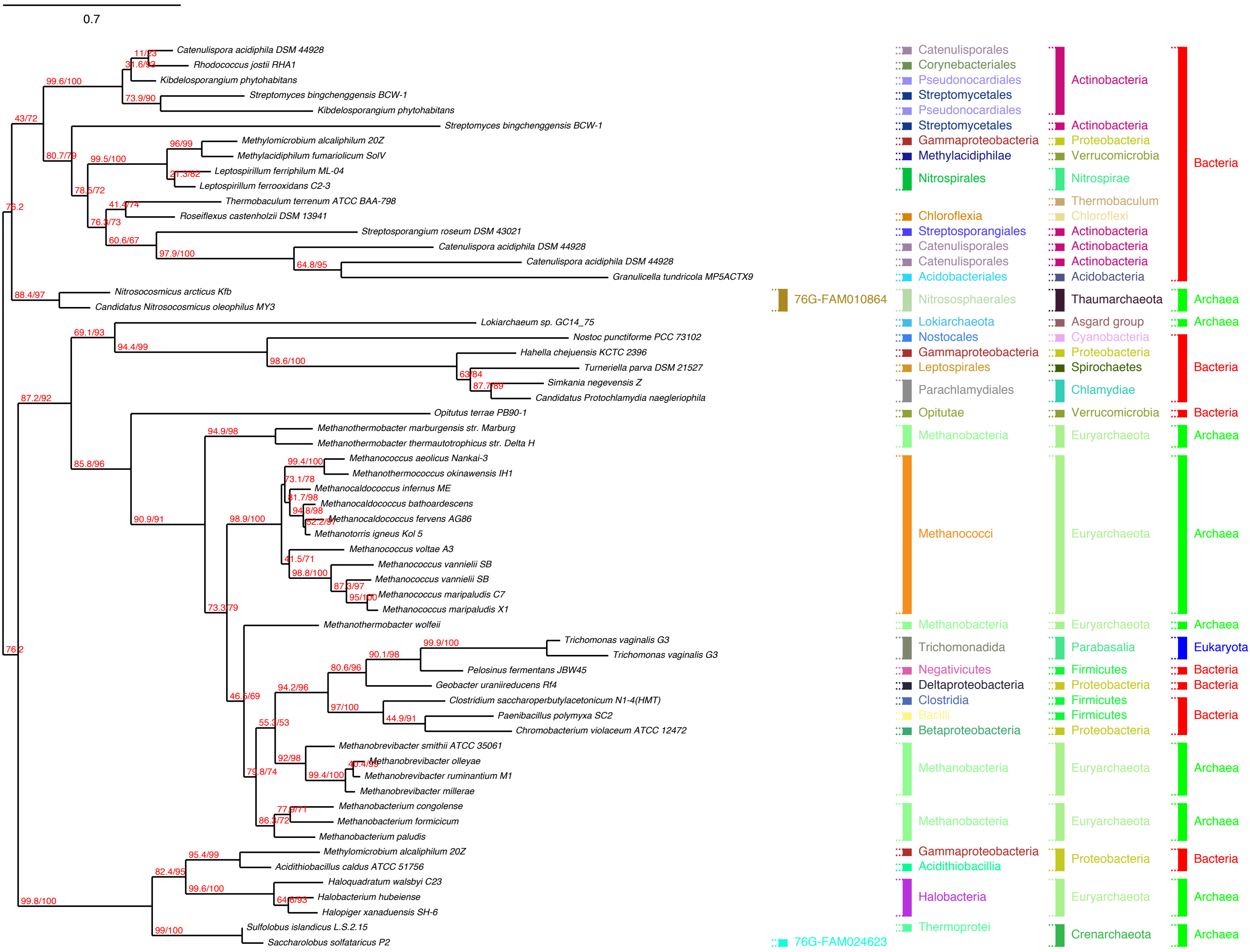
0.7
Catenulispora acidiphila DSM 44928
Rhodococcus jostii RHA1
Kibdelosporangium phytohabitans
Streptomyces bingchenggensis BCW-1
Kibdelosporangium phytohabitans
Streptomyces bingchenggensis BCW-1
Methylomicrobium alcaliphilum 20Z
Methylacidiphilum fumariolicum SolV
Leptospirillum ferriphilum ML-04
Leptospirillum ferrooxidans C2-3
Thermobaculum terrenum ATCC BAA-798
Roseiflexus castenholzii DSM 13941
Streptosporangium roseum DSM 43021
Catenulispora acidiphila DSM 44928
Catenulispora acidiphila DSM 44928
Granulicella tundricola MP5ACTX9
Nitrosocosmicus arcticus Kfb
Candidatus Nitrosocosmicus oleophilus MY3
Lokiarchaeum sp. GC14_75
Nostoc punctiforme PCC 73102
Hahella chejuensis KCTC 2396
Turneriella parva DSM 21527
Simkania negevensis Z
Candidatus Protochlamydia naegleriophila
Opitutus terrae PB90-1
Methanothermobacter marburgensis str. Marburg
Methanothermobacter thermautotrophicus str. Delta H
Methanococcus aeolicus Nankai-3
Methanothermococcus okinawensis IH1
Methanocaldococcus infernus ME
Methanocaldococcus bathoardescens
Methanocaldococcus fervens AG86
Methanotorris igneus Kol 5
Methanococcus voltae A3
Methanococcus vannielii SB
Methanococcus vannielii SB
Methanococcus maripaludis C7
Methanococcus maripaludis X1
Methanothermobacter wolfeii
Trichomonas vaginalis G3
Trichomonas vaginalis G3
Pelosinus fermentans JBW45
Geobacter uraniireducens Rf4
Clostridium saccharoperbutylacetonicum N1-4(HMT)
Paenibacillus polymyxa SC2
Chromobacterium violaceum ATCC 12472
Methanobrevibacter smithii ATCC 35061
Methanobrevibacter olleyae
Methanobrevibacter ruminantium M1
Methanobrevibacter millerae
Methanobacterium congolense
Methanobacterium formicicum
Methanobacterium paludis
Methylomicrobium alcaliphilum 20Z
Acidithiobacillus caldus ATCC 51756
Haloquadratum walsbyi C23
Halobacterium hubeiense
Halopiger xanaduensis SH-6
Sulfolobus islandicus L.S.2.15
Saccharolobus solfataricus P2
11/83
31.6/93
99.6/100
73.9/90
43/72
80.7/79
96/99
99.5/100
21.3/82
78.5/72
41.4/74
76.3/73
60.6/67
97.9/100
64.8/95
76.2
88.4/97
69.1/93
94.4/99
98.6/100
63/84
87.7/89
87.2/92
94.9/98
99.4/100
73.1/78
81.7/98
94.8/98
52.2/97
98.9/100
90.9/91
85.8/96
41.5/71
98.8/100
87.3/97
95/100
73.3/79
99.9/100
90.1/98
80.6/96
94.2/96
97/100
44.9/91
46.6/69
55.3/53
92/98
40.4/99
99.4/100
79.8/74
77.9/71
86.3/72
76.2
95.4/99
82.4/95
99.6/100
64.6/93
99.8/100
99/100
76G-FAM010864
76G-FAM024623
Catenulisporales
Corynebacteriales
Pseudonocardiales
Streptomycetales
Pseudonocardiales
Streptomycetales
Gammaproteobacteria
Methylacidiphilae
Nitrospirales
Chloroflexia
Streptosporangiales
Catenulisporales
Catenulisporales
Acidobacteriales
Nitrososphaerales
Lokiarchaeota
Nostocales
Gammaproteobacteria
Leptospirales
Parachlamydiales
Opitutae
Methanobacteria
Methanococci
Methanobacteria
Trichomonadida
Negativicutes
Deltaproteobacteria
Clostridia
Bacilli
Betaproteobacteria
Methanobacteria
Methanobacteria
Gammaproteobacteria
Acidithiobacillia
Halobacteria
Thermoprotei
Actinobacteria
Actinobacteria
Proteobacteria
Verrucomicrobia
Nitrospirae
Thermobaculum
Chloroflexi
Actinobacteria
Actinobacteria
Actinobacteria
Acidobacteria
Thaumarchaeota
Asgard group
Cyanobacteria
Proteobacteria
Spirochaetes
Chlamydiae
Verrucomicrobia
Euryarchaeota
Euryarchaeota
Euryarchaeota
Parabasalia
Firmicutes
Proteobacteria
Firmicutes
Firmicutes
Proteobacteria
Euryarchaeota
Euryarchaeota
Proteobacteria
Euryarchaeota
Crenarchaeota
Bacteria
Archaea
Archaea
Bacteria
Bacteria
Archaea
Archaea
Archaea
Eukaryota
Bacteria
Bacteria
Bacteria
Archaea
Archaea
Bacteria
Archaea
Archaea

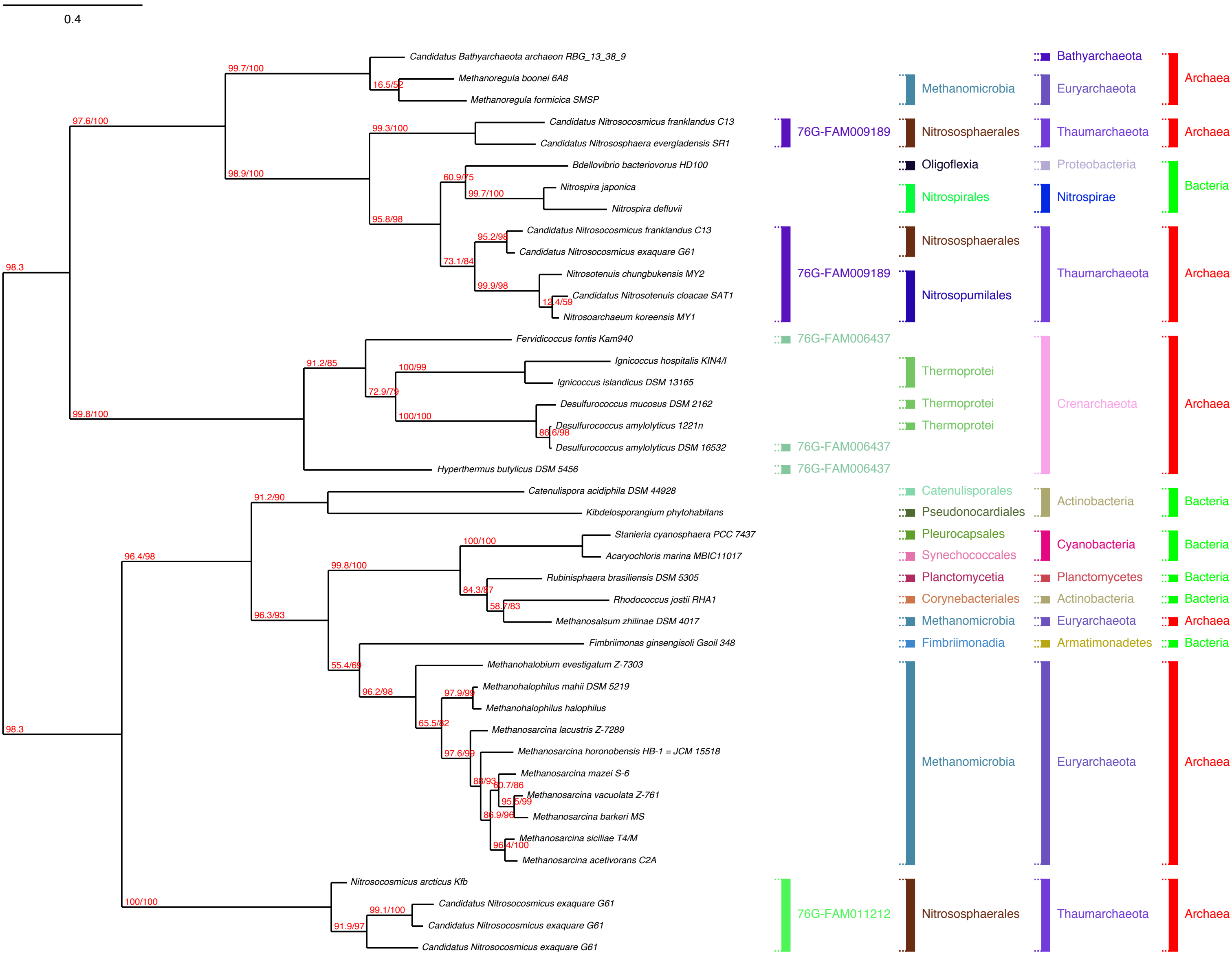
0.4
98.3
97.6/100
99.7/100
16.5/52
Candidatus Bathyarchaeota archaeon RBG_13_38_9
Methanoregula boonei 6A8
Methanoregula formicica SMSP
98.9/100
99.3/100
Candidatus Nitrosocosmicus franklandus C13
Candidatus Nitrososphaera evergladensis SR1
95.8/98
60.9/75
Bdellovibrio bacteriovorus HD100
99.7/100
Nitrospira japonica
Nitrospira defluvii
73.1/84
95.2/98
Candidatus Nitrosocosmicus franklandus C13
Candidatus Nitrosocosmicus exaquare G61
99.9/98
Nitrosotenuis chungbukensis MY2
12.4/59
Candidatus Nitrosotenuis cloacae SAT1
Nitrosoarchaeum koreensis MY1
99.8/100
91.2/85
Fervidicoccus fontis Kam940
72.9/79
100/99
Ignicoccus hospitalis KIN4/I
Ignicoccus islandicus DSM 13165
100/100
Desulfurococcus mucosus DSM 2162
86.6/98
Desulfurococcus amylolyticus 1221n
Desulfurococcus amylolyticus DSM 16532
Hyperthermus butylicus DSM 5456
98.3
96.4/98
91.2/90
Catenulispora acidiphila DSM 44928
Kibdelosporangium phytohabitans
96.3/93
99.8/100
100/100
Stanieria cyanosphaera PCC 7437
Acaryochloris marina MBIC11017
84.3/87
Rubinisphaera brasiliensis DSM 5305
58.7/83
Rhodococcus jostii RHA1
Methanosalsum zhilinae DSM 4017
55.4/69
Fimbriimonas ginsengisoli Gsoil 348
96.2/98
Methanohalobium evestigatum Z-7303
65.5/82
97.9/99
Methanohalophilus mahii DSM 5219
Methanohalophilus halophilus
97.6/99
Methanosarcina lacustris Z-7289
Methanosarcina horonobensis HB-1 = JCM 15518
88/93
60.7/86
Methanosarcina mazei S-6
95.5/99
Methanosarcina vacuolata Z-761
86.9/96
Methanosarcina barkeri MS
96.4/100
Methanosarcina siciliae T4/M
Methanosarcina acetivorans C2A
100/100
Nitrosocosmicus arcticus Kfb
91.9/97
99.1/100
Candidatus Nitrosocosmicus exaquare G61
Candidatus Nitrosocosmicus exaquare G61
Candidatus Nitrosocosmicus exaquare G61
76G-FAM009189
76G-FAM009189
76G-FAM006437
76G-FAM006437
76G-FAM006437
76G-FAM011212
Methanomicrobia
Nitrososphaerales
Oligoflexia
Nitrospirales
Nitrososphaerales
Nitrosopumilales
Thermoprotei
Thermoprotei
Thermoprotei
Catenulisporales
Pseudonocardiales
Pleurocapsales
Synechococcales
Planctomycetia
Corynebacteriales
Methanomicrobia
Fimbriimonadia
Methanomicrobia
Nitrososphaerales
Bathyarchaeota
Euryarchaeota
Thaumarchaeota
Proteobacteria
Nitrospirae
Thaumarchaeota
Crenarchaeota
Actinobacteria
Cyanobacteria
Planctomycetes
Actinobacteria
Euryarchaeota
Armatimonadetes
Euryarchaeota
Thaumarchaeota
Archaea
Archaea
Bacteria
Archaea
Archaea
Bacteria
Bacteria
Bacteria
Bacteria
Archaea
Bacteria
Archaea
Archaea

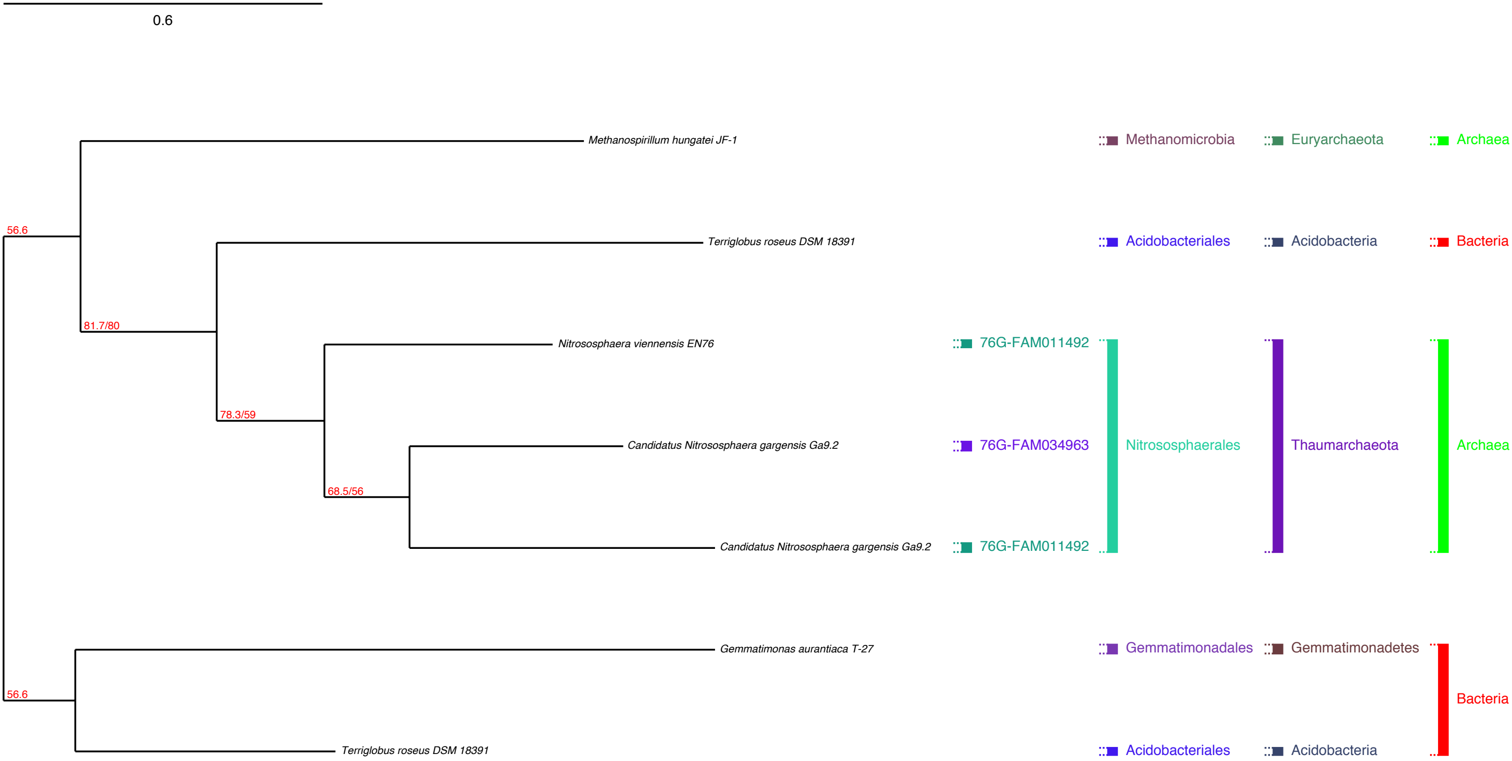
0.6
56.6
81.7/80
78.3/59
68.5/56
56.6
Methanospirillum hungatei JF-1
Terriglobus roseus DSM 18391
Nitrososphaera viennensis EN76
Candidatus Nitrososphaera gargensis Ga9.2
Candidatus Nitrososphaera gargensis Ga9.2
Gemmatimonas aurantiaca T-27
Terriglobus roseus DSM 18391
76G-FAM011492
76G-FAM034963
76G-FAM011492
Methanomicrobia
Acidobacteriales
Nitrososphaerales
Gemmatimonadales
Acidobacteriales
Euryarchaeota
Acidobacteria
Thaumarchaeota
Gemmatimonadetes
Acidobacteria
Archaea
Bacteria
Archaea
Bacteria

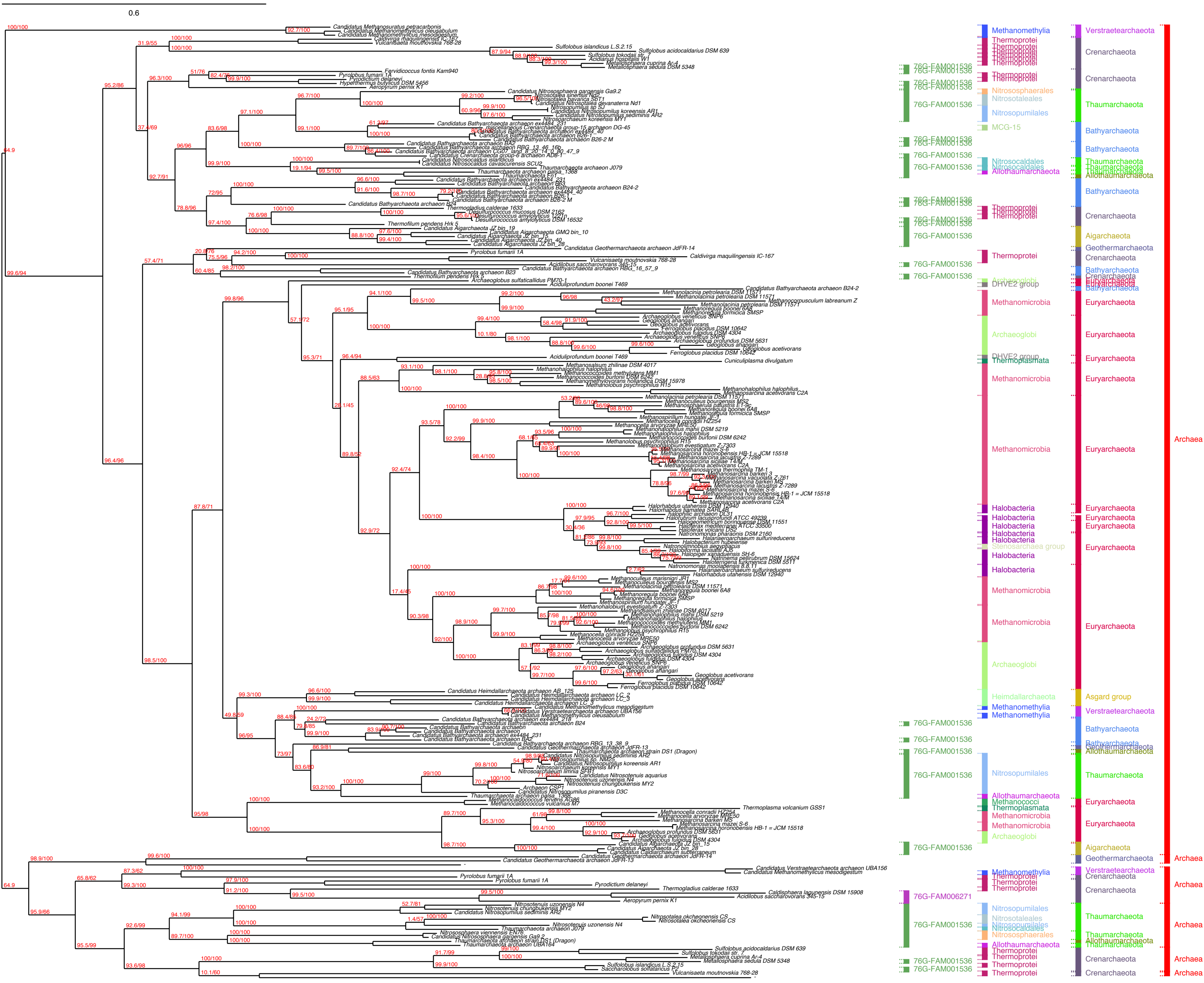
0.6
Methanomethylia
Thermoprotei
Nitrososphaerales
Nitrosotaleales
Nitrosopumilales
MCG-15
Nitrosocaldales
Allothaumarchaeota
Archaeoglobi
DHVE2 group
Methanomicrobia
Thermoplasmata
Halobacteria
Stenosarchaea group
Heimdallarchaeota
Methanococci
Verstraetearchaeota
Crenarchaeota
Thaumarchaeota
Bathyarchaeota
Aigarchaeota
Geothermarchaeota
Euryarchaeota
Asgard group
76G-FAM001536
76G-FAM006271
Archaea
